# International Society of Paediatric Surgical Oncology (IPSO) Surgical Practice Guidelines

**DOI:** 10.3332/ecancer.2022.1356

**Published:** 2022-02-17

**Authors:** Simone de Campos Vieira Abib, Chan Hon Chui, Sharon Cox, Abdelhafeez H Abdelhafeez, Israel Fernandez-Pineda, Ahmed Elgendy, Jonathan Karpelowsky, Pablo Lobos, Marc Wijnen, Jörg Fuchs, Andrea Hayes, Justin T Gerstle

**Affiliations:** 1 Pediatric Oncology Institute, GRAACC, Federal University of São Paulo, Rua Pedro de Toledo, 572 - Vila Clementino, São Paulo, SP 04021-001, Brazil; 2 Surgery Centre for Children, Mount Elizabeth Medical Centre, 3 Mount Elizabeth, 228510, Singapore; 3 Division of Paediatric Surgery, Red Cross War Memorial Children’s Hospital, University of Cape Town, Cape Town, South Africa; 4 Department of Surgery, St Jude Research Hospital 262 Danny Thomas Place. MS133, Memphis, TN 38105, USA; 5 Department of Pediatric Surgery, Virgen del Rocio Children’s Hospital, Av Manuel Siurot S/NN, Sevilla 41013, Spain; 6 Surgical Oncology Unit, Faculty of Medicine, Tanta University, Elgiesh Street, 31111, Tanta, Gharbeya, Egypt; 7 Department of Paediatric Surgery, Children’s Hospital at Westmead, Westmead NSW 2145, Australia; 8 Pediatric Surgery Division, Hospital Italiano de Buenos Aires, Andrés Lamas 812, Buenos Aires 1406, Argentina; 9 Department of Surgery, Princess Maxima Center for Pediatric Oncology, Huispostnummer KE 01.129.2, Postbus 85090, Utretcht 3508AB, The Netherlands; 10 Department of Pediatric Surgery and Pediatric Urology, University of Tuebingen, Hoppe-Seyler-Str. 3, Tübingen 72076, Germany; 11 Department of Surgery, Howard University Hospital, 1851 9th Street NW, 4th Floor, Washington, DC 20059, USA; 12 Department of Surgery, Memorial Sloan Kettering Cancer Center, 1275 York Avenue, New York, NY 10065, USA

**Keywords:** paediatric oncology surgery, paediatric cancer, surgery, children

## Abstract

Most children with tumors will require one or more surgical interventions as part of the care and treatment, including making a diagnosis, obtaining adequate venous access, performing a surgical resection for solid tumors (with staging and reconstruction), performing procedures for cancer prevention and its late effects, and managing complications of treatment; all with the goal of improving survival and quality of life. It is important for surgeons to adhere to sound pediatric surgical oncology principles, as they are closely associated with improved local control and survival. Unfortunately, there is a significant disparity in survival rates in low and middle income countries, when compared to those from high income countries.

The International Society of Paediatric Surgical Oncology (IPSO) is the leading organization that deals with pediatric surgical oncology worldwide. This organization allows experts in the field from around the globe to gather and address the surgical needs of children with cancer. IPSO has been invited to contribute surgical guidance as part of the World Health Organization Initiative for Childhood Cancer. One of our goals is to provide surgical guidance for different scenarios, including those experienced in High- (HICs) and Low- and Middle-Income Countries (LMICs). With this in mind, the following guidelines have been developed by authors from both HICs and LMICs. These have been further validated by experts with the aim of providing evidence-based information for surgeons who care for children with cancer.

We hope that this initiative will benefit children worldwide in the best way possible.

Simone Abib, IPSO President

Justin T Gerstle, IPSO Education Committee Chair

Chan Hon Chui, IPSO Secretary

## Introduction

Paediatric oncology surgeons play a critical role in diagnosing, staging and treating malignant solid tumours. Over the years, a more tailored surgical approach of the primary tumour site and the metastatic disease has been advocated by many solid tumour protocols [1]. Whether to perform an upfront tumour resection at diagnosis or a biopsy followed by neoadjuvant therapy is a critical decision that a multidisciplinary paediatric oncology team needs to make based on clinical, radiological and histological aspects. Unnecessary upfront resections can lead to short- and long-term morbidity, an incomplete tumour resection and may be associated with a delay in the initiation of adjuvant therapy.

The differential diagnosis of a solid mass is strongly influenced by the patient’s age, anatomic site, organ of origin, gender, race, presence of cancer predisposition syndromes and certain infectious agents. Since some paediatric malignancies are associated with the elevation of tumour markers, including alpha-fetoprotein (AFP), beta-human chorionic gonadotropin (βHCG), urinary catecholamine and certain hormones, a dedicated laboratory workup may help to establish the diagnosis.

Diagnosis of malignancy may be obtained from the primary tumour or its metastatic sites. Therefore, it is critical to recognise the pattern of disease dissemination for each histological subtype. The least invasive diagnostic procedure should be considered to establish a diagnosis if, following oncological guidelines, a complete resection of the primary is not possible [2]. Bone marrow aspirates and biopsies can be helpful for tumour subtypes that metastasise to the bone marrow including neuroblastoma, lymphoma, Ewing sarcoma family of tumours (ESFT) and rhabdomyosarcoma (RMS). Similarly, enlarged peripheral lymph nodes may be a good source of diagnostic tissue. This is particularly important for patients with a mediastinal mass, airway compromise and a high suspicion for lymphoma [3]. Finally, biopsy of visceral metastatic sites such as lung nodules may also help to establish tumour histology and confirm the presence of metastatic disease in certain histology entities like osteosarcoma as it eliminates inevitable local dissemination of the primary tumour and decreases the risk of pathologic fractures. Here we review the role of surgery in the diagnostic management of childhood solid tumours (Please refer to *Neuroblastoma*, and *Rhabdomyosarcoma and Non-Rhabdomyosarcoma Soft Tissue Sarcoma Guidelines*).

## Abdominal Mass

**Adrenal tumour** (Please refer to *Neuroblastoma and Rare Tumours Guidelines*)

The most common malignant adrenal tumour in children is neuroblastoma. It remains the most common extracranial solid tumour in children with an incidence of approximately 1 per 100,000 children per year [4]. The surgical management of neuroblastoma is based on the risk stratification of the patient. Localised adrenal masses without other associated findings detected in the perinatal period, although may not require any therapy, still warrant careful follow-up [5, 6]. Complete tumour resection is considered sufficient in the group of patients with a localised (L) primary tumour without image-defined risk factors (IDRFs) and negative metastatic work-up [7] in the absence of other high-risk factors. Those patients are categorised as L1 and are eligible for upfront tumour resection which is both diagnostic and therapeutic. Resection, when performed as the initial intervention, may obviate the need for chemotherapy as many of these patients will have low-risk disease and have an excellent prognosis.

Unfortunately, more than one-half of the patients with neuroblastoma present with advanced local (L2) or metastatic (M) disease. Those patients are better treated with initial diagnostic tumour biopsy to be followed by neoadjuvant chemotherapy if needed. Obtaining adequate tissue for diagnosis of neuroblastoma may be challenging due to the amount of histological and molecular tests that are necessary for a correct risk stratification. Therefore, small tissue samples may not always be fully diagnostic. A multidisciplinary discussion among surgeons, interventional radiologists and pathologists is critical in deciding the best approach to obtain adequate tissue for diagnosis. Diagnostic modalities include open tumour biopsy, laparoscopic or thoracoscopic tumour biopsy, image-guided (ultrasound, computed tomography (CT)) biopsy, bone marrow biopsy (rarely sufficient for performing all the molecular required studies) or biopsy of metastatic disease (pathologic enlarged lymph nodes, soft tissue masses including skin nodules, cortical bone lesions, liver metastases). The decision on the best surgical approach for diagnosis of stage L2 and M neuroblastoma is based on the disease pattern characteristics and the local resources of each institution. For patients with stage L1 who undergo upfront surgery, the extent of resection needed is uncertain [8].

Although rare, it is critical to take into consideration pheochromocytoma and adrenocortical tumours in the differential diagnosis of an adrenal mass when considering a possible biopsy, because adrenocortical carcinoma and malignant pheochromocytoma will be upstaged by performing a biopsy [9]. These tumours are generally chemo resistant and are better treated with upfront resection and local staging. Clinical signs and symptoms including patient age, hypertension, palpitations, hyperglycaemia, virilisation and hormonal lab studies for neuroblastoma may help to guide the diagnosis [10, 11] (Please refer to *Rare Tumours Guideline*).

**Renal tumour** (Please refer to *Wilms Tumour Guidelines*)

Although the International Society of Paediatric Oncology (SIOP) protocol calls for neoadjuvant chemotherapy without tissue confirmation of Wilms and the Children’s Oncology Group (COG) continues to advocate upfront surgery, there are instances, albeit different in each protocol, when biopsy of the primary is called for. The argument for biopsy of the primary tumour is similar but the timing and subsequent steps vary between the SIOP [12] and COG [13] protocols in both unilateral and in bilateral tumours. In a child with unilateral kidney tumour outside the age range where Wilms is most common or when the radiologic findings are inconclusive or suggestive of other than nephroblastoma, both exceptions either alone or in combination, the SIOP protocol, is one opportunity where upfront percutaneous retroperitoneal primary tumour biopsy becomes justifiable vis a vis upfront nephroureterectomy or empiric neoadjuvant chemotherapy. An important exception would be newborns and infants younger than 6 months where upfront nephrectomy remains the standard [14]. It is important to emphasise that the best option would be the one decided collectively by the managing multi-disciplinary team. Another situation when percutaneous biopsy should be considered is the absence of tumour response or progression during therapy. Again, discussion by the multidisciplinary team is of paramount importance and nephroureterectomy should be weighed in.

The COG protocol, which advocates upfront primary tumour resection for all children, provides a window for kidney biopsy to justify neoadjuvant chemotherapy [15] and it includes; A primary renal tumour with a tumour thrombus above the level of the hepatic veins [13] pulmonary compromise from a massive primary or extensive pulmonary metastases [13] when resection requires removal of contiguous structures (other than adrenal gland) or individual surgeons’ judgment stating that attempting nephrectomy would result in significant morbidity [15], tumour spill or residual tumour. Nevertheless, the COG continues to recommend for all patients to undergo initial exploration to assess operability as neoadjuvant chemotherapy does not result in improved survival rates and results in the loss of important staging information.

Open or laparoscopic primary tumour biopsy is discouraged by both protocols as it upstages the tumour. The only exception would be when a tumour is considered unresectable during an actual attempt at upfront nephrectomy.

In the setting of bilateral kidney tumours, the SIOP protocol does not require tissue diagnosis to initiate neoadjuvant chemotherapy. This is based on the extreme rarity of bilateral tumours other than Wilms. Biopsy, however, should be considered when there is no response or progression while receiving neoadjuvant chemotherapy. This applies to unilateral and bilateral renal tumours. Similarly, the COG protocol recognises that biopsy is not necessary before initiating chemotherapy in most children with bilateral Wilms tumours (BWTs). It is strongly recommended in cases where there are unusual features (e.g. older than 8 years and atypical intra-abdominal findings).

In 20% of BWT cases, the pathology is not the same bilaterally. Therefore, when deciding on biopsy, it is important to sample both kidneys and since anaplasia cannot be detected on percutaneous or core-needle biopsies, open biopsy remains an option.

**Liver tumour** (Please refer to *Hepatoblastoma and Hepatocellular Carcinoma Guidelines*)

Liver tumours are rare in children, accounting for 1% of all paediatric malignancies. Two distinct entities, hepatoblastoma (HBL) and hepatocellular carcinoma (HCC), are seen in this age group. While HBL is typically diagnosed in children younger than 4 years of age (usually younger than 2), HCC occurs in older children and adolescents. AFP is usually elevated in both histologies (>90% of patients with HBL and 60% of patients with HCC) [16].

HBL is considered to be resectable in 30%–50% of newly diagnosed patients [17]. Historically, the COG has recommended upfront resection for resectable tumours without a biopsy. However, if a gross total resection is not likely to be achieved, a primary resection should not be attempted as these tumours are very chemosensitive and a more complete resection is likely possible after neoadjuvant cisplatin-based chemotherapy [18]. The International Society of Paediatric Oncology Epithelial Liver Tumour Group (SIOPEL) study for management of liver tumours in Europe has traditionally recommended initial tumour biopsy followed by neoadjuvant chemotherapy and delayed resection. In an attempt to treat HBL cases following the same guidelines worldwide, a collaborative trial involving the major clinical groups running paediatric liver tumour trials, SIOPEL, the Liver Tumour Committee of the COG, the Japanese Children’s Cancer Group and the Society for Paediatric Oncology and Haematology, Germany has been designed. This collaborative trial has been designated as Paediatric Hepatic International Tumour Trial and the primary objectives are: 1) evaluation if the treatment of low-risk HBL can be reduced, 2) comparison of different treatment regimens for intermediate-risk HBL and 3) comparison of different post induction treatment regimens for high-risk HBL. Surgical candidates for upfront resection include pretreatment extent of tumour PRETEXT I (a tumour that involves only one section) and II (a tumour that involves two sections) and >1 cm radiographic margin on the middle hepatic vein, the retro-hepatic inferior vena cava (IVC) and or portal bifurcation. Non-surgical candidates for upfront resection undergo tumour biopsy followed by neoadjuvant chemotherapy. Biopsy may be performed by open or laparoscopic approach, although the ultrasound-guided biopsy is preferred to avoid any delay in chemotherapy initiation. Obtaining a biopsy at diagnosis does not automatically upstage a patient if subsequent complete resection is performed at the time of the definitive surgery [19].

### Pelvic tumour

Diagnosis of a pelvic tumour can be challenging, and the diagnostic work-up can be influenced by the age of the patient and the characteristics of the mass. Pelvic bony lesions are not the focus of this chapter.

In young girls, pelvic tumours are mainly represented by ovarian tumours (Please refer to *Germ Cell Tumours – Ovarian Tumours Guidelines and Non-GCT ovarian tumours*). Although mostly benign, retrospective case series reports an incidence of malignancy in 10%–20% of cases. The ovarian mass can be an incidental finding on examination or imaging, but some children present with complaints of abdominal pain, increasing abdominal girth, nausea and/or vomiting, dysuria. An acute presentation due to ovarian torsion is possible and the caregivers of such patients should be aware of this possibility (Please refer to *Surgical Emergencies in Paediatric Oncology Guidelines*). Some clinical features are more often associated with malignancy: bilateral masses, fixed masses with irregular borders, ascites, precocious puberty.

Different histotypes can be present:

- **Germ cell tumours (GCTs)** – the majority of ovarian tumours in children and adolescents are GCT, both benign (mature teratoma or gonadoblastoma) and malignant (immature teratoma or malignant GCT, dysgerminoma)
- **Epithelial tumours** – serous or mucinous cystadenoma is rare in children, but they must be considered when cystic lesions are discovered, in particular when bigger than 5 cm, or in prepubertal girls or not showing any influence by the hormonal status
- **Sex-cord-stromal tumours** – rare in children, they may present precocious puberty, both isosexual and heterosexual

Transabdominal ultrasonography is the first-line imaging, providing information about the size and origin of the mass, the consistency, the pattern of blood supply and other associated findings including the side affected. In case of large tumours or when malignancy is suspected, further information can be obtained with CT or magnetic resonance imaging (MRI). A complete panel of tumour markers, including αFP, βHCG, lactate dehydrogenase, Inhibin A and B, cancer antigen 125, oestradiol, testosterone can help in hormone secreting tumours, and if elevated can be useful in monitoring the response to treatment and/or detect early relapses. Surgery is the cornerstone of treatment, and the goals of surgical management include definitive diagnosis, complete removal of the tumour and staging for malignancy (through abdominal and pelvic exploration, peritoneal washing, contralateral ovary inspection, biopsy of the omentum and of other suspicious lesions and of periaortic and pelvic lymph node). Conservative surgery can be considered unless malignancy is highly suspected or confirmed on frozen section at the time of procedure: even huge cystic lesions can be successfully excised preserving normal ovarian cortex. The surgical approach can be open or laparoscopic, avoiding rupture and spillage of the tumour [20, 21].

Sacrococcygeal GCTs arising from the Hensen Node cells can be totally (Altman stage IV) or mainly (Altman stage III) intrapelvic. These tumours are mainly diagnosed antenatally or in infants, and in these cases they are mainly mature teratomas. Serum αFP and βHCG will confirm the benign nature of the tumour and in these cases an upfront complete resection with complete removal of the coccyx will be the unique treatment required. Oncological follow-up is still necessary for these patients since a small percentage of those might recur. In older children, malignancy is more represented, and often an elevation of the serum markers is sufficient to diagnose a Malignant GCT, with mainly a Yolk Sac Tumour or a Choriocarcinoma histotype. In this case, a complete workup is necessary to detect possible metastases, but chemotherapy can be started without further histological sampling [22] (Please refer to *Germ Cell Tumour – Sacrococcygeal Teratoma Guidelines*).

Neuroblastoma can arise in the pelvis, mainly from the Zuckerkandl organ or from other lower ganglion. They are often unresectable at diagnosis due to the encasement of vessels (such as aorta, cava vein, iliac vessels, or lower mesenteric artery), and therefore the diagnosis should be confirmed through a biopsy. As for neuroblastoma in other localisation, tru-cut biopsy is encouraged but laparoscopic or open biopsy can be performed, considered that the main objective of a biopsy is to obtain enough material to perform all the immunohistochemical and biological examination to characterise the tumour. Pelvic neuroblastoma often presents good prognostic factors, and after neoadjuvant chemotherapy and delayed complete or incomplete excision, has a good outcome [23, 24] (Please refer to *Neuroblastoma Guidelines*).

Soft Tissue Sarcoma, potentially arising in every site, can present as pelvic masses. Initial presentation can be determined by signs and symptoms relied to complications, such as urinary output obstruction or intestinal occlusion. In these conditions, the correct identification is often difficult at diagnosis, and a biopsy needs to be performed in order to obtain tissue for histological examination. A cystoscopy is often suggested: in case of a bladder/prostate RMS, it can be useful both for a diagnostic purpose and for a therapeutic aim, allowing to insert urethral stent and or bladder catheter in order to prevent further renal damage. A complete diagnostic workup includes a thoraco-abdominal CT scan, bone marrow biopsy, bone scintigraphy and positron emission tomography (PET) scan. After neoadjuvant chemotherapy, a re-evaluation of the tumour extension should be obtained in order to plan the better surgical plan [25, 26] (Please refer to *Rhabdomyosarcoma and Non-Rhabdomyosarcoma Soft-tissue Sarcoma Guidelines*).

## Scrotal Mass

Scrotal masses are caused by different conditions, ranging from benign diseases (inguinal hernia, hydrocele, varicocele) to those requiring emergent surgical intervention (testicular torsion) to testicular or paratesticular tumours that can present with pain, more or less acute, in 15% of cases. An accurate systematic evaluation including clinical history and physical findings (location of the mass) should allow a quite affordable indication, and Doppler ultrasonography can be helpful.

Testicular tumours could arise both from GCTs and from stromal cells (Please refer to *Germ Cell Tumours – Testicular Tumours Guidelines*). In suspected malignant GCT, the treatment of choice is radical orchiectomy that guarantees both an accurate diagnosis and the local control of the disease, that in presence of a localised stage of the tumour, can be adequate and sufficient [27, 28]. However, in specific cases, a more conservative approach should be considered. Testis-preserving strategies should be considered in unilateral or bilateral synchronous or metachronous GCT. Moreover, it should be performed for small testicular masses, which are mostly benign or borderline tumours (Sertoli cells tumours, Leydig cells tumours, adenomatoid tumours, epidermoid cysts). In these cases, an accurate follow-up is mandatory to detect possible recurrences or metachronous tumours.

Testis can also harbour recurrences of Acute Lymphoblastic Leukaemia (ALL). The suspect arises in children treated for ALL who present enlarged testis. The confirmation of diagnosis can be obtained through a fine needle aspiration (FNA) biopsy, both of the affected testis and the contralateral, and the treatment is based on the orchiectomy or on radiation therapy (RT), both causing castration [29].

Paratesticular tumours are mainly represented by paratesticular RMS. RMS can arise from the tissues surrounding the testis, and therefore it sometimes can be palpated as a solid mass separated from the testis. The initial correct approach should consist in orchiectomy with high ligation of the cord through an inguinal approach [30]. This is one of the few sites where initial excision is recommended for a RMS. In case of an initial surgical approach performed through the scrotum, it is suggested to complete the procedure with a hemiscrotectomy; otherwise an overstaging and consequently an overtreatment of the patient is recommended in order to avoid possible local relapse. Due to the high risk of nodal relapse registered in patients older than 10 years, the retroperitoneal lymph-node evaluation is strongly recommended. Many of the actual protocols agree in suggesting the use of laparoscopic retroperitoneal lymph node sampling at least for patients aged >10 years (Please refer to *Rhabdomyosarcoma Guidelines*).

## Thoracic Mass

(Please refer to *Thoracic Tumours, Neuroblastoma and Surgery for Lymphoma Guidelines*)

### Mediastinal tumours

Mediastinal tumours in children include a broad spectrum of diagnoses. Although up to 65%–80% of mediastinal lesions are malignant, the diagnostic possibilities of a mediastinal mass are multiple, as well as its presenting symptoms. In addition to the direct mass effect, patients may show up symptoms associated with systemic effects of the disease process.

Several techniques are available for the biopsy of a mediastinal mass, such as image guided (CT/ultrasound), anterior thoracotomy or Chamberlain procedure, video-assisted thoracoscopic surgery [31]. Nevertheless, image-guided transthoracic biopsy is the most frequently used. Each one of these techniques has its own advantages and disadvantages, but decision usually depends on the tumour size, location, age and experience of the surgical team. Collaboration and teamwork with the interventional radiology department is of the utmost importance.

Pathological confirmation of the malignancy will not always be necessary as some mediastinal masses show specific radiological findings. Taking into account mediastinal anatomy and its compartment (Table 1) is important when it comes to guiding diagnostic management.

### Anterior mediastinal tumours

A multidisciplinary and systematised approach is recommended for masses in the anterior mediastinum [33]. Our usual role as paediatric surgeons is to coordinate and communicate alongside paediatric oncologists, paediatric anaesthesiologists, interventional radiologists and paediatric critical intensivists. The least invasive approach that gives us enough sample to reach the diagnosis is always recommended. Principally if the patient presents respiratory symptoms, it is advisable to perform the surgical procedures under local anaesthesia [34].

Patients must be carefully evaluated regarding presence of any pleural effusion or palpable lymphadenopathy accessible to physical examination. If the diagnosis can be reached by obtaining a sample of any of the above, it is preferable to perform the biopsy of the mediastinal mass as a primary approach.

It is mandatory to evaluate the risk of any anaesthetic complications caused by mass effect together with the anaesthesiology team prior to deciding the optimal procedure, which must be individualised to each clinical scenario. Communication with the pathologist must be coordinated in the case of addressing an extrathoracic focus or using minimally invasive procedures in order to try to achieve an accurate diagnosis.

### Middle mediastinal tumours

Lymphomas are the most frequent tumour in this area, sometimes affecting both the anterior and middle mediastinum. In this compartment, for anatomical reasons, minimally invasive or interventional radiology approaches should be prioritised to reach the diagnosis.

### Posterior mediastinal tumours

Tumours of neural crest origin are the most common posterior mediastinal lesions. Its histology can range from benign ganglioneuroma to malignant neuroblastoma. As previously discussed in the adrenal tumours section, in the case of neuroblastoma there are patients categorised as L1 and who may receive an upfront tumour resection which is both diagnostic and therapeutic. In all other cases, the usual neuroblastoma staging protocol should be followed. If the mass is suspicious of benignity after a complete work-up (e.g. ganglioneuroma), primary resection without biopsy is recommended.

#### Chest wall tumours

Paediatric chest wall tumours can have a heterogeneous origin and may appear at any age from infancy to late adolescence. They can be benign or malignant (Table 2) and secondary or primary. After taking clinical history and performing a complete physical examination, imaging studies are mandatory. Full radiological work-up includes a chest X-ray, computerised axial tomography (CT scan), and/or MRI. CT scan and MRI have both advantages and disadvantages [35], so any of them can be performed if available. Usually, thoracic CT scan provides information on several characteristics such as size, location, bony involvement and infiltration into contiguous structures. CT scans are also the best method to screen the lungs for metastases. MRI can show details of the soft tissue area of the lesion, in addition to the presence of fluid within the chest wall and even spinal or epidural extension.

Once initial studies have been performed, retrieval of tissue for histopathological evaluation and diagnosis is mandatory. The options for biopsy technique in this case will depend on the size of the lesion and the availability. If the mass is small (less than 3 cm) or highly suggestive of being benign, an excisional biopsy may be considered. Careful planning of the incisions positioning should be made, always considering oncological principles, so that a future re-excision can be performed. As it is the usual practice, a border of healthy tissue must be excised around the lesion.

If the mass is large (greater than 4–5 cm), fixed to surrounding structures, involving multiple structures in the thorax, or if it is considered malignant by imaging, then either an incisional biopsy or core-needle technique biopsy is mandatory. The surgical technique of choice should be the one which the surgical team is more experienced with, always ensuring it obtains enough tissue for the pathologist to make the diagnosis. If an incisional biopsy is performed, the orientation and size of the incision must preserve the oncological principles and never compromise the definitive surgery. Overall, strictly for diagnostic purposes, a needle biopsy is favoured over an incisional or excisional biopsy in most of the cases.

We bear in mind that in some of the malignant processes included within chest wall tumours (such as EFST) the mainstay treatment is multimodal (combining surgery, chemotherapy and RT). Surgical resection can precede other treatment modalities only if it achieves negative margins and if disfigurement and loss of function are avoided. This concept must be emphasised, primary massive resections are absolutely contraindicated, leading to important long-term effects for the patient (including rib wall defects, scoliosis [36, 37] or pulmonary impairment).

It is not the focus of this chapter to discuss reconstructive surgical options, but this aspect should be perfectly planned in a detailed presurgical manner. Reconstructive techniques must take into account the effect on chest wall stability and function [38], as well as thoracic protection. Communication with the pathology team must be fluid in order to choosing the best biopsy technique which allows a correct diagnosis and has enough tissue sample for all the necessary studies (histopathological, cytogenetic and molecular). Once a diagnosis is confirmed, then specific therapeutic algorithms of each disease will be started.

**Pulmonary tumours** (Please refer to *Thoracic Tumours and Rare Tumours Guidelines*)

### Primary lung tumours

Primary pulmonary tumours of the lung are unusual in infants and children; them being secondary to metastatic disease [32]. Despite the extremely low incidence of these lesions in children, the majority are malignant. The approximate incidence of primary malignant tumours in the paediatric population is estimated to be 0.049 per 100,000 infants.

The diagnostic process can be challenging given the non-specificity of symptoms and rarity of the disease. This clinical entity presents with nonspecific symptoms that may mimic common entities, such as cough, pneumonia, haemoptysis or shortness of breath.

Initial workup should include laboratory studies and a chest radiography. Persistent symptoms or radiological image findings would require a CT scan of the chest and evaluation by a paediatric pneumologist [39].

Primary lung tumours are histopathologically diverse. Despite its low frequency, inflammatory myofibroblastic tumour (IMT) is the most common benign primary pulmonary neoplasm in paediatric population. It has very particular characteristics due to its natural tendency towards local invasion.

Bronchial adenoma is the most frequently primary malignant pulmonary tumour. They constitute a heterogeneous group of primarily endobronchial lesions, being the carcinoid variant is the most common one [40]. Other histological variants can be found in the Table 3.

The identification of a lung lesion requires to complete the study with a bronchoscopy for central lesions and thoracoscopic or image-guided biopsy for peripheral lesions. Bronchoscopy is initially considered as an inspection study, so the decision of performing a bronchoscopic endobronchial biopsy must be individualised and performed only in reference centres in paediatric airway pathology due to the high risk of bleeding with a fatal outcome.

Diagnosis is established by CT of the chest, bronchoscopy and biopsy.

Endoscopic resection is not recommended given the high risk of incomplete resection of the bronchial wall. Surgical resection of the tumour and associated regional lymphadenectomy is the preferred treatment, having an excellent rate of overall survival [41]. The surgical approach of choice should be a conservative pulmonary resection for peripheral lesions, or a bronchial sleeve resection with reconstruction for the central ones.

**Pulmonary metastases** (Please refer to *Pulmonary Metastasis Guidelines*)

The most frequent lung metastatic diseases in the paediatric age include Wilms tumours, osteosarcoma, Ewing sarcoma and rhabdomyosarcoma as the origin of the lesions. Pulmonary metastasectomy is considered most frequently for osteosarcoma. CT remains the gold standard for the identification of pulmonary nodules in paediatric solid tumours.

The diagnostic or therapeutic value of pulmonary metastasectomy will depend on the management protocol of each histological type and the evolutionary stage of the disease. When its role is only diagnostic, it can guide further systemic treatment.

The main technical difficulty in performing lung metastasectomy, especially when the chosen approach is minimally invasive, thoracoscopic as an example, is the location of the lesions. Multiple surgical strategies [42] have been described in order to solve this problem, so each centre and surgical team must choose their ideal solution according to their experience and available equipment. Some of the reported techniques include pre-operative marking with wires, coils or dye, and localisation with intraoperative image guided use of indocyanine green. Even if all of these strategies are useful, each one has its own drawbacks.

Among the contraindications to perform a pulmonary metastasectomy, the impossibility of achieving a complete resection while maintaining an acceptable lung function and the presence of uncontrolled disease in the primary location are two remarkable ones.

**Neck Mass** (Please refer to *Management of Enlargement of Lymph Node Guidelines*)

Neck masses represent a common, regularly encountered clinical entity in children. It is a challenging medical condition for the child, raises anxiety for family and can often be perplexing to the paediatrician [43].

### Where surgeons come in

Often times these children are referred to the surgeon for an opinion. Surgeons with a clear understanding of embryology and anatomy of the different structures in the cervical region and its facial planes and of the natural history of a specific lesion are at an advantage when suggesting a plan of management. The differential diagnosis is broad and includes an exhaustive list of congenital, inflammatory and neoplastic lesions. In the paediatric population, 80%–90% of all head and neck masses represent benign conditions [44]. The majority of such children are found to have enlarged lymph nodes that resolve either spontaneously or with antibiotics.

### When to consider a malignant process

In a small number, however, the presenting mass, lymph node or otherwise, persists or enlarges which should be a cause for concern [45]. Distinguishing benign from malignant masses is a critical first step to institute a multidisciplinary approach to the management of a suspected malignant lesion. Neoplasms of the head and neck account for approximately 5% of all childhood malignancies [46].

Age at onset, duration and mode of symptoms in addition to the anatomical site and size of the mass are important elements that aid in identifying the probable malignant pathologies to be included in the differential diagnosis. It is important to remember that although malignant tumours in the neck region are rare during the first 3–6 months of life, some malignant tumours present this early.

Beyond infancy, enlarged neck lymph node(s) are a common presentation which is commonly identified as cervical lymphadenopathy following a viral or bacterial illness. Persistent adenopathy in the anterior cervical triangle or a single dominant node that persists for more than 6 weeks, multiple nodes that are painless, firm and fixed, enlarged lymph node(s) within the posterior triangle or supraclavicular space should heighten concern to exclude malignancy.

### Orderly approach

A thorough physical examination of the head, neck and chest as well as the rest of the body with an appropriately directed workup will facilitate the diagnosis. Detailed ultrasonography of the entire neck region is the preferred initial test. It is painless, does not require anaesthesia and can provide useful information that will dictate the next most appropriate step in management. A suspected malignant mass in the neck can be the primary tumour itself, an extension or metastatic of a primary below the base of the skull or of a tumour in the upper mediastinum. It can be the site of metastasis of a primary in the abdomen.

When a malignant process is suspected on ultrasonography and the thyroid gland is seen to be normal, either CT or MRI with intra-venous contrast is advised [47] and should not be delayed. At this step, it is a good practice to consider the regions to be visualised on imaging *a priori* which is dictated by the disease entity under suspicion. This is where age of the child and precise anatomical site of the mass in the neck become decisive.

### Entities for consideration

In newborns and infants, neuroblastoma and rhabdoid tumour should be first on the list [48]. Congenital torticollis is a differential diagnosis to be considered in newborns. In older children, RMS is more common. Cervical skeletal anomalies (i.e. cervical rib, transverse mega-apophysis) should be added to the list when the mass is hard [47, 48]. Skeletal abnormalities can be easily confirmed by a simple X-ray. At older ages, lymphoma in many countries comes at the top of this list while in others leukaemic infiltrate, acute lymphocytic leukaemia and acute myeloid leukaemia come on top of the list [49].

### Establishing a diagnosis

Once CT or MRI is suggestive of malignancy, obtaining tissue for diagnosis becomes critical and should not be delayed. The mode of obtaining tissue for diagnosis is determined by the type of anticipated malignant process. Where lymphoma is suspected, FNA or true cut biopsy is useful and frequently diagnostic. FDG-PET is helpful not only to decide what would be the best site to target but would clarify the extent of the disease. If the FNA result is inconclusive for lymphoma, an open biopsy is recommended. It cannot be emphasised enough that obtaining sufficient tissue is as important as performing a safe excisional surgical intervention.

***Surgery upfront for diagnosis versus local control*** (Please refer to *Neuroblastoma,* and *Rhabdomyosarcoma and Non-Rhabdomyosarcoma Soft-tissue Sarcoma Guidelines)*

When the primary is thought to be neuroblastoma, RMS, primitive neuroectodermal tumor (PNET) or rhabdoid tumour, the temptation for upfront surgical excision out of urgency and necessity should be strongly resisted. For such tumours, staging and risk stratification take precedence over immediate local control. Many tumours require resection with negative margins which can be impossible to safely achieve upfront. On the other hand, neuroblastoma in particular, unlike other tumours, can be safely observed. Therefore, priority goes to obtain sample of the lesion’s tissue for diagnosis using a true cut or large bore needle biopsy.

In the neck it is more common to see neuroblastoma metastasis than a primary. Primary cervical neuroblastoma accounts for less than 5% of all neuroblastoma cases [50] and generally is an L1 disease with a favourable outcome. MYCN (v-myc myelocytomatosis viral related oncogene, neuroblastoma derived) amplification in this location is exceptionally rare. In instances where surgical morbidity is unacceptably high, or R0 is not achievable, as evidenced by imaging risk factors, neo-adjuvant therapy can and should be considered first. IDRF can accurately predict the completeness, safety and probable complications of surgical resection [50]. Since an L1 disease has excellent prognosis even in the presence of tumour, residue radicalism is unwarranted and the residue can be safely observed [51].

RMS is the second most common malignant tumour after lymphoma. 30% of head and neck RMS are termed nonorbital, nonparameningeal which arise from the oral cavity, larynx, parotid region, cheek, scalp and soft tissue of the neck. The diagnosis requires a tru-cut or large bore needle biopsy. Local control requires wide safe margin which is difficult to achieve in the neck at presentation. Since these tumours carry a favourable prognosis and are made more amenable to surgical excision following neoadjuvant therapy, delayed local control is the rule. The multimodality approach for these tumours is well established and is directed by stage [52, 53].

Head and neck primitive neuroectodermal tumours (PNETs) are rare, accounting for 5%–10% of all PNETs. Head and neck synovial sarcomas are uncommon and carry a poor prognosis. In the head and neck region, the primary is often located laterally in the Parapharyngeal space. The tumour can spread loco-regionally and systemically easily, so it makes management challenging [54]. Surgery as the sole mode of local control is not enough. The extensiveness of the local tumour precludes safe total resection with negative margins.

Malignant rhabdoid tumours (MRT) in the cervical region are very rare compared to other types of tumours in this region. Data from the UK showed that head and neck MRT accounted for about 15% of all extra‐cranial MRT [55, 56]. However, 45% of MRT are non-cranial and extra renal. The estimated 5‐year survival for the entire group was 33% ± 3.4% (SE). Univariate and multivariate analyses showed that age at diagnosis (2–18 years), localised stage of tumours and use of radiotherapy were significantly associated with improved survival [57]. When surgery is not feasible as a mode of local control, brachytherapy should be considered [58].

## Extremity Mass

Masses localised in extremities should always been regarded with suspicion, because they are often the first clinical sign of a sarcoma, both from the soft tissues and from the bone. (Please refer to *Rhabdomyosarcoma and Non-rhabdomyosarcoma Soft-Tissue Sarcoma, and Osteosarcoma and Ewing Sarcoma Guidelines*) Frequently the masses are detected after a trauma, and this leads sometimes in a delay in diagnosis due to the initial interpretation of a consequence of the trauma itself.

First imaging should include X-ray and an ultrasound scan of the extremity affected. Almost regularly will be followed by an MRI that can more precisely describe the extension and characteristic of the tumour. If bones are involved, a CT scan could add important information on the tumour.

Based on the imaging results, some diagnosis can be confirmed or excluded according to their specific features (nerve-tumours, schwannomas, myositis ossificans, lipomas, venous malformations, synovial cysts, etc.). In all other cases, a biopsy should be performed.

Most biopsies are percutaneous and allow a diagnosis in 95% of cases in specialised cancer centres: strict aseptic conditions must be used, and assuming that the tumour is malignant, the biopsy tract should be marked in order to include its resection when the tumour resection is performed. The biopsy is generally performed with a 16 or 18 G core needle, preferably under imaging control (ultrasonography or CT scan) [59].

The indications for surgical biopsy have decreased over time and nowadays it is only indicated when it is impossible to obtain usable anatomical pathology specimen. In both cases, the biopsy tract or incision should be discussed with the surgeon who will perform the subsequent excision, considered that it will need to be included in the incision for the subsequent excision. In the presence of RMS and some others soft-tissue sarcoma, the evaluation of the possible nodal spread is mandatory: sentinel node biopsy has demonstrated to be a useful tool to obtain significant material avoiding the complications of a more aggressive nodal surgical approach [60].

## Tips and Pitfalls in the Diagnosis of Paediatric Cancer

- Diagnosis of malignancy may be obtained from a primary tumour or its metastatic sites; therefore, it is critical to recognise the pattern of disease dissemination for each histological subtype.
- Patients with L1 neuroblastoma may receive upfront tumour resection which is both, diagnostic and therapeutic. Resection, when performed as the initial intervention, may obviate the need for chemotherapy as many of these patients will have low-risk disease and have an excellent prognosis (Please refer to *Neuroblastoma Guidelines*).
- In the setting of bilateral kidney tumours both, SIOP and COG protocols, do not require tissue diagnosis to initiate neoadjuvant chemotherapy (Please refer to *Wilms Tumour Guidelines*).
- Surgical candidates for upfront resection in HBL include PRETEXT I (a tumour that involves only one section) and II (a tumour that involves two sections) and >1 cm radiographic margin on the middle hepatic vein, the retro-hepatic IVC and or portal bifurcation (Please refer to *Hepatoblastoma Guidelines*).
- Surgery is the cornerstone of treatment of ovarian masses, and the goals of surgical management include definitive diagnosis, complete removal of the tumour and staging for malignancy (Please refer to *Germ Cell Tumours Guidelines*).
- Due to the high risk of nodal relapse registered in patients with paratesticular RMS, the retroperitoneal lymph-node evaluation is strongly recommended for any patient with positive imaging findings and patients aged >10 years with negative findings (Please refer to *Rhabdomyosarcoma Guidelines*).
- For patients with anterior mediastinal mass, it is mandatory to evaluate the risk of any anaesthetic complications caused by mass effect together with the anaesthesiology team prior to deciding the optimal procedure, which must be individualised to each clinical scenario (Please refer to *Thoracic Tumours Guidelines*).
- Massive resection of suspected Ewing sarcoma of the chest wall may lead to positive margins and long-term complications and it should be discouraged (Please refer to *Osteosarcoma and Ewing Sarcoma, and Non-rhabdomyosarcoma Soft-Tissue Sarcoma Guidelines*).
- The value of pulmonary metastasectomies will depend on each histological subtype and the evolutionary stage of the disease (Please refer to *Pulmonary Metastasis Guidelines*).
- Persistent adenopathy for more than 6 weeks should heighten concern to exclude malignancy (Please refer to *Management of Enlargement of Lymph Node Guideline*).
- The biopsy tract of a malignant tumour should be marked in order to be included when the definitive tumour resection is performed.

## Figures and Tables

**Table 1.** Mediastinal tumours classified by compartment.

Anterior mediastinum	Middle mediastinum	Posterior mediastinum
Lymphomas Hodgkin’s lymphoma Non-Hodgkin’s lymphoma GCTs Teratomas Malignant GCTs Thymus Thymoma Thymolipoma Vascular anomalies	Lymphoma Lymphatic malformations Haemangioma Pericardial cysts Bronchogenic cysts Gorham’s disease	Neuroblastoma Ganglioneuroma Ganglioneuroblastoma Schwannoma Neurofibroma Paraganglioma Primitive neuroectodermal tumour Oesophageal duplications

**Table 2.** Paediatric chest wall tumours.

Benign	Malignant
Aneurysmal bone cyst Chondroma Desmoid Fibroma Fibrous dysplasia Lipoblastoma Lipoma Mesenchymal hamartoma Osteochondroma Osteoma Vascular malformations	Chondrosarcoma Ewing’s sarcoma Fibrosarcoma Langerhans cell histiocytosis Leiomyosarcoma Leukaemia Liposarcoma Lymphoma Neuroblastoma RMS Osteosarcoma

**Table 3.** Most frequent paediatric primary lung tumours.

Benign	Malignant
IMT Hamartoma	Bronchial adenoma Bronchogenic carcinoma Pulmonary blastoma RMS
