# Supplementary material for: International Society of Paediatric Surgical Oncology (IPSO) Surgical Practice Guidelines
Source: Ecancermedicalscience. 2022 Feb 17;16:1356. doi: 10.3332/ecancer.2022.1356 (PMC9023308; doi:10.3332/ecancer.2022.1356)
Supplement: Related texts

# Discussion: Management of lymph node enlargement in children

**Published:** 2022

**Authors:** Ahmed Elgendy, Hafeez Abdelhafeez, Simone Abib

## Preoperative: Evaluation, Images, Special needs and Biopsy Need

Lymphadenopathy is a condition in which lymph nodes are abnormal in size and consistency. The neck is the most common peripheral site of enlarged lymph nodes. A lymph node is considered enlarged if it is more than 1 cm in diameter if cervical or axillary, and more than 1.5 cm in diameter if inguinal. Peripheral lymphadenopathy is common in children and adolescents, and approximately 38%–45% of healthy children have enlarged lymph nodes [1]. Conditions such as infections, reactive hyperplasia, autoimmune diseases, chronic inflammatory diseases and malignancies are associated with lymph node enlargement.

The most common cause of cervical lymphadenopathy is viral upper respiratory tract infection. Differential viral aetiologies also include Epstein–Barr virus (EBV) and cytomegalovirus (CMV). Group A beta-haemolytic streptococci and *Staphylococcus aureus* are the most common causes of bacterial cervical lymphadenitis in children. In addition, anaerobic bacteria from dental caries and periodontal disease are bacterial causes of lymphadenopathy. Therefore, evaluating the condition of teeth should always be part of the physical examination of children with enlarged lymph nodes on the neck. Cat scratch disease caused by *Bartonella henselae* can also cause lymphadenopathy, and thus the patient’s history should be studied for contact with cats. Atypical mycobacteria and *Mycobacterium tuberculosis* are important causes of subacute or chronic cervical lymphadenopathy. Immunocompromised patients should be tested for fungal infections. Parasitic infections, such as toxoplasmosis, can also cause lymphadenopathy. Paracoccidiomicosis and other infections that are typical in some countries should be investigated, depending on the patient’s country of origin. Furthermore, immunological diseases can cause lymph node enlargement (rheumatoid arthritis, mixed connective tissue disease, Sjögren syndrome, graft-versus-host disease). Other rarer conditions, such as lipid storage diseases, endocrine diseases and Kawasaki and Castleman diseases, can also be a differential diagnosis.

Benign cervical masses such as dermoid and thyroglossal cysts in the midline, salivary gland enlargement, branchial cyst and congenital torticollis are other differential diagnoses to be considered.

Hodgkin lymphoma, non-Hodgkin lymphoma, neuroblastoma, leukaemia, rhabdomyosarcoma and metastatic diseases are the most frequent neoplasms associated with cervical lymph node enlargement.

Therefore, paediatricians and paediatric surgeons need to rule out malignancy. A detailed history and careful physical examination are imperative steps in the initial evaluation of children presenting with peripheral lymphadenopathy.

Important information that needs to be included in the history are age, location and duration of lymph node enlargement and its evolution (e.g. stable in size, growing, changing characteristics); associated symptoms (e.g. cough, pain, tenderness, fever, night sweats, weight loss); and association of upper respiratory tract infection, contact with cats, family history of tuberculosis (TB) and human immunodeficiency virus (HIV) status.

Physical examination should include the overall state of health (e.g. healthy, malnutrition, poor growth), location of lymph nodes (e.g. posterior cervical, supraclavicular, axilla, groin), characteristics of lymph nodes (e.g. tenderness, erythema, warmth, mobility, fluctuance, consistency, coalescence), presence of lymphadenopathy in other lymphatic chains outside the neck, hepatomegaly and splenomegaly. Certain nodes can be treated without being investigated. Obvious bacterial infections or reactions to infections within the drainage area need to be treated and followed up until resolution.

Furthermore, certain laboratory investigations should be conducted in case of general or specific clinical manifestations to exclude the presence of infectious aetiology. Complete blood picture in addition to serological tests such as EBV, CMV, HIV, tuberculin skin tests, bartonella and toxoplasmosis should be performed for suspected patients. Imaging studies are recommended in children with lymphadenopathy, who present with progressively enlarging, firm, fixed nodes or with associated systemic features. Mediastinal involvement should be screened initially with a chest radiograph.

Ultrasound is the preferred initial modality because of its real-time assessment without the need for general anaesthesia. Moreover, it does not expose patients to ionising radiation. Ultrasound gives data on the size, shape and architecture for distinguishing benign from malignant nodes. In selected cases, computed tomography or magnetic resonance imaging can be used to gain further information. Ultrasound elastography can also be used as an adjunct tool for sonographic findings, which can improve the accuracy of predicting malignant lymph nodes [2]. Thick cortex with loss of fatty hilum, central necrosis and a heterogeneous echo pattern with hyperechoic foci are suspicious characteristics in imaging modalities.

Fine needle aspiration cytology (FNAC) may be an option for pathological diagnosis in the paediatric population, as it is a minimally invasive procedure. Older children may be cooperative, and the use of topical anaesthetic creams aids the procedure. Suitable conditions to perform fine needle aspiration biopsy at established facilities can facilitate the process and be a more efficient way of diagnosing the cause of lymphadenopathy and avoiding the need for theatre time and a general anaesthetic. However, when performing FNAC, several limitations can be encountered, such as insufficient material for examination, false-negative results and the need for an experienced paediatric pathologist and sedation or general anaesthesia in some patients. These factors suggest that relying on FNAC alone is inconclusive [5]. If FNAC gives a clear and precise diagnosis, the patients are treated accordingly, but if patients have equivocal, ‘reactive’ or indeterminate results, they need to undergo an open biopsy. Eventually, open biopsy remains the standard and precise procedure to obtain a tissue diagnosis of lymphadenopathy in children.

## Surgical Goals

If the primary diagnostic work-up cannot identify the valid cause of peripheral lymphadenopathy, a definitive histopathological diagnosis is required. Children who present with persistent enlarged lymph nodes for more than 4 weeks despite being administered empirical antibiotics should be prepared to undergo a surgical biopsy [3]. The possibility of malignancies based on clinical aspects usually drives surgeons to make this decision, and predictors include long duration (no decrease in size in 4–6 weeks; no resolution in 8–12 weeks), multiple levels of lymphadenopathy, supraclavicular location, hard or fixed nodes, increase in size over 2 weeks, suspicious radiological signs and increased nodal size (>2 cm) [4, 6, 7]. Concomitant clinical manifestations such as weight loss, organomegaly, persistent or unexplained fever and night sweats, in addition to older age of patients (>10 years) should be added to the aforementioned criteria for nodal biopsy. Sometimes, the surgeon’s role is to only drain the cervical abscess.

Please refer to *Surgery for Lymphoma and Thoracic Masses guidelines* for thoracic and abdominal lymph node enlargement that might need biopsy.

## Pitfalls

- Preoperative evaluation of mediastinum enlargement should be performed before a child is given general anaesthesia, due to the risk of ventilation problems and death during induction, when there is a significative mediastinum mass. In this situation, the surgeon should look for peripheral lymph nodes that can be biopsied under local anaesthesia or pleural effusion, so that a thoracocentesis diagnosis can be made. If there are no peripheral lymph nodes to biopsy, the anaesthesiologist should be aware of the risks and do whatever possible to prevent complications.
- Sometimes, more than one biopsy may be needed to diagnose Hodgkin lymphoma. To avoid that situation, the surgeon should ensure that a representative lymph node is biopsied. If the diagnosis is ‘reactive’ or inconclusive on the open biopsy, the patient should be followed up until the case is resolved or it is further investigated.
- It is important to note that patients from countries where TB and HIV are prevalent may have concurrent different diagnoses.

## Surgery

Open excisional biopsy is the procedure of choice to obtain adequate tissue samples for histopathological assessment. Both in localised and generalised lymphadenopathies, the largest and most accessible node, decided by clinical examination and preoperative imaging, should be removed completely with an intact capsule for a precise pathological result.

## Complications

Surgical biopsy is often a safe procedure; however, some complications such as bleeding, haematoma formation, nerve injuries (spinal accessory or marginal mandibular), infection and risks associated with general anaesthesia may occur.

## Conclusions

- Enlargement of peripheral lymph nodes is a very common finding in children and adolescents.
- Predictive factors should be correlated by careful analysis of history, examinations and radiological findings to make a decision about biopsy.
- Open surgical biopsy is the cornerstone of diagnosing paediatric lymphadenopathy with an equivocal aetiology, as the accuracy of results using FNAC still remains unclear.

## References

[1] Niedzielska G, Kotowski M, Niedzielski A (2007). Cervical lymphadenopathy in children: incidence and diagnostic management. Int J Pediatr Otorhinolaryngol.

[2] Zakaria OM, Mousa A, AlSadhan R (2018). Reliability of sonoelastography in predicting pediatric cervical lymph node malignancy. Pediatr Surg Int.

[3] Celenk F, Baysal E, Aytac I (2013). Incidence and predictors of malignancy in children with persistent cervical lymphadenopathy. Int J Pediatr Otorhinolaryngol.

[4] Nolder AR (2013). Paediatric cervical lymphadenopathy: when to biopsy?. Curr Opin Otolaryngol Head Neck Surg.

[5] Locke R, Comfort R, Kubba H (2014). When does an enlarged cervical lymph node in a child need excision? A systematic review. Int J Pediatr Otorhinolaryngol.

[6] Rajasekaran K, Krakovitz P (2013). Enlarged neck lymph nodes in children. Pediatr Clin N Am.

[7] Kliegman R, Geme JS, Kliegman RM, Stanton BF, St. Geme JW (2019). Nelson Textbook of Pediatrics.

# Discussion: Venous access for the paediatric cancer patient

**Published:** 2022

**Authors:** Israel Fernandez-Pineda, Sharon Cox, Chan Hon Chui, Jörg Fuchs, Simone Abib

## Devices Available

There are three categories of central venous access devices (CVAD) [1]

- non-tunnelled lines like peripherally inserted central catheters (PICCs) and ‘push-in’ central venous catheters (CVCs)
- tunnelled CVCs such as Hickman® or Broviac® lines with single or multiple lumens
- totally implantable venous access devices (TIVADs) or ports

Devices should be selected according to the indication and required duration. Simple CVC or PICC lines are suitable for days to weeks. Medium term access for weeks to months may require PICCs or tunnelled catheters. If access is required for longer than 3 months CVADs or TIVADs are indicated, the choice being determined by factors including patient comfort and activity, nursing experience, frequency of use and cost.

Low resource settings may have challenges with stocking multiple catheter types, lengths and sizes and should determine which device sizes are most frequently used in their unit [1].

The calibre of the selected catheter should be smaller than that of the vein to allow for flow around the catheter and prevent venous thrombosis. It is suggested that the outer diameter of the line should be equal to or smaller than one third of the venous diameter. The ‘French’ system, size of a catheter refers to its circumference, with 1 French being equal to one third of a millimetre. Measuring the vein diameter on ultrasound can thus assist with determination of the size of the catheter.

## Surgical Goals

CVCs are extremely important in the management of children with malignancies [2]. The surgical goals of CVC placement in the paediatric cancer patient include providing durable access for the administration of chemotherapy, antibiotics, blood products, support of patients undergoing haematopoietic stem cell transplantation or those receiving parenteral nutrition and dialysis, while minimising the intraoperative and postoperative complications [3]. These goals may be achieved by a PICC or more commonly, by accessing a central vein with the placement of an external device (Hickman®, Broviac®) or a subcutaneous infusion port. The choice of the CVC device is dependent on institutional protocols. External devices are visible, easy to access with choice of single or multiple lumens, no needle stick be required but they are associated with a higher incidence of infection, although device removal without general anaesthesia in an outpatient clinic is an advantage. Subcutaneous infusion ports are not visible, require needle stick to access and they are associated with a lower incidence of infection, although general anaesthesia is usually needed at port removal [4].

## Preoperative Evaluation, Images and Special Needs

Paediatric patients, in particular, are less likely to tolerate an awake procedure with local anaesthesia only. They are frequently taken to the operating room or a fluoroscopy suite for CVC placement under sedation or general anaesthesia by either a paediatric surgeon or an interventional radiologist. Preoperative evaluation requires physical examination of the possible surgical sites to rule out local skin conditions. If previous multiple CVCs have been inserted, or the child has history of catheter infections or thrombosis, a preoperative ultrasonography with Doppler and/or a preoperative contrast computed tomography scan may be able to check the patency of the veins for procedure planning.

A chest X-ray is useful to reveal potential mediastinal mass, especially in leukaemia/lymphoma patients, that may complicate the procedure under general anaesthesia. The presence of mediastinal enlargement or elbow/thoracic wall tumours may lead to difficulty in achieving the optimum position of the catheter. Therefore, it is advisable to initiate chemotherapy via a peripheral vein to shrink the mediastinal tumour so as to facilitate the CVC placement later. Normal serum haemoglobin, leucocyte/platelet count and coagulation parameters are required to avoid perioperative complications. Low absolute neutrophil count (ANC) is not a contraindication for CVC placement. A study from St. Jude Children’s Research Hospital reviewed the safety of CVC placement at diagnosis of acute lymphoblastic leukaemia (ALL). This study revealed that placement of a CVC is safe in children with ALL even when their ANC is <500/mm3 [3–6]. Absence of infection or antibiotic use should be checked before the procedure, in order to avoid catheter colonisation and loss.

## Surgery

### Peripherally inserted central catheters insertion:

PICC lines are threaded into central veins from peripheral veins in the upper (cephalic, basilic and median cubital veins) or lower (saphenous vein) limbs. It is possible to insert PICC lines under local anaesthesia in an older cooperative child or under sedation, without a general anaesthesia in younger children. Generally, PICC packs include a venous access canula, guidewire, peel-away catheter and the line itself and include a tape measure and instructions on insertion.

### Tunnelled line or port insertion

CVC placement procedures may be performed using the percutaneous or the cutdown techniques. The choice of the surgical technique is dependent on the surgeon’s experience and preference. For the purpose of this guideline, the percutaneous technique is preferred, for the vein can be used more times and long-term venous access is more easily achieved.

#### Percutaneous techniques

Percutaneous CVC placement procedures are performed in a standardised manner under general anaesthesia. The use of prophylactic preoperative antibiotics (cephalosporin or clindamycin, if cephalosporin allergy) is dependent on institutional protocol. The most frequently accessed sites are the internal jugular vein, subclavian vein and femoral vein. Venous anatomy is more favourable on the right side of the neck for catheter-positioning and avoids thoracic duct injury that may occur on the left side. The left side may be used when there has been venous thrombosis or a previous procedure on the right internal jugular vein, or when the venous anatomy has been distorted by the tumour.

The internal jugular vein (IJV) and subclavian vein are accessed with the supine patient positioned with a roll beneath the shoulders and the head turned slightly to the contralateral side. A roll beneath the buttocks and frog leg position is advisable when accessing the femoral vein.

In the operating theatre, ultrasound machines are highly advisable and a sterile probe or probe with a sterile plastic sheath is used to locate the vein and observe the needle, subsequently, the guidewire entering the vessel.

The vein is accessed by anatomical landmarks or ultrasound guidance. In centres where intraoperative real-time ultrasound is available, the ultrasound probe (within a sterile sheath) is placed on the skin over the vein to be canulated, which can be seen in both transverse and longitudinal aspects, and is useful in confirming vein patency. Where two lumens are seen – the vein and artery can be differentiated by gentle pressure on the probe which will compress the vein, but not the artery. Skin puncture and vein access are then performed under ultrasound guidance, and the guidewire can be placed and noted within the vein lumen in the longitudinal view. The guidewire is fed into the superior vena cava and its position is confirmed with fluoroscopy or conventional chest X-ray intraoperatively. The port or tunnelled line is inserted via an incision either on the anterior chest, avoiding the breast bud and tunnelled toward the guidewire access area using the instrument provided in the pack. The guidewire puncture wound needs a small extension to allow the tunnelled line to exit the skin. Seldinger technique and split-sheath are used for catheter placement, as illustrated in Figures 1–3.

The measurement of the desired length of the catheter may be done using fluoroscopy or anatomic landmarks.

The final position of the CVC is confirmed with fluoroscopy or conventional chest X-ray intraoperatively.

Guidelines state that the ideal level of lines inserted via the neck is near the junction of the Superior vena cava and the right atrium. Those inserted via the femoral vein should rest above the renal vein at the level of the first lumbar vertebra [1]. Once the tip position is confirmed, the port can be secured in an appropriate manner. In order to avoid the use of contrast, the use of radio-opaque catheters is advised. In addition, intraoperative fluoroscopy or conventional chest X-ray is useful in the detection of any possible immediate complications such as pneumothorax, haemothorax and catheter malposition.

Blood return through the catheter should be obtained at the end of the procedure and the CVC should be flushed with heparinised saline solution to avoid immediate postoperative catheter thrombosis.

#### Open or cutdown technique

Open techniques are used if percutaneous methods have failed, or in centres lacking experience or equipment in the form of ultrasound machines. Possible veins for cutdown technique are the external jugular vein, facial vein or the IJV. Some centres use the cephalic vein at the deltopectoral groove. The vein is controlled with proximal and distal vessel loops. The catheter is tunnelled from the chest wall into the operative site. The catheter is measured and cut at an appropriate length and passed through a small venotomy between the vessel loops. The venotomy may require upper ligation or may be closed around the catheter with interrupted sutures to create a seal.

## Postoperative Period

The immediate postoperative period is usually uneventful and pain is easily controlled in the first post-operative days. Meticulous postoperative management of the CVC device by a trained team is critical to avoid complications such as catheter dysfunction, infection and thrombosis. Catheters should be fixed, cleaned, dressed and accessed with infection control measures in accordance with local hospital policies.

## Complications

In order to avoid accidental removal, extra care should be taken while securing the line. Correct training of caregivers is imperative to prevent catheter dysfunction and infection.

Intraoperative and early complications include arterial puncture, haemothorax, pneumothorax, catheter malposition, cardiac arrhythmia and thoracic duct injury. Catastrophic life-threatening events have been described in the literature [7], such as massive haemothorax and haemopericardium due to atrial perforation by the dilatator or split-sheath during catheter placement or later due to the presence of the catheter, especially in small children. Such situations require damage-control expertise performing prompt and precise invasive procedures, such as thoracotomy, sternotomy and/or pericardium fenestration.

Late complications include infection, device extrusion, catheter fracture/embolism [8] and catheter malfunction.

The use of CVC is associated with complications, including infection, catheter malfunction and thrombosis [8, 9]. The incidence of complications during *de novo* insertion of CVCs in paediatric cancer patients has been described as high as 20% [10]. Paediatric cancer patients are at high risk for potential complications, given their compromised immune status [11]. They have central line-associated bloodstream infection (CLABSI) rates as high as 18%. Other catheter-related complications, such as malfunction and dislodgement, are described in the literature at a range from 4% to 20% during *de novo* insertion of CVCs [12].

Regarding the choice of vein used, there have been several studies examining the relative merits and common complications of CVC placement in the various venous locations, particularly comparing the subclavian vein versus the internal jugular vein. Most of these studies have been performed in adult oncology and critical care patients and generally support a higher infection rate in internal jugular lines and a higher thrombosis rate in subclavian lines. This is less consistent in paediatric studies, and there is a high variability of factors assessed when determining overall safety and complication rates between sites of access. In considering the relative incidence of CLABSI by site placement, femoral catheters have been associated with increased CLABSI rates.

Catheter extravasation needs surgical review and catheter replacement. Catheter fracture/embolism can occur during treatment or at catheter withdrawal, which may need radio-intervention procedures in order to remove the residual catheter from the patient [13, 14].

## Tips and Pitfalls

- Guidewire-catheter exchange in paediatric cancer patients does not appear to increase CLABSI rate and may maintain a low risk of CLABSI while decreasing potential complications associated with *de novo* insertion. This is particularly important in paediatric patients with difficult venous access [11].
- Ultrasound guidance for CVC placement represents a helpful tool to avoid arterial puncture, pneumothorax and haemothorax.
- Use of fluoroscopy or conventional chest X-ray during the CVC placement represents a helpful tool to avoid line-malposition.
- Expertise in damage control manoeuvres to deal with eventual catastrophic complications.
- Experienced nursing team care is of essence to prevent catheter dysfunction and infection.

## Figures and Tables

**Figure 1.** Placement of introduce set over the guidewire.

**Figure 2.** Peeling split-sheath while inserting catheter.

**Figure 3.** Anchoring the port on the chest.

## References

[1] Milford K, Delft D, Majola N (2020). Long-term vascular access in differently resourced settings: a review of indications, devices, techniques and complications. Pediatr Surg Int.

[2] Kim HJ, Un J, Kim KH (2010). Safety and effectiveness of central venous catheterization in patients with cancer: prospective observational study. J Korean Med Sci.

[3] Samaras P, Dold S, Braun J (2008). Infectious port complications are more frequent in younger patients with hematologic malignancies than in solid tumor patients. Oncology.

[4] Cesaro S, Corro R, Pelosin A (2004). A prospective survey on incidence and outcome of Broviac/Hickman catheter-related complications in pediatric patients affected by hematological and oncological diseases. Ann Hematol.

[5] VanHouwelingen LT, Veras LV, Lu M (2019). Neutropenia at the time of subcutaneous port insertion may not be a risk factor for early infectious complications in pediatric oncology patients. J Pediatr Surg.

[6] Gonzalez G, Davidoff AM, Howard SC (2012). Safety of central venous catheter placement at diagnosis of acute lymphoblastic leukemia in children. Pediatr Blood Cancer.

[7] Goutail-Flaud MF, Sfez M, Berg A (1991). Central venous catheter-related complications in new borns and infants: a 587 case survey. J Pediatr Surg.

[8] Ribeiro RC, Abib SCV, Aguar AS (2012). Long-term complications in totally implantable venous Access devices: randomized study comparing subclavian and internal jugular vein puncture. Pediatr Blood Cancer.

[9] Kelly MS, Conway M, Wirth KE (2013). Microbiology and risk factors for central line-associated bloodstream infections among pediatric oncology outpatients-a single institution experience of 41 cases. Am J Pediatr Hematol Oncol.

[10] Allen CR, Holdsworth MT, Johnson CA (2008). Risk determinants for catheter-associated blood stream infections in children and young adults with cancer. Pediatr Blood Cancer.

[11] Bamba R, Lorenz JM, Lale AJ (2014). Clinical predictors of port infections within the first 30 days of placement. J Vasc Interv Radiol.

[12] Fernandez-Pineda I, Ortega-Laureano L, Wu H (2016). Guidewire catheter exchange in pediatric oncology: indications, postoperative complications, and outcomes. Pediatr Blood Cancer.

[13] Patel PA, Parra DA, Bath R (2016). IR Approaches to difficult removals of totally implanted venous access port catheters in children: a single-center experience. J Vasc Interv Radiol.

[14] Wilson GJP, van Noesel MM, Hop WCJ (2006). The catheter is stuck: complications experienced during removal of a totally implantable venous access device. A single center study in 200 children. J Pediatr Surg.

# Discussion: Neuroblastoma

**Published:** 2022

**Authors:** Amos Loh, Derek Harrison, Justin T Gerstle, Michael LaQuaglia, Cristina Martucci, Alessandro Crocoli, Stefano Avanzini, Lucas Matthyssens, Rose Dantas, Hau D Le, Riccardo Rizzo, Akihiro Yoneda, Sergio Vegas Salas, Christa Grant, Luca Pio

## Epidemiology

Neuroblastoma is the most common cancer in infants, and the most common extracranial solid malignant tumour of childhood. Approximately 30% of neuroblastomas present before 1 year of age, and 90% before 5 years of age [1]. Although less common in low- and middle-income countries, they present with a higher incidence of high risk and advanced disease [2, 3].

## Preoperative Evaluation

### Clinical presentation

Neuroblastoma is an embryonal sympathetic nervous system tumour that may present at a variety of anatomical locations due to its origin from neural crest progenitors, most commonly in the adrenal glands (48%) and along bilateral sympathetic chains (22%–25%), commonly presenting as an abdominal mass, and occasional spinal cord compression or paresis of the lower limbs. In particular, pelvic tumours (3%–5%) can present with bladder or bowel obstruction/dysfunction, cervical tumours (3%–5%) can present with Horner’s syndrome or airway obstruction and thoracic tumours (16%–20%) are often incidentally detected on chest radiographs. Common constitutional and systemic symptoms include arterial hypertension, anaemia-induced malaise, fever and bone pain. Up to 70% can present with metastases [4]. Unique and characteristic manifestations of metastatic disease include: ‘raccoon eye’ periorbital ecchymoses from orbital disease, bluish ‘blueberry muffin’ subcutaneous nodules typically seen in stage 4S (International Neuroblastoma Staging System (INSS)) or MS (International Neuroblastoma Risk Group Staging System (INRGSS)) disease, and paraneoplastic syndromes such as chronic diarrhoea due to hypersecretion of intestinal vasoactive peptide, and opsoclonus-myoclonus syndrome with jerking movements of the limbs and trunk and rapid conjugate eye movements [5].

### Initial workup

Lab (blood): Complete blood count (as an indicator of potential marrow disease), coagulation profile (anticipating the need for surgical biopsy), lactate dehydrogenase, ferritin and neuron specific enolase (as surrogate prognostic indicators) [6].

Lab (urine): Urinary catecholamines and metabolites (spot, or 24-hour collection; found in >85% of neuroblastoma patients): especially homovanillic acid and vanillylmandelic acid (per mmoL creatinine) (for diagnosis, and as markers of disease response) [7, 8].

Imaging studies: to evaluate extent of primary and distant disease (Table 1).

## Diagnosis

A confirmatory diagnosis of neuroblastoma is made from either:

- A histopathological diagnosis made from tumour tissue or
- Presence of tumour cells in bone marrow trephine samples, and increased urine or serum catecholamines or metabolites.

### Staging

Pre-treatment staging should be performed according to the newer INRGSS based on image defined risk factors (IDRFs) [10, 11], and post-operatively according to the INSS [12] (Tables 2 and 3).

### Risk stratification

Risk group allocation is assigned based on the INRG stage, age, histopathology, molecular biology and the International Neuroblastoma Risk Group Classification System [10, 11], or risk stratification systems of local protocols [13].

### Critical information for surgical planning

At initial evaluation, important surgical imaging features are: site of the primary tumour, invasion or mass effect on regional structures and organs and suitability for upfront resection or biopsy. The latter is determined based on the presence or absence of ‘image defined risk factors’ (IDRFs) [9], one component of the INRGSS. The number of IDRFs directly correlates with the degree of potential morbidity of the operation and is inversely related to the chance of complete resection [14–18](Annex A).

## Indications for Surgery

Surgical intervention in neuroblastoma may be required for the following indications:

- **Biopsy**: for the establishment of histological and molecular diagnosis at the initial diagnosis of the disease, or at detection of a lesion suggesting potential relapse.
- **Resection**: for local control of the disease aiming at removing all visible and palpable tumour (gross total resection (GTR)) [19], or resection of equal or greater than 90 % of the primary tumour [20], while ensuring minimal morbidity.

Decisions for surgical intervention should take into consideration stage of disease:

- In localised disease, upfront resection can be considered for tumour stages INSS 1, 2A, 2B and INRGSS L1. This decision is based on the site and extent of the tumour on preoperative imaging, the experience of the surgeon and the operative and perioperative supportive care resources available. Upfront surgical resection is only of benefit if the tumour is deemed to be completely resectable in a single setting [4].
- In advanced stage disease (INSS 3, 4 and INRGSS L2, M), initial surgical intervention should be limited to biopsy only, to obtain tissue for histological and molecular diagnosis. Resection should be performed only after neoadjuvant therapy as this increases the likelihood of successful complete resection and decreases the potential morbidity of surgery [21–23],.
  - In stage INSS 4S or INRGSS MS disease, where there is a possibility of spontaneous regression of the disease [24], after tumour biopsy, most patients are observed or treated with systemic chemotherapy and/or radiation [25]. While surgical resection is not usually recommended (if molecular evaluation and follow-up are feasible, if not, it is safer to resect the tumour), in the event of tumour progression associated with hepatomegaly or abdominal compartment syndrome, supportive intervention such as placement of abdominal silo can be considered.

Decisions for surgical intervention should also take into consideration the risk group of the patient:

- In low-risk disease, particularly in infants younger than 18 months, small localised or benign tumours which are not growing on serial surveillance and not causing significant morbidity from locoregional mass effect, may be observed safely with appropriate follow-up [26, 27]. Surgical resection for low-risk INSS 1, 2A, 2B is all that may be required [28], and INRGSS L1, <5 cm in size may be removed using minimally invasive techniques [29–31].
- In intermediate-risk disease with advanced stages (INSS 3, 4 and INRGSS L2, M), resection should be performed only after neoadjuvant therapy, with the aim of performing the most complete resection possible (50%–90%/95%), but with the extent of resection making no difference on outcome, organ preservation and function must be maintained by leaving residual disease around critical structures [32–35].
- In high-risk disease, the potential survival benefit conferred by GTR remains debated. In general, large studies have shown that after induction chemotherapy, GTR (>90%) is associated with prolonged event-free and overall survival with decreased rates of local disease progression [19, 20, 36], though other studies show no significant differences in disease and survival outcome [37]. Nevertheless, significant complication rates (up to 10%–20%) have been associated with these radical surgeries and should be duly considered in preoperative decision making and tumour board discussions [35, 38, 39].

## Perioperative Management

### Considerations for diagnostic biopsy

Image-guided percutaneous biopsy using a core (tru-cut) needle is the recommended approach to obtain a sufficient number of representative tissue samples for initial histological and molecular diagnosis, where expertise and resources allow [40, 41]. A minimally invasive or open surgical biopsy is only necessary if percutaneous image-guided true-cut biopsy is not possible, or the obtained tissue is not sufficient/representative [40]. The fresh biopsy sample should be sent for testing for MYCN (v-myc myelocytomatosis viral related oncogene, neuroblastoma derived) amplification and chromosomal alterations of 1p and 11q.

### Role and timing of local control

Local control surgery should be performed towards the end of induction chemotherapy, optimally after cycle 4 [42]. In most protocols, it is performed after peripheral blood stem cell harvest and before (or after, depending on the protocol used) high dose chemotherapy and stem cell transplant. In general, surgical resection should be performed only when metastatic disease (particularly in the bone marrow) is deemed to have resolved or has shown substantial response – as evidence of efficacy of the chemotherapy regimen for control of metastatic disease, and in view of the interruption of systemic therapy that will result from surgery. In cases where the primary disease shows poor response or large numbers of IDRFs remain, multidisciplinary tumour boards should discuss and determine the appropriate treatment. It should be noted that further reduction of tumour size is often limited past 3–5 cycles of neoadjuvant chemotherapy, and therefore further systemic treatment may only rarely substantially reduce the complexity of surgery [14, 43]. Delayed surgery after high dose chemotherapy and stem cell transplant may also be feasible [44].

### Preoperative considerations

Peri-operative hypertension due to catecholamine release is rare (<3%), in contrast to pheochromocytomas, and although considered routine in pheochromocytoma surgery (please refer to *Rare Tumours – Phaeochromocytoma Guidelines*), extensive pre-operative preparation may not be regarded as necessary in neuroblastoma [45, 46]. General anaesthesia with arterial pressure monitoring is employed for most neuroblastoma resections, while biopsies can be performed with general or a combination of intravenous and local anaesthetic. Epidural anaesthesia or other regional blocks are helpful for perioperative pain relief. Pre-operative arterial hypertension is associated with involvement of the renal pedicle and necessitates careful intraoperative titration of intravenous adrenergic blockers and postoperative fluid management and pressors [47].

## Surgical Goals

The aim of local control surgery in neuroblastoma is complete removal of all visible and palpable tumour: GTR [19, 20], while avoiding operative complications: vascular accidents, injury to normal/critical structures or organ loss (particularly inadvertent nephrectomy). En bloc or radical surgery to achieve an R0 resection is not necessary, and organ preservation and function must be maintained [32–35, 48].

Since the major challenge of surgical resection in neuroblastoma is the problem of vessel encasement, a systematic approach is required to intentionally locate and control major vessels within the tumour mass, and remove intervening tumour. The basis of the surgical technique is that neuroblastoma tumours do not invade past the tunica adventitia of major blood vessels. Thus, a plane of dissection can be developed between the tumour and the tunica media. Notably, this dissection plane becomes more obvious following neoadjuvant chemotherapy, but can be obscured by prior radiation [23, 48].

### Key steps

The surgical approach is chosen based on the site and extent of the tumour. Surgical decision making based on IDRF and associated risks is an evolving area that is currently being evaluated by several groups [49].

**Minimally Invasive Surgery**: When tumours are small (<5 cm) and have no or limited IDRFs, a minimally invasive resection may be undertaken [29–31]. In the thorax, single lung ventilation is often required to provide adequate operating space for thoracoscopic excision of paraspinal tumours. In the abdomen, either transperitoneal laparoscopy or retroperitoneoscopy can be employed [50, 51] (Please refer to *Minimal Invasive Surgery Guidelines*).

**Open Surgery**: When an open surgical approach is adopted, adequate exposure must be obtained, ideally providing the surgeon access to major vessels proximal and distal to the operative site:

- **For cervicothoracic/superior mediastinal tumours**: Trapdoor incisions are favoured [52, 53], with division and lateral reflection of the upper sternum. Upon entry to the chest, critical structures such as the innominate and subclavian veins, and the brachiocephalic, subclavian and carotid arteries, phrenic, vagus, recurrent laryngeal nerves and brachial plexus are separately identified, isolated and retracted to gain access to the upper posterior mediastinum at the site of the inferior cervical (stellate) ganglion, where most of these tumours originate. Alternative approaches include anterior cervical trans-sternal approaches and clamshell thoracotomies [54, 55].
- **For thoracic paraspinal tumours**: Posterolateral thoracotomy is typically employed, or alternatively a thoracoscopic approach in selected cases where experience allows [56].
- **For thoraco-abdominal tumours**: Thoraco-abdominal/thoracophreno-laparotomy or extended rooftop (‘Mercedes Benz’) incisions are favoured, with early division of the diaphragm in an anteroposterior/radial direction. The thoraco-abdominal approach is generally well tolerated and provides superior exposure of these tumours which traverse both body compartments [57–59].
- **For abdominal and pelvic tumours**: Transverse abdominal incision is recommended, but a variety of subcostal, rooftop, thoraco-abdominal or pelvic incisions may be utilised depending on the craniocaudal extent of the disease. Upon entry to the abdomen, the ipsilateral colon is medialised by incising along the white line of Toldt, thereby entering the underlying bloodless plane between the mesocolon and retroperitoneum. More medial dissection often necessitates Kocherisation of the duodenum (from the right), or elevation of the spleen and tail of pancreas (from the left).

The process of dissecting tumour away from blood vessels is a key dimension of the operative treatment of neuroblastoma. Classically, three phases are described: vessel display, vessel clearance and tumour removal [48]:

- In the first phase (vessel display), typically, this process is commenced from the periphery of the tumour working inwards, taking reference from the anatomical position of the visible portions of vessels that emerge from the edges of the tumour. The tumour is split over the encased vessels that traverse the tumour, such that upon successful completion of this phase, at least part of the circumference of each encased vessel is visualised. The tumour and tunica adventitia are incised upon in order to enter the sub-adventitial plane, which defines the plane of dissection for the rest of the procedure.
- In the second phase (vessel clearance), the dissection plane between the tumour and tunica media is developed from the initial longitudinal exposure. The dissection is advanced along the length of the exposed vessel in 1–2 cm steps, thereby mobilising the vessel fully from the tumour. Upon completely freeing tumour off the entire circumference of a vessel, it should be progressively controlled with vessel loops, thereby gradually demarcating each of the major arterial and venous structures that were previously encased.
- In the third phase (tumour removal), with major vessels and normal structures identified and isolated, intervening tumour is removed, typically in a piecemeal fashion.

Upon completion of tumour extirpation, radio-opaque clips are placed to mark the most cranial, caudal, medial and lateral margins of the tumour bed, as well as any areas of residual tumour [60]. Regions with transected lymphatics such as in the retroperitoneum, or around the thoracic duct, may be oversewn with permanent sutures or overlaid with haemostatic agents to reduce the risk of postoperative chyle leak [61, 62]. Surgical drains may be left in the operative bed when significant risk of chyle leak is anticipated [63].

## Postoperative Considerations

Postoperative intravascular hypovolaemia from third-spacing and lymphatic leaks may be encountered. Thus, close monitoring of urine output and intravascular blood pressure monitoring should be continued in the first 1–2 postoperative days in a paediatric intermediate or intensive care setting. Pain relief should be maintained via epidural infusions, regional anaesthesia or patient (or nurse) controlled intravenous opioid infusions. It is helpful to resume lipid-containing enteral feeds before removing surgical drains, if present, in order to diagnose occult chyle leaks [62]. In general, postoperative recovery should be expeditious and not substantially delay resumption of adjuvant therapy.

## Complications

Key operative risks of local control surgery for neuroblastoma include major vascular injury and visceral ischaemia with potential for perioperative mortality of <0.5% [36].

Resection of adrenal tumours may incur a risk of inadvertent partial or total nephrectomy of 5%–9% [36, 64]. Historically, this risk is increased especially during upfront surgical resection [65]. Nephrectomy in these patients does lead to reduced renal function but has not been associated with compromise in disease or survival outcomes [66]. Encasement of major vessels as identified on IDRFs is also associated with increased risk of nephrectomy and associated complications such as thromboembolism and ureteral strictures [67].

Chylous ascites or chylothorax may occur particularly following resection of disease involving retroperitoneal lymphatics, around the thoracic duct or cisterna chyli. The risk for postoperative chyle leak increases with the extent of surgery [68]. Conservative management should be the preferred first-line treatment and not compromise oncological treatment and its outcome.

Persistent diarrhoea may result from autonomic denervation of the small bowel, particularly from resection of retroperitoneal disease around the coeliac axis and superior mesenteric artery (SMA) [69, 70].

Structured recording of any intra- and postoperative complications (especially within the first 30 days after surgery) is highly recommended. This is also an important part of the ‘International Neuroblastoma Surgical Report Form’ (INSRF), a multi-committee standard reporting system for neuroblastoma surgery [71].

## Tips and Pitfalls

Particular attention should be paid to the following points for primary tumour resections in these anatomical areas:

### Cervicothoracic region

Resection of cervicothoracic neuroblastoma is typically considered a technically challenging surgery, owing to the need for control of vascular and neural structures and the challenges of surgical access. Some authors have suggested a transmanubrial incision, which allows exposure of all the vascular and nervous structures (subclavian and jugular vein to the brachiocephalic vein, subclavian artery, phrenic and vagus nerves and control of the carotid artery and vertebral artery) [72].

### Thoracic (para-spinal)

Tumours in the para-spinal region may often be contiguous with an intra-spinal component in a ‘dumbbell’ configuration. Particularly in the chest, a significant risk of neurological compromise has been reported due to critical spinal cord compression, which can be intensified by tumour oedema or bleeding from upfront biopsy. If there is no symptomatic response to chemotherapy in 48 hours, surgical decompression is required. During subsequent local control surgery, combined multidisciplinary approaches involving intra-thoracic surgery and laminectomy/laminoplasty/laminotomy and neurosurgical resection from an intraspinal approach may be employed [73].

### Upper abdominal (central)

Tumours centred around coeliac axis and SMA origin often encase these midline branches of the abdominal aorta and place them at high risk of inadvertent injury. Dissection is best undertaken by following the plane first established from the surface of the aorta and following these key vessels distally towards the relevant organs and small bowel mesentery, in order to avoid vascular injury. Caution should be exercised with dissecting too far distally where the coeliac axis and SMA branches are less robust and at increased risk of vasospasm, though it is uncommon that tumours extend very far distally. Notably, in tumours of the mid/lower thoracic and upper lumbar region (T7-L3), the artery of Adamkiewicz may be encountered (usually in left-sided tumours when Adamkiewicz is encountered in >80% of cases). Injury to this vessel, particularly at its characteristic ‘hairpin’ turn, may cause ischaemia of the spinal cord from T7 to the conus medullaris. Some authors suggest performing preoperative spinal angiography in order to delineate the relationship of the artery of Adamkiewicz to the tumour for the purpose of guiding surgical resection [74].

### Adrenal and abdominal (para-spinal)

Surgeons should take care when dissecting tumour around the renal hila, particularly beyond the first branches of the renal hilar vessels. Small amounts of gross tumour may be left at these sites and marked by radio-opaque clips if and where deemed too risky, to avoid renal vascular injury that may lead to nephrectomy. IDRFs that may predict an increased risk of nephrectomy include: encasement and narrowing of renal vessels, delayed excretion, hydronephrosis and invasion of the renal pelvis and capsule and should be considered in surgical decision making, risk assessment and preoperative counselling [73].

Venous junctions such as between the renal vein and vena cava are also prone to iatrogenic vascular injury. In most cases, the gonadal veins may be ligated and excised if necessary, without much consequence. Lumbar arteries may be encountered during clearance of the aorta and they can often also be ligated and divided with the tumour. Abnormal anatomy of retroperitoneal venous structures may be associated with these tumours and may not be easily identified on preoperative imaging due to distortion and compression by the tumour [75].

### Pelvic

Pelvic neuroblastoma tumours arise from primitive pelvic sympathetic ganglia, such as the organ of Zuckerkandl. The resection strategy is mainly dictated by the tumour location and stage at diagnosis; the risk of complications (i.e. urinary and faecal incontinence related to injuries of sacral nerve roots) has previously reported as 15%–35% [76]. When dissection near to or resection of the sacral nerve roots is necessary, this should be limited to a unilateral approach [77].

When tumours do not involve the neurovascular structures of the pelvis, patients with localised tumours (stage I or II) may undergo GTR with relative ease. However, in cases of locally advanced disease, patients should be managed with neoadjuvant chemotherapy, partial resection in order to avoid neurovascular morbidity, followed by adjuvant therapy with equivalent outcomes to complete resection. It is crucial to consider the lack of survival advantage after gross total resection for advanced disease, coupled with the risk of injuring involved sacral nerve roots and pelvic vascular structures [78].

If the pelvic tumour extends below the peritoneal reflection, a combined pelvic perineal approach may be necessary. This approach involves the need to change the patient’s position from supine to prone to complete the surgery.

### Recurrent disease

Survival of children with recurrent neuroblastoma is very poor, and up to 50% of high-risk neuroblastoma will experience a relapse which is invariably fatal. These cases require careful board discussion taking into account previous chemotherapy, radiotherapy and/or surgical treatments. Resections in an area of prior surgery often carry a higher risk of complications [80].

In general, patients with local or delayed relapse may benefit from further conventional treatment, but for the patients with more extensive disease recurrence, salvage regimens or experimental therapies may have to be considered [80].

Recording of surgical details should adhere to the framework of the INSRF, a multi-committee standard reporting system for neuroblastoma surgery [71].

## Annex A

### Image-Defined Risk Factors in Neuroblastic Tumours

Ipsilateral tumour extension within two body compartments

- Neck-chest, chest-abdomen, abdomen-pelvis

Neck

- Tumour encasing carotid and/or vertebral artery and/or internal jugular vein
- Tumour extending to base of skull
- Tumour compressing the trachea

Cervico-thoracic junction

- Tumour encasing brachial plexus roots
- Tumour encasing subclavian vessels and/or vertebral and/or carotid artery
- Tumour compressing the trachea

Thorax

- Tumour encasing the aorta and/or major branches
- Tumour compressing the trachea and/or principal bronchi
- Lower mediastinal tumour, infiltrating the costo-vertebral junction between T9 and T12

Thoraco-abdominal

- Tumour encasing the aorta and/or vena cava

Abdomen/pelvis

- Tumour infiltrating the porta hepatis and/or the hepatoduodenal ligament
- Tumour encasing branches of the SMA at the mesenteric root
- Tumour encasing the origin of the coeliac axis and/or of the SMA
- Tumour invading one or both renal pedicles
- Tumour encasing the aorta and/or vena cava
- Tumour encasing the iliac vessels
- Pelvic tumour crossing the sciatic notch
- Isolated contact with renal vessels

Intraspinal tumour extension whatever the location provided that:

- More than one third of the spinal canal in the axial plane is invaded and/or the perimedullary leptomeningeal spaces are not visible and/or the spinal cord signal is abnormal

Infiltration of adjacent organs/structures

- Pericardium, diaphragm, kidney, liver, duodeno-pancreatic block and mesentery

Conditions to be recorded, but not considered IDRFs

- Multifocal primary tumours
- Pleural effusion, with or without malignant cells
- Ascites, with or without malignant cells

*Adapted from: Monclair T, Brodeur GM, Ambros PF, Brisse HJ, Cecchetto G, Holmes K, Kaneko M, London WB, Matthay KK, Nuchtern JG, von Schweinitz D, Simon T, Cohn SL, Pearson AD; INRG Task Force. The International Neuroblastoma Risk Group (INRG) staging system: an INRG Task Force report. J Clin Oncol. 2009 Jan 10;27(2):298-303; and Brisse HJ, McCarville MB, Granata C, Krug KB, Wootton-Gorges SL, Kanegawa K, GiammarileF, Schmidt M, Shulkin BL, Matthay KK, Lewington VJ, Sarnacki S, Hero B, Kaneko M, London WB, Pearson AD, Cohn SL, Monclair T; International Neuroblastoma Risk Group Project. Guidelines for imaging and staging of neuroblastic tumors: consensus report from the International Neuroblastoma Risk Group Project. Radiology. 2011 Oct;261(1):243-57.*

## Figures and Tables

**Table 1.** Assessment of extent of disease.

Disease site		Recommended tests
Primary tumour		Computed tomography (CT)/magnetic resonance imaging (MRI) scan and ^131/123^I-meta-iodo-benzyl-guanidine (^131/123^I-mIBG) scan, or ^18^FDG-positron emission tomography (^18^FDG-PET) (if mIBG non-avid or not available)
Metastatic sites	Bone marrow	Bilateral bone marrow trephine and aspirates
	Bone	^99^Tc bone scan
	Lymph nodes (typically retroperitoneal)	CT/MRI scan
	Liver and other abdominal sites	CT/MRI scan
	Cranial or spinal extension	MRI
	Lungs	Chest CT scan
	All sites	^131/123^I-mIBG scan, or ^18^FDG-PET (if mIBG non-avid)

FDG: Fluorodeoxyglucose

**Table 2.** International Neuroblastoma Risk Group Staging System (INRGSS).

Stage	Description
L1	Localised tumour not involving vital structures, as defined by the list of IDRFs, and confined to one body compartment.
L2	Locoregional tumour with presence of one or more IDRFs.
M	Distant metastatic disease (except stage MS tumour).
MS	Metastatic disease in children younger than 18 months, with metastases confined to skin, liver and/or bone marrow.

**Table 3.** International Neuroblastoma Staging System (INSS).

Stage	Description
1	Localised tumour with complete gross excision, with or without microscopic residual disease; representative ipsilateral lymph nodes negative for tumour microscopically. Nodes attached to and removed with the primary tumour may be positive.
2A	Localised tumour with incomplete gross excision; representative ipsilateral nonadherent lymph nodes negative for tumour microscopically.
2B	Localised tumour with or without complete gross excision, with ipsilateral nonadherent lymph nodes positive for tumour; enlarged contralateral lymph nodes negative microscopically.
3	Unresectable unilateral tumour infiltrating across the midline (beyond the opposite side of the vertebral column) with or without regional lymph node involvement, or midline tumour with bilateral extension via infiltration (unresectable) or lymph node involvement.
4	Any primary tumour with dissemination to distant lymph nodes, bone, bone marrow, liver, skin and/or other organs (except as defined for stage 4S disease).
4S	Localised primary tumour (as defined for stage 1, 2A or 2B disease) with dissemination limited to skin, liver and/or bone marrow (limited to infants younger than 1 year, marrow involvement of less 10%, of total nucleated cells, and mIBG scan findings negative in the marrow).

## References

[1] London WB, Castleberry RP, Matthay KK (2005). Evidence for an age cutoff greater than 365 days for neuroblastoma risk group stratification in the Children’s Oncology Group. J Clin Oncol.

[2] Van Heerden J, Kruger M (2020). Management of neuroblastoma in limited-resource settings. World J Clin Oncol.

[3] Stefan C, Bray F, Ferlay J (2017). Cancer of childhood in sub-Saharan Africa. Ecancermedicalscience.

[4] Van Heerden J, Hendricks M, Geel J (2019). Overall survival for neuroblastoma in South Africa between 2000 and 2014. Pediatr Blood Cancer.

[5] Hiyama E, Yokoyama T, Ichikawa T (1994). Poor outcome in patients with advanced stage neuroblastoma and coincident opsomyoclonus syndrome. Cancer.

[6] Cangemi G, Reggiardo G, Barco S (2012). Prognostic value of ferritin, neuron-specific enolase, lactate dehydrogenase, and urinary and plasmatic catecholamine metabolites in children with neuroblastoma. Onco Targets Ther.

[7] LaBrosse EH, Com-Nougue C, Zucker JM (1980). Urinary excretion of 3-methoxy-4-hydroxymandelic acid and 3-methoxy-4-hydroxyphenylacetic acid by 288 patients with neuroblastoma and related neural crest tumors. Cancer Res.

[8] Laug WE, Siegel SE, Shaw KN (1978). Initial urinary catechola- mine metabolite concentrations and prognosis in neuroblastoma. Pediatrics.

[9] Brisse HJ, McCarville MB, Granata C (2011). Guidelines for imaging and staging of neuroblastic tumors: consensus report from the International Neuroblastoma Risk Group Project. Radiology.

[10] Cohn SL, Pearson AD, London WB (2009). The International Neuroblastoma Risk Group (INRG) classification system: an INRG Task Force report. J Clin Oncol.

[11] Monclair T, Brodeur GM, Ambros PF (2009). The International Neuroblastoma Risk Group (INRG) staging system: an INRG Task Force report. J Clin Oncol.

[12] Brodeur GM, Pritchard J, Berthold F (1993). Revisions of the international criteria for neuroblastoma diagnosis, staging, and response to treatment. J Clin Oncol.

[13] Parikh NS, Howard SC, Chantada G (2015). SIOP-PODC adapted risk stratification and treatment guidelines: recommendations for neuroblastoma in low- and middle-income settings. Pediatr Blood Cancer.

[14] Avanzini S, Pio L, Erminio G (2017). Image-defined risk factors in unresectable neuroblastoma: SIOPEN study on incidence, chemotherapy-induced variation, and impact on surgical outcomes. Pediatr Blood Cancer.

[15] Monclair T, Mosseri V, Cecchetto G (2015). Influence of image-defined risk factors on the outcome of patients with localised neuroblastoma. A report from the LNESG1 study of the European International Society of Paediatric Oncology Neuroblastoma Group. Pediatr Blood Cancer.

[16] Cecchetto G, Mosseri V, De Bernardi B (2005). Surgical risk factors in primary surgery for localized neuroblastoma: the LNESG1 study of the European International Society of Pediatric Oncology Neuroblastoma Group. J Clin Oncol.

[17] Simon T, Hero B, Benz-Bohm G (2008). Review of image defined risk factors in localized neuroblastoma patients: results of the GPOH NB97 trial. Pediatr Blood Cancer.

[18] Yoneda A, Nishikawa M, Uehara S (2016). Can image-defined risk factors predict surgical complications in localized neuroblastoma?. Eur J Pediatr Surg.

[19] La Quaglia MP, Kushner BH, Su W (2004). The impact of gross total resection on local control and survival in high-risk neuroblastoma. J Pediatr Surg.

[20] Von Allmen D, Davidoff AM, London WB (2017). Impact of extent of resection on local control and survival in patients from the COG A3973 study with high-risk neuroblastoma. J Clin Oncol.

[21] Yoneda A, Nishikawa M, Uehara S (2016). Can neoadjuvant chemotherapy reduce the surgical risks for localized neuroblastoma patients with image-defined risk factors at the time of diagnosis?. Pediatr Surg Int.

[22] Irtan S, Brisse HJ, Minard-Colin V (2015). Image-defined risk factor assessment of neurogenic tumors after neoadjuvant chemotherapy is useful for predicting intra-operative risk factors and the completeness of resection. Pediatr Blood Cancer.

[23] Rich BS, McEvoy MP, Kelly NE (2011). Resectability and operative morbidity after chemotherapy in neuroblastoma patients with encasement of major visceral arteries. J Pediatr Surg.

[24] Tas ML, Nagtegaal M, Kraal KCJM (2020). Neuroblastoma stage 4S: tumor regression rate and risk factors of progressive disease. Pediatr Blood Cancer.

[25] Schleiermacher G, Rubie H, Hartmann O (2003). Treatment of stage 4s neuroblastoma--report of 10 years’ experience of the French Society of Paediatric Oncology (SFOP). Br J Cancer.

[26] Nuchtern JG, London WB, Barnewolt CE (2012). A prospective study of expectant observation as primary therapy for neuroblastoma in young infants: a Children’s Oncology Group study. Ann Surg.

[27] Hero B, Simon T, Spitz R (2008). Localized infant neuroblastomas often show spontaneous regression: Results of the prospective trials NB95-S and NB97. J Clin Oncol.

[28] Strother DR, London WB, Schmidt ML (2012). Outcome after surgery alone or with restricted use of chemotherapy for patients with low-risk neuroblastoma: results of Children’s Oncology Group study P9641. J Clin Oncol.

[29] Leclair MD, Lagausie P, Becmeur F (2008). Laparoscopic resection of abdominal neuroblastoma. Ann Surg Oncol.

[30] Kelleher CM, Smithson L, Nguyen LL (2013). Clinical outcomes in children with adrenal neuroblastoma undergoing open versus laparoscopic adrenalectomy. J Pediatr Surg.

[31] Mattioli G, Avanzini S, Pini Prato A (2014). Laparoscopic resection of adrenal neuroblastoma without image-defined risk factors: a prospective study on 21 consecutive pediatric patients. Pediatr Surg Int.

[32] Kohler JA, Rubie H, Castel V (2013). Treatment of children over the age of one year with unresectable localised neuroblastoma without MYCN amplification: results of the SIOPEN study. Eur J Cancer.

[33] Baker DL, Schmidt ML, Cohn SL (2010). Outcome after reduced chemotherapy for intermediate-risk neuroblastoma. N Engl J Med.

[34] Iehara T, Yagyu S, Tsuchiya K (2016). Residual tumor in cases of intermediate-risk neuroblastoma did not influence the prognosis. Jpn J Clin Oncol.

[35] Amano H, Uchida H, Tanaka Y (2018). Excellent prognosis of patients with intermediate-risk neuroblastoma and residual tumor postchemotherapy. J Pediatr Surg.

[36] Holmes K, Pötschger U, Pearson ADJ (2020). Influence of surgical excision on the survival of patients with stage 4 high-risk neuroblastoma: a report from the HR-NBL1/SIOPEN Study. J Clin Oncol.

[37] De Ioris MA, Crocoli A, Contoli B (2015). Local control in metastatic neuroblastoma in children over 1 year of age. BMC Cancer.

[38] Von Schweinitz D, Hero B, Berthold F (2002). The impact of surgical radicality on outcome in childhood neuroblastoma. Eur J Pediatr Surg.

[39] Fischer J, Pohl A, Volland R (2017). Complete surgical resection improves outcome in INRG high-risk patients with localized neuroblastoma older than 18 months. BMC Cancer.

[40] Campagna G, Rosenfeld E, Foster J (2018). Evolving biopsy techniques for the diagnosis of neuroblastoma in children. J Pediatr Surg.

[41] Overman RE, Kartal TT, Cunningham AJ (2020). Optimization of percutaneous biopsy for diagnosis and pretreatment risk assessment of neuroblastoma. Pediatr Blood Cancer.

[42] Rojas Y, Jaramillo S, Lyons K (2016). The optimal timing of surgical resection in high-risk neuroblastoma. J Pediatr Surg.

[43] Kushner BH, Kramer K, LaQuaglia MP (2004). Reduction from seven to five cycles of intensive induction chemotherapy in children with high-risk neuroblastoma. J Clin Oncol.

[44] Hishiki T, Fujino A, Watanabe T (2020). Definitive tumor resection after myeloablative high dose chemotherapy is a feasible and effective option in the multimodal treatment of high-risk neuroblastoma: a single institution experience. J Pediatr Surg.

[45] Kako H, Taghon T, Veneziano G (2013). Severe intraoperative hypertension after induction of anesthesia in a child with a neuroblastoma. J Anesth.

[46] Hernandez MR, Shamberger RC, Seefelder C (2009). Catecholamine-secreting neuroblastoma in a 4-month-old infant: perioperative management. J Clin Anesth.

[47] Pio L, Avanzini S, Mattioli G (2017). Perioperative management of hypertensive neuroblastoma: A study from the Italian Group of Pediatric Surgical Oncologists (GICOP). J Pediatr Surg.

[48] Kiely E (2007). A technique for excision of abdominal and pelvic neuroblastomas. Ann R Coll Surg Engl.

[49] Iehara T, Yoneda A, Kikuta A (2020). A phase II JN-I-10 efficacy study of IDRF-based surgical decisions and stepwise treatment intensification for patients with intermediate-risk neuroblastoma: a study protocol. BMC Pediatr.

[50] Lee YT, Samsudin H, Ong CCP (2019). Posterior retroperitoneoscopic adrenalectomy for pediatric adrenal tumors. J Pediatr Surg.

[51] Benson Ham P, Twist CJ, Rothstein DH (2019). Retroperitoneoscopic resection of a T11-L2 right-sided ganglioneuroma. J Pediatr Surg.

[52] Pranikoff T, Hirschl RB, Schnaufer L (1995). Approach to cervicothoracic neuroblastomas via a trap-door incision. J Pediatr Surg.

[53] Chui CH, Thirugnanam A (2020). Trapdoor anterior thoracotomy for cervicothoracic and apical thoracic neuroblastoma in children. Pediatr Surg Int.

[54] Christison-Lagay ER, Darcy DG, Stanelle EJ (2014). "Trap-door" and "clamshell" surgical approaches for the management of pediatric tumors of the cervicothoracic junction and mediastinum. J Pediatr Surg.

[55] De Corti F, Avanzini S, Cecchetto G (2012). The surgical approach for cervicothoracic masses in children. J Pediatr Surg.

[56] Malek MM, Mollen KP, Kane TD (2010). Thoracic neuroblastoma: a retrospective review of our institutional experience with comparison of the thoracoscopic and open approaches to resection. J Pediatr Surg.

[57] Qureshi SS, Patil VP (2012). Feasibility and safety of thoracoabdominal approach in children for resection of upper abdominal neuroblastoma. J Pediatr Surg.

[58] Ross SL, Greenwald BM, Howell JD (2009). Outcomes following thoracoabdominal resection of neuroblastoma. Pediatr Crit Care Med.

[59] Martucciello G, Paraboschi I, Avanzini S (2020). Thoraco-abdominal neuroblastoma resection: the thoracophrenolaparotomic (TPL) approach. Gen Thorac Cardiovasc Surg.

[60] Halperin EC, Constine LS, Tarbell NJ (2011). Pediatric Radiation Oncology.

[61] Zeidan S, Delarue A, Rome A (2008). Fibrin glue application in the management of refractory chylous ascites in children. J Pediatr Gastroenterol Nutr.

[62] Chui CH (2014). Mesenteric lymphatic ligation in the prevention of chylous fistulae in abdominal neuroblastoma surgery. Pediatr Surg Int.

[63] Qureshi SS, Rent EG, Bhagat M (2016). Chyle leak following surgery for abdominal neuroblastoma. J Pediatr Surg.

[64] Lim II, Goldman DA, Farber BA (2016). Image-defined risk factors for nephrectomy in patients undergoing neuroblastoma resection. J Pediatr Surg.

[65] Shamberger RC, Smith EI, Joshi VV (1998). The risk of nephrectomy during local control in abdominal neuroblastoma. J Pediatr Surg.

[66] Fahy AS, Roberts A, Nasr A (2019). Long term outcomes after concurrent ipsilateral nephrectomy versus kidney-sparing surgery for high-risk, intraabdominal neuroblastoma. J Pediatr Surg.

[67] Warmann SW, Seitz G, Schaefer JF (2011). Vascular encasement as element of risk stratification in abdominal neuroblastoma. Surg Oncol.

[68] Liu Y, Pan C, Tang JY (2012). What is the result: chylous leakage following extensive radical surgery of neuroblastoma. World J Pediatr.

[69] Tokiwa K, Fumino S, Ono S (2003). Results of retroperitoneal lymphadenectomy in the treatment of abdominal neuroblastoma. Arch Surg.

[70] Rees H, Markley MA, Kiely EM (1998). Diarrhea after resection of advanced abdominal neuroblastoma: a common management problem. Surgery.

[71] Matthyssens LE, Nuchtern JG, Van De Ven CP (2020). A novel standard for systematic reporting of neuroblastoma surgery: The International Neuroblastoma Surgical Report Form (INSRF): A Joint Initiative by the Pediatric Oncological Cooperative Groups SIOPEN*, COG**, and GPOH**. Ann Surg.

[72] Sauvat F, Brisse H, Magdeleinat P (2006). The transmanubrial approach: a new operative approach to cervicothoracic neuroblastoma in children. Surgery.

[73] Pio L, Blanc T, Saint Denis T (2019). Multidisciplinary surgical strategy for dumbbell neuroblastoma: a single-center experience of 32 cases. Pediatr Blood Cancer.

[74] Nordin AB, Fallon SC, Jea A (2013). The use of spinal angiography in the management of posterior mediastinal tumors: case series and review of the literature. J Pediatr Surg.

[75] Tip SWM, Lee YT, Tang PH (2019). Retroperitoneal tumors and congenital variations in vascular anatomy of retroperitoneal great vessels. J Pediatr Surg.

[76] Cruccetti A, Kiely EM, Spitz L (2000). Pelvic neuroblastoma: low mortality and high morbidity. J Pediatr Surg.

[77] Todd LT, Yaszemski MJ, Currier BL (2002). Bowel and bladder function after major sacral resection. Clin Orthop Relat Res.

[78] Zobel M, Zamora A, Sura A (2020). The clinical management and outcomes of pelvic neuroblastic tumors. J Surg Res.

[79] Garaventa A, Parodi S, De Bernardi B (2009). Outcome of children with neuroblastoma after progression or relapse A retrospective study of the Italian neuroblastoma registry. Eur J Cancer.

[80] Cole KA, Maris JM (2012). New strategies in refractory and recurrent neuroblastoma: translational opportunities to impact patient outcome. Clin Cancer Res.

# Discussion: Wilms tumour

**Published:** 2022

**Authors:** Abdelhafeez H. Abdelhafeez, Simone Abib

## Evaluation

### Epidemiology

Wilms tumour (WT) is the second commonest childhood solid tumour and accounts for more than 90% of childhood renal tumours.

### Clinical presentation

Most children present with a large asymptomatic abdominal mass and rarely exhibit symptoms secondary to tumour rupture or extensive pulmonary metastasis. A few cases present with haematuria and hypertension. WT is also diagnosed during routine surveillance for patients with known predispositions, which include diffuse hyperplastic perilobar nephroblastomatosis (DHPN) and predisposition syndromes (WT1 related: WAGR (WT, aniridia, genitourinary anomalies and intellectual disability) (risk of WT 30%), Denys–Drash syndrome (risk of WT 90%); WT2 related: Beckwith–Wiedemann syndrome (risk of WT 5%), Perlman syndrome and Li–Fraumeni syndrome).

### Workup

Lab: Complete blood count, complete metabolic profile and coagulation profile.

Imaging: Chest radiograph or computed tomography (CT) chest, abdominal ultrasound and CT/MRI abdomen.

The ability to interpret cross-sectional imaging is essential for surgeons managing patients with WT. Differentiating between WT and neuroblastoma, examining vascular anatomy in relation to the tumour or determining the proximal level of intravascular extension are some competencies required for image interpretation. Basic information for surgical planning includes the following:

1. Evaluation of findings suggestive of WT or other differential diagnoses such as DHPN and neuroblastoma.
2. Relation of the renal tumour with surrounding organs and vascular structures.
3. Evaluation of preoperative tumour rupture and signs of abdominal dissemination and pulmonary metastasis.
4. Assessment of cystic areas that are prone to intraoperative rupture.
5. Evaluation of bilateral disease (5%) or coexisting urinary malformations (e.g. single or horseshoe kidney).
6. Evaluation of intravascular extension (10%–15%). If present, assessment of the level and presence or absence of blood flow around it and response to chemotherapy.
7. Evaluation of tumour response and pulmonary metastasis response to chemotherapy.

## Indications and Principles of Biopsy

A patient with suspected WT having a typical age and imaging findings will NOT require a diagnostic biopsy [1, 2]. The typical age of diagnosis for WT is more than 6 months and less than 7 years. Typical imaging features of WT include mass with renal origin and claw sign, absence of tissue infiltration and absence of vascular encasement.

Biopsy in the context of the typical presentation is not expected to change therapy, since it is unreliable for diagnosing anaplasia, unlikely to show alternative diagnosis [1–3] and may delay the initiation of therapy if performed routinely for all patients, especially in healthcare centres having limited pathology services. Tissue diagnosis (nephrectomy, if feasible; or biopsy) is required to plan therapy for patients who present at an atypical age or have atypical imaging features.

## Perioperative Management

### Role and timing of multimodality therapy

There are two main protocols to treat patients with WT: Children’s Oncology Group (COG) and the International Society of Paediatric Oncology (SIOP). Both protocols report similar survival results. The difference between the two protocols is the use of preoperative chemotherapy (SIOP) or upfront surgery (COG). Both protocols recommend upfront resection for patients less than 6 months of age, because of the relatively higher incidence of congenital mesoblastic nephroma and rhabdoid tumours in this age group (Table 1).

Neoadjuvant chemotherapy may mitigate bleeding, tumour rupture and the need for radiation therapy (RT) or doxorubicin [4–9]; therefore, this strategy may need to be considered if there is limited access to RT, high tumour spillage rate or limited surgical capacity [4].

### Preoperative considerations

Preoperative multidisciplinary planning should include the assessment of comorbidities, magnitude of the operation, capacity of the anaesthesia team, intraoperative monitoring, reliable upper extremity vascular access, urinary catheter, availability of blood, appropriate allocation of postoperative level of care and monitoring and postoperative pain control. If a neoadjuvant chemotherapy protocol is used, surgery should follow blood count recovery, and the planned timing of surgery should not be delayed [10, 11]. Treatment with vincristine should continue only when delay is unavoidable to prevent further delay of surgery due to neutropenia.

## Surgery

### Surgery goals

#### Early stages:

Goals of surgery (for all stages, including stage IV) are to perform and document a thorough surgical staging, achieve R0 resection, prevent tumour spillage and mitigate complications and resection of other organs.

#### Advanced stages and relapsed disease:

The outcome of chemo-responsive metastatic disease is favourable; therefore, there is no clear therapeutic need for upfront resection of metastatic sites. Examination of viable tumour in persistent pulmonary metastasis after chemotherapy may help guide therapy and prevent RT. Outcomes of patients with recurrent disease remain poor, and the role of surgery in recurrent disease is not defined.

### Key steps

Lower tumour rupture rate and fewer complications are reported when the surgeon performs a higher volume of resections for patients with WT [9]. Both transverse abdominal and thoracoabdominal incisions provide adequate access. The latter is associated with more complications [12], but may be considered for huge upper pole tumours that grow behind the liver. Midline incision provides limited access, resulting in a higher rate of tumour rupture and complications [9, 12, 13].

Surgery includes a staging component and local control components. Therapy depends on accurate documentation of surgical findings, including peritoneal seeding, tissue infiltration, lymph node sampling, capsular integrity and tumour spillage. Failure to sample lymph nodes is associated with local recurrence. Regional lymph nodes in the hilum, periaortic or peri-cava zones should be sampled even if they appear morphologically normal [14, 15]. A radical lymphadenectomy is not necessary, but proper lymph node sampling is crucial.

It is recommended that approximately seven lymph nodes be sampled [16]. This can be achieved by actively collaborating with pathologists to examine the lymph node found in the hilar area of the tumour specimen and the lymph node harvested from perivascular dissection (IPSO33 SIOP19-0533).

The initial step of tumour exposure involves mobilisation of the colon medially; en-bloc resection of the involved part of the colon is rarely needed. The adrenal gland and diaphragm can be resected en-bloc if they appear to be invaded by the tumour. Colon mobilisation is followed by lateral, superior and inferior mobilisation of the mass. The inferior-medial mobilisation exposes the ureter, aorta or vena cava. The ureter should be ligated and divided as close as possible to the bladder. The renal vein and vena cava should be palpated for intravascular tumour extension. Access to hilar vessels is facilitated by adequate circumferential tumour mobilisation. There is no reliable evidence supporting the theoretical advantage of early control of vessels before tumour mobilisation; on the contrary, this approach may obscure vascular anatomy due to limited exposure and increase the risk of major technical errors [12, 17–21]. Inadvertent injuries to major vessels are reported and intraoperative catastrophe can occur due to limited mobilisation and identification of critical vascular anatomy [12, 17–21]. Huge tumours distort anatomy, and it is paramount to delineate the anatomical landmarks of major branches of the aorta to prevent ligation of the contralateral renal vein or the superior mesenteric artery.

The renal vein and artery are ligated sequentially. The order of the vessel to be ligated first is likely not consequential, especially when the two vessels are controlled within a few minutes of each other. It may be easier and also a sound oncologic step to control the anteriorly positioned renal vein first, followed expeditiously by controlling the renal artery [22].

Intravascular tumour thrombus extension occurs in 15% of cases and involves only the renal vein in at least two thirds of cases. Tumour thrombus may extend to the vena cava and rarely to the atrium. Both protocols mandate neoadjuvant chemotherapy for tumour thrombus extending up to the hepatic vein or above. Neoadjuvant chemotherapy for 6 weeks induces thrombus reduction and may avoid the need for cardiopulmonary bypass in up to two thirds of patients with supradiaphragmatic extension of tumour thrombus; further extension of chemotherapy cycles beyond 6 weeks offers no added advantages [23–28]. Surgery for WT with intravascular extension should be performed at referral centres and in collaboration with the cardiothoracic team for patients with supradiaphragmatic extension of the thrombus. Although patients with supradiaphragmatic thrombus extension require cardiopulmonary bypass, for patients with transitional diaphragmatic level thrombus (up to the level of hepatic veins), cardiopulmonary bypass may be avoided by achieving supradiaphragmatic vena cava, hepatic veins control and complete liver isolation; however, bypass backup should be readily available if safe control above the thrombus was not achieved [23–28]. Cavotomy and thrombus resection is indicated when there is blood flow around the thrombus. On the other hand, a robust venous collateral drainage is already established whenever there is complete cava occlusion without flow. Cavectomy is the procedure of choice in this situation, as cava replacement is not physiologically needed and unlikely to remain patent because of shunting of venous return mostly through collaterals and the low flow through the cava route [29]. RT is required when the thrombus is resected piecemeal or when residual viable tumour is suspected. In facilities lacking the surgical capacity to resect the intravascular extension of the WT, RT may be the only option for local control of the intravascular component of the tumour.

Nephron-sparing resection is indicated for patients with a predisposition, such as bilateral WT, solitary kidney or horseshoe kidney. Neoadjuvant chemotherapy should be used for 6 or 12 weeks, depending on tumour response, but should not be extended for more than 12 weeks, as the poor response may be secondary to anaplastic histology [30].

The vascular and ureteric anatomy of horseshoe kidney is remarkably variable. Special precautions to delineate the collecting system anatomy need to be taken to prevent injuring the ureter of the contralateral kidney. Tactile feedback is instrumental in identifying tumour margin for nephron-sparing resection; therefore, an open approach is more widely accepted. Nephron-sparing resection is associated with risks such as urine leak, significant blood loss, positive resection margin and recurrence. A double J stent or perinephric drains are not routinely used but should be considered when resection involves complex collecting system reconstruction. Parenchyma pressure or intermittent hilar compression can be used to control blood loss and minimise ischaemia time; surface cooling can be used if the length of warm ischaemia is anticipated to be more than 30 minutes. Bench surgery and auto-transplantation are rarely needed.

Documentation for nephron-sparing resection should include assessment of the pseudo capsule breach, the type of resection (partial nephrectomy or enucleation) and the percentage of residual kidney.

Bilateral tumours should be treated in referral centres, and bilateral resection can be done in one operation or staged. Partial nephrectomy is more ontologically sound and is the procedure of choice when feasible; however enucleation is acceptable provided that anaplasia is ruled out. At least one adrenal gland should be preserved to maintain function.

Nephron-sparing surgery for unilateral WT and a minimal invasive approach are not yet evidence-based practice and should only be performed in centres with a high volume of cases and under established collaborative protocols.

## Tips, Pitfalls and Complications

Tumour spillage can result in significant therapy escalation and have prognostic implications. The key steps to prevent spillage are ensuring adequate access and gentle handling of the tumour. Attempts to minimise access should not be made at the expense of sound oncologic principles. Recovery after large laparotomy is excellent, but the recurrence of WT might be unsalvageable. Adherence to adequate lymph node sampling and complete documentation of surgical staging improves local control strategy and outcome.

Adequate planning and special skills set are required for WT with intravascular extension. Therefore, preoperative diagnosis of intravascular thrombus extension may prevent intraoperative catastrophes.

The incidence of end-stage renal failure in unilateral and bilateral WT is 0.2% and 12%, respectively, and this risk is higher in patients with predisposition syndromes, especially the Denys–Drash syndrome.

## Postoperative Considerations

The postoperative period is usually uneventful, and the child is usually discharged on the second or third postoperative day. The incidence of intussusception and adhesions is low and should be considered if the patient develops signs of obstruction.

Postresection locoregional RT is indicated for patients with stage III disease (when microscopic residual disease is suspected, as in positive lymph node, tumour spillage or piecemeal resection of the intravascular thrombus) and for stage II patients with anaplasia. RT, when indicated, should not be delayed beyond postoperative day 10.

Patients having complete remission of pulmonary metastasis post-chemotherapy do not need lung radiation [31–34]. When surgical resection of residual pulmonary metastasis is feasible, pathological confirmation of no viable tumour may help avoid lung radiation in patients with favourable histology and good partial remission of pulmonary disease [33].

## Prognosis, Prognostics and Follow-up

Overall survival of patients can be as high as 90%, and outcomes for those with metastatic-stage tumours with favourable histology are equally excellent. The most powerful prognostic factor is histology; other prognostic factors include stage, age and molecular factors, the strongest of which is 1q gain.

Surgery has an important role in performing adequate lymph node sampling and preventing tumour rupture. Lymph node involvement and tumour spillage increase the risk of recurrence; survival rate for those with recurrent disease is 40%. Biannual imaging follow-up for the first 2 years is essential, as most recurrences occur within that time period.

## Figures and Tables

**Table 1.** Comparison between COG and SIOP protocols.

	COG	SIOP
Preoperative chemotherapy	No (only in special situations: bilateral WT, solitary kidney, predisposition syndromes, inferior vena cava tumour thrombus extension to involve the level of hepatic veins, extensive symptomatic pulmonary metastases)	Yes (for renal tumours with typical clinical features)
Staging		
I	Tumour limited to the kidney and completely resected No rupture and no biopsy before surgery No involvement of the renal capsule or renal sinus vessels Lymph nodes status is known and negative	Tumour limited to the kidney or surrounded by a pseudocapsule if outside the normal contour of the kidney Renal pseudocapsule may be infiltrated but was completely resected and does not reach the outer surface of the kidney Tumour protrudes into the collecting system/ureter, but does not infiltrate their walls Intrarenal vessels may be involved Renal sinus vessels are not involved
II	Tumour extends beyond the capsule of the kidney and was not completely resected with no evidence of tumour at or beyond the margins of resection. Involvement of renal sinus and renal capsule Lymph nodes status is known and negative	Tumour extends beyond the kidney or penetrates the renal capsule and/or pseudocapsule into the perirenal fat, but is completely resected. Tumour infiltrates renal sinus and/or blood and lymphatic renal vessels outside the renal parenchyma but is completely resected. Tumour infiltrates adjacent organs or the vena cava, but is completely resected Tumour has been biopsied (wedge biopsy) before chemotherapy or surgery
III	Gross macroscopic residual tumour remains after surgery (inoperable, positive surgical margins, tumour spillage surfaces, regional lymph node metastasis, positive peritoneal cytology, transected tumour thrombus) Tumour rupture Tumour biopsy before removal	Incomplete resection of the tumour (gross or microscopic postoperative residual) Positive lymph nodes Tumour rupture (pre or intraoperatively) Tumour penetrated peritoneal surface Peritoneal tumour implants Tumour thrombi present at resection Margins of vessels or ureter transected or removed piecemeal by surgeon
IV	Haematogeneous metastases or lymph node metastases outside the abdomen (e.g. bone, lungs, liver, brain)	Haematogeneous metastases (bone, lungs, liver, brain) or lymph node metastases outside the abdominopelvic region
V	Bilateral renal tumours at diagnosis. Each side should be staged according to the above classification	Bilateral renal tumours at diagnosis. Each side should be staged according to the above classification

## References

[1] Irtan S, Ehrlich PF, Pritchard-Jones K (2016). Wilms tumor: “State-of-the-art” update, 2016. Semin Pediatr Surg.

[2] Schenk JP, Schrader C, Zieger B (2006). [Reference radiology in nephroblastoma: accuracy and relevance for preoperative chemotherapy]. Rofo.

[3] Hamilton TE, Green DM, Perlman EJ (2006). Bilateral Wilms’ tumor with anaplasia: lessons from the National Wilms’ Tumor Study. J Pediatr Surg.

[4] Israels T, Moreira C, Scanlan T (2013). SIOP PODC: clinical guidelines for the management of children with Wilms tumour in a low income setting. Pediatr Blood Cancer.

[5] Hadley GP, Shaik AS (2006). The morbidity and outcome of surgery in children with large pre-treated Wilms’ tumour: size matters. Pediatr Surg Int.

[6] Graf N, Tournade MF, Kraker J (2000). The role of preoperative chemotherapy in the management of Wilms’ tumor. The SIOP studies. International Society of Pediatric Oncology. Urol Clin North Am.

[7] Lemerle J, Voute PA, Tournade MF (1983). Effectiveness of preoperative chemotherapy in Wilms’ tumor: results of an International Society of Paediatric Oncology (SIOP) clinical trial. J Clin Oncol.

[8] Powis M, Messahel B, Hobson R (2013). Surgical complications after immediate nephrectomy versus preoperative chemotherapy in non-metastatic Wilms’ tumour: findings from the 1991–2001 United Kingdom Children’s Cancer Study Group UKW3 Trial. J Pediatr Surg.

[9] Fuchs J, Kienecker K, Furtwangler R (2009). Surgical aspects in the treatment of patients with unilateral wilms tumor: a report from the SIOP 93-01/German Society of Pediatric Oncology and Hematology. Ann Surg.

[10] Abuidris DO, Elimam ME, Nugud FM (2008). Wilms tumour in Sudan. Pediatr Blood Cancer.

[11] Israels T, Borgstein E, Pidini D (2012). Management of children with a Wilms tumor in Malawi, sub-Saharan Africa. J Pediatr Hematol Oncol.

[12] Ritchey ML, Shamberger RC, Haase G (2001). Surgical complications after primary nephrectomy for Wilms’ tumor: report from the National Wilms’ Tumor Study Group. J Am Coll Surg.

[13] Ehrlich PF, Ritchey ML, Hamilton TE (2005). Quality assessment for Wilms’ tumor: a report from the National Wilms’ Tumor Study-5. J Pediatr Surg.

[14] Kieran K, Anderson JR, Dome JS (2012). Lymph node involvement in Wilms tumor: results from National Wilms Tumor Studies 4 and. J Pediatr Surg.

[15] Othersen HB, DeLorimer A, Hrabovsky E (1990). Surgical evaluation of lymph node metastases in Wilms’ tumor. J Pediatr Surg.

[16] Vujanic GM, Gessler M, Ooms A (2018). The UMBRELLA SIOP-RTSG 2016 Wilms tumour pathology and molecular biology protocol. Nat Rev Urol.

[17] Ehrlich RM (1983). Complications of Wilms’ tumor surgery. Urol Clin North Am.

[18] Leape LL, Breslow NE, Bishop HC (1978). The surgical treatment of Wilms’ tumor: results of the National Wilms’ Tumor Study. Ann Surg.

[19] Ritchey ML, Kelalis PP, Breslow N (1992). Surgical complications after nephrectomy for Wilms’ tumor. Surg Gynecol Obstet.

[20] Ritchey ML, Lally KP, Haase GM (1992). Superior mesenteric artery injury during nephrectomy for Wilms’ tumor. J Pediatr Surg.

[21] Stehr M, Deilmann K, Haas RJ (2005). Surgical complications in the treatment of Wilms’ tumor. Eur J Pediatr Surg.

[22] Wei S, Guo C, He J (2019). Effect of vein-first vs artery-first surgical technique on circulating tumor cells and survival in patients with non-small cell lung cancer: a randomized clinical trial and registry-based propensity score matching analysis. JAMA Surg.

[23] Al Diab A, Hirmas N, Almousa A (2017). Inferior vena cava involvement in children with Wilms tumor. Pediatr Surg Int.

[24] Aspiazu D, Fernandez-Pineda I, Cabello R (2012). Surgical management of Wilms tumor with intravascular extension: a single-institution experience. Pediatr Hematol Oncol.

[25] Morris L, Squire R, Sznajder B (2019). Optimal neoadjuvant chemotherapy duration in Wilms tumour with intravascular thrombus: a literature review and evidence from SIOP WT 2001 trial. Pediatr Blood Cancer.

[26] Ribeiro RC, Schettini ST, Abib Sde C (2006). Cavectomy for the treatment of Wilms tumor with vascular extension. J Urol.

[27] Schettini ST, Fonseca JH, Abib SC (2000). Management of Wilms’ tumor with intracardiac extension. Pediatr Surg Int.

[28] Shamberger RC, Ritchey ML, Haase GM (2001). Intravascular extension of Wilms tumor. Ann Surg.

[29] Loh A, Bishop M, Krasin M (2015). Long-term physiologic and oncologic outcomes of inferior vena cava thrombosis in pediatric malignant abdominal tumors. J Pediatr Surg.

[30] Hamilton TE, Ritchey ML, Haase GM (2011). The management of synchronous bilateral Wilms tumor: a report from the National Wilms Tumor Study Group. Ann Surg.

[31] Kieran K, Ehrlich PF (2016). Current surgical standards of care in Wilms tumor. Urol Oncol.

[32] Dome JS, Graf N, Geller JI (2015). Advances in Wilms tumor treatment and biology: progress through international collaboration. J Clin Oncol.

[33] van den Heuvel-Eibrink MM, Hol JA, Pritchard-Jones K (2017). Position paper: Rationale for the treatment of Wilms tumour in the UMBRELLA SIOP-RTSG 2016 protocol. Nat Rev Urol.

[34] Verschuur A, Van Tinteren H, Graf N (2012). Treatment of pulmonary metastases in children with stage IV nephroblastoma with risk-based use of pulmonary radiotherapy. J Clin Oncol.

# Discussion: Rhabdomyosarcoma and non-rhabdomyosarcoma soft-tissue sarcoma

**Published:** 2022

**Authors:** Sandeep Agarwala, Jan Godzinski, Andrea Hayes

## Introduction

The soft tissues include connective tissues, lymphatics, vessels, smooth and striated muscles, fat, fascia, synovium, endothelium and reticuloendothelium. Tumours arising from any of these are soft tissue sarcomas (STSs) and these tumours behave in a very different biological manner from those tumours arising from blastemal elements. STS are uncommon in children, accounting for about 6% of all childhood malignancies. Most common of these are those arising from the immature mesenchymal cells that are committed to skeletal muscle lineage and are called rhabdomyosarcomas (RMS). The remaining group consists of a heterogenous collection of subtypes referred to as non-rhabdomyosarcoma STSs (NRSTSs).

# Discussion: Rhabdomyosarcoma

**Published:** 2022

**Authors:** Sandeep Agarwala, Jan Godzinski, Andrea Hayes

There is a bimodal incidence with almost two-thirds of cases of RMS being diagnosed in children <6 years of age with another mid-adolescence peak. RMS can occur almost everywhere (Table 1).

## Treatment of RMS

Treatment of children with RMS is multimodal including surgery, radiation therapy (RT) and systemic chemotherapy [1, 2]. While multidrug combination chemotherapy is used for primary cytoreduction and RT and surgery are used for local control of the disease either alone or in combination. RT and surgery may also be sometimes used for eradication of metastases.

All patients with RMS receive chemotherapy [3, 4]. Most active agents are actinomycin D (A), vincristine (V), cyclophosphamide (C) and doxorubicin (D). Other agents with moderate to high activity include melphalan, methotrexate, ifosfamide (I), cisplatin, carboplatin, etoposide (E), topotecan (T) and irinotecan (I). The gold standard multiagent combination has been the Vincristine, Actinomycin D, Cyclophosphamide (VAC) protocol that has been used by Intergroup Rhabdomyosarcoma Study Group (IRSG) for nearly three decades and is still the choice of treatment in the current Children’s Oncology Group (COG) protocols [5–7]. Other combinations that have been tried and compared with VAC are Vincristine, Actinomycin D, Ifosphamide (VAI) and Vincristine, Ifosphamide, Etoposide (VIE). Similar multi-agent protocols have been described by the International Society of Paediatric Oncology Malignant Mesenchymal Tumor Study Group, German Cooperative Weichteilsarkom Studiengruppe (CWS) and more recently European Pediatric Soft Tissue Sarcoma Group (EpSSG) and the COG-STS protocols. As the chemotherapy goes on for many weeks, the placement of a Hickman catheter or a Port-a-cath is useful.

RT is an important component of the multimodality management of patients with RMS as it improves local disease control and outcomes. In general all patients except low-risk subset A (clinical group (CG) I) receive RT. RT may be considered as an adjunct to surgery in case of microscopic residue (CG II) or gross residue (CG III) following biopsy, surgical resection or neoadjuvant chemotherapy. In certain sites, e.g., parameningeal RMS, orbital RMS, bladder-prostate RMS, RT is preferred to surgery for local disease control with an aim of achieving organ preservation. In patients with node positive disease, the involved lymph node (LN) region or site is included in the radiation portal.

Staging and risk categorisation for STSs (Tables 1, 2, 3, and 4)

Definitions:

Site:

Favourable sites: Orbit, head and neck (excluding parameningeal), or genitourinary (excluding bladder/prostate)

Unfavourable sites: Bladder/prostate, parameningeal, extremities, trunk and all others

Tumour: T1 = Tumour confined to anatomic site of origin

a) <5 cm in diameter

b) >5 cm in diameter

T2 = Extension and/or fixation to surrounding tissues

a) <5 cm in diameter

b) >5 cm in diameter

Regional nodes:

N0: Regional nodes not clinically involved

N1: Regional nodes clinically involved by tumour

Nx: Clinical status of regional nodes unknown (specially sites which preclude LN evaluation)

Metastasis:

M0: No distant metastases

MI: Metastases present

## Guidelines for Surgery for RMS

**Sampling for biopsy:** The biopsy for STS should preferably be obtained as an open incisional biopsy. Today with excellent interventional imaging techniques these can be done using co-axial core biopsy needles that can obtain multiple samples through one puncture that is guided by either ultrasound or computed tomography (CT) scan. During an open biopsy, ensure that under a general anaesthetic an adequate open incision is made at a carefully chosen site, that will be included in the eventual formal resection performed later. Careful incision is made through the capsule of the tumour if there is one. The tumour must be sent fresh to the pathology department because of the variety of biological and histochemical tests that need to be carried out on this type of tissue. All attempts at removal of the tumour en bloc at initial presentation should be resisted. The tumours are often gross at presentation and there is a real risk of compromising adjacent tissues, which may have been invaded by this tumour. Trunk and extremity biopsies should be performed along the long axis of the tumour so that subsequent excisions are not compromised (Please refer to *Role of Surgery in Paediatric Cancer Diagnosis Guideline*).

**Resection of primary tumour:** The goal of surgery for RMS is complete removal of the tumour, preserving the cosmesis and function as much as possible. Complete removal with no microscopic disease offers the best chance of cure. The surgical approach depends on the primary site, size, presence of LNs and distant metastases. At resection if positive margin is suspected, biopsy of the margin should be performed. Unresectable microscopic or gross residual disease should be marked with titanium clips in the tumour bed so as to direct re-excision and later RT and if required. Most tumours are usually unresectable at presentation and so will receive chemotherapy with or without RT for achieving reduction in size for a safe and complete surgery.

**Primary re-excision (PRE)** consists of complete re-excision of prior operative site with pathologically confirmed negative margins. It is prior to institution of any other adjuvant therapy. PRE is recommended for those where only biopsy was performed for a resectable tumour, or if a non-oncologic surgical excision was done and if the status of margins is unclear. In localised lesions of the trunk and extremities, PRE can lead to an improved survival.

**LN evaluation** is important for the planning of treatment and also for overall outcome as positive LN is an important independent poor prognostic factor for both failure free survival and overall surviva. Regional LN should be assessed both clinically, radiologically and for sites like extremities, pathologically also. Any suspicious LN requires pathologic confirmation. Nodal metastases are rare in head and neck disease (3%) and so routine regional LN biopsy is not mandated and only enlarged nodes should be biopsied. In RMS involving the extremities, the incidence of nodal metastases is high (40%–50%) and so these should have routine pathologic evaluation of the draining nodes. Axillary nodes for upper extremity and inguinal nodes for lower extremities. Nearly 17% of the nodes that are clinically negative can be pathologically positive and so even if no nodes are detected clinically or radiologically they need to be biopsied but complete LN removal has no therapeutic benefit. In the paratesticular RMS, the incidence of LN metastases is around 25%–30%. At this site, if the retroperitoneal nodes (iliac and para-aortic/paracaval) are greater than 2 cm on CT scans, they are considered positive and staged accordingly, and need not be biopsied. If the nodes are <2 cm, especially in children above 10 years of age, they should be biopsied. Retroperitoneal nodes above the level of renal hilum are considered as distant metastases (Stage 4 disease).

**Second look operation (SLO)**: Following initial multiagent neoadjuvant chemotherapy with or without RT, repeat imaging is performed followed by surgical exploration and excision of the residual primary tumour. This is called second look operation. SLO can reclassify a radiologic partial response (PR) to histologic complete response (CR) in 75% cases and this may eliminate the need for additional local measures like RT. Also about 10%–12% cases of radiologic CR may be found to have residual viable tumour and therefore, be reclassified as PR and these may require additional local therapy. SLO may even permit dose reduction or RT for patients that were initially CG III. SLO is most effective in RMS of the extremities and trunk and least useful in head and neck regions.

## Surgical Guidelines for Various Sites

**RMS of the head and neck regions:**

- These are rarely amenable for upfront surgical resection and so incisional biopsy is done.
- Routine regional LN biopsy is not required.
- For orbital tumours, biopsy followed by chemoradiotherapy is all that is mostly required.
- For all other sites, surgical excision may be required after tumour reduction is achieved with chemotherapy and radiotherapy.
- Regional LN dissection is not done, except for alveolar histology.
- Lymph nodal metastatic regions, if any, are included in radiation portal.

**RMS of the bladder and prostate:**

- The surgical approach for RMS of the bladder and prostate has evolved from pelvic exenteration in the 1960s and 70s to the current organ preserving surgeries that has been made feasible with the current multi-agent chemotherapy and RT.
- Upfront resection at these sites is reserved for a small proportion of patients who have tumour involving the dome of the bladder in whom the bladder and urethral functions can be preserved.
- Total cystectomy and anterior pelvic exenteration is now recommended only for those patients who fail to respond to induction chemotherapy and RT.
- After chemotherapy and RT, a number of patients with bladder/prostate RMS may not require extensive resections.
- Even with bladder preserving surgeries, the long-term bladder functions remains suboptimal in a number of survivors with urinary incontinence, frequency, nocturia and high pressure systems leading to subsequent renal damage.

**Paratesticular RMS:**

- All paratesticular tumours need to be resected, with the entire spermatic cord, through an inguinal incision, either upfront or following neoadjuvant chemotherapy.
- Any biopsy or excision through the scrotal route should be avoided as it will alter the lymphatic drainage basin and will require hemiscrotectomy.
- LN are considered involved if they are enlarged radiologically (>2 cm) or clinically and pathological confirmation biopsy is not required.
- Retroperitoneal LN (RPLN) biopsy and ipsilateral RPLN dissection are required only for children more than 10 years of age.

**Vulval, vaginal and uterine tumours:**

- For vulval, vaginal and uterine tumours, organ preservation is important and so primary resection has very limited role.
- Surgical resection is reserved for those who fail to achieve CR (radiographic) or have early disease progression on induction chemotherapy and RT.
- Residual tumour of the uterus and proximal vagina may mandate hysterectomy but distal vaginal preservation is nearly always feasible.
- Vaginal reconstruction may be required if vaginectomy is performed.

**RMS of the extremities:**

- For RMS of the extremities, upfront excision should only be done for small tumours that can be excised completely with negative margins and will not lead to major compromise of the function.
- All other should only have an incisional biopsy.
- Regional draining LN should always be sampled during the initial biopsy, even if they are clinically and radiologically not involved. Sentinel LN mapping and guided biopsy is the most accurate for identifying the LN where the meatastasis will be, if there is metastasis to the LNs.
- Sentinel LN mapping and guided biopsy requires technical expertise.
- Most patients will need excision of the tumour following neoadjuvant chemotherapy.
- Amputation may be required for those patients who fail to respond or in whom extensive tumours are involving the bone or neurovascular structures.
- Involvement of axillary nodes for upper extremity tumours and iliac or paraaortic nodes for lower extremity tumours are considered distal metastasis (Stage 4).
- Role of surgery in pulmonary metastases: please refer to pulmonary metastasis and thoracic tumour chapters

## Complications of Surgery

Radical LN dissections are not recommended as this leads to scarring and lymphoedema. There is no convincing evidence that radical LN dissections obviate the need for RT or even decrease its dosage. It does not improve outcomes. Resection in the head and neck region could result in major disfigurement unless performed skilfully. Resection done in the bladder base/urethra region can result in voiding difficulties and issues with urinary continence requiring bladder augmentation and/or need for CIC. RPLN dissection in cases of paratesticular RMS can result in ejaculatory dysfunction. Resection of extremity tumours can result in significant limb dysfunction at times requiring use of prosthesis.

## Figures and Tables

**Table 1.** RMS sites and incidences.

Region (incidence)	Favourable sites	Unfavourable sites
Head and Neck (35%)	Nonorbital non-parameningeal (30% – oral cavity, larynx, parotid region, cheek, scalp and soft tissues of the neck)	Parameningeal (44% – ear, mastoid, nasal cavity, paranasal sinuses, infratemporal fossa and pterygopalatine fossa)
	Orbital (25%)	
Genito-urinary (22%)	Vulva, vagina, uterus, paratesticular	Bladder, prostate, kidney and ureters
Extremities (14%)		All
Trunk (27%)		All
Others	Biliary	Retroperitoneum, perineum and perianal

**Table 2.** STS – Clinical grouping system used by the IRSG.

Group I: Localised disease, completely resected A. Confined to the organ or muscle of origin. B. Infiltration outside organ or muscle of origin; regional nodes not involved.
Group II: Total gross resection with evidence of regional spread A. Grossly resected tumours with ‘microscopic’ residual tumour. B. Regional disease completely resected with regional nodes involved, tumour extension into adjacent organs or both.
Group III: Incomplete resection or biopsy with gross residual disease remaining. A. Localised or locally extensive tumour, gross residual disease after biopsy only. B. Localized or locally extensive tumour, gross residual disease after ‘major’ resection (>50% debulking).
Group IV: Any size primary tumour, with or without regional lymphnode involvement, with distant metastases, irrespective of surgical approach to the primary tumour.

**Table 3.** STS-TNM (tumour, node and metastasis) pre-treatment staging classification for RMS.

Stage	Sites	T	Size	N	M
1	Favourable	T1 or T2	a or b	N0 or N1 or Nx	M0
2	Unfavourable	T1 or T2	a	N0 or Nx	M0
3	Unfavourable	T1 or T2	a	N1 M0	
b	N0 or N1 or Nx	M0			
4	Either	T1 or T2	a or b	N0 or N1	M1

**Table 4.** STS-Risk Categorisation for RMS.

Risk Group	TNM	CG	Site	Histology	Outcome (EFS)
Low Subset A	1 or 2	I or II	FH or UFH	ERMS	Excellent (<85%)
	1	III	Orbit only	ERMS	
Low Subset B	1	III	FH	ERMS	Very good (70%–85%)
	3	I or II	UFH	ERMS	
Intermediate	2 or 3	III	UFH	ERMS	Good (50%–70%)
	1–3	I–III	FH or UFH	ARMS	
High	4	IV	FH or UFH	ERMS or ARMS	Poor (<35%)

EFS, event-free survival; FH, Favourable site; UFH, Unfavourable site

(See Table 3 on TNM for a list of FH and UFH sites).

ERMS, Embryonal RMS; ARMS, Alveolar RMS

## References

[1] Wexler LH, Meyer WH, Helman LJ, Pizzo PA, Poplak DG (1899). Rhabdomyosarcoma. Principles and Practice of Pediatric Oncology.

[2] Austin MT, Andrassy RJ, Carachi R, Grosfeld JL (2016). Soft tissue sarcoma. The Surgery of Childhood Tumors.

[3] Raney RB, Walterhouse DO, Meza JL (2011). Results of the Intergroup Rhabdomyosarcoma Study Group D9602 protocol, using vincristine and dactinomycin with or without cyclophosphamide and radiation therapy, for newly diagnosed patients with low-risk embryonal rhabdomyosarcoma: a report from the Soft Tissue Sarcoma Committee of the Children’s Oncology Group. J Clin Oncol.

[4] Arndt CAS, Stoner JA, Hawkins DS (2009). Vincristine, actinomycin, and cyclophosphamide compared with vincristine, actinomycin, and cyclophosphamide alternating with vincristine, topotecan, and cyclophosphamide for intermediate-risk rhabdomyosarcoma: Children’s Oncology Group Study D9803. J Clin Oncol.

[5] Minn AY, Lyden ER, Anderson JR (2010). Early treatment failure in intermediate-risk rhabdomyosarcoma: results from IRS-IV and D9803—a report from the Children’s Oncology Group. J Clin Oncol.

[6] Pappo AS, Lyden E, Breitfeld P (2007). Two consecutive phase ii window trials of irinotecan alone or in combination with vincristine for the treatment of metastatic rhabdomyosarcoma: the Children’s Oncology Group. J Clin Oncol.

[7] Malempati S, Hawkins DS (2012). Rhabdomyosarcoma: review of the Children’s Oncology Group (COG) soft-tissue sarcoma committee experience and rationale for current COG studies. Pediatr Blood Cancer.

# Discussion: Non-Rhabdomyosarcoma Soft-Tissue Sarcoma

**Published:** 2022

**Authors:** Jan Godzinski, Sandeep Agarwala, Andrea Hayes

Among paediatric STSs, RMS is the most frequent. NRSTSs are commonly seen in adolescents and young adults (age 15–30 years).

Among more than 50 histological types of NRSTS, the chemotherapy insensitive tumours are the ones in which surgical local control is critically important [1]. Chemotherapy- or radiotherapy-sensitive tumours should be treated preoperatively to reduce tumour size as much as possible and therefore limit surgical morbidity [2].

## Surgical Principles

The surgery for STS has three competing tasks, namely, R0 (primary or secondary) resection, function-sparing and of less importance, the post-operative appearance. If we take these objectives as an outline for the guidance, NRSTS have to be divided into three groups according to available ‘choix des armes’: (1) chemo-radio-responsive (= RMS-like according to Cooperative Weichteilsarkom Studiengruppe (CWS), e.g. synovial sarcoma and extra-skeletal Ewing sarcoma), (2) incidentally or sometimes chemo- and radio-responsive (e.g. desmoplastic small round cell tumour) and (3) not responsive to those therapies (e.g. malignant peripheral nerve sheath tumour) [3–5].

1. **Chemo-radio responsive NRSTS should be treated according to the following:**
  - Primary excision only if the surgeon is certain of achieving complete (R0) resection, and the intervention will not be mutilating.
  - Secondary post-chemotherapy surgery is recommended in all the other situations, and this should be the surgeon’s preference in chemo- and radio-sensitive NRSTS.
  - In patients where complete and non-mutilating resection is not possible despite neoadjuvant chemotherapy, second-line chemotherapy or a pre-operative radiotherapy should be considered; if it fails – an aggressive, sometimes mutilating approach is justified.
  - Failure of the surgical completeness can be compensated (to some extent) by radiotherapy. Pre-operative radiotherapy may be effective in not only shrinking the tumour, but also providing an oedematous margin between the tumour and adjacent neurovascular structures. This can only be achieved successfully by planning the surgery 3–6 weeks after the end of radiotherapy. Surgery beyond this period may result in severe scarring and inability to identify the surgical planes. In addition, wound healing may be more impaired if surgery is delayed longer after radiotherapy. Adequate planning is required to optimise the balance between the surgical radicality and the function-sparing resulting in excellent local control.
  - Caution: The above approach is not appropriate for abdominal tumours. The potential damage to surrounding organs such as kidneys, small bowel, colon and bladder by radiation can be prohibitive. Complete surgical resection continues to be the standard, after chemotherapy in the abdomen.
2. **NRSTS incidentally/sometimes chemo- and radio-responsive:**
  - Surgery is the treatment of choice. Limited mutilation is acceptable especially if reconstructive surgery may realistically be applied.
  - In case of a life-threatening or markedly mutilating primary surgery, the options of neoadjuvant chemotherapy and/or radiotherapy should be submitted to a multidisciplinary board discussion.
  - The classical priorities must be kept in mind, in the following order: (1) life, (2) function and (3) appearance.
3. **NRSTS not responsive to chemotherapy or radiotherapy**
  Surgery is the only effective treatment. Neoadjuvant radiotherapy or chemotherapy is justified only when surgery is extremely mutilating, or the disease has spread and becomes life-threatening.

## Surgical Considerations

Majority of NRSTS are located in the limbs. Tumour-specific rules apply. Surgical resections should be well planned considering the following objectives [6–9]:

1. The tumour must be excised; R0 is optimal, R1 in radiosensitive histologies is acceptable, R2 is a failure.
2. Having perfect imaging is never about wasting time; however magnetic resonance imaging (MRI) or CT is not any treatment. Careful imaging with MRI and gadolinium contrast provides the most detail and should be done prior to surgical resection, particularly in tumours greater than 5 cm.
3. Alteration of function that may result from the resection should be considered.
4. It is important to ensure the availability of necessary surgical tools, such as neuromonitoring or nerve stimulation and intraoperative Doppler ultrasonography. Also, the expertise for plastic and reconstructive surgery and/or vascular reconstructive surgery should be anticipated prior to the surgery.
5. Is the group of muscles that you will be operating responsible for a unique and important function? – If ‘yes’ consider the possibility of switching a less important spared group of muscles to that resected. Example: the function of the deep flexors of fingers (upper limb) invaded by the tumour can be replaced by the superficial flexors by the tendon replacement.
6. If the patient is transferred to you from a low experience centre after a (primary) R1 resection of the mass, or after a doubtful R0, consider early re-resection if it can be completed in a non-mutilating way.
7. Chemo/radio-resistant and non-resectable tumours – Consider the possibility of mutilating surgery followed by reconstructions against the dynamics and life-expectancy. If still not acceptable, consider the targeted therapies and/or experimental studies. Always value the disease dynamics against possible benefits and harms of the experimental therapy.
8. Abdominal tumours/sarcomatosis such as Desmoplastic Small Round Cell Tumour (DSRCT). DSRCT is a rare NRSTS that holds the Ewing genetic signature. Its treatment is similar to Ewing sarcoma in that patients receive neoadjuvant chemotherapy according to the Ewing sarcoma protocol, for 12 weeks prior to re-imaging to assess the tumour response. If the tumours decrease in volume, then surgery should be planned for several cycles later, if the surgeon believes that 100% of the disease is resectable. If after chemotherapy there is persistent, active disease outside of the abdomen, surgery will only be of minimally benefit to the patient. After complete surgical resection (debulking does not extend life to the patient), as in Ewing disease, radiation should be added for microscopic residual disease. Typically 30 Gy is delivered to the whole abdomen after recovery from surgery and this is followed with non-bone marrow suppressive chemotherapy for a few more months. Adding hyperthermic intraperitoneal chemotherapy (HIPEC) to the surgical resection will help control the disease. It is important to use Cisplatin as the agent at 40.5°C –41°C as this is most effective and limits postoperative toxicity. Expertise in DSRCT as well as HIPEC is necessary for success [10].
9. The metastatic patients suffering from non-RMS STS should be submitted to the surgical treatment of their Mets only if all the lesions can be completely resected and the local control on primary is assured. Undertaking a surgery on Mets at progression has a very weak chance for success. The literature does not offer any real evidence-based data on this specific issue, but all three aspects (potential chemo- and radio sensitivity, and a chance for completeness) must be valued.

## References

[1] Cecchetto G, Carli M, Sotti G (2000). Importance of local treatment in pediatric soft tissue sarcomas with microscopic residual after primary surgery: results of the Italian Cooperative Study RMS‐88. Med Pediatr Oncol.

[2] Loeb DM, Thornton K, Shokek O (2008). Pediatric soft tissue sarcomas. Surg Clin N Am.

[3] Spunt SL, Million L, Chi YY (2020). A risk-based treatment strategy for non-rhabdomyosarcoma soft-tissue sarcomas in patients younger than 30 years (ARST0332): a Children’s Oncology Group prospective study. Lancet Oncol.

[4] Lautz TB, Hayes-Jordan A (2019). Recent progress in pediatric soft tissue sarcoma therapy. Semin Pediatr Surg.

[5] Venkatramani R, Xue W, Randall RL (2021). Synovial Sarcoma in children, adolescents, and young adults: a report from the Children’s Oncology Group ARST0332 study. J Clin Oncol.

[6] Morris CD, Tunn PU, Rodeberg DA (2020). Surgical management of extremity rhabdomyosarcoma: a consensus opinion from the Children’s Oncology Group, the European Pediatric Soft‐Tissue Sarcoma Study Group, and the Cooperative Weichteilsarkom Studiengruppe. Pediatric Blood Cancer.

[7] Godzinski J, Flamant F, Rey A (1994). Value of postchemotherapy bioptical verification of complete clinical remission in previously incompletely resected (stage I and II pT3) malignant mesenchymal tumors in children: International Society of Pediatric Oncology 1984 Malignant Mesenchymal Tumors Study. Med Pediatr Oncol.

[8] Hays DM, Lawrence Jr W, Wharam M (1989). Primary reexcision for patients with ‘microscopic residual’tumor following initial excision of sarcomas of trunk and extremity sites. J Pediatr Surg.

[9] Million L, Hayes-Jordan A, Chi YY (2021). Local control for high-grade nonrhabdomyosarcoma soft tissue sarcoma assigned to radiation therapy on ARST0332: a report from the Childrens Oncology Group. Int J Radiat Oncol Biol Phys.

[10] Hayes-Jordan AA, Coakley BA, Green HL (2018). Desmoplastic small round cell tumor treated with cytoreductive surgery and hyperthermic intraperitoneal chemotherapy: results of a phase 2 trial. Ann Surg Oncol.

# Discussion: Osteosarcoma and Ewing sarcoma

**Published:** 2022

**Authors:** Abdelhafeez Abdelhafeez, Florin Filip, Jan Godzinski

## Epidemiology

Osteosarcoma and Ewing sarcoma occur predominantly in adolescents and young adults (second decade) and account for 6% of all childhood cancer [1–3]. The incidence of osteosarcoma is approximately double the incidence of Ewing sarcoma, and the latter is seven times more common in Caucasian populations as opposed to other racial groups [4]. Ewing sarcoma and osteosarcoma affect the diaphysis and metaphysis, respectively. Long bones primarily account for more than half of both tumours and occur mostly in the lower extremity. Osteosarcoma rarely originates in soft tissue; however, in Ewing sarcoma extraosseous primaries include the trunk followed by the extremities, head and neck and retroperitoneum [5]. Ewing’s sarcoma possesses a specific gene translocation [Ewing sarcoma-Friend leukemia integration 1 transcription factor (EWS-FLI-1) fusion] which is responsible for tumour proliferation and transformation [6, 7]. On the other hand, osteosarcoma is rarely associated with genetic conditions such as Li–Fraumeni (p53) and the Retinoblastoma gene (RB1) [8–20].

## Preoperative Evaluation, Images, Special Needs, Biopsy and Indications for Surgery

Osteosarcoma and Ewing sarcoma most commonly present as an asymptomatic mass, although one third may present with pain. In active teenagers, a traumatic origin of the presenting complaint is frequently considered and may lead to delay in diagnosis. Initial assessment includes imaging and then biopsy, which confirms the diagnosis and allows proper staging of the tumour. As plain radiography is the hallmark of diagnosis, initial evaluation of X-rays should be thorough, especially in any unexplained limb pain. Magnetic resonance imaging (MRI) of the primary site is the imaging modality of choice to delineate tumour extent and plan the biopsy site.

20% of patients with osteosarcoma have metastatic disease at presentation of which 90% occur in the lung. Staging should include computer tomography scan of chest and nuclear medicine bone scan. Any bone metastasis other than the primary bone or across a joint should be considered distant metastasis rather than skip lesions [21].

One quarter of patients with Ewing sarcoma have metastatic disease at presentation, most commonly in the lung [2]. Ewing sarcoma also metastasises to the bone marrow in 5% of new patients. Bone marrow aspiration and biopsy is the gold standard to stage the presence of disease within the bone marrow, however, 18F-Fluorodeoxyglucose positron emission topography (18F-FDG PET) has a 100% negative predictive value. If FDG PET is available, then bone marrow aspiration and biopsy can be omitted in patients with localised disease [22]. FDG PET is more sensitive and specific than other staging imaging and significantly informs decision change [23].

Core needle biopsy is the diagnostic technique of choice. Review of the imaging is needed to plan the correct surgical approach to achieve representative biopsies [24–32]. Delays should be avoided as timely diagnosis is crucial for cancer control. Both biopsy and resection should be performed at a centre with orthopaedic oncology experience to minimise the risk of contaminating uninvolved tissues and to achieve adequate resection margins. Incisional open biopsy may sometimes be needed when core biopsy is nondiagnostic and should be performed through a small longitudinal incision that is most direct to the tumour and will fall within the planned resection incision (Please refer to *Role of Surgery in Paediatric Cancer Diagnosis Guidelines*).

## Surgical Goals

The surgical goals of resection of osteosarcoma and Ewing sarcoma are to achieve R0 resection margins. Management has evolved over the past three decades to improve preservation of function. Emerging effective neoadjuvant and adjuvant chemotherapy has been used to preserve uninvolved tissue, and, in conjunction with biological and non-biological bone and soft tissue reconstruction, these techniques have demonstrated a favourable oncologic outcome together with conservation of function [33]. Improvement of survival following inclusion of modern chemotherapy in the 1970s has shifted attention toward limb salvage surgery (LSS) techniques. LSS procedure is defined as successful resection of the tumour and reconstruction of a viable, functional extremity. Successful local control can be achieved in more than 95% of patients with LSS with comparable oncologic outcome to ablative surgery [33].

Surgery is the local control strategy of choice for osteosarcoma pulmonary metastases, and patients with osteosarcoma pulmonary metastases achieve survival benefit from pulmonary metastasectomy [34]. For unresectable osteosarcoma, radiation therapy serves as palliation of primary tumour or symptomatic metastases.

In Ewing sarcoma, pulmonary metastases whole lung radiation is the standard of care. Patients with pulmonary only metastatic Ewing sarcoma have a better prognosis. For unresectable tumours, radiotherapy can serve as the primary local control strategy for Ewing sarcoma; however, the reported local control failure is higher in selected patients when compared to surgery [35–39].

## Perioperative Management

For low-grade osteosarcomas, wide surgical excision is the only treatment needed. Neoadjuvant chemotherapy is the standard of care for both osteosarcoma and Ewing sarcoma to facilitate resection and evaluate tumour response to therapy. Vincristine, Adriamycin and cyclophosphamide alternating with ifosfamide and etoposide (VDC/IE) are the regime of choice for Ewing sarcoma [40–43]. The most active agents for osteosarcoma are high dose methotrexate, doxorubicin and cisplatin [44].

In selected cases of Ewing sarcoma where positive resection margin is anticipated, neoadjuvant radiation therapy is the armamentarium to improve precision of multimodal local control strategy [45–47].

Limb sparing surgery is associated with a high incidence of delayed wound healing, thus assurance of bone marrow recovery after chemotherapy is mandatory prior to scheduling resection.

Generally, reassessment images are obtained for preoperative planning after three cycles of neoadjuvant chemotherapy including plain X-rays, MRI of the primary and computed tomography (CT) of the chest. Preoperative MRI studies are valuable in determining the extent of tumour and relationship with major neuro-vascular bundles, detecting soft tissue involvement, skip lesions and response to therapy. Determining the proximal and distal tumour extent, joint involvement and proximity of epiphyseal plate are essential for preoperative planning, determining the level of resection, options for soft tissue reconstruction, selection of graft type and size.

## Surgical Approach

For patients with high-grade tumours to be eligible for LSS, clinical and radiological response to neoadjuvant chemotherapy should be demonstrated. The feasibility of LSS depends on the ability to resect with adequate margin and to reconstruct the extremity with preservation of satisfactory function. Also, LSS needs to be accomplished with minimal morbidity and early resumption of chemotherapy. The patient should also be assessed prior to surgery to evaluate social support and compliance with the postoperative protocol. Contraindications for limb salvage procedures include pan-compartmental involvement, gross infection and encasement of major neuro-vascular bundles. Ablative surgery in the form of amputation or disarticulation is indicated when complete resection with adequate margins is not feasible. Ablative surgery may also be indicated in areas with no access to the various available prosthetic appliances or complex surgical techniques. In this instance, sarcoma management comes at the cost of limb loss. The role of surgical resection versus primary radiotherapy for pelvic Ewing sarcoma is challenged by conflicting evidence; therefore, the anticipated morbidity of resection should be weighted in selection of local control strategy [48–50]. Selection of the extent of internal hemipelvectomy depends on the boundaries of tumour margin.

LSS procedure involves wide excision including the biopsy tract and the primary tumour en-bloc including surrounding reactive tissue and a circumferential cuff of normal tissue. The margin of bone resection of 2 cm away from the apparent bone marrow involvement as shown by the MRI studies is adequate; however, the proximal margin should also be evaluated with intraoperative frozen section. During the initial phase of the operation, the neuro-vascular bundle is identified, isolated and protected while preventing tumour rupture (Figure 1). Tumour anatomy will dictate bone resection type either osteoarticular, intercalary or whole bone resection. Since most osteosarcomas are of long bone metaphysis origin, osteoarticular resection is the commonest LSS approach. On the other hand, long bone diaphysis tumour location is common in Ewing sarcoma where intercalary resection with allograft replacement is utilised. Occasionally the entire bone is involved and to achieve a negative margin a whole bone resection with endoprosthesis reconstruction is elected.

Once en-bloc resection is completed, reconstruction of the bone and soft tissue defect proceeds. A successful reconstruction should be durable, restore a functional limb, allow rapid postoperative rehabilitation and compensate for skeletal growth when applicable. Local muscle flap reconstruction for adequate coverage of soft tissue defect is sometimes required especially for proximal tibia resection.

### i. Endoprosthesis

Endoprosthesis (Figure 2) is the most used technique for limb-salvage reconstruction. Several technical options are available, depending on the site of tumour resection. After resection of tumour around the knee, it is recommended to choose the rotation hinged custom prosthesis or the assembled prosthesis. In addition, bone cement or cementless fixation may be selected according to the patient’s bone condition. Bipolar hemiarthroplasty replacement is selected for proximal femur resection. For tumours of the proximal humerus, a Malawer type I resection is commonly used, and reconstruction is performed using a half-shoulder prosthesis. For other rare sites, an individual design is recommended for reconstruction.

It should be emphasised that the use of endoprostheses has many complications, such as aseptic loosening and infection, with high rates of biological and structural failure. Also, limb length discrepancy and joint dysplasia are long-term issues.

### ii. Biological reconstruction

**Allografts** – Bone allografts describe implantation of bone donated from a third-party, with the aim of integration with host bone. Structural grafts are load bearing and used to replace intercalary resection segments. They have been associated with high rates of complications such as non-union, infection and pathological fractures. Studies have shown that after 10 years, there is a 40% risk of allograft removal, joint replacement or amputation, with the risk highest for osteo-articular tibia grafts [51].

**Autografts** – Autograft is the implantation of a patient’s own bone tissue when reconstructing the resection defect. Tumour devitalised autografts and free-vascularised fibula grafts (FVFGs) are the two categories of autografts. Tumour devitalised autografts involve the reimplantation of tumour bearing bone after devitalisation, to fill the resection defect. Heating/cooling or radiation has been used to devitalise the grafts prior to reinsertion. Devitalisation can be achieved with pasteurisation or liquid nitrogen freezing. Freezing is a more effective method than pasteurisation, as it better preserves the osteoinductive ability of the graft. Tumour devitalised grafts have a similar rate of effectiveness and complications as allografts, with the advantage of being cheaper and more available.

FVFG is a well-established form of autograft used in OS cases. Given their vascular supply and biological nature, the graft continues to grow after implantation, whilst aiding bone union and providing better resistance to infection. FVFG appears to have better oncogenic abilities, aiding union and a decreased risk of infection, but decreased strength posing an increased risk of fracture.

**Graft combinations**, first known as Capanna technique (1993), represent the simultaneous use of allografts and FVFG. This aims to combine the structural advantages of the allograft with the vascular and osteogenic properties of the FVFG. Reported success rates were as high as 93%, with decreased risk of non-union and fracture (8.8% and 13.3%, respectively). While the classical Capanna technique combines allograft with the FVFG, later versions replaced the allograft with frozen tumour-bearing autograft for lower limb osteosarcoma, with similar functional and oncologic results but lower time needed for achieving bone union.

For proximal tibia resection, reconstruction of knee extensor mechanism is of utmost importance. Complications associated with proximal tibia resection are frequent: poor patellar tendon reattachment, infection, poor skin coverage, mechanical loosening and damage to neuro-vascular structures. To overcome this, normal patellar ligament may be preserved and attached to the prosthesis by a wire. To minimise the incidence of loosening, synovitis and trauma, allografts may be used. A medial gastrocnemius flap may be used to keep stretches stable and supply a comprehensive soft tissue coverage which promotes healing and decreased infection rate. Bone-muscle flap is used to stabilise the extensor mechanism. Rates of infection are variable, and infection is related to operative time, blood loss and wound complications. Immune suppression by chemotherapy and soft-tissue defects are also related to infection.

### iii. Extendable Endoprosthesis

Extendable endoprosthesis can be used for bone defects resulting from tumour resection in the distal femur or proximal tibia in children at the developmental age. To mitigate the need for repeated surgery, the design of extendable endoprosthesis allows for small incremental expansions that can be completed in the outpatient setting under sedation. However, structural failure of the prosthesis, infection and aseptic loosening are known complications.

### iv. Rotationplasty

Rotationplasty is a less frequently used LSS strategy for distal femur and proximal tibia tumour. This technique involves an *en bloc* resection of the tumour and fusion of the normal residual proximal femur to the normal residual tibia after 180° rotation of the distal tibia to allow the ankle to function as a knee joint. Complication rate of this approach is low and functional outcome is satisfactory.

## Complications

**Infection:** The risk of infection after LSS procedures is 8%–15%, with most of them being staphylococcal. Periprosthetic infections occur in 10% of the cases, most of them within the first 2 years after surgery. Proximal tibia and pelvic ring carry the highest risk of infection. Radiation therapy and expandable prostheses are also reported to be high risk factors for infection. Infection is usually treated with debridement and antibiotic therapy (both systemic and local cement antibiotic beads) in case of megaprosthesis infection. In case of failure, removal of the implant, followed by thorough debridement and lavage is recommended. Usually, an antibiotic impregnated cement spacer is placed before a new implant is inserted as a two-stage procedure. Amputation may be required when a conservative approach fails to resolve complications and restore function.

**Local recurrence:** Occurs in 5% of the patients with LSS in specialised centres. It carries a grim impact on the overall survival of the patients (5-year survival rate of 10%–40%), especially if it occurred within the first 2 years after initial surgery for high-grade osteosarcoma. Risk factors for local recurrence include failure to achieve clear margins at the time of surgery, poor histological response to chemotherapy and tumour growth during chemotherapy. Treatment depends on several factors, such as timing of recurrence or association with distant metastases. Both amputation and LSS procedures can be used as treatment options in such cases. It is recommended that the resection range of recurrent lesions be at least 1 cm beyond the normal tumour margins. Survival of patients who underwent LSS or amputation is equivalent, and LSS is associated with higher recurrence especially if margin adequacy was jeopardised [52].

**Implant failure:** Current data suggest a rate of implant survival between 50% and 90% at 10 years following surgery, with lower values for proximal tibia implants [53–57]. Aseptic loosening of the prosthesis intramedullary needle is the most common reason for failure of reconstruction with endoprosthesis, with an incidence of 5%–11% [53–57]. Mechanical failure of the prosthesis includes several problems, such as fracture of the prosthesis or dislocation of the hinge device. They occur in 3%–6% of the cases. Several technical achievements in the prosthetic technology (rotating platform design, hydroxyapatite coated collar and stem, porous tantalum and compression osteointegration technology) will probably overcome this kind of complications.

**Non-union:** The incidence of non-union and fracture of allograft bone is 12%–63% and 17%–34%, respectively. Risk factors for these complications include length of allograft bone over 15 cm, radiation sterilisation, simple or locking intramedullary nail fixation and diaphysis transplantation. Patients over 18 years old are more prone to develop this complication. The use of fibula vascularised grafts was shown to decrease the risk of non-union and fracture.

## Postoperative Considerations

Surgical wound healing time frame is usually within 2 weeks and postoperative adjuvant chemotherapy should be started as soon as wound healing progress is satisfactory. Evaluation of limb function after LSS is generally performed using the Musculoskeletal Tumor Society efficiency scoring system, which proved to be reliable and repeatable. It can also be used for 6-month assessment in patients with resection-prosthesis procedures.

In terms of rehabilitation, functional exercises can be started 24 hours after surgery. The specific method of rehabilitation depends on the surgical site and the reconstruction method. Special attention should be given to patients where ligament healing is being followed.

**Follow-up:** The patient should be monitored for local and systemic recurrence, as well as complications related to reconstruction. Loosening, infection and mechanical failure are the most common complications. Radiological studies are recommended for follow-up as follows:

- CT scan of the chest and plain-film X-ray of the reconstructed extremity every 3 months for the first 2 years after surgery, at least every 6 month for the next 3 years and subsequently on an early basis.
- Annual bone scintigraphy is recommended for the first 2 years following surgery.
- The physical examination of the reconstructed extremity should be carefully check for local masses. Complaints of pain, joint instability, joint effusion, prosthetic failure or local warmth/redness of the extremity may signal mechanical complications or infection. Also, radiological findings (osteolysis, radiolucent lines surrounding the prosthetic stem) suggest local complications.

Postoperative radiotherapy is indicated for intralesional resection of osteosarcoma when redo complete resection is not feasible. For Ewing sarcoma, postoperative radiation therapy is standard of care; however, patients can be spared radiation therapy if resection with >1 mm negative margin is achieved in the context of >90% post neoadjuvant therapy tumour necrosis.

## Tips

Performing the biopsy at an institution different from limb sparing centre is associated with increased recurrence rate because resection may be compromised when surgical approach alteration is required to accommodate the biopsy site [58, 59]. Referral network needs to be strengthened when considering implementation of a limb sparing programme.

Up to 25% of pulmonary nodules in patients with osteosarcoma are benign; histologic confirmation is especially required in the absence of typical characteristics of metastases [60].

## Pitfalls

Delaying chemotherapy for more than 21 days after definitive surgery is associated with poor outcome. Limb sparing surgery may jeopardise the chance of cure when resumption of chemotherapy is delayed by healing issues related to complex reconstruction. Therefore, the decision of surgical local control strategy needs to be weighed with healing time frame, planned resumption of chemotherapy, wound complication rate and available resources. Tumour biopsy strategy should facilitate future *en bloc* resection of biopsy tract at the time of local control and avoid field contamination, debulking or drains.

## Figures and Tables

**Figure 1.** Neurovascular bundle isolation during limb sparing surgery.

**Figure 2.** Limb sparing surgery with endoprosthesis reconstruction for distal femur osteosarcoma. (a) and (b): X-ray and MRI picture of osteosarcoma of distal femur. (c) and (d): On table assessment of resected distal femur segment and reconstructed bone defect.

## References

[1] Mirabello L, Troisi RJ, Savage SA (2009). Osteosarcoma incidence and survival rates from 1973 to 2004: data from the surveillance, epidemiology, and end results program. Cancer.

[2] Esiashvili N, Goodman M (2008). Changes in incidence and survival of Ewing sarcoma patients over the past 3 decades: surveillance epidemiology and end results data. J Pediatr Hematol Oncol.

[3] Ward E, DeSantis C, Robbins A (2014). Childhood and adolescent cancer statistics, 2014. CA Cancer J Clin.

[4] Stiller CA, Parkin DM (1996). Geographic and ethnic variations in the incidence of childhood cancer. Br Med Bull.

[5] Raney RB, Asmar L, Newton WA (1997). Ewing’s sarcoma of soft tissues in childhood: a report from the Intergroup Rhabdomyosarcoma Study, 1972 to 1991. J Clin Oncol.

[6] Grünewald TG, Bernard V, Gilardi-Hebenstreit P (2015). Chimeric EWSR1-FLI1 regulates the Ewing sarcoma susceptibility gene EGR2 via a GGAA microsatellite. Nat Genet.

[7] Delattre O, Zucman J, Melot T (1994). The Ewing family of tumors–a subgroup of small-round-cell tumors defined by specific chimeric transcripts. N Engl J Med.

[8] Chen X, Bahrami A, Pappo A (2014). Recurrent somatic structural variations contribute to tumorigenesis in pediatric osteosarcoma. Cell Rep.

[9] Perry JA, Kiezun A, Tonzi P (2014). Complementary genomic approaches highlight the PI3K/mTOR pathway as a common vulnerability in osteosarcoma. Proc Natl Acad Sci USA.

[10] Ognjanovic S, Olivier M, Bergemann TL (2012). Sarcomas in TP53 germline mutation carriers: a review of the IARC TP53 database. Cancer.

[11] Mirabello L, Yeager M, Mai PL (2015). Germline TP53 variants and susceptibility to osteosarcoma. J Natl Cancer Inst.

[12] McIntyre JF, Smith-Sorensen B, Friend SH (1994). Germline mutations of the p53 tumor suppressor gene in children with osteosarcoma. J Clin Oncol.

[13] Toguchida J, Yamaguchi T, Dayton SH (1992). Prevalence and spectrum of germline mutations of the p53 gene among patients with sarcoma. N Engl J Med.

[14] German J (1997). Bloom’s syndrome XX The first 100 cancers. Cancer Genet Cytogenet.

[15] Lipton JM, Federman N, Khabbaze Y (2001). Osteogenic sarcoma associated with Diamond-Blackfan anemia: a report from the Diamond-Blackfan Anemia Registry. J Pediatr Hematol Oncol.

[16] Li FP, Fraumeni JF, Mulvihill JJ (1988). A cancer family syndrome in twenty-four kindreds. Cancer Res.

[17] Grimer RJ, Cannon SR, Taminiau AM (2003). Osteosarcoma over the age of forty. Eur J Cancer.

[18] Wong FL, Boice JD, Abramson DH (1997). Cancer incidence after retinoblastoma Radiation dose and sarcoma risk. JAMA.

[19] Hicks MJ, Roth JR, Kozinetz CA (2007). Clinicopathologic features of osteosarcoma in patients with Rothmund-Thomson syndrome. J Clin Oncol.

[20] Goto M, Miller RW, Ishikawa Y (1996). Excess of rare cancers in Werner syndrome (adult progeria). Cancer Epidemiol Biomarkers Prev.

[21] Kager L, Zoubek A, Kastner U (2006). Skip metastases in osteosarcoma: experience of the Cooperative Osteosarcoma Study Group. J Clin Oncol.

[22] Campbell KM, Shulman DS, Grier HE (2021). Role of bone marrow biopsy for staging new patients with Ewing sarcoma: a systematic review. Pediatr Blood Cancer.

[23] Gyorke T, Zajic T, Lange A (2006). Impact of FDG PET for staging of Ewing sarcomas and primitive neuroectodermal tumours. Nucl Med Commun.

[24] White VA, Fanning CV, Ayala AG (1988). Osteosarcoma and the role of fine-needle aspiration: a study of 51 cases. Cancer.

[25] Ahrar K, Himmerich JU, Herzog CE (2004). Percutaneous ultrasound-guided biopsy in the definitive diagnosis of osteosarcoma. J Vasc Interv Radiol.

[26] Puri A, Shingade VU, Agarwal MG (2006). CT-guided percutaneous core needle biopsy in deep seated musculoskeletal lesions: a prospective study of 128 cases. Skeletal Radiol.

[27] Kilpatrick SE, Ward WG, Bos GD (2001). The role of fine needle aspiration biopsy in the diagnosis and management of osteosarcoma. Pediatr Pathol Mol Med.

[28] Dodd LG, Scully SP, Cothran RL (2002). Utility of fine-needle aspiration in the diagnosis of primary osteosarcoma. Diagn Cytopathol.

[29] Domanski HA, Akerman M (2005). Fine-needle aspiration of primary osteosarcoma: a cytological histological study. Diagn Cytopathol.

[30] Jelinek JS, Murphey MD, Welker JA (2002). Diagnosis of primary bone tumors with image-guided percutaneous biopsy: experience with 110 tumors. Radiology.

[31] Mitsuyoshi G, Naito N, Kawai A (2006). Accurate diagnosis of musculoskeletal lesions by core needle biopsy. J Surg Oncol.

[32] Carrino JA, Khurana B, Ready JE (2007). Magnetic resonance imaging-guided percutaneous biopsy of musculoskeletal lesions. J Bone Joint Surg Am.

[33] Bacci G, Longhi A, Cesari M (2006). Influence of local recurrence on survival in patients with extremity osteosarcoma treated with neoadjuvant chemotherapy: the experience of a single institution with 44 patients. Cancer.

[34] Rodriguez-Galindo C, Liu T, Krasin MJ (2007). Analysis of prognostic factors in Ewing sarcoma family of tumors: review of St. Jude Children’s Research Hospital studies. Cancer.

[35] Ahmed SK, Randall RL, DuBois SG (2017). Identification of patients with localized Ewing sarcoma at higher risk for local failure: a report from the Children’s Oncology Group. Int J Radiat Oncol Biol Phys.

[36] Wilkins RM, Pritchard DJ, Burgert EO (1986). Ewing’s sarcoma of bone. Experience with 140 patients. Cancer.

[37] Rosen G, Caparros B, Nirenberg A (1981). Ewing’s sarcoma: ten-year experience with adjuvant chemotherapy. Cancer.

[38] DuBois SG, Krailo MD, Gebhardt MC (2015). Comparative evaluation of local control strategies in localized Ewing sarcoma of bone: a report from the Children’s Oncology Group. Cancer.

[39] Werier J, Yao X, Caudrelier JM (2016). A systematic review of optimal treatment strategies for localized Ewing’s sarcoma of bone after neoadjuvant chemotherapy. Surg Oncol.

[40] Nesbit ME, Gehan EA (1990). Multimodal therapy for the management of primary, nonmetastatic Ewing’s sarcoma of bone: a long-term follow-up of the First Intergroup study. J Clin Oncol.

[41] Brennan B, Kirton L, Marec-Berard P Comparison of two chemotherapy regimens in Ewing Sarcoma: overall and subgroup results of the Euro Ewing 2012 randomized trial (EE2012). J Clin Oncol.

[42] Grier HE, Krailo MD, Tarbell NJ (2003). Addition of ifosfamide and etoposide to standard chemotherapy for Ewing’s sarcoma and primitive neuroectodermal tumor of bone. N Engl J Med.

[43] Womer RB, West DC, Krailo MD (2012). Randomized controlled trial of interval-compressed chemotherapy for the treatment of localized Ewing sarcoma: a report from the Children’s Oncology Group. J Clin Oncol.

[44] Goorin AM, Frei E III, Abelson HT (1981). Adjuvant chemotherapy for osteosarcoma: a decade of experience. Surg Clin North Am.

[45] Dirksen U, Le Deley MC, Brennan B (2016). Efficacy of busulfanmelphan high dose chemotherapy consolidation (BuMel) compared to conventional chemotherapy combined with lung irradiation: results of the EURO-EWING 99-R2pulm randomized trial (EE99R2pul). J Clin Oncol.

[46] Whelan J, Le Deley MC, Dirksen U (2018). High-dose chemotherapy and blood autologous stem-cell rescue compared with standard chemotherapy in localized high-risk Ewing sarcoma: results of Euro-E.W.I.N.G.99 and Ewing-2008. J Clin Oncol.

[47] Foulon S, Brennan B, Gaspar N (2016). Can postoperative radiotherapy be omitted in localised standard-risk Ewing sarcoma? An observational study of the Euro-E.W.I.N.G group. Eur J Cancer.

[48] Indelicato DJ, Keole SR, Shahlaee AH (2008). Long-term clinical and functional outcomes after treatment for localized Ewing’s tumor of the lower extremity. Int J Radiat Oncol Biol Phys.

[49] Puri A, Gulia A, Jambhekar NA (2012). Results of surgical resection in pelvic Ewing’s sarcoma. J Surg Oncol.

[50] Yock TI, Krailo M, Fryer CJ (2006). Local control in pelvic Ewing sarcoma: analysis from INT-0091—a report from the Children’s Oncology Group. J Clin Oncol.

[51] Aponte-Tinao LA, Ayerza MA, Albergo JI (2020). Do massive allograft reconstructions for tumors of the femur and tibia survive 10 or more years after implantation?. Clin Orthop Relat Res.

[52] Reddy KI, Wafa H, Gaston CL (2015). Does amputation offer any survival benefit over limb salvage in osteosarcoma patients with poor chemonecrosis and close margins. Bone Joint J.

[53] Jeys LM, Kulkarni A, Grimer RJ (2008). Endoprosthetic reconstruction for the treatment of musculoskeletal tumors of the appendicular skeleton and pelvis. J Bone Joint Surg Am.

[54] Biau D, Faure F, Katsahian S (2006). Survival of total knee replacement with a megaprosthesis after bone tumor resection. J Bone Joint Surg Am.

[55] Myers GJ, Abudu AT, Carter SR (2007). The long-term results of endoprosthetic replacement of the proximal tibia for bone tumours. J Bone Joint Surg Br.

[56] Myers GJ, Abudu AT, Carter SR (2007). Endoprosthetic replacement of the distal femur for bone tumours: long-term results. J Bone Joint Surg Br.

[57] Futani H, Minamizaki T, Nishimoto Y (2006). Long-term follow-up after limb salvage in skeletally immature children with a primary malignant tumor of the distal end of the femur. J Bone Joint Surg Am.

[58] Mankin HJ, Mankin CJ, Simon MA (1996). The hazards of the biopsy, revisited Members of the musculoskeletal tumor society. J Bone Joint Surg Am.

[59] Iemsawatdikul K, Gooding CA, Twomey EL (2005). Seeding of osteosarcoma in the biopsy tract of a patient with multifocal osteosarcoma. Pediatr Radiol.

[60] Bacci G, Rocca M, Salone M (2008). High grade osteosarcoma of the extremities with lung metastases at presentation: treatment with neoadjuvant chemotherapy and simultaneous resection of primary and metastatic lesions. J Surg Oncol.

# Discussion: Liver tumours: hepatoblastoma

**Published:** 2022

**Authors:** Rebecka Meyers, Reto Baertschiger, Greg Tiao, Eiso Hiyama, Daniel Aronson, Piotr Czauderna, Jim Wilde, Sophie Branchereau, Gloria Gonzalez, Bibekanand Jindal, Nitin James Peters

## Evaluation

### Epidemiology

Although rare, hepatoblastoma (HB) is the most common malignant childhood liver tumour. The incidence has doubled from about 0.1/100,000 in the 1980s to about 0.2/100,000 in 2008 and the percentage increase in incidence is more than that for almost any other childhood tumour [1]. Several inherited conditions, including familial adenomatous polyposis and congenital hemihypertrophies like Beckwith–Wiedemann syndrome, raise risk for HB but account for few cases overall. Case–control studies investigating risk factors for sporadic HB show there is a roughly 20-fold increased risk of HB among children with very low birth weight (<1,500 g) [2].

### Clinical presentation

Most children present with subtle symptoms of poor appetite and failure to thrive associated with large upper abdominal mass. Rare cases will present with abdominal pain or hypotension secondary to tumour rupture and bleeding.

### Workup

Lab: Alpha-fetoprotein (AFP), beta-human chorionic gonadotropin (beta-hCG), white blood count, haematocrit, platelet count, absolute neutrophil count, electrolytes, lactate, PT/INR, AST/ALT, fractionated bilirubin.

Imaging: Abdominal ultrasound to confirm liver as organ of origin, CT chest and CT/MRI abdomen with intravenous contrast. If using MRI, consider anaesthesia to avoid motion artefact and hepatocyte specific contrast which increases ability to detect multifocal nodules (Gadoxetate disodium/Eovist, Primovist or gadopentetate dimeglumine/Gadotex).

The ability to interpret cross-sectional imaging is essential (Figure 1). Most important is the determination of the number of *contiguous uninvolved* sections of liver to determine the PRETEXT group and the evaluation of the relationship between the tumour and surrounding structures and major inflow and outflow vasculature to determine PRETEXT annotation factors. Basic information for surgical planning includes the following:

1. Determination of PRETEXT (pretreatment extent of tumour) and POST-TEXT (post chemotherapy extent of tumour) (Figure 2) (Towbin *et al* [3]).
  - Group (I, II, III, IV)
  - Annotation Factors (V, P, E, F, R, C, N, M)
2. Relation of the tumour with surrounding organs (E) and vascular structures (V and P).
3. Evaluation of preoperative tumour rupture (R).
4. Detection of multifocal tumour nodules (F). MRI with hepatocyte specific contrast agents may identify multifocal tumour nodules not seen with other types of imaging.
5. Chest CT to evaluate for lung metastasis (M).
6. Serial AFP and radiographic imaging to monitor response to neoadjuvant chemotherapy (POST-TEXT).

## Indications and Principles of Biopsy Versus Resection at Diagnosis

A patient with suspected HB having a typical age, elevated AFP and imaging findings suggesting it is resectable at diagnosis, will *not* require biopsy for clinical diagnosis. The most common age of diagnosis for HB is 4 months to 4 years. Differential diagnosis in infants includes congenital and infantile hepatic haemangioma, rhabdoid tumour and very rarely germ cell tumour. Older children may have hepatocellular carcinoma (HCC). Cystic HB is possible, but very rare, and the more common cystic appearing liver tumours in children are mesenchymal hamartoma and undifferentiated embryonal sarcoma [4].

HB considered resectable at diagnosis on the Pediatric Hepatic International Tumour Trial (PHITT) are PRETEXT group I or II, negative annotation factors and at least 1 cm of uninvolved liver parenchyma separating the tumour from the middle hepatic vein, the retrohepatic vena cava and the remaining portal vein [5]. If resected at diagnosis and found to have well-differentiated foetal histology, no post-operative chemotherapy may be needed. Other histologies resected at diagnosis will require limited post-operative chemotherapy.

If not resectable, or if the diagnosis is in question, the technique of biopsy can be percutaneous, laparoscopic or open. In contemporary practice, most common is percutaneous core needle biopsy (PCNB). PCNB is done under ultrasound guidance avoiding vascular structures and sampling different areas of the tumour. Ideal PCNB approach is via a core needle tract passing through a buffer zone of overlying normal liver. Upon completion, the tract should be embolised if possible (e.g. gelfoam plug), and the trajectory should be planned for eventual resection as part of the surgical specimen. Participation in biologic studies of multicentre trials requires at least 7–13 cores of tumour (2–3 cores for diagnosis, 5–10 cores frozen for biologic studies), and at least one core of frozen adjacent normal liver for biologic determination of germ-line mutations. Proper tissue assessment for adequate viable tumour, and specimen freezing for biologic studies, requires the immediate presence, tissue analysis and handling by the pathologist.

## Role and Timing of Multimodality Therapy

Definitive treatment of HB involves a combination of surgery and chemotherapy. The two most powerful chemotherapy agents used are cisplatin and doxorubicin. Other agents variably used in the past by different trial groups have included: 5-fluorouracil, vincristine, irinotecan, temsirolimus, pirarubicin, carboplatin and etoposide. If not resected at diagnosis, chemotherapy is given until surgery can be performed on the primary tumour, as well as any remaining detectable extrahepatic disease, and then continued postoperatively. Potential chemotherapy side effects include fever, neutropenia, infections, cisplatin ototoxicity, renal toxicity, cardiomyopathy and secondary malignancies. Although different trial groups have historically defined their risk (treatment) categories in disparate ways, there is now international agreement. Pediatric Hepatic International Tumour Trial (PHITT) is an international collaborative trial studying paediatric HB and HCC which opened to enrollment in 2017 by managing centres in Europe/SIOPEL, North America/COG (AHEP1531) and Japan/JCCG. Children with HB are assigned to treatment on either the very low-risk, low-risk, intermediate-risk or high-risk treatment strata based upon the Children’s Hepatic Tumors International Collaboration Hepatoblastoma Risk-Stratification CHIC-HS [6] (Figure 3).

Details of the PHITT treatment protocols are available from the coordinating trial groups: SIOPEL, COG/AHEP1531 and JCCG. In abbreviated summary: a) Very-low-risk HB are those tumours at diagnosis and receive no postoperative chemotherapy for well-differentiated foetal histology, or two cycles of post-operative cisplatin for all other histologies; b) Low-risk HB are assessed for resectability after two cycles of neoadjuvant therapy and if resectable are randomised to two, or four, cycles of post-operative chemotherapy; c) Intermediate-risk HB are randomised to different chemotherapy regimens and receive four cycles pre-operative, and two cycles post-operative cisplatin monotherapy, or, cisplatin/5fu/vincristine/doxorubicin (C5VD). For intermediate-risk HB enrolled in Europe/SIOPEL (*not* COG, JCCG), randomisation is possible to a third possible chemotherapy regimen based upon SIOPEL 3HR; d) High-risk tumours receive preoperative chemotherapy based upon the prior SIOPEL 4 study with dose compressed weekly cisplatin and q-3-weekly doxorubicin [7] and a post-operative chemotherapy regimen that is dependent upon the response to the induction therapy.

## Surgical Management

**Preoperative and Perioperative Management**

**Lab:** AFP, WBC, haematocrit, platelet count, absolute neutrophil count, electrolytes, lactate, PT/INR, AST/ALT, fractionated bilirubin.

**Anaesthetic Management**: Critical coordination between surgeon and anaesthetic team is mandatory during times of patient compromise. Preparation should include a discussion of the following:

- *Central venous catheter* for monitoring and fluid resuscitation. Two large bore peripheral catheters. Groin or lower extremity lines should be avoided due to potential need for intra-operative clamping of the IVC.
- *Upper extremity arterial line* is highly recommended for continuous arterial pressure monitoring. Pre-incision *antibiotics*, first-generation cephalosporin, <60 minutes before incision.
- *Blood Products* should include *c*ross-matched, immunosuppressed patient compatible, packed red blood cells (20 mL/kg), fresh frozen plasma (20 mL/kg), platelets (10 mL/kg) available. Blood loss is typically higher than seen in most other operations performed by paediatric surgeons and the entire surgical team must be prepared.
- *Hypovolaemic resuscitation.* The principle of hypovolaemic resuscitation during the parenchymal phase of resection, followed by post-resection, intraoperative volume resuscitation is of considerable value and surgeons should discuss this strategy with the anaesthesiologist preoperatively. Limiting volume resuscitation during the parenchymal transection phase, by maintaining relatively low central venous pressure, is important in reducing blood loss. Hypotension is a routine part of the operation, particularly during partial compression of the vena cava, and may require administration of vasopressor support. Aggressive volume-loading causes hepatic congestion that increases blood loss from exposed hepatic veins. Urine output during the hypovolaemic resection is often low and will resume with post-resection volume resuscitation.
- *Air embolisation* can occur during uncontrolled bleeding from IVC or large hepatic veins and is a potential cause, along with concomitant hypovolaemia, of intraoperative cardiac arrest. End tidal CO2 will be lost and the anaesthesiologist must have a treatment plan in place. This includes placement in Trendelenberg position, with the table rolled so the patient right side is up, along with effective CPR. Otherwise central venous catheter may be rapidly pushed into the right atrium and residual air can be sucked out. These measures are often rewarded by rapid return of stability as long as source control for the air and blood loss is achieved.
- *Hypothermia* can be more of a problem in children than in adults. Active measures for warming children include warmed forced air under the patient and keeping the operating room temperature higher than may be comfortable for the surgical team.
- *Post-operative pain control* can be planned in conjunction with the anaesthesia and intensive care unit teams.

**Surgical planning checklist**: Few types of surgery are less forgiving of poor preparation and decision making than the surgical resection of a large liver tumour in a small child. The following checklist is designed to organise the data which informs a safe and successful surgical resection.

- Does the proposed liver remnant have an unanticipated focus of tumour? Will the liver remnant be of adequate health and size, 1% of body weight? Fibrosis, cirrhosis, fatty or other underlying liver disease will increase the risk of postoperative hepatic insufficiency and may represent a contraindication to an anticipated small for size liver remnant.
- Is there extensive multifocal disease? This may be a contraindication for conventional resection unless liver transplant options have been considered in detail and the team has specifically rejected the option of transplantation based upon: a) not available, b) uncontrolled extrahepatic tumour and/or c) limited, chemosensitive multifocal sites which the surgeon feels are amenable to resection with low risk of local relapse.
- Is an adequate margin of resection in doubt? Is intraoperative ultrasound available if needed to help make this determination?
- What is the status of the vascular inflow and outflow to the proposed liver remnant? Assessment of the vascular anatomy should include an estimation of ischaemic time, if any, needed for vascular reconstruction. Can this be done safely?
- Intravascular tumour thrombus. Any evidence of tumour thrombus on preoperative imaging should have prompted a discussion of possible liver transplant. Tumour thrombectomy of viable tumour will increase the risk of relapse. If the thrombus is NOT viable and has responded to preoperative chemotherapy, resection of the portion of vein with adherent tumour thrombus and reconstruction with autologous jugular vein, or equivalent, is possible but is an advanced technique that will require increased ischaemic time and should not be undertaken casually.
- Is the entire operative team prepared for the resection required by the operative findings? Does the institution have all resources necessary to care for potential operative complications if required?

## Key Steps of the Surgical Procedure: Hepatectomy

**Incision**: Incision is either a unilateral subcostal with an epigastric extension or a chevron bilateral subcostal with an epigastric extension. In cases of extensive diaphragm involvement or suprahepatic vena cava thrombus, a thoracoabdominal approach or median sternotomy extension can be considered.

**Liver mobilisation**: The liver should be methodically mobilised by dividing ligamentous attachments of the left lateral and right posterior sections to the diaphragm.

**Suprahepatic vena cava dissection**: The liver is placed on downward traction and thick investing fascia is carefully teased away from the suprahepatic cava. Left and middle hepatic vein often share a common trunk. As the dissection is carried down the right lateral aspect of the suprahepatic vena cava, the right hepatic vein is cleaned and identified. On both sides, phrenic veins are at risk and should be identified and protected or suture ligated.

**Retrohepatic vena cava dissection**: Resection of tumours involving Couinaud segments 1, 6, 7 and 8 will benefit from a meticulous dissection of the retrohepatic vena cava to free the posterior aspects of the liver and caudate lobe. Ties or clips on the vena cava should be placed with care as increases in the central venous pressure can push off an imperfect knot or loose clip resulting in significant blood loss. This is particularly true of the major hepatic veins which are optimally oversewn or stapled. Proximal and distal control of the supra- and infra-hepatic vena cava can be achieved either by preplacement of umbilical tape/vessel loops, or exposure and test clamping with vascular clamps which are immediately available on the field.

**Porta hepatis dissection**: Cholecystectomy is performed unless the gallbladder is to be removed en-bloc with the tumour. Porta hepatis lymph nodes should be biopsied and sent to pathology. Hepatic artery, portal vein and biliary drainage to the planned liver remnant are identified and preserved. Hepatic artery, portal vein and bile ducts to the liver involved by tumour are identified for planned ligation. Cholangiogram may be necessary to define atypical biliary anatomy. All vasculature to the planned liver remnant must be carefully preserved; do not ligate anything until the anatomy is certain and clear. If viable portal tumour thrombus is encountered extending anywhere near the portal bifurcation, the surgeon should seriously consider whether a total hepatectomy with liver transplant might yield a resection with less risk of tumour relapse. Exposure and plans for a Pringle manoeuvre, should it become necessary, should be made.

**Hepatic veins**: Most commonly divided prior to the onset of the parenchymal dissection, in select cases the hepatic veins may not be divided until improved access can be obtained as part of the parenchymal dissection. Ultrasound and/or CUSA are sometimes used to identify and skeletonise the veins to see them clearly before ligation. Inadvertent uncontrolled tears in the hepatic veins put the patient at risk for major bleeding and air embolus. Maintaining Positive End-Expiratory Pressure (PEEP) at this phase of dissection by anaesthesiologist is helpful to prevent air embolism.

**Parenchymal transection**: Once the hepatic arterial and portal inflow has been divided, the ipsilateral liver becomes dark. This color demarcation may be subtle and is best seen if the liver is not manipulated during the test clamp. Parenchymal transection has historically been accomplished with finger fracture, crush and clamp, clips and suture ligature, however one of a variety of auxiliary devices is now preferred by various surgical teams including harmonic scalpel, cavitron ultrasonic surgical aspirator (CUSA), waterjet knife, Aquamantis and/or GIA stapler or vessel sealing devices. Independent of device or technique, the surgeon should be alert for large crossing vessels and vigilance maintained in order not to wander off the plane of dissection that could potentially damage vital structures or bile ducts to segments of liver that are intended to be preserved. Where tumour boundaries are uncertain, intraoperative ultrasound is useful. Focused situational awareness, and accurate definition of anatomy, will prevent complications. Any sudden decrease in venous return accompanying an uncontrolled hepatic vein opening can result in bleeding, hypotension and possible air embolism. Manual compression of the liver can emergently reduce the bleeding and provide some restoration of venous return allowing the anaesthesia team to catch up. Definitive control may sometimes require vascular isolation with clamping of supra-and infra-hepatic vena cava and portal triad (Pringle). Multiple short-duration (10–15 minutes), intermittent clamping is better tolerated than prolonged clamping (greater than 30–45 minutes). Patients are not usually anticoagulated when undergoing short periods of vascular isolation. Loss of control of a major hepatic vein is the most common disastrous intraoperative complication by inexperienced surgeons.

**Pathology assessment, haemostasis and closure**: Gross surgical margins should *always* be negative. When there is any question, the surgeon should request an intraoperative pathologic assessment with inking and cutting of the specimen with both surgeon and pathologist assessing the gross margin. In any area where the margin is in doubt, if anatomically feasible, the surgeon should resect and submit addition margin for histologic analysis. The cut surface of the liver is inspected for bleeding and bile leaks – all of which must be oversewn. Haemostatic agents such as Surgicel™, Hemospray™, Tachosil™, Tisseal™, Eviseal™ and others may be used on the raw surface at the surgeons’ discretion but should not be depended upon to stop active bleeding. If the ligamentous support to the remaining liver has been divided, it is advisable to suture the remaining liver to the remnant falciform ligament to prevent postoperative rotation or kink causing obstruction to venous outflow. A surgical drain is not mandatory, but in many cases is helpful to identify and control a bile leak. If placed, the drain is generally removed once a diet is resumed. The abdominal wall is closed in anatomic layers with running, absorbable, monofilament suture.

## Types of Liver Resections

Types of liver resection include: non-anatomic wedge, single sectionectomy, hemihepatectomy, extended hemihepatectomy, complete trisectionectomy and complete hepatectomy/orthotopic liver transplant. This terminology of resection is based on the Brisbane consensus from the Committee of the International Hepato-Pancreato-Biliary Association from 2000. Laparoscopic hepatic resection is increasingly done in adults, although experience in children is limited with the possible exception of wedge resection for small focal tumours.

- Non-anatomic resection. Data from the German HB 89 and 94 studies suggested that non-anatomic resections may have inferior outcomes [8]. However, sometimes a tumour will be pedunculated and exophytic or ‘hanging’ inferiorly from either Couinaud segments 5/6 or segment 3; in these cases, some surgeons prefer a non-anatomic resection by parenchymal transection of a rim of uninvolved parenchyma at a distance from both the tumour and from the segmental inflow [9].
- Left lateral sectionectomy (Couinaud segments 2 and 3). The ease of this operation should not lead the surgeon to skip important steps. In order to avoid injury to the left portal vein, hepatic arteries or bile duct branches to segment 4, the individual bundles to segments 2 and 3 are best taken individually. If a replaced left hepatic artery exists, it will need to be identified and divided. If the middle and left hepatic veins have a common trunk, tumour margin permitting, the left hepatic vein may need to be divided within the parenchyma.
- Left hemihepatectomy (Couinaud segments 2, 3, 4, with or without 1/caudate). Complete mobilisation of the right lobe is not always necessary but can be helpful. Mobilisation of the left triangular ligament is required and provides exposure, with some additional dissection along the right side of the cava, to permit vascular control of the liver. Due to the variable anatomy of the middle vein, there is some ambiguity in terminology of resections. ‘Extended’ left hepatectomies are those that include resection of the middle vein and a portion of segments 5 or 8. The large size of the right lobe as a liver remnant allows resection of the middle vein with little consequence if needed for a margin; if not needed for a margin leaving the middle vein as the border of the resection is preferred. Resection of segment 1 requires ligation of multiple small hepatic veins draining directly to the vena cava which potentially places segment 6 and 7 veins at risk.
- Right hemihepatectomy (Couinaud segments 5, 6, 7, 8). The right adrenal gland and vein are preserved when possible. Retrohepatic vena cava dissection will include division of short hepatic veins from segments 6 and 7. During the parenchymal transection phase, it is important to be constantly aware of the position of the tumour and the plane of dissection to prevent travelling to the left and injuring the middle hepatic vein superiorly and/or bile ducts to the left liver inferiorly. ‘Extended’ right hemihepatectomy would include resection of the middle hepatic vein and portions of segments 4a or 4b.
- Trisectionectomy. Left (Couinaud segments 1, 2, 3, 4, 5, 8) or right trisectionectomy (Couinaud segments 4, 5, 6, 7, 8). The challenge of these extensive resections is the potential for a compromised or small-for-size liver remnant. Trisectionectomies are typically done for very large tumours; extensive multifocal tumours are sometimes better managed by complete hepatectomy/liver transplant. The liver remnant after a left *trisectionectomy* is the right posterior section (segments 6 and 7) and ideally the right lobe should not be extensively mobilised off of the vena cava, because important auxiliary venous drainage of segments 6 and 7 should be preserved. In left trisectionectomy, the parenchymal transection will be on a sagittal plane just above the right hepatic vein. Caudate short hepatic veins should be approached first inferiorly and then from the left, although leaving a portion, or all of segment 1 in place is preferred if margins allow this luxury. The gallbladder is often taken *en-bloc* with the specimen. The liver remnant after a *right trisectionrectomy* is the left lateral sector, segments 2 and 3. The preserved portal bundle is the left portal bundle minus the medial branches to segments 4a and 4b. The venous outflow is the left hepatic vein. The right lobe and tumour are extensively mobilised off the vena cava, with or without segment 1.
- Mesohepatectomy or central liver resection (Couinaud segments 4, 5, 8). A central resection is more complex and requires the surgeon to perform dissection and preservation of major vasculature to both left and right and thus put the entire liver at some risk. Short periods of hepatic exclusion can be helpful during the parenchymal transection phase to decrease risk of bleeding. Mesohepatectomy, when feasible, will leave the child with significantly more residual liver parenchyma and thus the physiologic impact may be less than a trisectionectomy [10, 11]. Margins of resection on the left are the Rex fissure, then vertically to the junction of the left and middle hepatic veins. On the right, the line of resection extends from near the infundibulum of the gallbladder, laterally to just anterior to the right hepatic vein. In the hilum of the liver, the resection line is nearly horizontal and just above the branching portal veins, arteries and bile ducts to segments 2, 3, and 6, 7.
- Caudate lobe resection (Couinaud segment 1). Isolated caudate resection is rare. The surgeon will need to mobilise both the left and right hemi-livers. It is tempting to take the short hepatic vein branches to the caudate during this mobilisation but this is a mistake as swelling from premature interruption of venous outflow will increase risk of rupture. Posterior portal and arterial branches to caudate must be taken while carefully preserving those to the anterior segments. Working alternately from the right and the left sides is often helpful. *After* the inflow has been controlled, ligation of short caudate hepatic veins frees the cava. Parenchymal transection is inferior to superior taking care not to stray into the right posterior section. The superior point of resection is a narrow wedge between the cava and the middle hepatic vein.
- Total hepatectomy/orthotopic liver transplant. Detailed discussion of surgical technique of liver transplant is beyond the scope of these guidelines. Conventional resection is usually feasible, in experienced hands, if at least one portal and one ipsilateral hepatic vein can be salvaged. Indications for total hepatectomy/liver transplant include a post-chemotherapy assessment that shows: a) extensive multifocal tumours with macroscopic, or suspected microscopic, involvement of all four sections; b) unresectable tumour involvement, or viable tumour thrombus, of main portal vein, both right and left portal veins, and/or all three hepatic veins. Tumour involvement of the vena cava can sometimes be resected and reconstructed. Transplant is not recommended in the setting of lung metastasis which do not resolve with chemotherapy and/or are not surgically resectable.

## Post-operative Management

Patients are admitted to intensive care unit for continuous hemodynamic monitoring and ongoing resuscitation to restore core temperature (>36.5°C), restore urine output (> 1 mL/kg/hour), blood volume (haematocrit > 22, platelet > 50,000, international normalisation ratio (INR <1.8). Laboratory interrogation for hepatic insufficiency will include serial measurement of lactate, glucose, PT/INR, fibrinogen and acid base balance. Over-resuscitation is avoided and a central venous pressure (CVP) < 8 is preferred provided other indicators of perfusion are adequate. Patients are weaned off mechanical ventilation and extubated, mobilised and pain managed as needed. Perioperative antibiotics are generally discontinued after 24 hours. Diet is introduced once ileus has resolved. Wound drain (if present) is monitored for blood or bile output. As liver regeneration progresses, supplemental magnesium, phosphorus or potassium may be needed.

## Pitfalls, and Potential Surgical Complications

Potential challenges and complications include haemorrhage, hepatic insufficiency, ascites and portal hypertension, renal dysfunction, bile leak, delayed gastric emptying, ileus and pleural effusion.

- ***Haemorrhage***. Occult haemorrhage should be suspected if the response to blood transfusion is inappropriate, and the patient does not improve with resuscitation. When a drain is clotted or loculated, it may not be a reliable indicator of bleeding. Abdominal ultrasound may be helpful to identify bleeding, although when in doubt the surgeon should maintain a low threshold for operative re-exploration and accept the possibility of a negative exploration rather than delay the control of significant bleeding.
- ***Hepatic dysfunction***. Liver failure is most often a result of hepatic ischaemia from damaged inflow, or congestion from damaged outflow. A small for size remnant is also possible. The degree of hepatic dysfunction can be monitored by serial lactate, glucose, PT/INR and fibrinogen levels. Hypoglycaemia is monitored and treated with 10% dextrose, elevated PT/INR is treated with FFP and sometimes exogenous vitamin K, fibrinogen level < 100 mg/dL is treated with cryoprecipitate. Transient elevation of AST/ALT and bilirubin levels is common; however, more prolonged elevation can signal hepatic insufficiency. If the acute vascular insufficiency is severe, dysfunction of the remnant liver can lead to encephalopathy, coagulopathy, hypoglycaemia and death. Prevention is key since once this has happened, attempted repairs involve further ischaemia reperfusion injury and often do not provide durable improvement. Emergent rescue liver transplants have been done but survival is not always possible.
- ***Hepatic congestion from obstructed venous outflow***. Obstruction of the hepatic veins will create a Budd–Chiari physiology with venous congestion in the intestines, ascites and liver dysfunction (see above). Venous obstruction may cause swelling of the liver remnant and venous hypertension will increase the risk of bleeding from the cut surface. Remember to make sure there is no obstructing kink or twist in the remnant hepatic vein which may need operative suspension and fixation. Again, prevention is key and if there is any question about the ability to achieve a definitive tumour resection, without compromising venous drainage, transplant should be considered.
- ***Portal hypertension***. Immediate postoperative venous congestion of the bowel may result from thrombus or encroachment on the remaining portal vein from a misplaced ligature or kinking/twisting of an excessively mobile remnant. If the postoperative ultrasound suggests a technical error, it should be corrected. Late-onset portal hypertension may rarely result from biliary cirrhosis or ischaemic fibrosis.
- ***Bile leak***. Most bile leaks are self-limiting and aggravated by swelling, and partial obstruction of the distal biliary tree which promotes leakage from the cut surface. If minor, the leak is treated with temporary controlled drainage. Persistent leaks may be the result of more severe distal obstruction and may require ERCP or transhepatic biliary stenting, and/or surgical drainage.
- ***Renal failure***. Low urine output is common in the early postoperative phase pending warming and definitive resuscitation. Prolonged post-operative renal dysfunction is rare and if it occurs after a vena cava reconstruction, it should prompt investigation of the reconstruction.
- ***Prolonged ileus and delayed gastric emptying***. Prolonged ileus may result from injury to the remnant portal causing venous congestion of the bowel. Gastroparesis may occasionally complicate an extensive dissection in the region of the gastric lesser curve.
- ***Pleural effusion or pneumothorax***. Tube thoracostomy may be needed after right-sided resections with involvement of the right hemidiaphragm. Persistent pleural drainage may suggest a sub-diaphragmatic bile collection, haematoma or alternately an injury to the thoracic duct at the caval hiatus.

## Other Surgical Considerations

**Transarterial chemo- or radio-embolisation:** TACE or TARE is occasionally used to increase resectability in children who are not resectable and are not liver transplant candidates due to uncontrolled metastatic disease [12, 13]. It has also been used to maintain tumour control for patients who have completed protocol systemic chemotherapy but for whom a donor organ for a needed transplant is not yet available.

**ALPPS (Association liver partition and portal vein ligation):** In the scenario of potential insufficient liver remnant size (less than 1% body weight), preresection hypertrophy of the remnant liver can be induced by percutaneous embolisation of the portal vein inflow to the tumour side of the liver, or ALPPS may be performed as the first stage of a two-stage procedure.

**Three-dimensional computer enhanced imaging:** Concern for safety of vascular tumour margins, added to the complexity of the liver vasculature, motivated the development of a patient-specific, computer-assisted planning platform by the Fraunhofer MEVIS company in Germany. This company has developed software which analyses CT and/or MRI radiological images provided by the treating institution and calculates information on the drainage and perfusion of the organ using mathematical models. The algorithms quantify risks for the intervention and generate a detailed 3D visualisation of the liver and its vascular systems. Supply areas of these blood vessels, such as the portal vein and hepatic arteries, are calculated and help to evaluate and optimise the surgical planning. The utility of this surgical planning tool is being investigated as an adjunct to the PHITT study (personal communication Steven.Warman@med.uni-tuebingen.de).

**Management of lung metastases:** Pulmonary metastectomy can be an effective strategy for lung lesions which fail to resolve on chemotherapy [14, 15]. The role of metastasectomy for relapse is less definitive but the bulk of evidence supports surgical resection as a safe and, in the context of multimodal therapy, efficacious approach to manage pulmonary relapse [16]. Recently, preoperative intravenous indocyanine green (ICG) has been used to localise occult nodules at the time of metastasectomy and may enhance our ability to clear the lungs of metastatic disease.

**Indocyanine green (ICG) navigation surgery**: With ICG navigation, tumour nodules otherwise not visible may be seen by green fluorescence at the time of surgery. For lung nodule surgery in HB, ICG (0.5 mg/kg) is injected 24 hours before pulmonary metastasectomy [17]. The sensitivity for viable tumour cells is 95%, but the specificity is only about 80% due to the false-positive fluorescence of inflammatory or ischaemiccells. ICG has also been used to detect multifocal nodules in liver but for this purpose it must be given at a higher dose 3 days before surgery because ICG is secreted in the bile and requires time to clear the normal liver. A limitation of ICG navigation, in both the lungs and the liver, is the inability to detect nodules deep in the parenchyma (deeper than 10–15 mm).

Microscopic positive surgical margin. For HB patients who have had a good chemotherapy response, repeat surgery may not be required in the setting of a positive microscopic surgical margin. The results of the SIOPEL studies reviewed by Aronson *et al* [23] showed no statistically significant worse outcome in patients with positive microscopic margins. However, the Japanese JPLT 2 study showed that microscopic positive margin negatively affected EFS because of a significantly higher rate of intrahepatic recurrence in that group [22, 23].

## Outcome

Hepatic regeneration is remarkably fast and most patients will have a normal liver volume within several months of liver resection. Successful surgical resection rates have increased over time and complete resection remains the cornerstone of curative therapy. The most recent published trial results for three of the major multicentre trial groups involved in the study of HB are shown in Table 1. *Cross group comparison of results in Table 1 is challenging because all of these studies pre-date the new common global CHIC-HS risk stratification system used in the current PHITT trial. Treatment/risk categories listed in Table 1 are different for each trial group.* The most contemporary results for SIOPEL are SIOPEL 4 and 6. SIOPEL 6 was able to reduce ototoxicity and maintain good outcomes in standard-risk tumours using six cycles of cisplatin monotherapy randomised with/ without the otoprotectant sodium thiosulfate (STS) [18]. SIOPEL 4 used a neoadjuvant induction of weekly, dose-compressed cisplatin and 3-weekly doxorubicin in high risk (either PRETEXT IV or metastatic) with EFS/OS of 76%/83%. Although this was not a randomised study and included a limited number of metastatic patients, these are the best results to date for patients presenting with metastatic disease [7].

Results for COG AHEP-0731, which enrolled 225 eligible patients from 2009–2018, by treatment strata were as follows: a) Very low risk and low risk, PRETEXT I and II tumours resectable at diagnosis, maintained excellent outcomes with reductions in chemotherapy [19]. b) Intermediate risk showed improved survival and surgical resection rates, compared to historic controls, by adding doxorubicin to their historic regimen and encouraging early involvement of liver specialty surgical centres [20] and c) High risk, metastatic patients were randomised to upfront experimental window chemotherapy of either vincristine-irinotecan (VI) [21] or vincristine-irinotecan-temsirolimus (VIT). There was response to the upfront experimental therapy, but this response was not superior to the C5VD backbone. The Japanese JPLT 2 study, which enrolled 361 patients from 1999 to 2012, showed that patients ‘ruptured at diagnosis’ may not do as well if the tumours are resected prior to chemotherapy. This Japanese study achieved outstanding results for CITA responders and did not support intensified chemotherapy, nor stem cell transplant, for CITA non-responders [22].

## Figures and Tables

**Figure 1.** Brisbane liver terminology. Hemiliver>Liver section>Couinaud segment.

**Figure 2.** Schematic representation of the PRETEXT system.

**Figure 3.** Children’s Hepatic Tumors International Collaboration Hepatoblastoma Risk-Stratification (CHIC-HS). Colour highlights of groups within each tree indicate which prognostic factor determined patient assignment to the ultimate group assignment: very low-, low-, intermediate- or high-risk group.

**Table 1.** Recent multicentre trial results.

Study	Risk group: Chemotherapy	Patients and PRETEXT	Outcomes
**SIOPEL 4** 2005–2009 [7]	HR: Block A: Weekly CIS + 3 weekly DOXO; Block B CARBO/DOX	*n = 62* PRETEXT: I = 2; II = 17; III = 27; IV = 16; Mets: 39	3-Year EFS/OS HR all: 76%/83% PRETEXTIV = 75%/88% Mets: 77%/79%
**SIOPEL 6** 2007–2014 [18]	SR: CIS versus CIS+STS	*n = 109*; CIS PRETEXT: I/II = 31; III = 21 CIS+STS PRETEXT: I/II = 41; III = 16	3-Year EFS/OS CIS: 79%/92% CIS+STS: 82%/98%
**AHEP-0731** 2009–2012 [17–19]	Very low risk: none Low risk: C5V 2 postop Int Risk (SCU or COG Stage III) C5VD Metastatic: COG Stage 4 VI or VIT Window^a^ followed by C5VD	*n = 225* Very low risk/PRETEXT I/II = 8 Low Risk PRETEXT I/II = 47; III =2; Int Risk PRETEXT: I/II = 34; III = 54; IV = 14;MetsVI: 30 Mets/VIT^a^: 36 (to be published)	5-Year EFS/OS Very low risk: 100%/100% Low risk: 91%/97% IntRisk: 87%/95% MetsVI: 49%/62%
**JPLT 2** 1999–2012 [20]	1: low-dose CITA postop only 2: low-dose CITA 3: CITA full dose 4: high dose +/−SCT	*n = 361*; Course 1 PRETEXT I/II rxn@ dx; Course 2 PRETEXT I/II preopchemo; Course 3 PRETEXT III/IV; Course 4 metastatic or CITA nonresponder	5-Year EFS/OS 1: 74%/90% 2: 85%/91% 3: 77%/87% 4: 37%/53%

a VIT window enrolled 2013–2016, not yet published, open writings of HCC and FLHCC trials

AFP, Alpha-fetoprotein; CARBO, Carboplatin; CIS, Cisplatin; EFS, Event-free survival; C5V, Cisplatin + 5-flurouracil (5FU) + vincristine; C5VD, Cisplatin +5-fluorouracil (5FU) + vincristine + doxorubicin; CITA, Cisplatin+ pirarubicin; DOXO, doxorubicin; HR, High risk; IPA, Ifosfamide + cis + adriamycin; OS, Overall survival; PFH, Pure foetal histology; SCT, Stem cell transplant; SCU, Small cell undifferentiated; SR, Standard risk; STS, Sodium thiosulfate otoprotectant; VP16, etoposide

## References

[1] Hubbard AK, Spector LG, Fortuna G (2019). Trends in the international incidence of pediatric cancers in children under 5 years of age: 1988-2012. JNCI Cancer Spectr.

[2] Spector LG, Birch J (2012). The epidemiology of hepatoblastoma. Pediatr Blood Cancer.

[3] Towbin AJ, Meyers RL, Woodley H (2018). PRETEXT 2017: radiologic staging system for primary hepatic malignancies of childhood revised for the Paediatric Hepatic International Tumour Trial (PHITT). Pediatr Radiol.

[4] Aronson DC, Meyers RL (2016). Malignant tumors of the liver in children. Sem Pedatr Surg.

[5] Meyers RL, Hiyama E, Czauderna P (2021). Liver tumors in pediatric patients. Surg Oncol Clin N Am.

[6] Meyers RL, Maibach R, Hiyama E (2017). Risk stratified staging in paediatric hepatoblastoma: a unified analysis from the Children’s Hepatic tumor International Collaboration (CHIC). Lancet Oncol.

[7] Zsiros J, Brugieres L, Brock P (2013). Dose-dense cisplatin-based chemotherapy and surgery for children with high risk hepatoblastoma (SIOPEL 4): a prospective, single-arm, feasibility study. Lancet Oncol.

[8] Fuchs J, Rydzynski J, Hecker H (2002). The influence of preoperative chemotherapy and surgical technique in the treatment of hepatoblastoma: a report from the German Cooperative Liver Tumour Studies HB 89 and HB 94. Eur J Pediatr Surg.

[9] Qureshi SS, Kembhavi SA, Kazi M (2020). Feasibility of nonanatomical liver resection in diligently selected patients with hepatoblastoma and comparison of outcomes with anatomic resection. Eur J Pediatr Surg.

[10] Amesty MV, Chocarro G, Sanchez AV (2016). Mesohepatectomy for centrally located tumors in children. Eur J Pediatr Surg.

[11] Guerin F, Gauthier F, Martelli H (2010). Outcome of central hepatectomy for hepatoblastoma. J Pediatr Surg.

[12] Lungren MP, Towbin AJ, Roebuck DJ (2018). Role of interventional radiology in managing pediatric liver tumors part two: percutaneous interventions. Pediatr Radiol.

[13] Aguado A, Dunn SP, Averill LW (2020). Successful use of transarterial radioembolization with yttrium-90 (TARE-Y90) in two children with hepatoblastoma. Pediatr Blood Cancer.

[14] O’Neill AF, Towbin AJ, Krailo MD (2017). Characterization of pulmonary metastases in children with hepatoblastoma treated on Children’s Oncology Group protocol AHEP 0731 (The ttreatment of children with all stages of hepatoblastoma): a report from the Children’s Oncology Group. J Clin Oncol.

[15] Hishiki T, Watanabe K, Ida K (2017). The role of pulmonary metastasectomy for hepatoblastoma in children with metastasis at diagnosis: Results from the JPLT-2 study. J Pediatr Surg.

[16] Shi Y, Geller JI, Ma IT (2015). Relapsed hepatoblastoma confined to the lung is effectively treated with pulmonary metastasecotmy. J Pediatr Surg.

[17] Yamada Y, Ohno M, Fujino A (2019). Fluorescence-guided surgery for hepatoblastoma with indocyanine green. Cancers (Basel).

[18] Brock PR, Maibach R, Childs M (2018). Sodium thiosulfate for protection from cisplatin induced hearing loss. N Engl J Med.

[19] Katzenstein HM, Langham MR, Malogolowkin MH (2019). Minimal adjuvant chemotherapy for children with hepatoblastoma resected at diagnosis (AHEP0731): a Children’s Oncology Group, multicentre, phase 3 trial. Lancet Oncol.

[20] Meyers RL, Malogolowkin MH, Krailo M (2017). Doxorubicin in combination with cisplatin/5-flourouracil/vincristine is effective in unresectable hepatoblastoma: a report from the Children’s Oncology Group (COG) AHEP0731 study committee. Presented High Impact Clinical Trials Session.

[21] Katzenstein HM, Furman WL, Malogolowkin MH (2017). Upfront window vincristine/irinotecan treatment of high risk hepatoblastoma: a report from the children’s oncology group AHEP 0731 study committee. Cancer.

[22] Hiyama E, Hishiki T, Watanabe K (2020). Outcome and late complications of hepatoblastomas treated using the Japanese Study Group for Pediatric Liver Tumor 2 Protocol. J Clin Oncol.

[23] Aronson DC, Weeda VB, Maibach R (2019). Microscopicallly positive resection margin after hepatoblastoma resection: what is the impact on prognosis? A Childhood Liver Tumors Strategy Group (SIOPEL) report. Eur J Cancer.

# Discussion: Liver tumours: paediatric hepatocellular carcinoma

**Published:** 2022

**Authors:** Eiso Hiyama, Rebecka Meyers, Reto Baertschiger, Greg Tiao, Daniel Aronson, Piotr Czauderna, Jim Wilde, Sophie Branchereau, Gloria Gonzalez, Bibekanand Jindal, Nitin Peters

## Evaluation

### Epidemiology

Hepatocellular carcinoma (HCC) is the second most common malignant childhood liver tumour and has an incidence of 0.7/1,000,000 per year, following hepatoblastoma (HB) [1]. HCC is typically diagnosed in older children and adolescents, accounting for more than 80% of primary hepatic tumours between 15 and 19 years of age [1]. More than 80% of paediatric HCC at diagnosis are unresectable due to large, often multifocal lesions and the high incidence of metastasis. Surveillance, Epidemiology and End Results database shows that only 0.4% of HCC occurs in paediatric patients and the incidence of HCC is significantly higher in countries with endemic hepatitis B infection, such as in Eastern and South East Asia and in Africa, for both paediatric and adult population [2].

Unlike adult HCC which occurs mainly in the cirrhotic liver, the majority of paediatric HCC occurs in the normal liver, and some paediatric HCC occurs in patients with inherited metabolic diseases such as familial cholestatic syndromes (progressive familial intrahepatic cholestasis and Alagille’s syndromes), extrahepatic biliary atresia, total parenteral nutrition and in association with tyrosinaemia, glycogenosis, neurofibromatosis, ataxia-telangiectasia, Fanconi’s anaemia and other constitutional and genetic abnormalities [2–4]. HCC in children and adolescents and young adults (AYA) may occur in the following different biological patterns: (I) conventional HCC; (II) transitional type of tumour with features of both HCC and HB defined as ‘Hepatocellular Neoplasm not otherwise specified’ (HCN-NOS) and often described as HB with HCC features [4] and (III) fibrolamellar HCC (FL-HCC) [5–7]. The term ‘fibrolamellar’ is derived from the presence of thick fibrous collagen bands surrounding the tumour cells. It usually has no underlying liver disease or cirrhosis and higher incidence of lymph node involvement than conventional HCC patients. A recent publication on the genomic heterogeneity of paediatric HCC did note a molecularly distinct pattern of 15 sequenced tumours [8]. Moreover, there is clinical heterogeneity of HCC comparing younger children to AYA, thus far not well described.

Both genetic and anatomic predisposition to HCC are seen in paediatric patients, especially younger children [3, 9, 10]. While previous data suggested that 30%–50% of paediatric HCC is associated with either genetic or anatomic predisposition [1], there was no difference in survival between those with and those without.

### Clinical presentation

Children usually have more advanced disease compared to adult patients, characterised by more frequent distant disease and lower rate of localised tumours [2]. Clinical signs and symptoms of paediatric and adolescent HCC include abdominal mass and pain, hepatosplenomegaly and gastric reflux. Paediatric HCC have larger tumour size at the time of detection, being >4 cm in 79.6% of cases in children and 62% in adults [2]. Advanced cases are often associated with cachexia and jaundice. In addition, those patients with inherited metabolic diseases or chronic liver diseases often show some concomitant hepatic dysfunction [1].

### Workup

Laboratory tests: Alpha-fetoprotein (AFP), white blood cell count (WBC), haematocrit, platelet count, absolute neutrophil count, electrolytes, lactate, prothrombin - international normalized ratio (PT/INR), AST/ALT, gamma glutamyl transferase, fractionated bilirubin, choline esterase, alpha-1 antitrypsin and vitamin-B12-binding proteins (transcobalamin-1).

Only 55%–67% of children with HCC have elevated blood level of AFP, while in one third of patients the AFP might be normal [11]. The levels of vitamin-B12-binding proteins especially transcobalamin-1, are useful markers to monitor disease progression and response to therapy.

Imaging: Abdominal ultrasound to confirm liver as organ of origin, computed tomography (CT) chest and CT/ magnetic resonance imaging (MRI) abdomen with intravenous contrast. After administration of contrast, triphasic CT shows HCC to be hypovascular in the arterial phase and isodense or hypodense in the portal venous phase. MRI gives good definition of tumour location and surrounding infiltration. HCC on MRI tend to be heterogeneous masses on T1-weighted images and mildly hyperintense on T2-weighted images. Hepatocyte specific contrast increases ability to detect multifocal nodules (Gadoxetate disodium/Eovist, Primovist or gadopentetate dimeglumine/gadotex). Positron emission tomography scan imaging is useful to detect localised relatively early small metastases or recurrence of disease when any mass effect is difficult to be detected on routine imaging.

### Differential diagnosis between HB and HCC

The differential diagnosis between these two entities fundamentally depends on histology. HB with embryonal-type epithelial, or mesenchymal elements is easily defined. But, for some with macrotrabecular architecture or with purely well-differentiated foetal epithelial histology, distinguishing between the two is difficult. Immunohistochemical profiles are frustratingly similar. Gene aberration or expression studies will provide a different pattern in HCC in comparison to HB.

The age of the child is important. HB usually occurs under 5 years of age and sometimes in children born with very-low-birth-weight or with a multisystemic syndrome such as Beckwith–Wiedemann syndrome. On the other hand, the presence of underlying liver diseases might indicate HCC.

Recently, international classification of paediatric liver tumour proposed a category of ‘HCN-NOS’ to acknowledge the difficulty of this differential diagnosis [4, 12]. The HCN-NOS, which is almost same as previous entity of transitional liver cell tumour, occurs in older children but is treated as HB.

FL-HCC, a rare variant of HCC, usually affects AYA and is a distinctive neoplasm arising in non-cirrhotic liver. Serum levels of AFP are not as elevated as for most HB/HCC. This tumour is typically composed of large cells in a lamellated hyalinised stroma. The immunohistochemically profiles contain both hepatocellular and biliary markers. It shows fewer genetic alterations and less methylation in comparison with conventional HCC [8].

### Clinical staging

There is no uniformly accepted staging system in paediatric AYA HCC. The widely accepted system is the Barcelona Clinic Liver Cancer score [13], which is correlated with Child–Pugh system. In practice, the pretreatment extent of disease (PRETEXT) system, developed in the HB classification and amended in 2017 [14, 15], may be suitable for the physicians and surgeons who treat paediatric liver tumours including HB and HCN-NOS and currently used in the Pediatric Hepatic International Tumour Trial (PHITT).

Biochemical liver function tests and clinical grading systems only provide indirect information about liver function. Therefore, especially in patients with non-cirrhotic livers, there is a need for objective tests to evaluate liver function in addition to clinical judgment. To this end, several dynamic quantitative tests of liver function have been devised [16]. As the various functional tests are based on different metabolic pathways, it is difficult to compare the value of each test in the context of risk assessment for liver resection. Several of these tests are discussed below.

**Biopsy**: Unless primary surgery is feasible, biopsy is required for diagnosis in all patients without cirrhosis. In patients with liver cirrhosis, tumour biopsy may be required in equivocal cases, such as small lesions less than 2 cm in diameter. Image-guided needle biopsy is preferable for multifocal tumours. To reduce the risk of tumour seeding and haemorrhage, attention should be paid to the following:

1) Do not approach the tumour directly, but biopsy through the unaffected liver, taking care to cross only the segments that will be resected at subsequent surgery. 2) A coaxial biopsy system should be used to allow several cores of tissues to be obtained with a single puncture. 3) The needle tract should be plugged/embolised with the patient’s blood or artificial foams such as gelatin or collagen.

## Treatments

The fundamental management of all hepatic malignancies consists of a combination of surgery and chemotherapy. The cornerstone for HCC is a complete surgical resection including liver transplantation (LT), however two‐thirds of paediatric patients with HCC present with unresectable disease (Table 1).

## Surgical Procedures in Paediatric HCC

### Local treatment: hepatectomy

Radical tumour resection is the cornerstone of cure for HCC. If the tumour is localised to the liver, primary surgical resection with negative margins is recommended. Especially, in an older child or AYA with a background of liver disease who presents with a resectable HCC or HCC suspected tumour, primary resection should be considered taking into account the function of the remnant liver and the risk of recurrence or *de novo* tumour in the diseased liver (field defect) in the predisposition of the background disease. Unlike HB where only PRETEXT I and II tumours are recommended for resection at diagnosis, with HCC any tumour confined to the liver should be evaluated for possible resection at diagnosis if this is technically feasible. This is because of the relative chemoresistance of HCC and the unrealistic hope that preoperative chemotherapy can reliably be expected to make the tumour more resectable. If the patient has underlying liver disease or dysfunction, the remaining functional liver volume and carcinogenicity of the liver remnant determine the surgical resectability of HCC. Since these background diseases vary, the condition of the liver should be carefully evaluated case by case.

For details of the technical aspects of the different types of liver resections (Please see *Hepatoblastoma Guidelines*). In the ‘*IPSO Hepatoblastoma Guidelines*’, the technical details of sectionectomy, hemihepatectomy, extended hemihepatectomy and trisectionectomy are described in detail.

In paediatric and AYA HCCs, 30%–50% of paediatric patients will have a background of cirrhosis or underlying liver disease [1, 3]. Patients with extensive PRETEXT III and IV tumours, and concomitant background liver disease, may need to be treated at specialised centres with experience in liver surgery including liver transplant and intensive care. Surgical consideration will have to take into account the possible compromised function of the remnant liver as well as the possible need to treat postoperatively for chronic liver disease.

Because of the relative chemoresistance of HCC, the resection margin is much more important than with HB. Multiple reports in adults have shown decreased survival associated with a microscopic positive margin and hence the ultimate goal of surgery is to achieve complete tumour resection with at least a 1 cm of safety margin. However, some reports on adult HCC show that any clear margin (<1 cm) may be acceptable. A complete margin negative resection and regional lymphadenectomy is recommended whenever possible [5].

Therefore, to define surgical procedure of initial resection or indication of LT, patients should be referred to the units of liver surgery that also have access to LT unit, preoperatively. The volume of the liver that can be removed by extensive resection can be predicted by CT or MRI based calculation. In children, the usual limit of the remnant liver volume in millilitre by patient body weight in kilogram must exceed 0.8 mL/kg; however, in children with underlying liver dysfunction this may need to be more [1].

Intraoperative ultrasound examination is useful for determining the boundaries of the tumour, the proximity to major vasculature and safe resection planes. Recently, indocyanine green navigation with direct intrahepatic portal branch has been proven useful for identifying tumours and their margins [22].

Recently, computer-assisted surgery planning substantially contributed to the decision for surgical strategies in children with complex hepatic tumours. This tool possibly allows the determination of specific surgical procedures such as extended surgical resection in the future [23, 24].

Lymph node sampling in the hepatoduodenal ligament should be done in all cases because lymph node involvement is correlated to outcome [25, 26]. In the cases that underwent initial resection, the recurrence rate was 20%–30%, which has not improved in the past decade.

### Liver transplantation

In the case of localised and nonmetastatic HCC, surgical resection at diagnosis, even by extreme resection or LT, should be considered. Since a total hepatectomy may be the only way to completely resect large or multifocal HCC, LT may need to be considered. Not only can LT provide a chance for a cure, but transplantation has made significant progress and is currently associated with relatively low morbidity and mortality rates [27–29].

The role of LT in the treatment strategy in paediatric/AYA HCC may not be the same as in adults. The indication for LT in adults is restricted to the Milan criteria, i.e. the evidence of a single tumour < 5 cm in size or no more than three foci with each not exceeding 3 cm and no vascular invasion or extrahepatic involvement [30]. Recently, patients transplanted outside Milan and University of California at San Francisco (UCSF) criteria had event-free survival (EFS) of 82% and overall survival (OS) of 78%, respectively, with older age and metastatic disease associated with worse outcomes [5, 31]. The practice guidelines of the American Association for Transplantation and the North American Society for Pediatric Gastro-enterology, Hepatology and Nutrition recommend that the indication for LT in childhood/AYA HCC must be discussed individually for each patient. Specific transplantation criteria for paediatric/AYA patients suffering from non-metastatic HCC should be developed [32].

There have been no prospective and randomised studies between partial hepatectomy and LT in children and AYA. Although the reports of LT in paediatric/AYA HCC are limited, the survival of the patients who underwent LT has improved and no significant correlation has been found between survival, tumour size and vascular invasion. Full resection with negative margins is the cornerstone of good outcomes [33]. Because of the biological difference between paediatric/AYA and adult HCC, adult experience might not be extrapolated into child and AYA patients [32]. In general, contraindications for LT include the existence of extrahepatic disease and FL-HCC.

## Chemotherapy

Complete surgical resection is fundamental for cure of HCC. However, less than 20% of the paediatric/AYA HCC patients are considered eligible for initial resection. Historically, in North American Intergroup Hepatoma study (INT-0098) and the International Childhood Liver Tumor Strategy Group study (SIOPEL1), HCC patients have been treated with the same protocols as patients with HB. The agents used include cisplatin, doxorubicin, pirarubicin, carboplatin, 5-FU and vincristine [17, 18]. In the German Society for Pediatric Oncology and Hematology (GPOH), HCC has been treated by the same regimen as HB with recommendation of upfront surgery to primary treatment in resectable patients. The adjuvant chemotherapy in HB99 consisted of two courses of carboplatin (200 mg/m2/d × 4) and etoposide (100 mg/m2/d × 4) and 3-year EFS and OS were 72% and 89%, respectively. In non-resectable HCC, GPOH used high-dose carboplatin and etoposide regimen but did not show increase in resectability, nor improved outcome, with preoperative chemotherapy.

On the other hand, increasing experience in adult HCC provides interest in exploring newer targeted therapies in paediatric/AYA HCC. In a placebo-controlled randomised study, sorafenib, which is a multikinase inhibitor targeting vascular endothelial growth factor (VEGF) and the Raf kinase pathway in combination with doxorubicin showed significant better progression-free survival and tumour shrinkage in advanced HCC in adults [34]. Recently, GPOH tried to use sorafenib with PLADO in paediatric HCC patients. Six of 12 localised HCC achieved complete remission and 3 of 7 unresectable HCCs showed partial response. Several approaches with the combination of sorafenib such as gemcitabine/oxaliplatin (GEMOX) have been tried in adult HCC. These two regimens are now tested as a randomised study for unresectable HCC in the PHITT in North America (AHEP1531), Europe and East Asia including Japan (JPLT4) for evaluating efficacy and tolerable toxicity in children and AYA. In this trial, Childhood/AYA patients with HCC are assigned to treatment on either resectable or unresectable tumours. In resectable HCC, the patients with underlying diseases are followed as observation and those with *de novo* HCC are treated by PLADO regimen (cisplatin and doxorubicin). Unresectable HCCs are treated by the randomised study of PLADO with sorafenib versus GEMOX with sorafenib (Figure 1).

The biological pathways that lead to oncogenesis and progression of HCC have the potential of developing targeted therapies for HCC. One example is the VEGF/VEGF receptor pathway which play roles in initiation, progression and dissemination of HCC, suggesting that sorafenib, bevacizumab, brivanib and sunitinib take advantage to inhibit tumour growth. Erlotinib, which targets the epidermal growth factor pathway is also a phase 2 study as a single agent and in combination with sorafenib. In addition, the mammalian target of rapamycin pathway inhibitor everolimus has shown antitumour effect in adult HCC. There has also been interest in agents targeting cMET, a tyrosine kinase receptor for the hepatocyte growth factor, in HB and HCC. Recently, phase 2 randomised study of the cMET inhibitor receptor tivantinib has demonstrated activity in patients with advanced HCC that had progressed on sorafenib. Cabozantinib, dual inhibitor to cMET and VEGF pathways, might have a potential to target advanced HCC.

FL-HCC in adults has been considered to be a slower growing tumour than conventional HCC and may metastasise at a later phase. Adult protocols often recommend it be treated surgically without the need for adjuvant chemotherapy. Experience with FL-HCC in children is less well described and prior paediatric studies have treated it the same as conventional HCC with similar outcomes [6, 35].

## Ablative Therapies

Radiofrequency ablation and percutaneous ethanol injection are the most common methods of percutaneous ablation in adult HCC. These have been proved to be comparable to surgery for tumours less than or equal to 3 cm diameter. There is limited experience with percutaneous ablative therapies in children [32].

Chemoembolisation: Palliative Transarterial chemoembolisation (TACE) (transfemoral hepatic artery chemoembolisation) is a standard procedure in adults with solitary or multifocal HCC without extrahepatic metastases. However, in children, reported cases are few. In 2000, Malogolowkin *et al* [36] reported that all 11 children (18 months–14 years old) treated with TACE for unresectable, chemotherapy-resistant liver tumours (three with HCC) responded. Five of these patients (one with HCC) went on to surgical resection and three survived. TACE with a suspension of cisplatin, doxorubicin and mitomycin mixed with lipiodol is feasible, well-tolerated and effective in achieving surgical resectability in paediatric patients. These encouraging results were confirmed by Czauderna *et al* [37] (five patients, 1–12 years old, one with HCC). Thus, TACE could be offered to patients with chemotherapy-resistant liver tumours for palliative care or even with the goal of achieving surgical resectability and cure. The experience of TACE in children is limited and pulmonary embolism has been reported. Moreover, as TACE may be associated with thrombosis of hepatic artery or its branches, it may interfere with further surgical resection. On the other hand, TACE could be offered to patients with chemotherapy-resistant liver tumours to potentially achieve surgical resectability as well as in palliative care [19, 37–39]. TACE should be considered after multidisciplinary discussion and taking into account the surgical options at the end of the treatment.

## Outcome

Multivariable Cox regression analysis was done using children with HCC (<18 years) reported to the National Cancer Database (2004–2015) [33]. T stage disease and tumour histology (fibrolamellar versus not) were not associated with OS. LT displayed a survival benefit when compared to either margin negative or margin positive resection. Chemotherapy and tumour size (>5 cm) were not associated with OS in this cohort. Vascular invasion (*p* < 0.001) and number of tumours (*p* < 0.001) were highly correlated with T stage, and thus were not included in the multivariable model (Table 2).

Paediatric/AYA HCCs are obviously different from adult HCCs in cirrhotic patients [40]. Research has to be performed to better characterise the pathological, molecular and genetic mechanisms of paediatric/AYA HCC, to support the development of novel diagnostic and therapeutic approaches (including surgery) and the implementation of personalised medicine. The most recent published trial results for three of the major multicentre trial groups involved in the study of resectable and unresectable HCC are shown in Figure 1. This is the first randomised trial for paediatric/AYA HCC. Such global trials for paediatric/AYA HCC promote the identification of suitable treatments and highlight the difference between paediatric/AYA HCCs and adult HCCs.

## Figures and Tables

**Figure 1.** Paediatric/AYA HCC treatment strategies in PHITT. PLADO, Cisplatin/doxorubicin; GEMOX, Gemcitabine/oxaliplatin.

**Table 1.** Response to chemotherapy and resection rates in paediatric HCC and FL-HCC trials.

Trial	Chemotherapy	PR rate	Resection rate
INT 0098 [11]	PLADO versus C5V	21% (8/38)	5% (2/38)
International Childhood Liver Tumor Strategy Group study (SIOPEL 1) [17]	PLADO	49% (18/32)	63% (12/19)
SIOPEL 2 [18]	Cisplatin, carboplatin, doxorubicin	50% (29/58)	44% (14/32)
HB99 (GPOH) [19]	Carboplatin and etoposide with autologous **Stem cell transplantation**	47% (7/15)	36% (8/22)
JPLT-2 [20]	CITA	45% (5/11)	27% (3/11)
PLADO/sorafenib [21]	Cisplatin, doxorubicin, sorafenib	57% (4/7)	29% (2/7)

PLADO, Cisplatin and doxorubicin; C5V, Cisplatin, 5-fluorouracil (5-FU) and vincristine; CITA, Cisplatin and pirarubicin

**Table 2.** Cox proportional hazards models for all-cause mortality rate in the National Cancer Database queried (2004–2015) for children [33].

Characteristics	Univariable			Multivariable (*n* = 93)		
	*n*	HR (95% CI)	*p*-Value	*n*	HR (95% CI)	*p*-Value
Fibrolamellar histology						
No	64	Ref.		55	Ref.	
Yes	42	0.96 (0.41–2.26)	0.93	38	0.33 (0.10–1.05)	0.06
T						
T1	36	Ref.		32	Ref.	
T2/T3/T4	68	3.62 (1.07–12.23)	0.04	61	4.50 (0.91–22.20)	0.06
Size						
≤5 cm	35	Ref.		34	Ref.	
>5 cm	62	2.41 (0.80–7.22)	0.12	59	0.64 (0.12–3.29)	0.59
Chemotherapy						
No	53	Ref.		49	Ref.	
Yes	48	3.52 (1.28–9.71)	0.02	44	1.70 (0.46–6.22)	0.42
Surgical treatment						
LT	34	Ref.		33	Ref.	
LR(−)	62	2.20 (0.63–7.74)	0.22	53	6.53 (1.21–35.30)	0.03
LR(+)	8	9.41 (2.22–39.87)	0.002	7	42.73 (4.83–378.07)	0.001

CI, Confidence interval; HR, Hazard ratio; LR (−), Margin-negative liver resection; LR (+), Margin-positive liver resection; LT, Liver transplantation.

## References

[1] Kelly D, Sharif K, Brown RM (2015). Hepatocellular carcinoma in children. Clin Liver Dis.

[2] Lau CS, Mahendraraj K, Chamberlain RS (2015). Hepatocellular carcinoma in the pediatric population: a population based clinical outcomes study involving 257 patients from the surveillance, epidemiology, and end result (SEER) database (1973-2011). HPB Surg.

[3] Khanna R, Verma S (2018). Pediatric hepatocellular carcinoma. World J Gastroenterol.

[4] Lopez-Terrada D, Alaggio R, de Davila MT (2014). Towards an international pediatric liver tumor consensus classification: proceedings of the Los Angeles COG liver tumors symposium. Mod Pathol.

[5] Darcy DG, Malek MM, Kobos R (2015). Prognostic factors in fibrolamellar hepatocellular carcinoma in young people. J Pediatr Surg.

[6] Katzenstein HM, Krailo MD, Malogolowkin MH (2003). Fibrolamellar hepatocellular carcinoma in children and adolescents. Cancer.

[7] Xu L, Hazard FK, Zmoos AF (2015). Genomic analysis of fibrolamellar hepatocellular carcinoma. Hum Mol Genet.

[8] Haines K, Sarabia SF, Alvarez KR (2019). Characterization of pediatric hepatocellular carcinoma reveals genomic heterogeneity and diverse signaling pathway activation. Pediatr Blood Cancer.

[9] D’Souza AM, Towbin AJ, Gupta A (2020). Clinical heterogeneity of pediatric hepatocellular carcinoma. Pediatr Blood Cancer.

[10] Varol FI (2020). Pediatric hepatocellular carcinoma. J Gastrointest Cancer.

[11] Katzenstein HM, Krailo MD, Malogolowkin MH (2002). Hepatocellular carcinoma in children and adolescents: results from the Pediatric Oncology Group and the Children’s Cancer Group intergroup study. J Clin Oncol.

[12] Zhou S, Venkatramani R, Gupta S (2017). Hepatocellular malignant neoplasm, NOS: a clinicopathological study of 11 cases from a single institution. Histopathology.

[13] Golfieri R, Bargellini I, Spreafico C (2019). Patients with Barcelona clinic liver cancer stages B and C hepatocellular carcinoma: time for a subclassification. Liver Cancer.

[14] Roebuck DJ, Aronson D, Clapuyt P (2007). 2005 PRETEXT: a revised staging system for primary malignant liver tumours of childhood developed by the SIOPEL group. Pediatr Radiol.

[15] Towbin AJ, Meyers RL, Woodley H (2018). 2017 PRETEXT: radiologic staging system for primary hepatic malignancies of childhood revised for the Paediatric Hepatic International Tumour Trial (PHITT). Pediatr Radiol.

[16] Chen JC, Chen CC, Chen WJ (1998). Hepatocellular carcinoma in children: clinical review and comparison with adult cases. J Pediatr Surg.

[17] Czauderna P, Mackinlay G, Perilongo G (2002). Hepatocellular carcinoma in children: results of the first prospective study of the International Society of Pediatric Oncology group. J Clin Oncol.

[18] Czauderna P, Maibach R, Aronson D (2003). Hepatocellular carcinoma in children: results of the secound prospective study of the International Society of Paediatric Oncology (SIOP): SIOPEL2. Med Pediatr Oncol.

[19] Schmid I, Schweinitz D (2017). Pediatric hepatocellular carcinoma: challenges and solutions. J Hepatocell Carcinoma.

[20] Hiyama E (2013). Current therapeutic strategies for childhood hepatic malignant tumors. Int J Clin Oncol.

[21] Schmid I, Haberle B, Albert MH (2012). Sorafenib and cisplatin/doxorubicin (PLADO) in pediatric hepatocellular carcinoma. Pediatr Blood Cancer.

[22] Ishizawa T, Saiura A, Kokudo N (2016). Clinical application of indocyanine green-fluorescence imaging during hepatectomy. Hepatobiliary Surg Nutr.

[23] Warmann SW, Schenk A, Schaefer JF (2016). Computer-assisted surgery planning in children with complex liver tumors identifies variability of the classical Couinaud classification. J Pediatr Surg.

[24] Procopio F, Cimino M, Vigano L (2020). Prediction of remnant liver volume using 3D simulation software in patients undergoing R1vasc parenchyma-sparing hepatectomy for multiple bilobar colorectal liver metastases: reliability, clinical impact, and learning curve. HPB (Oxford).

[25] Lee CW, Chan KM, Lee CF (2011). Hepatic resection for hepatocellular carcinoma with lymph node metastasis: clinicopathological analysis and survival outcome. Asian J Surg.

[26] McAteer JP, Goldin AB, Healey PJ (2013). Hepatocellular carcinoma in children: epidemiology and the impact of regional lymphadenectomy on surgical outcomes. J Pediatr Surg.

[27] Baumann U, Adam R, Duvoux C (2018). Survival of children after liver transplantation for hepatocellular carcinoma. Liver Transpl.

[28] Namgoong JM, Choi JU, Hwang S (2019). Pediatric living donor liver transplantation with homograft replacement of retrohepatic inferior vena cava for advanced hepatoblastoma. Ann Hepatobiliary Pancreat Surg.

[29] Ziogas IA, Ye F, Zhao Z (2020). Population-based analysis of hepatocellular carcinoma in children: identifying optimal surgical treatment. J Am Coll Surg.

[30] Abdelfattah MR, Elsiesy H, Al-Manea H (2018). Liver transplantation for hepatocellular carcinoma within the Milan criteria versus the University of California San Francisco criteria: a comparative study. Eur J Gastroenterol Hepatol.

[31] Pham TA, Gallo AM, Concepcion W (2015). Effect of liver transplant on long-term disease-free survival in children with hepatoblastoma and hepatocellular cancer. JAMA Surg.

[32] de Ville de Goyet J, Meyers RL, Tiao GM (2017). Beyond the Milan criteria for liver transplantation in children with hepatic tumours. Lancet Gastroenterol Hepatol.

[33] Ziogas IA, Benedetti DJ, Matsuoka LK (2020). Surgical management of pediatric hepatocellular carcinoma: an analysis of the National Cancer Database. J Pediatr Surg.

[34] Rimassa L, Santoro A (2009). Sorafenib therapy in advanced hepatocellular carcinoma: the SHARP trial. Expert Rev Anticancer Ther.

[35] Weeda VB, Murawski M, McCabe AJ (2013). Fibrolamellar variant of hepatocellular carcinoma does not have a better survival than conventional hepatocellular carcinoma--results and treatment recommendations from the Childhood Liver Tumour Strategy Group (SIOPEL) experience. Eur J Cancer.

[36] Malogolowkin MH, Stanley P, Steele DA (2000). Feasibility and toxicity of chemoembolization for children with liver tumors. J Clin Oncol.

[37] Czauderna P, Zbrzezniak G, Narozanski W (2006). Preliminary experience with arterial chemoembolization for hepatoblastoma and hepatocellular carcinoma in children. Pediatr Blood Cancer.

[38] Lungren MP, Towbin AJ, Roebuck DJ (2018). Role of interventional radiology in managing pediatric liver tumors : Part 1: Endovascular interventions. Pediatr Radiol.

[39] Yamada K, Shinmoto H, Kawamura Y (2015). Transarterial embolization for pediatric hepatocellular carcinoma with cardiac cirrhosis. Pediatr Int.

[40] Weeda VB, Aronson DC, Verheij J (2019). Is hepatocellular carcinoma the same disease in children and adults? Comparison of histology, molecular background, and treatment in pediatric and adult patients. Pediatr Blood Cancer.

# Discussion: Extracranial germ cell tumour

**Published:** 2022

**Authors:** Sajid Qureshi, Marianna Cornet, Alessandro Crocoli, Patrizia Dall’Igna, Sabine Sarnacki

## Introduction

### Epidemiology

Extracranial germ cell tumours (GCTs) are rare, accounting for approximately 3% of cancers in children younger than 15 and 14% of cancers in adolescents aged 15–19 years [1, 2]. GCT occurs more commonly in patients with cryptorchidism, gonadal dysgenesis and patients with Klinefelter syndrome, Swyer syndrome and Turner syndrome [3–5].

### Clinical presentation

GCT may arise in the gonads or various extragonadal extracranial sites, including sacrococcygeal, mediastinal, retroperitoneal and other para-axial locations. The clinical features at presentation are specific for each site.

### Preoperative evaluation

Biology: Complete blood count, complete metabolic profile, tumour markers (alpha-fetoprotein (AFP), beta-human chorionic gonadotropin (βHCG), lactate dehydrogenase (LDH)) and coagulation profile. Although AFP is a relevant marker in more than 90% of yolk sac tumours, the clinical picture in infancy can be confusing since infants up to the age of 6–8 months have raised serum AFP, exceptionally high immediately after birth, and AFP does not attain its average half-life of 5 days until 4 months of age. A return to normal concentrations sometimes takes as long as 12 months [4]. In yolk sac tumours, serum AFP concentration is increased and does not show the expected decrease with time.

Imaging: Chest radiograph or computed tomography (CT) chest, abdominal ultrasound and CT/magnetic resonance imaging abdomen are done according to the location. Basic information on imaging for surgical planning includes the following:

1. Evaluation of the primary tumour extent and the regional lymph nodes.
2. Relation of the tumour with surrounding organs and vascular structures.
3. Evaluation of compartmental dissemination and pulmonary metastasis.
4. Evaluation of tumour response and pulmonary metastasis response to chemotherapy.

**A patient with suspected GCT and elevated tumour markers usually will NOT require a diagnostic biopsy. A biopsy in the context of an atypical presentation or normal levels of tumour markers may be considered.**

## Sacrococcygeal Germ Cell Tumour

The sacrococcygeal region is the most familiar extragonadal site for benign and malignant GCT seen in neonates, infants and children younger than 4 years [6]. They may present at birth with a large mass protruding from the perineal region. Surgery is an essential component of treatment. Complete resection of the coccyx is vital to minimise tumour recurrence, which has been reported as 2%–35% with an odds ratio of 17.78 when complete resection is not possible [7]. Surgery may be facilitated by preoperative chemotherapy for malignant GCT.

### Indications for surgery

Upfront resection: For teratoma, including antenatal surgery at <28 weeks’ gestation (foetal intervention), between 28 and 36 weeks’ gestation (EXIT procedures) and well-circumscribed malignant GCT where a complete surgical excision is feasible without added morbidity. After-birth procedures are preferred, antenatal procedures should be considered if physiologic impairment of the foetus is severe.

Delayed resection after preoperative chemotherapy: For most malignant GCT of the sacrococcygeal region.

### Surgery goals

Complete and safe surgical excision together with the coccyx and to avoid tumour spillage during the operation.

### Anaesthesia considerations

Foetal and EXIT procedures require meticulous planning and execution and are preferably performed at specialised centres.

### Key steps

Approach:

- Posterior sagittal: For lesions confined to the perineal and low presacral region.
- Anterior: Laparotomy for high presacral lesions with a predominant abdominal component.
- Combined anterior and posterior: for large lesion involving the perineal and high presacral and abdominal extension.

### Surgical steps

Posterior sagittal approach: The patient is placed in the prone position. A vertical midline skin incision extending from the coccyx to near the anal orifice or a chevron incision is designed incorporating the redundant skin over the tumour to be excised ‘en-bloc’ with the tumour mass. The mass is dissected laterally on both sides from the gluteal muscles and the ischioanal fossa. The sacrococcygeal vertebral junction is divided, and the coccyx is included with the tumour mass. The median sacral artery will be exposed at this point, which is secured, and ligated. The dissection continues freeing the tumour mass from the posterior wall of the rectum made prominent by inserting an appropriate-sized Hegar dilator through the anal orifice. After excision of the tumour, the perineal wound is closed vertically by reapproximating the pelvic floor muscles in the midline behind the rectum. Electrostimulators can facilitate muscle reconstruction. However, complete reapproximation may not be possible, especially after resection of malignant GCT. A drain is left in the tumour bed, getting out through a separate lateral skin incision.

### Tips, pitfalls and complications

The key steps to prevent spillage are ensuring adequate access and gentle handling of the tumour. Early ligation of the middle sacral artery for large vascular lesions reduces significant operative blood loss risk. Separation from the posterior wall of the rectum can be challenging, and consent for diversion colostomy is essential. Injury to the rectum can be tested after tumour removal by insufflating normal saline into the anal canal. Buttock reconstruction following resection of large masses could be beneficial during the initial surgery.

### Postoperative considerations

The postoperative period is usually uneventful, and drains are removed once the quantity is reduced. Wound infection remains a concern. Neurogenic bladder and continence may result in large tumours. It is highly recommended to follow these patients for 3 years to detect any benign or malignant recurrence (Ultrasonography (US) and AFP levels three times a year), which occur in 7%–10% of cases.

## Mediastinal Germ Cell Tumour

(please refer to *Thoracic Tumour Guidelines*)

Mediastinal GCT accounts for approximately 3%–4% of paediatric GCT [8–10]. The majority of children present with respiratory symptoms, chest pain or superior vena cava syndrome [11]. The histological type of GCT in the mediastinum includes benign teratoma, especially in infants and yolk sac tumour among older children [9]. Radiological evaluation and measurements of the tumour markers AFP and HCG help decide the treatment strategy. The radiologic observation of cystic structures or calcifications may suggest a teratoma, and a primary tumour resection will be most appropriate in these patients. In other patients for whom the tumour is unresectable because of size or infiltration of vital organs, chemotherapy even without a biopsy can be initiated. However, there may be a diagnostic dilemma with nonsecreting tumours, and a biopsy (image-guided needle biopsy or open biopsy) may help achieve diagnosis [9].

### Indications for surgery

Upfront resection: For teratoma and well-circumscribed lesion where a complete surgical excision is feasible without added morbidity.

Delayed resection after preoperative chemotherapy: For most malignant GCT of the mediastinum.

### Surgery goals

Complete and safe surgical excision.

### Anaesthesia considerations

The presence of orthopnoea, airway compression (>35%–50%) on imaging or a peak expiratory flow rate of <50% predicted is considered at elevated risk for cardiovascular or airway collapse with general anaesthesia [11, 12]. These patients should be meticulously planned for any surgical intervention in a multidisciplinary team comprising an anaesthetist, intensivists, surgeon and cardiothoracic team.

### Key steps

Approach:

- Median sternotomy: for lesions confined to the anterior mediastinum.
- Clam-shell thoracotomy: for lesions extending into both thoracic cavities.
- Hemi clam-shell thoracotomy: for lesions extending in one thoracic cavity.
- Posterolateral thoracotomy: for lesions with a predominant thoracic cavity component.

### Surgical steps

Following adequate exposure, the extent of the lesion and relations with adjacent structures are confirmed. Generally, the thymus is involved or inseparable from the lesion; hence a total or partial thymectomy is usually required. In doing so, the thymic veins, usually a pair of veins draining into the left brachiocephalic vein, should be secured. Separation from the parietal pericardium is generally possible; however, pericardiectomy may be required. Lesions extending into the pulmonary hilum are carefully separated to avoid injury to the phrenic and the vagus nerve. Meticulous dissection for tumour extensions around the great vessels is performed. Rarely a vascular resection and graft are required to reconstruct the superior vena cava or the large branches from the aorta. Dense adhesion with the lung parenchyma may require wedge excision or a lobectomy.

### Tips, pitfalls and complications

Intrapericardial extension and dissection around the arch of the aorta and aortopulmonary window should be planned along with a cardiothoracic surgeon. The sacrifice of the phrenic nerve may require plication of the diaphragm to prevent eventration.

### Postoperative considerations

The postoperative period is usually uneventful; however, pain management is crucial. Mediastinal and thoracic cavity drains are removed once the quantity is reduced. Postoperative ventilator support, mediastinitis and sternal wound infection are concerns.

## Abdominal and Retroperitoneal Germ Cell Tumour

Primary retroperitoneal GCT accounts for less than 4% of all GCT [13]. The majority of these tumours are teratomas, while malignant GCT occurs in approximately 15% of cases [13].The differentiation between benign and malignant GCT is established by the presence of characteristic imaging features (calcification, fat density, cystic areas) and tumour markers (AFP, HCG). An image-guided core biopsy is required when the imaging features are uncharacteristic and the tumour markers are negative.

### Indications for surgery

Upfront resection: For teratomas and well-circumscribed malignant GCT, complete surgical excision is feasible without added morbidity.

Delayed resection after preoperative chemotherapy: For most malignant GCT of the retroperitoneal region.

### Surgery goals

Complete and safe surgical excision and avoid tumour spillage during the operation.

### Key steps

Approach:

- Transperitoneal
- Laparoscopy (unsuitable for large lesions)

### Surgical steps

The retroperitoneum is exposed by reflecting the colon and the spleen towards the midline. The tumour is dissected all around, avoiding a rupture of the capsule. Displaced or encased blood vessels are meticulously dissected from the tumour, securing all the feeding or draining tributaries. All efforts are made to prevent the removal of adjacent organs; however, the exceptional presence of extensive vascular stretching or distortion compromising the vascularity of the affected organ may necessitate an excision.

### Tips, pitfalls and complications

The surgical difficulties emanate from the need for extensive vascular dissection and distortion of adjacent structures, which may necessitate their removal [14]. These difficulties may be compounded by blood loss and occasionally incomplete removal of the tumour.

### Postoperative considerations

Extensive retroperitoneal dissection may necessitate prolonged intensive care and judicious fluid management.

## Head and Neck Germ Cell Tumour

Benign and malignant GCTs of the head and neck region are the rarest primary sites of extragonadal GCTs [15]. Generally, they present as huge masses that frequently cause airway obstruction and high perinatal mortality. The other areas in the head and neck region include the orbit, oral cavity and maxillary sinus.

### Indications for surgery

Upfront resection: For teratoma, including antenatal surgery at <28 weeks’ gestation (foetal intervention), between 28 and 36 weeks’ gestation (EXIT procedures) and well-circumscribed malignant GCT where a complete surgical excision is feasible without added morbidity. After-birth procedures are preferred.

Delayed resection after preoperative chemotherapy: For malignant GCT.

### Surgery goals

Complete and safe surgical excision without injury or sacrifice of vital neck structures.

### Anaesthesia considerations

Foetal and EXIT procedures require meticulous planning and execution and are preferably performed at specialised centres. These patients should be meticulously planned for any surgical intervention in a multidisciplinary team, including anaesthetists, surgeons, head and neck specialists and plastic and reconstructive surgeons.

### Tips, pitfalls and complications

Injury to the trachea or tracheomalacia can complicate the surgery and may necessitate a tracheostomy. Reconstructive surgery for soft tissue defects at the primary surgery is beneficial.

## Genitourinary Germ Cell Tumour

Genital lesions are primarily vaginal, and most patients present with vaginal bleeding with a protruding mass. Delayed surgical excision of residual disease after preoperative chemotherapy is curative in most cases and avoids radical surgeries [16].

## Gonadal Germ Cell Tumour

### Ovarian tumours

#### Epidemiology and histology of ovarian tumours

The incidence of ovarian tumours increases with age starting from 0.4 per 100,000 during infancy to 25–30 per 100,000 at the age of 18. Around 90% of these tumours will be benign, and the proportion of malignancy will increase from birth (18% malignant) until 6–7 years old (30% of malignancy) and will decrease drastically thereafter (less than 10% around 14 years old) [1].

At the paediatric age, four main groups are individualised according to the tissue origin of the tumour:

1. Germ cell tumours (GCTs) (60%–75%) which comprise 80% benign tumours (mature teratoma) and 20% malignant tumours (yolk sac tumours, choriocarcinoma, gonadoblastoma, germinomas also termed dysgerminomas in females, embryonal carcinoma and mixed malignant GCT, immature teratoma); the cell of origin is believed to be totipotent germ cells [18, 19]. Yolk sac tumour (YST) is the most frequent and aggressive malignant entity in young children that can metastasise to regional lymph nodes, liver, lung and brain. YST is characterised by the secretion of αFP. Choriocarcinoma is characterised by the secretion of HCG.
2. Epithelial cell tumours (10%–20%), mainly serous and/or mucinous cystadenoma in the paediatric population. In rare cases, the malignant cystadenocarcinoma component may be present in these tumours. These tumours are rare in the first decade and are primarily encountered in post-pubertal girls. However, according to Elias *et al* [20], a mucinous ovarian tumour may be more appropriately classified as GCT variants.
3. Sex cord stromal tumours (SCST), which cover juvenile granulosa cell tumours (JGCTs), Sertoli and/or Leydig cell tumours (SLCTs), rare theca cell tumours and unclassified SCST; they are developed from the peri-oocyte follicular and stromal cells. Ovarian SCST are endocrinologically active tumours. JGCT is characterised by secretion of Inhibin B. Incidence has been reported around 10% of all paediatric ovarian tumours before the age of 2, 20% between 2 and 8 and falling drastically thereafter to 2%–3% after the age of 10, as in adult patients [17]. SLCTs are reported to be associated with germline-inactivating DICER1 mutations as a part of the tumour spectrum of the DICER1 pleuropulmonary blastoma familial tumour predisposition syndrome [21–23].
  (Please also refer to *Non-GCT Guidelines*)
4. Secondary neoplasms, mainly Hodgkin’s disease, but also neuroblastoma, rhabdomyosarcoma (RMS), nephroblastoma, retinoblastoma or other haemopathy [24, 25].

#### Clinical presentation of ovarian tumours

Abdominal pain (70%–80%) and lower abdominal mass are the most common symptoms. GCT and epithelial cell tumours are often asymptomatic until they reach a considerable size with a palpable mass and compression of the neighbouring structures. Constipation, amenorrhoea, vaginal bleeding are less frequent [26]. About 10% of cases present as acute abdomen due to torsion, infarction or spontaneous rupture of the mass [27].

The pattern of regional spread of tumours with malignant components often includes ascites, retroperitoneal lymph nodes and peritoneal metastases.

The most specific clinical presentation will thus be associated with endocrinologically active tumours, namely SCST and choriocarcinomas. Specific signs of abnormal hormonal impregnation may frequently reveal them: premature pubarche, breast enlargement, vaginal bleeding, increased growth velocity, virilisation in prepubertal girls and menstrual disturbances (menometrorrhagia or secondary amenorrhoea) in adolescent girls [28].

In the clinical evaluation of any girl managed for ovarian tumour should appear Tanner scale (breast, and pubic hair development grading from 1 to 5), presence or absence of menses and regularity, face and chest hair growth assessment, presence of severe acne, enlargement of the clitoris or the labia or abnormal vaginal discharge for age.

Another particular clinical presentation is the one associated with dysgerminoma on dysgenetic gonads. In this case, an absent pubertal development will be the rule (no thelarche by age 13) except in very rare situations non-developed here.

#### Workup of ovarian tumours

Lab: AFP, HCG, Inhibin B, Anti-Mullerian hormone (AMH), Calcaemia, LDH. Complete blood count, complete metabolic profile and coagulation profile.

Evaluation of serum markers is essential to address the nature of the tumours. Elevation of AFP or HCG means the presence of malignancy.

Tumours that may be responsible for precocious pseudo-puberty or virilisation signs are, in order of frequency: 1) JGCT; inhibin B and AMH are very good markers at any age, and oestradiol is a very good marker in prepubertal children, 2) choriocarcinoma or GCT with a choriocarcinoma component, HCG is the marker, 3) SLCT, testosterone is the marker and 4) rarely theca cell tumours.

When an ovarian neoplasm is found to be a SLCT, a genetic screening for anomalies of the DICER1 gene at the germinal level is now mandatory as it could be the initial clinical presentation of a DICER1 tumour predisposition syndrome [21–23].

Imaging: Pelvic and abdominal ultrasound, abdominal CT/ magnetic resonance imaging (MRI) scan.

The ability to interpret cross-sectional imaging is essential for surgeons managing patients with ovarian tumours. MRI is the best exam to define the size, the structure of the mass and the involvement of neighbouring structures. Benign and malignant entities may have similar imaging features with solid and cystic components. On imaging, whereas mature teratomas will be more cystic with a small solid part (‘jingle bell’ sign), immature ones will present a more prominent solid component with small cysts around. However, caution is recommended in all cases because the histological subtype is not predictable at imaging evaluation.

Metastatic spread has to be considered when a malignant tumour is suspected. In these cases, thoracic and abdominal CT scans are required to evaluate the lungs, the liver and the retroperitoneal lymph nodes.

Diagnosis relies on three main features: 1) age and hormonal status at diagnosis, 2) imaging features and 3) biological markers (AFP, HCG, Inhibin B, calcaemia, LDH).

No ovarian biopsies should be performed because of the high oncological risk associated with peritoneal spread by malignant cells.

The major issue challenging the surgeon is to be able to perform conservative surgery for benign lesions but also to strictly follow the rules of carcinologic surgery for malignant lesions. The intrinsic contradiction between both approaches underlies the need to diagnose the malignant or benign nature of the lesion before surgery.

A multidisciplinary approach (surgeon, oncologist and imager) is highly recommended to avoid misdiagnosis of rare but potentially aggressive malignant tumours.

#### Surgery goals

The goals of surgery are to achieve R0 resection, prevent tumour spillage and make a precise staging of the disease. Therapy depends on accurate documentation of surgical findings, including peritoneal seeding and tumour spillage.

Surgery of ovarian tumours in children requires a good knowledge of these lesions. Complete resection is mandatory for malignant lesions, and in the case of benign tumours, preservation of healthy ovarian tissue is crucial. Laparoscopy is of great help to ensure diagnosis and staging. However, laparotomy should be preferred to avoid any tumour spillage and ensure a safe treatment in an unsuspected non-secreting malignant tumour.

The timing of surgery is relying on imaging and tumour marker levels. If the initial level of AFP is above 10,000 UI/L and/or HCG above 5,000 UI/L and/or if there are distant metastases, the tumour is considered as high risk and neoadjuvant chemotherapy should be considered. If neither of these risk factors is present, surgery may be performed as a first step if resection is anticipated to be R0.

When surgery is performed, laparoscopic exploration is recommended as the first step of the procedure. If the tumour is too huge arising above the umbilicus, exploration of the peritoneum by laparoscopy will be done after the open surgery. The goal of laparoscopy is to better appreciate the location and the nature of the lesion, and in the case of malignant tumour, to make a precise staging of the disease. Visualisation of the sus-mesocolic area and anterior parietal peritoneum is far better with an endoscopic camera than with a supra-pubic or lower median laparotomy. A sample of ascites or peritoneal washing (in the absence of ascites) is obtained for cytology and inspection and palpation of the peritoneal surfaces, including diaphragmatic domes, with biopsy of any suspicious areas, an inspection of abdominal organs, with particular attention to the liver, omentectomy if the omentum (or parts of it) is abnormal, pelvic and retroperitoneal lymph node inspection, with biopsy of abnormal nodes, and inspection of the contralateral ovary and biopsy only if macroscopically suspicious. The second step of the procedure is thus a laparotomy. A supra-pubic incision is ideally performed, preserving rectus muscles, which allows in many of the cases the exteriorisation and treatment of the lesion. In case of a very huge suspected malignant tumour, a median laparotomy can be performed to be sure not to rupture the tumour and do a one-piece resection.

Although proposed in some situations by some paediatric teams, laparoscopic tumoural resection is not the recommended approach by the present authors [29]. The rationales of open surgery for an ovarian tumour in children are:

- first, the possibility of facing a malignant tumour even if the lesion is cystic (rare cases of JGCTs) or appears as a mature teratoma (mix lesion with mature and immature component), exposing the patient to the worst prognosis if a per operative rupture occurs [30], and
- second the possibility to better preserve the peritumoural healthy ovarian parenchyma when benign histology is suspected [31]. The wish to favour aesthetic considerations in the treatment of ovarian masses in children could then lead to a chance loss, which seems unacceptable considering that the classic treatment proposes a supra pubic approach which results in the same post-operative course.

In all cases, protection of the operative field is highly recommended.

If the preoperative work-up favours a benign lesion (90% of cases), e.g., negative tumour markers and imaging features suggesting a benign teratoma, a partial ovariectomy is considered. The suprapubic incision gives a very good exposure to find a dividing plan ensuring carcinologic resection with preservation of healthy ovarian tissue. After exteriorisation of the pathological adnexa, a circular section is performed on the ovarian cortex near the fallopian tube. The cut surface should arrive at the direct contact of the lesion without opening it. Progressively the lesion will be peeled from the ovarian cortex, and haemostasis will be performed step by step with bipolar forceps. Once the lesion has been removed, the ovarian ‘pancake’ will be tubularised with a running suture in order to avoid post-operative adhesions. In the case of teratomas, if post-operative pathological analysis reveals a part of malignant contingent, a second procedure will be performed in order to complete the ovariectomy usually associated to omentectomy. The same approach will be made if any signs of malignancy or borderline patterns are revealed on histology of suspected benign surface epithelial tumours.

If there is any doubt on the benign nature of the lesion with the results of the preoperative exams (imaging and/or biology), complete unilateral ovariectomy or adnexectomy will be done. In this case, the enlargement of the cutaneous incision could be performed in order to avoid any tumour rupture or spreading during extraction. Adnexectomy will be done if the fallopian tube is involved.

If, during the exploratory step of the surgery, the tumour presents some malignant features (with vegetation or suspicious adherences), complete ovariectomy or adnexectomy should be performed with biopsies of suspicious lesions. If the complete resection of the tumour leads to the injury of an adjacent organ, resection should thus be cancelled, and neoadjuvant chemotherapy undertaken. In this case, a second surgery will be done to remove the residual tumours and/or lymph nodes.

In case of bilateral tumours, conservative surgery will be favour on both sides if the lesion is certainly benign (see above). Bilateral adnexectomy is mandatory for the exceptional bilateral ovarian malignant tumours. Ovarian cryopreservation may be discussed although most of the time, the amount of the remaining healthy tissue is not sufficient to retrieve later enough oogonias/oocytes.

In case of emergency, when the child presents with an acute abdomen because of adnexal torsion secondary to an ovarian lesion, recommended management should be a laparoscopy. The aim of the laparoscopy is to perform a cautious detorsion of the adnexal torsion, to explore the abdominal cavity and the contralateral ovary. This step is then followed by post-operative diagnosis workup (tumoural markers and imaging studies) before removing the tumour. Indeed, even if the diagnosis of benign lesion is certain, ovarian sparing surgery on a swollen ovary due to ischaemia can be challenging. A delayed tumourectomy is thus recommended.

#### Tips, pitfalls and complications

Tumour spillage can result in significant therapy escalation and have prognostic implications. The key steps to prevent spillage are ensuring adequate access and gentle handling of the tumour. Attempts to minimise access should not be made at the expense of sound oncologic principles. Recovery even after a large suprapubic incision is excellent (as there is not muscles cutting) and the patient may be discharged on the first or second postoperative day. In contrast, recurrence might be unsalvageable. Complete documentation of surgical staging improves local control strategy and outcome.

Chemotherapy is indicated for high-risk GCT, i.e., with incomplete resection or with pre or perioperative tumour spillage, with metastasis (6% of cases), with an initial level of AFP above 10,000 UI/L or other malignant tumours (dysgerminoma, JGCT, high-grade immature teratoma) with incomplete resection or tumour spillage. When indicated, chemotherapy is usually performed after surgery, but if the levels of preoperative tumour markers are high, or in cases with disseminated disease at diagnosis, it can be indicated before surgery.

#### Prognosis and follow-up of ovarian tumours

Five-year overall survival of non-seminomatous GCT is 85%–95%. Five-year overall survival of dysgerminoma is about 95% in localised forms and 75% for all stages. Five-year overall survival of localised JGCT is 83%–98%.

Of note, around 25% of ovarian teratomas are bilateral (either synchronous or metachronous) [32]. The contralateral tumour is more frequent during the first 3 years after primary surgery but can occur 15 years after the primary surgery. Ultrasound follow-up twice a year during the first 2 years and then annually is needed to enable early diagnosis, ovary preserving surgery and maintenance of fertility in the case of a metachronous tumour.

## Testicular Tumours

### Epidemiology and histology of testicular tumours

Prepubertal group

Prepubertal testicular tumours represent 1%–2% of all solid paediatric neoplastic lesions with an incidence of 0.5–2 per 100,000 children [33]. Testicular tumours show a bimodal age distribution, with a prominent peak in young adults and a much small, but distinct, peak in the first 3 years of life. Although the peak age at presentation in this group is 2 years, 60% of tumours present earlier, and the median age for the presentation of YSTs is 16 months and for teratomas is 13 months [34].

In contrast to testicular tumours in adolescents and adults, more than 75% of testicular tumours in prepubertal boys are benign [35–37]. At prepubertal age, two main groups are individualised according to the tissue origin of the tumour, GCTs and SCST.

1. GCTs which comprise 65% benign tumours (61% mature and 4% immature teratoma) and 15% malignant tumours (YST).
2. SCST (15%) divided among granulosa cell (5%, benign tumour), Leydig cell (5%, benign tumour), Sertoli cell (3%, malignant or benign) and mixed stromal cell (2%). Inhibin B and AMH are very good markers for JGCTs, and testosterone is a very good marker for Leydig tumours in prepubertal boys. It has to be underlined, that in prepubertal boys, JGCT and Leydig tumours are not considered as malignant tumours, whereas this is the case in prepubertal girls.

One of the principal differential diagnoses is paratesticular RMS which may be challenging to differentiate from an intratesticular lesion when the tumour is large.

Post-pubertal group

Testicular tumours account for 8% of all tumours in the age group 15–19 years [38] with an estimated prevalence in Europe of 24.5 cases per million inhabitants [39]. GCTs comprise 95% of malignant tumours arising in the testes in post pubertal male and are categorised into two main histologic subtypes: seminoma and nonseminoma [40]. The majority of these patients are diagnosed with mixed histology non-seminomatous GCTs, followed by seminoma [10, 41]. Predisposing risk factors include family history of testicular tumour, cryptorchidism and Klinefelter’s syndrome [41–43].

According to 2016 World Health Organization classification, post pubertal testicular GCTs are derived from germ cell neoplasia *in situ* (GCNIS) and are clinically and histologically subdivided into seminomas and non-seminomas, the later encompassing somatic and extra-embryonal elements of embryonal carcinoma, yolk sac, choriocarcinoma and teratoma.

Those tumours usually have a low mutational burden and few somatic changes. A specific recurrent genetic marker – an isochromosome of the short arm of chromosome 12 – (i12p) – is overrepresented. However, some type II testicular GCTs, mainly seminomas, appear to lack a 12p gain and have preferential cKIT mutations. Without the occurrence of these mutations, GCNIS will not progress to invasive GCT. Other significant chromosomal aberrations in type II testicular GCTs are the gain of 7, 8, 21 and the loss of chromosomes 1p, 11, 13 and 18

Post pubertal testicular GCTs are typically thought to behave more aggressively than pre-pubertal tumours, especially in paediatric population.

#### Clinical presentation of testicular tumours

The most common presentation (>90%) is a painless scrotal mass, with a history of trauma and hydrocele or hernia in <10%. Examination usually reveals an enlarge testis and occasionally a mass that may be related to the testis parenchyma. A hydrocele is associated with a testicular tumour in 15%–50% of cases.

Patients with Leydig tumour often present with isosexual or heterosexual precocious puberty symptoms due to the production of androgens (testosterone) by the tumour.

On rare instances, the primary testicular lesion is not clinically nor radiologically evident whilst nodal or visceral metastases remain viable. This phenomenon is described as ‘burned-out testicular tumor’ or ‘spontaneously regressed testicular tumor’; these patients present with mass symptoms secondary to retroperitoneal, mediastinal or supraclavicular lymph nodes or visceral metastases from GCTs, in the absence of clinically apparent testicular masses [44–46].

#### Workup of testicular tumours

Lab: AFP, HCG, Inhibin B and testosterone.

Imaging:

Ultrasonography (US): US is the modality of choice for characterising testicular lesions. The detection of testicular neoplasms with US approaches 100%.

In cases where there is a suspicion of malignancy, cross-sectional imaging with CT or MRI of the abdomen, pelvis and chest is mandatory to determine the preoperative stage and plan therapy, since around 20% of yolk-sac tumours are associated with lung metastases.

In post-pubertal males with elevated HCG, the occurrence of choriocarcinoma should be considered. In these patients, brain MRI should be performed in addition to CT or MRI of abdomen, pelvis and chest given the high likelihood of haematogenous metastases to the brain [40, 47, 48].

No testicular biopsies should be performed.

#### Preoperative treatment for testis tumour in pre-pubertal boys

Neoadjuvant chemotherapy is applied to malignant GCT with elevated AFP (above 10,000 UI/mL in the current European protocol) and/or HCG elevation.

#### Surgical procedure for testis tumour in pre-pubertal boys

Today, the orchiectomy, which was usually carried out in the past, appears to be no longer justified in most prepubertal boys due to the high incidence of benign tumours. It has been shown in various studies that organ-sparing surgery in GCTs (teratoma), gonadal stromal tumours (SLCTs and GCTs) and cystic lesions is reliable and safe. In cases with preoperative significantly increased tumour markers, or if no testicular parenchyma is sonographically detectable or if there is any doubt with a paratesticular RMS, orchiectomy must be carried out. The reasons for orchiectomy in prepubertal boys must be well documented.

The surgical procedure is carried through an inguinal incision, with early control of the spermatic cord, prior to delivering the testis. After opening the tunica vaginalis, the gonad is inspected by palpation and/or intraoperative ultrasound.

The tunica albuginea is generously incised over or in line with the tumour. The neoplasm is then enucleated along with a small rim of parenchyma. When there is a doubt on the benign nature or behaviour of the lesion, extemporaneous exam may be done. After the closure of the tunica, the testis is reperfused after unclamping, while awaiting pathological confirmation. If the tumour is huge and not amenable by the inguinal approach, it is less risky to ligate the spermatic cord and to perform a scrotal incision to extract the testis with the mass to avoid tumour rupture.

Testis sparing surgery is critical to preserve the fertility potential and may reduce psychological and cosmetic consequences associated with radical orchiectomy. It is essential to acknowledge the family and then the patient of the possibility to implant a testicular prothesis in the pre-pubertal period.

In prepubertal malignant testicular GCT and SCST, no treatment is indicated after surgery if the resection was complete and if the surgery was performed according to the protocol (considering the level of markers).

#### Surgical procedure for testicular tumour in post-pubertal boys

In post-pubertal testicular tumours, patients with Stage I disease are treated by complete orchiectomy alone, followed by clinical, serum markers and radiological surveillance. The surgical procedure is carried through an inguinal incision, with early control of the spermatic cord, prior to delivering the testis. If the tumour is huge and not amenable by the inguinal approach, it is less risky to ligate the spermatic cord and to perform a scrotal incision to extract the testis with the mass to avoid tumour rupture. Age greater than 10 years, mixed histology and presence of lymphovascular invasion are each associated with relapse [10].

Patients with advanced stage disease will receive chemotherapy. After chemotherapy, patients with persistent en plateau or further marker regression or retroperitoneal lesions larger than 1 cm should undergo resection of all residual radiologic lesions including retroperitoneal lymph nodes associated with complete orchiectomy [49].

#### Prognosis, prognostics and follow-up for testicular tumour

Overall mortality rates are low, with a rate of 1 death per 10 million per year, and survival for prepubertal testicular cancer is about 99% at 5 years [50].

Even in post-pubertal tumours, the 5-year relative survival rate is over 96%, confirming the necessity to include attention to the long-term outcomes of these patients [51].

## References

[1] Ries LAG (1999). Cancer Incidence and Survival among Children and Adolescents: United States SEER Program, 1975–1995.

[2] Poynter JN, Amatruda JF, Ross JA (2010). Trends in incidence and survival of pediatric and adolescent patients with germ cell tumors in the United States, 1975 to 2006. Cancer.

[3] Bonouvrie K, Ten Bosch J van der W, van den Akker M (2020). Klinefelter syndrome and germ cell tumors: review of the literature. Int J Pediatr Endocrinol.

[4] Nagai T, Hasegawa K, Motegi E (2019). Usefulness of imprint cytology of gonadoblastoma with dysgerminoma in a patient with Turner syndrome and a Y chromosome: a case report and literature review. Diagn Cytopathol.

[5] Zhu J, Liu X, Jin H (2011). Swyer syndrome, 46, XY gonadal dysgenesis, a sex reversal disorder with dysgerminoma: a case report and literature review. Clin Exp Obstet Gynecol.

[6] Schneider DT, Calaminus G, Koch S (2004). Epidemiologic analysis of 1,442 children and adolescents registered in the German germ cell tumor protocols. Pediatr Blood Cancer.

[7] Wang Y, Wu Y, Wang L (2017). Analysis of recurrent sacrococcygeal teratoma in children: clinical features, relapse risks, and anorectal functional sequelae. Med Sci Monit Int Med J Exp Clin Res.

[8] Gübel U, Haas RJ, Calaminus G (1990). Treatment of germ cell tumors in children: results of European trials for testicular and non-testicular primary sites. Crit Rev Oncol Hematol.

[9] Schneider DT, Calaminus G, Reinhard H (2000). Primary mediastinal germ cell tumors in children and adolescents: results of the German cooperative protocols MAKEI 83/86, 89, and 96. J Clin Oncol.

[10] Rescorla FJ, Ross JH, Billmire DF (2015). Surveillance after initial surgery for Stage I pediatric and adolescent boys with malignant testicular germ cell tumors: report from the Children’s Oncology Group. J Pediatr Surg.

[11] Azizkhan RG, Dudgeon DL, Buck JR (1985). Life-threatening airway obstruction as a complication to the management of mediastinal masses in children. J Pediatr Surg.

[12] Shamberger RS, Holzman RS, Griscom NT (1991). CT quantitation of tracheal cross-sectional area as a guide to the surgical and anesthetic management of children with anterior mediastinal masses. J Pediatr Surg.

[13] Billmire D, Vinocur C, Rescorla F (2003). Malignant retroperitoneal and abdominal germ cell tumors: an intergroup study. J Pediatr Surg.

[14] Qureshi SS, Kammar P, Kembhavi S (2017). Excision of retroperitoneal germ cell tumor in children: a distinct surgical challenge. J Pediatr Surg.

[15] Bernbeck B, Schneider DT, Bernbeck B (2009). Germ cell tumors of the head and neck: report from the MAKEI Study Group. Pediatr Blood Cancer.

[16] Mauz-Körholz C, Harms D, Calaminus G (2000). Primary chemotherapy and conservative surgery for vaginal yolk-sac tumour. Lancet.

[17] Hermans AJ, Kluivers KB, Janssen LM (2016). Adnexal masses in children, adolescents and women of reproductive age in the Netherlands: a nationwide population-based cohort study. Gynecol Oncol.

[18] Isaacs Jr H (2004). Perinatal (fetal and neonatal) germ cell tumors. J Pediatr Surg.

[19] Lakhoo K (2010). Neonatal teratomas Early Hum Dev.

[20] Elias KM, Tsantoulis P, Tille J (2018). Primordial germ cells as a potential shared cell of origin for mucinous cystic neoplasms of the pancreas and mucinous ovarian tumors. J Pathol.

[21] Faure A, Atkinson J, Bouty A (2016). DICER1 pleuropulmonary blastoma familial tumour predisposition syndrome: what the paediatric urologist needs to know. J Pediatr Urol.

[22] Schultz KAP, Pacheco MC, Yang J (2011). Ovarian sex cord-stromal tumors, pleuropulmonary blastoma and DICER1 mutations: a report from the International Pleuropulmonary Blastoma Registry. Gynecol Oncol.

[23] Schultz KAP, Williams GM, Kamihara J (2018). DICER1 and associated conditions: identification of at-risk individuals and recommended surveillance strategies. Clin Cancer Res.

[24] Young RH, Kozakewich HP, Scully RE (1993). Metastatic ovarian tumors in children: a report of 14 cases and review of the literature. Int J Gynecol Pathol Off J Int Soc Gynecol Pathol.

[25] Cunningham I (2013). The clinical behavior of 124 leukemic ovarian tumors: clues for improving the poor prognosis. Leuk Lymphoma.

[26] Schneider DT, Terenziani M, Cecchetto G (2012). Gonadal and extragonadal germ cell tumors, sex cord stromal and rare gonadal tumors. Rare Tumors in Children and Adolescents.

[27] Cecchetto G (2014). Gonadal germ cell tumors in children and adolescents. J Indian Assoc Pediatr Surg.

[28] Kalfa N, Patte C, Orbach D (2005). A nationwide study of granulosa cell tumors in pre-and postpuberal girls: missed diagnosis of endocrine manifestations worsens prognosis. J Pediatr Endocrinol Metab.

[29] Sarnacki S, Brisse H (2011). Surgery of ovarian tumors in children. Horm Res Paediatr.

[30] Fresneau B, Orbach D, Faure‐Conter C (2015). Sex‐cord stromal tumors in children and teenagers: results of the TGM‐95 study. Pediatr Blood Cancer.

[31] Raffoul L, Capito C, Sarnacki S (2016). Fertility considerations and the pediatric oncology patient. Seminars in Pediatric Surgery.

[32] Taskinen S, Urtane A, Fagerholm R (2014). Metachronous benign ovarian tumors are not uncommon in children. J Pediatr Surg.

[33] Coppes MJ, Rackley R, Kay R (1994). Primary testicular and paratesticular tumors of childhood. Med Pediatr Oncol.

[34] Ross JH, Rybicki L, Kay R (2002). Clinical behavior and a contemporary management algorithm for prepubertal testis tumors: a summary of the Prepubertal Testis Tumor Registry. J Urol.

[35] Pohl HG, Shukla AR, Metcalf PD (2004). Prepubertal testis tumors: actual prevalence rate of histological types. J Urol.

[36] Ahmed HU, Arya M, Muneer A (2010). Testicular and paratesticular tumours in the prepubertal population. Lancet Oncol.

[37] Taskinen S, Fagerholm R, Aronniemi J (2008). Testicular tumors in children and adolescents. J Pediatr Urol.

[38] Ward E, DeSantis C, Robbins A (2014). Childhood and adolescent cancer statistics, 2014. CA Cancer J Clin.

[39] Steliarova-Foucher E, Stiller C, Kaatsch P (2004). Geographical patterns and time trends of cancer incidence and survival among children and adolescents in Europe since the 1970s (the ACCIS project): an epidemiological study. Lancet.

[40] Gilligan T, Lin DW, Aggarwal R (2019). Testicular cancer, version 2.2020, NCCN clinical practice guidelines in oncology. J Natl Compr Canc Netw.

[41] Shaikh F, Murray MJ, Amatruda JF (2016). Paediatric extracranial germ-cell tumours. Lancet Oncol.

[42] Krege S, Beyer J, Souchon R (2008). European consensus conference on diagnosis and treatment of germ cell cancer: a report of the second meeting of the European Germ Cell Cancer Consensus group (EGCCCG): part I. Eur Urol.

[43] Loebenstein M, Thorup J, Cortes D (2019). Cryptorchidism, gonocyte development, and the risks of germ cell malignancy and infertility: a systematic review. J Pediatr Surg.

[44] Fabre E, Jira H, Izard V (2004). ‘Burned‐out’ primary testicular cancer. BJU Int.

[45] Angulo JC, González J, Rodríguez N (2009). Clinicopathological study of regressed testicular tumors (apparent extragonadal germ cell neoplasms). J Urol.

[46] Williamson SR, Delahunt B, Magi‐Galluzzi C (2017). The World Health Organization 2016 classification of testicular germ cell tumours: a review and update from the International Society of Urological Pathology Testis Consultation Panel. Histopathology.

[47] Jiang F, Xiang Y, Feng F-Z (2014). Clinical analysis of 13 males with primary choriocarcinoma and review of the literature. OncoTargets Ther.

[48] Reilley MJ, Pagliaro LC (2015). Testicular choriocarcinoma: a rare variant that requires a unique treatment approach. Curr Oncol Rep.

[49] Kollmannsberger C, Daneshmand S, So A (2010). Management of disseminated nonseminomatous germ cell tumors with risk-based chemotherapy followed by response-guided postchemotherapy surgery. J Clin Oncol.

[50] Alanee S, Shukla A (2009). Paediatric testicular cancer: an updated review of incidence and conditional survival from the Surveillance, Epidemiology and End Results database. BJU Int.

[51] Hayes-Lattin B, Nichols CR (2009). Testicular cancer: a prototypic tumor of young adults. Seminars in Oncology.

# Discussion: Thoracic tumours

**Published:** 2022

**Authors:** Jaime Shalkow, Robert C Shamberger, Ivan Dario Molina Ramirez, Federia De Corti, Andrew J Murphy

## Background

Thoracic tumours represent a challenge for the paediatric surgeon. They encompass a diverse and heterogeneous group of rare neoplasms with varied pathology, location, presentation, biological behaviour and response to treatment and prognosis. Two thirds of thoracic tumours in children are malignant. Most lung tumours are metastatic (5:1). Primary lung tumours in children and adolescents are exceptionally rare, but 76% of them are malignant. The surgeon treating paediatric patients with thoracic tumours must possess a solid understanding of the three-dimensional anatomy of the region, and the physiology of its contained structures. Tumour excision is particularly challenging due to tumour involvement of several critical structures. There is no standardised approach or surgical protocols, thus patient selection, risk assessment, knowledge of different surgical approaches and tracking perioperative outcomes are mandatory.

## Tumours of the Chest Wall

Tumours of the chest wall are rare in the paediatric population. Only 1.8% of the solid childhood tumours admitted to St. Jude Children’s Research Hospital were in the thorax, but up to two-thirds of them are malignant [1]. The majority arise from the bony structures of the chest wall (55%), as opposed to soft tissue (45%) [1–3]. In a summary of seven reported series, most malignant tumours were Ewing sarcoma (56%) followed by rhabdomyosarcoma (25%) and smaller numbers of osteosarcoma, fibrosarcoma, chondrosarcoma and lymphoma [4] (please also refer to *Surgery for Lymphoma and Rhabdomyosarcoma and Non-Rhabdomyosarcoma Soft-Tissue Sarcoma Guidelines*).

### Presentation

Masses of the chest wall most frequently present with respiratory symptoms or pain, with the latter being the most frequent in malignant lesions [5]. For some unknown reason, most tumours grow into the pleural cavity with limited external growth, hence the greater frequency of respiratory symptoms than presentation with a palpable mass. In infants and young children, the benign lesions are often found incidentally by caregivers, while older children and young adults with malignant lesions often present with larger masses and respiratory symptoms. The tissue of origin is generally mesenchymal, regardless of whether the tumours are malignant or benign. Hence, sarcomas are the most common malignant tumours, while carcinomas are almost nonexistent. The symptoms of respiratory compromise or dysfunction – tachypnoea, hypoxia, cough, dyspnoea on exertion – may have been present for quite a while before the adolescents present for medical advice. Symptoms arise from pulmonary parenchymal compression by the mass and/or from a secondary pleural effusion.

### Evaluation

Pulmonary function tests may be indicated prior to proceeding with any intervention based on respiratory symptoms. The initial imaging studies are frequently posterior-anterior and lateral chest radiographs to evaluate the respiratory symptoms or ‘bump’ on the chest. They often reveal the location, size, presence of calcifications and the osseous involvement of the mass as well as the presence of pulmonary parenchymal disease and a pleural effusion. An ultrasonogram will define the echo features of the mass (solid versus cystic, degree of homogeneity and vascularity). Axial imaging (computed tomography (CT) or magnetic resonance imaging (MRI)) will best demonstrate the anatomic relations of the mass to other mediastinal structures and the chest wall. The advantages of CT reside in its ability to clearly define the lung parenchyma and the presence of metastatic lesions. It is also a rapid technique requiring minimal to no sedation, even in the youngest of patients. The benefits of MRI versus CT include better definition of the soft tissue components, as well as enhanced evaluation of the osseous and neural structures to determine the extent of central or peripheral nerve involvement and/or the presence of skip lesions or metastases. It also avoids the radiation exposure of the CT scan [6]. Unfortunately, this technique is time consuming and generally requires sedation or even general anaesthesia to obtain optimal studies and it may not be available in resource limited settings. Motion artefact from the heart and lungs can also interfere with this technique limiting its utility, but this obstacle is being overcome with the use of cardiac-gated, respiratory-triggered protocols. It is important to recognise that diagnosis cannot be established by radiographic studies.

Additional imaging studies may be required to assess the presence of metastases in malignant lesions (brain and abdominal CT, bone scan, positron emission tomogram (PET) scan). Recent reports have suggested that the combination of PET and CT scans yields more accurate data in assessing the primary tumour, local and regional lymph node basins, evidence of recurrence or metastases and for response to ongoing therapies [7]. However, PET-CT is suboptimal for detecting lung metastases when compared with high-resolution CT-Scan [8]. Because of the propensity for Ewing sarcoma and rhabdomyosarcoma to metastasise to bone marrow, these patients should also undergo bone marrow biopsy and aspiration as part of their diagnostic and staging workup. For patients with primitive neuroectodermal tumor (PNET)/Ewing sarcoma, lactate dehydrogenase elevation is a prognostic marker, since marked elevation is found in patients with metastatic disease. Once initial studies have been performed, biopsy is required to define future therapy.

### Indications and principles of biopsy

Biopsy options include small or large specimen approaches. It is imperative to know the diagnosis of chest wall tumours before proceeding to resection. Some benign lesions in infants and young children will spontaneously regress and in malignant lesions, neoadjuvant chemotherapy is often the best initial therapy. Many of these lesions can be diagnosed with a 14-gauge core needle biopsy which provides adequate material for assessing the molecular biology of the tumour as well as its histology [9, 10]. It is important to avoid contamination of the pleural cavity when obtaining a biopsy as this would require radiotherapy to the hemithorax in Ewing sarcoma. Most of these lesions are of adequate size that an image-guided percutaneous co-axial core needle biopsy can be obtained readily without pleural contamination. Ewing sarcomas are also quite vascular and incisional biopsies pose a significant risk of haemorrhage and contamination of the superficial tissues requiring wider resection at the time of the definitive procedure. Placing the incision in-line with any future resection is of paramount importance, regardless of the technique utilised. Once a diagnosis is confirmed, disease-specific treatment algorithms may be initiated.

There is an additional diagnostic issue in patients who present with a pleural effusion which must be addressed prior to initiation of neoadjuvant chemotherapy. The effusion may be reactive or malignant. In the latter situation in patients with Ewing’s sarcoma and rhabdomyosarcoma, radiotherapy is often recommended if an effusion was present. While the dose is significantly lower than required for treatment of the primary lesion, it still carries significant morbidity. Hence, simple aspiration of the effusion if the tumour is malignant will help resolve the question later regarding the need for radiation to the entire hemithorax.

### Surgery

Though treatment regimens are tumour-specific, there are certain general principles that apply. For malignant lesions, multimodal therapy is still the accepted paradigm for most cases. Neoadjuvant chemotherapy can reduce the size of the mass, particularly for Ewing sarcoma, the most frequent tumour of the chest wall; but also, for osteosarcoma and rhabdomyosarcoma. It has been clearly demonstrated that in Ewing sarcoma, neoadjuvant chemotherapy will increase the number of patients in whom a complete surgical resection can be obtained [11]. This is critical because it will decrease the number of patients in whom high-dose radiotherapy is required, and avoid the morbid complications of pulmonary fibrosis, cardiomyopathy and subsequent malignant tumours.

When planning operative resection of the chest wall mass, posterior tumours can exhibit spinal invasion or require rib disarticulation at the facet joint to achieve a negative margin. Involvement of a surgeon with spine expertise should therefore be considered. Even without direct spinal involvement, excessive traction or poor haemostasis during rib disarticulation can lead to spinal haematoma and cord compression. Tumours involving the first rib may also benefit from neurosurgical expertise due to proximity to the brachial plexus.

Simple extirpation is the rule with benign entities. For malignant tumours, the most important concept to emphasise is the need for complete resection with negative pathologic margins to decrease the risk of recurrence and need for subsequent therapy. It is generally accepted that a 1 cm margin is required. But again, the desire is to avoid any use of radiotherapy for tumours in the chest due to its increased morbidity to the heart and lungs. The need for resection of the overlying skin and musculofascial layers is determined by involvement of these layers by the tumour. If they were not involved at presentation, they can be preserved to facilitate closure of the wound. If the tumour is not palpable externally, an incision between the ribs should be made which is clearly anterior or posterior to the tumour based on radiographic imaging. This will avoid disruption of the tumour. Then, by digital exam or thoracoscopic guidance, it can be determined which ribs will require resection and where they should be divided. It is important in a malignant tumour, such as Ewing sarcoma, that all areas of dense fibrotic scar following neoadjuvant chemotherapy are removed as they may harbour microscopic areas of tumour and produce a positive resection margin. In malignant tumours, only the involved portion of the rib must be removed, not the entire rib. Extension of the tumour within the medullary canal or a ‘skipped metastasis’ on the initial scans at presentation will require resection of that segment of the rib with a 1 cm margin. When adhesions are found between the tumour and the parenchyma of the lung or diaphragm, a wedge of the adherent lung or a segment of the diaphragm should be resected with the tumour. This avoids disruption of the capsule of the tumour which would produce a positive pathologic margin. In most cases, primary repair of the diaphragm is feasible even following resection of the involved area. If primary repair would result in undue tension, a prosthetic patch (PTFE/Gore-tex) can be utilised for diaphragmatic reconstruction. Pericardium might also require *en-bloc* resection in selected cases. The defect should be repaired with fenestrated prosthetic material if it is large enough to allow herniation of the heart. Fenestration is critical to prevent accumulation of pericardial fluid and cardiac tamponade.

Surgical resection also requires wound reconstruction. Reconstructive options include autologous or prosthetic reconstruction. Posterior chest wall defects or reconstructions can be covered by autologous latissimus dorsi flaps, while anterior defects can be covered by pectoralis major flaps. Large defects (greater than 5 cm or involving three or more ribs) are often reconstructed with a prosthetic patch, but this is dependent upon the site of the resection. Posterior and superior lesions where the defect will be buttressed by the scapula often do not require the use of prosthetic grafts. In older children and adolescents, flexible prosthetic materials such as Gore-tex (WL Gore & Associates), Marlex mesh (C.R. Bard/Davol) and Prolene mesh (Ethicon) can be utilised. Some surgeons use a rigid prosthesis with methyl methacrylate configured to the contour of the chest wall, but in active teenagers, a pliable graft is probably best although some indentation of the chest wall will invariably occur. In younger school aged children in whom these tumours are fortunately rare, pedicle flaps, biologic graft or absorbable mesh such as Vicryl (Ethicon) can be used, as they will be absorbed and replaced with fibrous scar which can allow some growth of the chest wall with time and decrease the severity of scoliosis. Newer titanium rib replacement systems are available and could be of benefit for adolescents and young adults. Unfortunately, these systems are not readily accessible in the limited-resource setting.

It was thought that the material used to reconstruct the chest wall would render postoperative radiation for local control more toxic. However, it is now well established that those refraction artefacts are easily controlled with adequate pre-radiation planning and beam corrections [12, 13].

As mentioned, R-0 resections are paramount for cure and resectability should be thoroughly assessed preoperatively. However, if the patient’s safety is considered endangered during the procedure, attempts at resection should cease.

### Postoperative considerations

Following surgery, chemotherapy is generally held until the early postoperative period is completed and there are no early complications with bleeding or infection. Whether postoperative radiotherapy is required will be determined by the completeness of the resection. Resection is generally avoided if a complete resection does not appear feasible as it is best to avoid patients facing the potential complications of both surgery and radiotherapy.

Long-term survivors of chest wall resection for sarcoma, particularly when combined with chest wall irradiation, have an increased incidence of scoliosis, impaired pulmonary function and worse self-reported health outcomes including daily functional impairment and cancer-related pain [14]. Patients who undergo chest wall resection during periods of rapid growth have posterior chest wall tumours, and who undergo resection of two or more ribs are the most likely to develop scoliosis and should be monitored for this long-term effect of surgery [15]. A multi-institutional long-term follow-up study of 175 patients who underwent surgery for chest wall sarcoma showed that 13% developed scoliosis and 5% required corrective spine surgery [16].

## Mediastinal Tumours

Mass lesions of the mediastinum in children are rare, but they represent the most common intrathoracic tumours in this age group. They have multiple origins and may appear at any age throughout infancy, childhood and adolescence. The mass may be cystic or solid, and of either congenital or neoplastic origin. Sixty-five to 80% of mediastinal tumours in children are malignant, with over 40% occurring in patients younger than 2 years of age.

### Presentation

The symptoms produced by a mediastinal mass are almost as diverse as the underlying pathology of these lesions, but most symptoms are due to the ‘mass effect’ of the lesion which may compress the airway, vasculature, oesophagus or the lung. Occasionally, they present with pain resulting from inflammation produced by infection or perforation of a cyst. Invasion of the chest wall by a malignant tumour will also produce pain. Many mediastinal lesions, in fact, are incidentally found by serendipity as a radiographic abnormality on a study obtained for symptoms unrelated to the mass. Respiratory symptoms of expiratory stridor, cough, dyspnoea, orthopnoea, tachypnoea or hypoxia require urgent investigation. Cystic or solid lesions located at the carina may produce major airway obstruction. Lesions at this site are often obscured by the normal mediastinal shadow and may not be apparent on the anterior–posterior or lateral chest radiographs. Orthopnoea and venous engorgement from superior vena cava syndrome occur with extensive involvement of the anterior mediastinum and are harbingers of respiratory obstruction upon induction of a general anaesthetic (Please refer to *Surgery for Lymphomas Guidelines*). Less frequently, dysphagia from pressure on the oesophagus is the presenting symptom. Neurologic symptoms from spinal cord compression or Horner’s syndrome may occur with neurogenic tumours arising in the posterior mediastinum.

Some tumour-specific symptoms can be distinguished. These include fever, night sweats and weight loss in lymphoma, myasthenia gravis in thymoma, virilisation in some germ-cell tumours, high blood pressure in paragangliomas and paraneoplastic syndromes in neuroblastoma, such as Kinsbourne syndrome (opsoclonus-myoclonus) and Verner–Morrison syndrome (watery diarrhoea with hypokalaemia due to vasoactive intestinal peptide (VIP) production).

### Evaluation

Most lesions in the anterior and middle mediastinum will require biopsy due to the wide variety of solid tumours which can occur at this site and their distinct treatment protocols. Resection is rarely required for Hodgkin’s lymphoma and non-Hodgkin’s lymphoma while teratomas and thymomas will generally require resection. Knowledge of the tumour type is thus critical prior to surgical resection.

Anterior mediastinal tumours comprise 44% of mediastinal masses, 80% of them being malignant. They are often recognised as the four terrible ‘T´s’, in order of frequency:

- T-cell lymphoma (Non-Hodgkin lymphoma)
- Teratoma
- Thymoma
- Thyroid

Rare lesions in the anterior mediastinum include parathyroid tumours, Langerhans histiocytosis, sarcoidosis, Castleman and Rosai–Dorfman disease. Twenty percent of mediastinal tumours are in the middle mediastinum. They are most frequently lymphocytic in origin (Hodgkin and Non-Hodgkin lymphoma) – (Please refer to Surgery for Lymphomas Guidelines), as well as pericardial and myocardial tumours.

### Anaesthesia in Patients with an Anterior Mediastinal Mass

Patients presenting with an anterior mediastinal mass pose a therapeutic challenge to the anaesthesiologist and the surgeon. Correct diagnosis of the tumour will provide the greatest chance of cure. There are, however, a plethora of reports in the anaesthesia and surgical literature of patients with an anterior mediastinal mass suffering respiratory collapse upon the induction of general anaesthesia. This occurrence has led to an understandable reluctance among anaesthesiologists to use general anaesthesia in this setting. Respiratory symptoms are a poor index of the risk of respiratory collapse with the clear exception of orthopnoea and Superior vena cava syndrome which suggest a high risk that respiratory collapse will occur [17]. An initial attempt to establish the radiographic parameters that would correlate with respiratory collapse upon induction of general anaesthesia involved a retrospective evaluation of 74 adults with Hodgkin’s disease [18]. In this study, the authors used the ratio of the transverse diameter of the mediastinal mass and the transverse diameter of the chest as the critical parameter. They found the incidence of respiratory collapse was 2.1% with a mediastinal mass less than 31% of the transverse diameter compared with 10.5% for a mass with a ratio of 32%–44% and 33.3% for a mass with a ratio greater than 45%. Similar results were reported by others [19]. These parameters obviously lacked specificity to identify patients at risk.

Griscom [20] demonstrated that CT scan can accurately determine the tracheal dimensions in children and adolescents and he established the normative values in children. Azizkhan *et al* [21] first used this methodology in reviewing 50 children with an anterior mediastinal mass. They found that 13 patients in their cohort had ‘marked’ tracheal compression that was defined as a tracheal cross-sectional area of less than 66% of predicted. When general anaesthesia was induced in 8 of these 13 patients, total airway obstruction occurred in 5, all of whom had 50% or less of the predicted tracheal area [21]. It was suggested, based on these findings, that general anaesthesia should be avoided in children with less than 66% of the predicted tracheal cross-sectional area on CT scan.

A prospective study used Griscom’s tracheal area measures to determine risk of anaesthesia in 31 children with a mediastinal mass who were evaluated with both CT scan and pulmonary function tests [22]. While the use of pulmonary function tests had been proposed by multiple investigators as a method to assess anaesthetic risk, little evaluation of this modality had been performed. The most pronounced perturbation of pulmonary function tests caused by airway obstruction due to an anterior mediastinal mass is marked reduction in the maximum expiratory flow rate. Hence, in this prospective study, the peak expiratory flow rate (PEFR) was utilised as a critical factor. Patients with either a PEFR of less than 50% of predicted or a tracheal area of less than 50% of predicted received local anaesthesia for their procedures. General endotracheal anaesthesia was used only in patients with greater than 50% of predicted for both parameters. All patients in this study did well when general anaesthesia was applied. Further analysis of this cohort revealed that patients with a tracheal area greater than 50%, but a PEFR less than 50% had one of two characteristics; either a very low total lung capacity (38% and 55% of predicted) resulting from the massive size of the tumour or moderate to severe bronchial narrowing by qualitative estimation (four of these five patients). Hence, if able to obtain only one study, the PEFR is probably the most reliable and is most readily available in resource limited settings as it can be obtained with a simple hand-held spirometer.

A recent study correlated the risk of respiratory collapse with the ‘standardized tumor volume’ which was determined by calculating the tumour volume (volume as an ellipsoid using the three tumour dimensions) and dividing it by the patient’s height [23]. In this retrospective study, they found good correlation between the ‘standardized tumor volume’ and respiratory collapse upon induction of anaesthesia. Using a cut-off value of 2.5, the sensitivity and specificity for predicting respiratory collapse upon induction of general anaesthesia were both 0.86. This assessment has not yet been evaluated in a prospective fashion.

According to the above parameters, patients who are determined to be at high risk of respiratory collapse with induction of general anaesthesia (orthopnoea, greater than 50% reduction of tracheal cross-sectional diameter, any mainstem bronchial compression or greater than 50% reduction of PEFR from predicted) should be managed according to an algorithm which includes multiple non-invasive attempts to obtain a diagnosis (Figure 1) [24, 25].

Many of these non-invasive measures can be performed in parallel to expedite diagnosis and treatment. First, peripheral blood can be submitted for flow cytometry, which can sometimes be diagnostic for haematological malignancies such as lymphoma. Second, bone marrow aspiration and biopsy can be performed under local anaesthesia and can yield diagnostic information for patients with lymphoma, neuroblastoma and some other malignancies. Third, pleural effusions can be sampled by thoracentesis using anatomic landmarks or ultrasound guidance under local anaesthesia [26]. Patients with lymphoblastic lymphoma have a higher incidence of an associated pleural effusion (71%) than children with Hodgkin’s (11.4%). Cells in the effusion can be assayed by immunocytochemical studies as well as by cytogenetic evaluation, immunophenotyping and cytology. Then, for patients that have palpable lymph nodes or lymph nodes visible by ultrasound outside the chest, a lymph node biopsy can be performed under local anaesthesia. Finally, in children with significant respiratory compromise, no pleural effusion and no lymph nodes accessible outside the chest, either a percutaneous image-guided needle biopsy or an open anterior thoracotomy (Chamberlain procedure) can be performed successfully under local anaesthesia (Please refer to *Surgery for Lymphomas Guidelines*). Exposure to the anterior mediastinum can be obtained with this procedure by removal of a costal cartilage preserving the perichondrium. To perform this biopsy, children should be seated in a semi-upright position and be spontaneously breathing to maximise their pulmonary function. This position will also decrease venous congestion if present. Spontaneous ventilation minimises collapse of the trachea by the negative pressure exerted by the chest wall. Following these guidelines for the use of general and local anaesthesia and the biopsy techniques discussed, a biopsy can be obtained safely in essentially all children and adolescents with an anterior mediastinal mass.

If these guidelines do not result in successful diagnosis, patients can be treated in a preliminary fashion with either radiation therapy or steroids/chemotherapy for the most likely diagnosis based on the clinical findings. This approach often prevents accurate diagnosis or immunophenotyping of the tumour due to the rapid response to treatment and should therefore be used only as a last resort [27].

## Surgery

Management of mediastinal masses is determined by the presumed diagnosis. Cystic lesions in the anterior mediastinum are generally resected. Acute enlargement in thymic cysts has been noted following a viral respiratory illness. Lymphatic malformations may involve the mediastinum with their predominant component in the cervical–facial area. Isolated mediastinal involvement is seen infrequently. Pericardial cysts are the most innocent of these lesions and if well demonstrated on scans and radiographs often are simply followed because they rarely increase in size and are unlikely to compress any vital structures.

The solid lesions require establishment of a histopathologic diagnosis. The most common solid tumour in the anterior mediastinum is Hodgkin lymphoma followed by non-Hodgkin lymphoma. Primary treatment of these tumours is chemotherapy often in conjunction with radiotherapy; the surgeon’s role is to establish the diagnosis (Please refer to *Surgery for Lymphomas Guidelines*). Potential germ-cell tumours which occur in the anterior mediastinum require evaluation of tumour markers to detect malignancy (Please refer to *Germ Cell Tumour Guidelines*). These include beta-human chorionic gonadotropin to detect choriocarcinoma elements, or alfa-fetoprotein for endodermal sinus tumours [28]. The primary treatment for malignant germ cell tumours of the mediastinum is also chemotherapy, with surgical resection of residual masses after treatment [28]. Teratomas or dermoids are the only neoplastic lesions which require primary resection as they may become secondarily infected or undergo malignant degeneration if they contain immature elements. Retrosternal thyroid goitres are generally resected through the neck. These latter tumours and substernal extension of cervical lymphatic malformations (cystic hygromas) can often be quite readily removed through a suprasternal incision; with progressive retraction and dissection one can remove quite sizable retrosternal masses originating in the neck.

Thymomas and thymic carcinomas are the rarest of anterior mediastinal tumours and they account for less than 1% of all mediastinal tumours in children [29]. Of note, 30%–50% of cases are asymptomatic and found incidentally on radiographic studies obtained for unrelated symptoms [30]. A thymoma-related paraneoplastic syndrome occurs in 30%–65% of patients, myasthenia gravis being most common, but myocarditis, polymyositis, lupus erythematosus, rheumatoid arthritis, thyroiditis, Sjögren syndrome, aplastic and haemolytic anaemia, Addison disease, Cushing syndrome, scleroderma, nephrotic syndrome, radiculopathy and others have been described. CT and MRI scans offer detailed information to facilitate the differential diagnosis and surgical planning. It must be recognised that children may develop rebound thymic hyperplasia following chemotherapy which can mimic a tumour.

Surgery is the mainstay of therapy for tumours of the thymus. Core needle biopsy is often insufficient for definitive diagnosis. Diverse minimally invasive techniques are available for adequate tissue sampling. Lymphoma often requires surgical biopsy to establish its diagnosis but is the only anterior mediastinal mass in which resection plays no role in treatment. Radiotherapy may be given in combination with chemotherapy for some lymphomas as well as for other unresectable or recurrent tumours, but dose-limitation in children deserves consideration. International cooperative studies may improve evaluation of treatment modalities for these rare tumours.

Bronchogenic cysts and oesophageal duplications arise in the middle and posterior mediastinum. They develop in the embryo during separation of the aerodigestive systems. Bronchogenic cysts are generally lined by respiratory epithelium and oesophageal duplications by intestinal mucosa, but ectopic mucosa may be present in both lesions. These should be resected because of their potential for growth with progressive accumulation of secretions. They can also become secondarily infected or develop malignancy. Lesions with gastric mucosa can erode into the bronchus, oesophagus or pleural cavity. Enteric duplication cysts usually share a common muscular wall with the oesophagus and have the endoscopic appearance of extrinsic compression on the oesophagus with normal overlying mucosa. Two thirds occur in the lower oesophagus and one-third occur in the upper or middle portion [31]. The most common location for an oesophageal duplication is the inferior right posterior oesophagus. Oesophageal duplications are resected using a minimally invasive or open enucleation technique. The muscular layer of the oesophagus can be reapproximated at the conclusion of the enucleation to prevent pseudodiverticulum formation [32]. For bronchogenic cysts that are intimately associated with the airway or share a common wall with the tracheo-bronchial tree, and would require formal pulmonary resection for complete removal, a rim of the cyst wall can be left on critical airway structures and a mucosectomy can be performed. Failure to remove all the cyst mucosa can result in reaccumulation of the cyst or persistent secretions with development of local infection or symptoms [33]. Resection of cystic lesions of the mediastinum can often be accomplished thoracoscopically [34].

Masses of the posterior mediastinum in children are seen in 36% of cases, two thirds of them being malignant [35]. These are usually represented by neurogenic tumours, such as neuroblastoma, ganglioneuroma and neurofibroma (Please refer to *Neuroblastoma Guidelines*). These tumours generally should be resected. For thoracic neuroblastoma, this is a major component of its treatment. The requirement for radiation therapy or chemotherapy will depend on the age of the child, the specific histologic type of the tumour (neuroblastoma, ganglioneuroblastoma or ganglioneuroma), the presence of metastatic disease and the molecular biology of the tumour, particularly amplification of the *MYCN* oncogene or normal ploidy, both of which predict an aggressive tumour, although unfavourable lesions are more common in the abdomen than in the thorax. Ganglioneuroma and ganglioneuroblastoma, while benign tumours, may grow locally, may erode the ribs and may extend into the spinal canal producing neurologic symptoms. While these benign lesions are often identified when asymptomatic, resection generally is recommended to establish diagnosis and prevent local extension (Please refer to *Neuroblastoma Guidelines*).

Thoracic neuroblastomas may exhibit IDRFs that require specific surgical considerations. IDRFs relevant to thoracic resections include encasement of the carotid artery, vertebral artery, internal jugular vein, subclavian artery/vein, aorta or superior vena cava [36]. Encasement of arterial vessels is defined as contact with greater than 50% of the circumference of an artery and for venous structures it involves collapse with failure to visualise the lumen. Tracheal or mainstem bronchial compression also constitutes IDRFs. Spinal canal invasion is the most common IDRF present in thoracic neuroblastoma. IDRFs upstage the tumour from L1 to L2 according to the International Neuroblastoma Risk Group radiographic staging system and this affects risk stratification and the decision to use neoadjuvant chemotherapy [37]. In cases of L2 neurogenic tumours with extension of the tumour into the spinal canal, the spinal component should generally be resected initially with the thoracic resection to follow. Spinal canal invasion as an IDRF is defined as greater than one third of the spinal canal being involved on axial cross-sectional imaging, the obliteration of the perimedullary leptomeningeal space or signal change in the adjacent spinal cord on MRI [36]. More limited involvement of the neural foramina and vertebral canal without these specific features can be present, but this would not require surgical resection of the spinal component. Swelling of the spinal component following resection of the thoracic portion alone has produced neurologic injury to the spinal cord. The thoracic tumour component should be cut flush at the level of the neural foramina instead of dissecting into the neural foramina, which can result in intraspinal haematoma.

Many thoracic neurogenic tumours can be safely and completely resected using minimally invasive techniques. Phelps *et al* [38] found that a tumour volume of less than 100 mL and the absence of IDRF were associated with a neuroblastomas which was amenable to a minimally invasive technique without compromise of oncologic integrity (please refer to the *Minimal Invasive Surgery Guidelines*). Patients’ families should be made aware that resection of apical neurogenic tumours can be expected to yield a persistent Horner syndrome (ptosis, myosis, anhidrosis) postoperatively due to tumour origin or involvement of the superior cervical ganglion. Inferior thoracic neurogenic tumours involving the costovertebral junction from T9-T12 are also considered to have an IDRF because they may involve the artery of Adamkiewicz, the thoracolumbar segmental artery that supplies the spinal cord in this location, and therefore CT or conventional arteriography could be considered for surgical planning. Some surgeons choose to utilise intraoperative spinal monitoring for tumours in this location. Cervicothoracic and thoracoabdominal neuroblastomas are considered to have IDRFs. Cervicothoracic tumours may require a trap-door type incision for optimal exposure and thoracoabdominal tumours could require a thoracoabdominal incision with division and eventual repair of the diaphragm to achieve optimal exposure. Most localised (L1 or L2) thoracic neuroblastomas are low- or intermediate-risk tumours with a very favourable long-term prognosis if a partial response (>50% tumour volume reduction) is achieved either with systemic therapy or surgical resection [39, 40]. Therefore, patient safety should always take precedence over completeness of resection and small portions of tumour can be safely left unresected in many of these patients to spare critical vessels or nerves (Please refer to *Neuroblastoma Guidelines*).

Thoracic paraganglioma (extra-adrenal pheochromocytoma) should be removed to control the systemic manifestations of neuropeptide production. The patient should be well prepared for surgery with alpha- and beta-blocking agents and volume repletion. Because patients are often diagnosed due to systemic manifestations (hypertension, headaches, anxiety attacks) or genetic predisposition to paragangliomas, the tumours are usually small in volume and are usually amenable to minimally invasive resection. If possible, patients with a diagnosis of paraganglioma should undergo genetic testing, as greater than 50% are associated with a germline tumour predisposition [41].

## Primary Pulmonary Tumours

Most pulmonary neoplasms in the paediatric age group are metastatic rather than primary lesions, with a ratio of 5:1 [42]. Although primary lung tumours in children are exceedingly rare, it is important to recognise that up to 76% of them are malignant [43].

## Pleuropulmonary Blastoma (PPB)

PPB is the most common primary malignancy of the lungs in childhood [44]. It is a rare and aggressive tumour that originates from the mesenchymal blastema of the lung, pleura or mediastinum [45, 46]. It is analogous to other disontogenic tumours such as nephroblastoma, neuroblastoma and hepatoblastoma. Histologically, PPB has the appearance of multiseptated cysts with scant primitive small round blue cells with rhabdomyoblastic differentiation in the interstitial spaces between septa [47]. Advanced cases progress from a fundamentally cystic to a more solid histology with dense blastematous or sarcomatous cells with anaplastic features. Grossly, advanced cases of PPB demonstrate loss of cystic architecture and exhibit aggressive pleural spread. A definitive diagnosis is notoriously difficult to make based on histology alone, and thus molecular genetic approaches have greatly facilitated the proper identification and treatment of this disease [48].

In 2009, the micro-RNA processing gene *DICER1* was identified as the first known genetic cause for PPB by Hill et al [49] as a heterozygous germline mutation. A detailed understanding of the clinical outcomes and molecular pathogenesis of this rare disease was achieved by the International Pleuropulmonary Blastoma/DICER1 registry (https://www.ppbregistry.org/), which has enrolled over 500 patients in the last 20 years [45, 50]. All PPB cases should be enrolled in this registry and undergo central pathology review. Confirmation of a diagnosis of PPB is greatly assisted by *DICER1* germline testing. 70%–80% of children with PPB have pathogenic, loss-of-function germline variants of *DICER1* which can be inherited in an autosomal dominant fashion. The remainder of patients without *DICER1* germline mutations exhibit biallelic somatic loss of *DICER1* in the tumour [51]. PPB is often the earliest manifestation of the *DICER1* Familial Tumour Predisposition Syndrome, which increases risk for a multitude of malignant and benign tumours most commonly in the lungs, kidneys, ovaries and thyroid. This syndrome has specific screening guidelines and management recommendations which should be followed once a diagnosis is confirmed [52].

Patients with *one or more of the following major criteria* should undergo germline *DICER1* testing: PPB, paediatric lung cysts (if multi-septated, multiple or bilateral), thoracic embryonal rhabdomyosarcoma, cystic nephroma, genitourinary sarcomas, ovarian Sertoli–Leydig cell tumour, gynandroblastoma, uterine cervical or ovarian rhabdomyosarcoma, genitourinary/gynaecologic neuroendocrine tumours, multinodular goitre or thyroid cancer in two or more first-degree relatives, childhood onset multinodular goitre or differentiated thyroid cancer, ciliary body medulloepithelioma, nasal chondromesenchymal hamartoma, pineoblastoma and pituitary blastoma. In addition, a patient should undergo *DICER1* germline testing for any *two or more of the following minor criteria*: lung cysts in adults, renal cysts, Wilms tumour, multinodular goitre or differentiated thyroid cancer, embryonal rhabdomyosarcoma other than thoracic or gynaecologic, poorly differentiated neuroendocrine tumour, undifferentiated sarcoma and macrocephaly. Germline testing should also be considered for any other childhood cancer in constellation with any single minor criteria. If a patient has a pathogenic *DICER1* variant, focused genetic evaluation should be performed for that variant in all first-degree relatives given the autosomal dominant pattern of inheritance. Enrollment in a cancer predisposition or genetics clinic is strongly recommended for individuals with pathogenic variants in *DICER1* [52].

PPBs are classified into three types as per Dehner *et al* [44, 50]:

Type I: Cystic

Type II: Cystic and solid

Type III: Solid

Type Ir (involuted/regressed type I): A fourth type of ‘regressed’ PPB (type Ir) retains the mulitcystic architecture of type I PPB, but has either lost or not developed the underlying primitive small round blue cell/rhabdomyoblastic component.

A direct relationship between the histologic type, the age at diagnosis and the aggressiveness of PPB is suspected. Types I are found in younger patients (0–2 years of age), tend to be more localised, smaller in size and are often more readily resectable. Type I PPB has a better long-term prognosis, with a 5-year event free survival of 82% and an overall survival of 91% [45]. Progression between types is well documented. Type I PPB cannot metastasise without first progressing to a Type II or III PPB. Types II and III PPB can metastasise [45].

### Presentation

PPB occurs primarily in children under 4 years of age, usually presenting with cough, dyspnoea, chest pain, recurrent ‘pneumonia’ refractory to antibiotics and haemoptysis. Pleural effusion and pneumothorax have also been described. Most cases occur in the right hemithorax. The most frequent sites of metastasis are liver, brain and spinal cord. Recurrence is frequent and mortality is 40% with metastatic disease [45].

### Diagnosis

Diagnosis is made with CT-scan of the chest, bronchoscopy and often biopsy. Biopsy can be avoided for smaller lesions, especially for type I (pure cystic) lesions in which non-morbid resection (pulmonary wedge resection or lobectomy) would be anticipated to achieve negative margins. These soft, friable and necrotic tumours can be confused with empyema on initial radiographic imaging. A diagnostic challenge for paediatric surgeons is the differentiation of PPB from congenital pulmonary airway malformations (CPAMs). Type 4 CPAM may have a similar presentation and radiographic appearance to PPB. CPAMs are usually resected due to the potential for infection or, very rarely, malignant degeneration [53]. While case reports have detailed malignant degeneration of CPAMs into sarcomas or other tumour types, CPAM and PPB are currently regarded as distinct entities [53]. However, asymptomatic CPAMs are sometimes observed rather than resected in many centres [54, 55]. It is imperative to confidently differentiate CPAM from PPB if observation without resection is planned. A recent study developed a clinical algorithm for the management of cystic pulmonary abnormalities in children with an emphasis on differentiating CPAM from PPB. The clinical characteristic most strongly associated with CPAM compared to PPB was prenatal detection. Radiographic features associated with a high likelihood of CPAM included any hyperinflated region of lung or presence of a systemic feeding vessel. Radiographic features associated with a high likelihood of PPB included multilobar or bilateral abnormalities, complex cysts and presence of mediastinal shift. Also, *DICER1* screening was recommended for all patients with cystic pulmonary lesions who were originally intended to be observed without resection [56, 57].

PPB can exhibit great vessel or cardiac extension, and thus an echocardiogram is indicated for advanced cases. In addition, endobronchial extension has been reported and therefore bronchoscopy should be considered prior to surgical resection [58].

### Treatment

Primary surgical resection is a reasonable option when the lesions are small (<10 cm) and when complete, non-morbid surgical resection (pulmonary wedge resection or lobectomy) with negative margins can be anticipated. This is most common in Type I PPB. Up-front resection should also be considered when the diagnosis is not clear between a PPB or a congenital pulmonary malformation. Surgical resection alone can be curative for completely resected Type I PPB with negative margins and no intraoperative tumour spill. For larger lesions (>10 cm), most Type II or II PPB, lesions with extensive pleural involvement, or when radical, morbid resection (pneumonectomy, extrapleural pneumonectomy) would be required to achieve negative margins, it is best to perform a core needle biopsy and initiate neoadjvuant chemotherapy [59]. In this scenario, the best treatment option seems to be chemotherapy (actinomycin D, vincristine, cyclophosphamide and cisplatin) followed by complete surgical resection, adding adjuvant radiotherapy for types II and III, and/or patients with disseminated disease. Surgical treatment aims for R-0 resection and this can require anything on the spectrum from generous wedge resection to pulmonary lobectomy, pneumonectomy or extrapleural pneumonectomy [48]. For metastatic or recurrent tumours, some authors recommend high-dose consolidation therapy with autologous stem cell rescue [60, 61]. Prognosis is poor for most children with metastatic PPB. Overall survival is 45% at 5 years and only 8% at 10 years [45].

Type I (pure cystic) and type Ir (regressed/involuted) disease are identical on imaging but have a considerably different clinical presentation. The median age at diagnosis for type Ir patients is 46.5 months, suggesting that these are either precursor lesions that never underwent progression or type I PPB that involuted or ‘burned out’ [45]. For teenagers newly diagnosed with *DICER1* syndrome based on non-pulmonary tumour types, observation of incidental lung cysts can be considered because the overwhelming likelihood is that they are type Ir PPB [52]. Tension pneumothoraces can result from rupture of large cystic PPB, and so large type Ir lesions should be considered for resection despite the age of the patient [62–64]. In contrast, all infants and young children (<10 years) with pulmonary cysts characteristic of type I PPB should undergo surgical resection. 62% of patients with type I PPB were diagnosed by age 1 and 97% by age 3 [45].

## Pulmonary Carcinoid Tumours

Bronchial carcinoids in children are extremely rare; their peak incidence is in adults who are 40–60 years old [65]. The incidence in children is unknown as they are reported in small series or as individual cases in the literature [66]. Pulmonary neuroectodermal tumours (NETs) originate from neurosecretory cells in the bronchial mucosa [65]. The neuroendocrine cells of Kulchitsky are capable of synthesising hormones and neuro-amines (adrenocorticotropic hormone, serotonin, somatostatin, etc.). Pulmonary (and thymic) NETs are classified by World Health Organization, based on their histology and degree of malignancy into: low grade (typical carcinoids), intermediate grade (atypical carcinoids) and high degree of malignancy (non-small cell neuroendocrine carcinoma and small cell carcinoma) [65].

Fortunately, children present primarily with low-grade and slow-growing tumours with low metastatic potential. Presentation with carcinoid syndrome is extremely rare in bronchial carcinoid tumours (1%). Half are asymptomatic [67]. Paediatric bronchial carcinoids affect females 3:1 in contrast with adults where no sex predominance is noted. Most primary tumours have been found in the right middle lobe [66].

### Imaging diagnosis

18-Fluorodeoxyglucose (FDG) PET CT characteristically lacks sensitivity in NETs. Radiotracers for somatostatin or dopamine analogues yield a much better sensitivity (93%) and specificity (87%) for these tumours [65, 68].

### Treatment

Surgical resection of endobronchial carcinoid tumours is the mainstay of treatment. R0 resections achieve a 5-year overall survival of 95% [68, 69]. Specific pulmonary lobectomy techniques are beyond the scope of this guideline.

## Inflammatory Myofibroblastic Tumour

Studies show that inflammatory myofibroblastic tumour (IMT) is likely the third-most common primary pulmonary malignancy in children [70, 71]. Presenting symptoms include respiratory tract infection, chest pain with cough and weight loss. These lesions may also be discovered incidentally on chest radiograph or cross-sectional imaging for other purposes. IMTs are most frequently diagnosed in children and adolescents [72]. Histology is consistent with a spindle cell neoplasm with high expression of the *ALK* tyrosine kinase protein by immunohistochemistry. One half of patients with IMT are found to have clonal activating mutations or rearrangements resulting in oncogenic fusions involving the *ALK* locus at chromosome 2p23 [73]. These tumours are regarded as having intermediate malignant potential because they rarely metastasise but tend to be locally invasive [74]. Complete surgical removal is the cornerstone of therapy for IMTs. Surgery should be limited to non-disfiguring and non-morbid resections because tumours are responsive to some systemic therapies, including targeted ALK inhibitors, non steroidal anti-inflammatory drugs (NSAIDs), steroids or chemotherapy. One study showed that of 14 patients with IMT who were treated with the ALK inhibitor crizotinib, 5 had complete responses, 7 had partial responses and 2 had stable disease [75]. There were no relapses or deaths in this series. The 5-year overall survival rate for this disease is greater than 90% [76].

## Figures and Tables

**Figure 1.** Flow diagram for airway access for anterior mediastinum masses (adapted from Perger et al [24]).

## References

[1] Kumar AP, Green AL, Smith JW (1977). Combined therapy for malignant tumors of the chest wall in children. J Pediatr Surg.

[2] Faber LP (1999). Chest wall tumors: introduction. Semin Thorac Cardiovasc Surg.

[3] Wyttenbach R, Vock P, Tschäppeler H (1998). Cross-sectional imaging with CT and/or MRI of pediatric chest tumors. Eur Radiol.

[4] Shamberger RC, Grier HE (1994). Chest wall tumors in infants and children. Semin Pediatr Surg.

[5] Smith SE, Keshavjee S (1985). Primary chest wall tumors. Ann Thorac Surg.

[6] Panicek DM, Gatsonis C, Rosenthal DI (1997). CT and MR imaging in the local staging of primary malignant musculoskeletal neoplasms: Report of the Radiology Diagnostic Oncology Group. Radiology.

[7] Piperkova E, Mikhaeil M, Mousavi A (2009). Impact of PET and CT in PET/CT studies for staging and evaluating treatment response in bone and soft tissue sarcomas. Clin Nucl Med.

[8] Völker T, Denecke T, Steffen I (2007). Positron emission tomography for staging of pediatric sarcoma patients: results of a prospective multicenter trial. J Clin Oncol.

[9] Hoffer FA, Kozakewich H, Shamberger RC (1990). Percutaneous biopsy of thoracic lesions in children. Cardiovasc Intervent Radiol.

[10] Garrett KM, Fuller CE, Santana VM (2005). Percutaneous biopsy of pediatric solid tumors. Cancer.

[11] Shamberger RC, LaQuaglia MP, Gebhardt MC (2003). Ewing sarcoma/primitive neuroectodermal tumor of the chest wall: impact of initial versus delayed resection on tumor margins, survival, and use of radiation therapy. Ann Surg.

[12] Papanikolaou N, Stathakis S (2004). Tissue inhomogeneity corrections for megavoltage photon beams. Report of Task Group No 65 of the Radiation Therapy Committee of the American Association of Physicists in Medicine.

[13] Shtraus N, Schifter D, Corn BW (2010). Radiosurgical treatment planning of AVM following embolization with Onyx: possible dosage error in treatment planning can be averted. J Neurooncol.

[14] Interiano RB, Kaste SC, Li C (2017). Associations between treatment, scoliosis, pulmonary function, and physical performance in long-term survivors of sarcoma. J Cancer Surviv.

[15] Scalabre A, Parot R, Hameury F (2014). Prognostic risk factors for the development of scoliosis after chest wall resection for malignant tumors in children. J Bone Joint Surg Am.

[16] Harris CJ, Helenowski I, Murphy AJ (2020). Implications of tumor characteristics and treatment modality on local recurrence and functional outcomes in children with chest wall sarcoma: a pediatric surgical oncology research collaborative study. Ann Surg.

[17] Garey CL, Laituri CA, Valusek PA (2011). Management of anterior mediastinal masses in children. Eur J Pediatr Surg.

[18] Piro AJ, Weiss DR, Hellman S (1976). Mediastinal Hodgkin’s disease: a possible danger for intubation anesthesia Intubation danger in Hodgkin’s disease. Int J Radiat Oncol Biol Phys.

[19] Turoff RD, Gomez GA, Berjian R (1985). Postoperative respiratory complications in patients with Hodgkin’s disease: relationship to the size of the mediastinal tumor. Eur J Cancer Clin Oncol.

[20] Griscom NT (1991). CT measurement of the tracheal lumen in children and adolescents. AJR Am J Roentgenol.

[21] Azizkhan RG, Dudgeon DL, Buck JR (1985). Life-threatening airway obstruction as a complication to the management of mediastinal masses in children. J Pediatr Surg.

[22] Shamberger RC, Holzman RS, Griscom NT (1995). Prospective evaluation by computed tomography and pulmonary function tests of children with mediastinal masses. Surgery.

[23] Kawaguchi Y, Saito T, Mitsunaga T (2018). Prediction of respiratory collapse among pediatric patients with mediastinal tumors during induction of general anesthesia. J Pediatr Surg.

[24] Perger L, Lee EY, Shamberger RC (2008). Management of children and adolescents with a critical airway due to compression by an anterior mediastinal mass. J Pediatr Surg.

[25] Malik R, Mullassery D, Kleine-Brueggeney M (2019). Anterior mediastinal masses—a multidisciplinary pathway for safe diagnostic procedures. J Pediatr Surg.

[26] Chaignaud BE, Bonsack TA, Kozakewich HP (1998). Pleural effusions in lymphoblastic lymphoma: a diagnostic alternative. J Pediatr Surg.

[27] Borenstein SH, Gerstle T, Malkin D (2000). The effects of prebiopsy corticosteroid treatment on the diagnosis of mediastinal lymphoma. J Pediatr Surg.

[28] Billmire DF (2006). Malignant germ cell tumors in childhood. Semin Pediatr Surg.

[29] Ramon y Cajal S, Suster S (1991). Primary thymic epithelial neoplasms in children. Am J Surg Pathol.

[30] Stachowicz-Stencel T, Orbach D, Brecht I (2015). Thymoma and thymic carcinoma in children and adolescents: a report from the European Cooperative Study Group for Pediatric Rare Tumors (EXPeRT). Eur J Cancer.

[31] Liu R, Adler DG (2014). Duplication cysts: diagnosis, management, and the role of endoscopic ultrasound. Endosc Ultrasound.

[32] Bobanga ID, Redline RW, DeRoss AL (2016). Oesophageal pseudodiverticulum after foregut duplication cyst excision: case report and literature review. Afr J Paediatr Surg.

[33] Rampersad R, Singh M, Parikh D (2019). Foregut duplications in the superior mediastinum: beware of a common wall with the tracheo-bronchial tree. Pediatr Surg Int.

[34] Partrick DA, Rothenberg SS (2001). Thoracoscopic resection of mediastinal masses in infants and children: an evaluation of technique and results. J Pediatr Surg.

[35] Gun F, Erginel B, DC;nüvar A (2012). Mediastinal masses in children: experience with 120 cases. Pediatr Hematol Oncol.

[36] Brisse HJ, McCarville MB, Granata C (2011). Guidelines for imaging and staging of neuroblastic tumors: consensus report from the International Neuroblastoma Risk Group Project. Radiology.

[37] Cohn SL, Pearson AD, London WB (2009). The International Neuroblastoma Risk Group (INRG) classification system: an INRG Task Force report. J Clin Oncol.

[38] Phelps HM, Ayers GD, Ndolo JM (2018). Maintaining oncologic integrity with minimally invasive resection of pediatric embryonal tumors. Surgery.

[39] Twist CJ, Schmidt ML, Naranjo A (2019). Maintaining outstanding outcomes using response- and biology-based therapy for intermediate-risk neuroblastoma: a report from the children’s oncology group study ANBL0531. J Clin Oncol.

[40] Adams GA, Shochat SJ, Smith EI (1993). Thoracic neuroblastoma: a Pediatric Oncology Group study. J Pediatr Surg.

[41] Jain A, Baracco R, Kapur G (2020). Pheochromocytoma and paraganglioma-an update on diagnosis, evaluation, and management. Pediatr Nephrol.

[42] Weldon CB, Shamberger RC (2008). Pediatric pulmonary tumors: primary and metastatic. Semin Pediatr Surg.

[43] Lal DR, Clark I, Shalkow J (2005). Primary epithelial lung malignancies in the pediatric population. Pediatr Blood Cancer.

[44] Dehner LP, Schultz KA, Hill DA (2019). Pleuropulmonary blastoma: more than a lung neoplasm of childhood. Mo Med.

[45] Messinger YH, Stewart DR, Priest JR (2015). Pleuropulmonary blastoma: a report on 350 central pathology-confirmed pleuropulmonary blastoma cases by the International Pleuropulmonary Blastoma Registry. Cancer.

[46] Pierce JM, LaCroix P, Heym K (2017). Pleuropulmonary blastoma: a single-center case series of 6 patients. J Pediatr Hematol Oncol.

[47] Hill DA, Jarzembowski JA, Priest JR (2008). Type I pleuropulmonary blastoma: pathology and biology study of 51 cases from the international pleuropulmonary blastoma registry. Am J Surg Pathol.

[48] Knight S, Knight T, Khan A (2019). Current management of pleuropulmonary blastoma: a surgical perspective. Children (Basel).

[49] Hill DA, Ivanovich J, Priest JR (2009). DICER1 mutations in familial pleuropulmonary blastoma. Science.

[50] Dehner LP, Messinger YH, Schultz KA (2015). Pleuropulmonary blastoma: evolution of an entity as an entry into a familial tumor predisposition syndrome. Pediatr Dev Pathol.

[51] Foulkes WD, Priest JR, Duchaine TF (2014). DICER1: mutations, microRNAs and mechanisms. Nat Rev Cancer.

[52] Schultz KA, Williams GM, Kamihara J (2018). DICER1 and associated conditions: identification of at-risk individuals and recommended surveillance strategies. Clin Cancer Res.

[53] Leblanc C, Baron M, Desselas E (2017). Congenital pulmonary airway malformations: state-of-the-art review for pediatrician’s use. Eur J Pediatr.

[54] Robinson A, Romao R, Mills J (2018). Decision-making criteria for observational management of congenital pulmonary airway malformations (CPAMs). J Pediatr Surg.

[55] Downard CD, Calkins CM, Williams RF (2017). Treatment of congenital pulmonary airway malformations: a systematic review from the APSA outcomes and evidence based practice committee. Pediatr Surg Int.

[56] Feinberg A, Hall NJ, Williams GM (2016). Can congenital pulmonary airway malformation be distinguished from Type I pleuropulmonary blastoma based on clinical and radiological features?. J Pediatr Surg.

[57] Wu H, Tian J, Li H (2020). Computed tomography features can distinguish type 4 congenital pulmonary airway malformation from other cystic congenital pulmonary airway malformations. Eur J Radiol.

[58] Priest JR, Andic D, Arbuckle S (2011). Great vessel/cardiac extension and tumor embolism in pleuropulmonary blastoma: a report from the International Pleuropulmonary Blastoma Registry. Pediatr Blood Cancer.

[59] Sparber‐Sauer M, Seitz G, Kirsch S (2017). The impact of local control in the treatment of type II/III pleuropulmonary blastoma Experience of the Cooperative Weichteilsarkom Studiengruppe (CWS). J Surg Oncol.

[60] Kaneko H, Isogai K, Kondo M (2006). Autologous peripheral blood stem cell transplantation in a patient with relapsed pleuropulmonary blastoma. J Pediatr Hematol Oncol.

[61] de Castro CG, de Almeida SG, Gregianin LJ (2003). High-dose chemotherapy and autologous peripheral blood stem cell rescue in a patient with pleuropulmonary blastoma. J Pediatr Hematol Oncol.

[62] Cabeza B, Oñoro G, Salido AG (2012). Pleuropulmonary blastoma as characteristic cause of pneumothorax. J Pediatr Hematol Oncol.

[63] Kuzucu A, Soysal O, Yakinci C (2001). Pleuropulmonary blastoma: report of a case presenting with spontaneous pneumothorax. Eur J Cardiothorac Surg.

[64] Piastra M, Ruggiero A, Caresta E (2005). Critical presentation of pleuropulmonary blastoma. Pediatr Surg Int.

[65] Borczuk AC (2020). Pulmonary neuroendocrine tumors Surg Pathol Clin.

[66] Roby BB, Drehner D, Sidman JD (2011). Pediatric tracheal and endobronchial tumors: an institutional experience. Arch Otolaryngol Head Neck Surg.

[67] McMullan DM, Wood DE (2003). Pulmonary carcinoid tumors. Semin Thorac Cardiovasc Surg.

[68] Encinas JL, Ávila LF, García-Cabeza MA (2006). Tumor carcinoide bronquial y apendicular. An Pediatr.

[69] Varela P, Pio L, Torre M (2016). Primary tracheobronchial tumors in children. Semin Pediatr Surg.

[70] Yu DC, Grabowski MJ, Kozakewich HP (2010). Primary lung tumors in children and adolescents: a 90-year experience. J Pediatr Surg.

[71] Lichtenberger JP, Biko DM, Carter BW (2018). Primary lung tumors in children: radiologic-pathologic correlation from the radiologic pathology archives. Radiographics.

[72] Karnak I, Senocak ME, Ciftci AO (2001). Inflammatory myofibroblastic tumor in children: diagnosis and treatment. J Pediatr Surg.

[73] Coffin CM, Hornick JL, Fletcher CD (2007). Inflammatory myofibroblastic tumor: comparison of clinicopathologic, histologic, and immunohistochemical features including ALK expression in atypical and aggressive cases. Am J Surg Pathol.

[74] Brodlie M, Barwick SC, Wood KM (2011). Inflammatory myofibroblastic tumours of the respiratory tract: paediatric case series with varying clinical presentations. J Laryngol Otol.

[75] Mossé YP, Voss SD, Lim MS (2017). Targeting ALK with crizotinib in pediatric anaplastic large cell lymphoma and inflammatory myofibroblastic tumor: a children’s oncology group study. J Clin Oncol.

[76] Kube S, Vokuhl C, Dantonello T (2018). Inflammatory myofibroblastic tumors-a retrospective analysis of the Cooperative Weichteilsarkom Studiengruppe. Pediatr Blood Cancer.

# Discussion: Surgical approach to pulmonary metastasis in children

**Published:** 2022

**Authors:** Jonathan Karpelowksy, Gloria Gonzalez, Guido Seitz

## Introduction

Approximately 10%–40% of all children with solid tumours present with lung metastases at the time of diagnosis and another 20% develop metastases during or after treatment [1] with overall survival (OS) ranging from 20% to 70%, depending primarily on histology [1, 2]. This group of patients still pose significant challenges and while progress has been made in non-metastatic patient groups, similar strides have not been mirrored. Until improvements in systemic or targeted therapy are developed, surgery still has an important role to play in the management of this group.

Tumour biology and the response of the tumour to chemotherapy, radiotherapy and targeted chemotherapy are important factors when making a decision regarding the role of surgery in pulmonary metastasis. Tumours that respond poorly to systemic therapy are more likely to have a beneﬁcial response to surgical resection. Nevertheless, in the absence of effective adjuvant therapy to non-pulmonary metastatic sites, a relative contraindication to pulmonary metastasectomy should be considered.

The following are important principles when managing pulmonary metastasis: (1) the aims of resection are localised resections with clear margins with the aim of preserving adequate lung volume, (2) unnecessary toxic therapy can sometimes be avoided by accurate diagnosis, (3) tumour type and biology is of utmost importance (4) the number of metastases and the disease-free interval are not contraindications to metastasectomy and (5) staged or synchronous bilateral resections are well-tolerated [3].

## Surgical Goals

The goal of metastasectomy needs to be clarified as to whether the goal is diagnostic to aid risk stratification, or therapeutic to enable curative intent. For diagnostic purposes, one to two representative nodules should be obtained through the most minimally invasive technique, this may include thoracoscopic resection (including localisation marking procedures) or needle biopsy under computed tomography (CT) guidance in larger lesions.

Metastasectomy for curative intent should aim to achieve a R0 resection through small non-anatomical localised wedge resections/enucleation with a small margin of normal tissue with maximal preservation of normal lung tissue; lobectomies or segmentectomies are used only in specific circumstances with central lesion adjacent to hilar structures.

## Work-Up

CT scan remains the current standard for identifying pulmonary lesions. Although the high sensitivity of CT can be beneficial, its lack of specificity with respect to differentiating malignant from benign nodules, leading to false-positive interpretations [4]. Conversely for Osteosarcoma, the number of lesions reported on CT scan can often underestimate the true burden of disease by at least 30%–40% [1, 5–7]. It is imperative the surgeon works closely with the radiologist to clearly identify the number and location of the lesions to be resected prior to surgery. Clear documentation with intraoperative correlation of the radiological and operative resection is key to ensuring all lesions are identified.

## Surgical Technique

Both open and thoracoscopic approaches may be suitable. It depends largely on the intent of the procedure (diagnostic or therapeutic), the number and position of the lesions and the local experience available. Each lesion should be resected with a small amount of surrounding lung tissue to ensure clear margins while preserving lung parenchyma. Stapled resections may be utilised but tend to resect more lung parenchyma and provide artefact on subsequent imaging, diathermy and suture resections may minimize this.

A number of techniques have been utilised to aid intraoperative lesion localisation. This is particularly relevant in patients undergoing a thoracoscopic approach where the lack of tactile feel makes localisation even more challenging. Preoperative localisation using CT guided placement of localised dye (Methylene blue +/− and autologous blood patch or lipiodol) [8–12], hook wires or coils [13] have been used in isolation or in combination [14]. Each technique has a failure rate of either coil/wire dislodgment or dye spill, but rarely both [5–7] as such some have advocated for the use of more than one technique to avoid technical failures.

Recently newer techniques using indocyanine green (ICG) and near-infrared fluorescence imaging have gained in popularity. It may either be used as a localised dye injected under CT guidance or by systemic intravenous administration. When injected systemically, its use has typically been in hepatic neoplasms including hepatoblastoma. Although both normal hepatocytes and tumour cells can take up ICG, the excretion by tumour cells is significantly slower leading to relative retention. Its use in identifying hepatoblastoma metastasis is both sensitive and specific [15–17]. More recently ICG use is also being studied in identifying sarcoma pulmonary metastasis although at 10 fold the doses of ICG used in hepatoblastoma [15]. The exact underlying physiological basis for this is yet uncertain.

When presented with bilateral pulmonary metastasis, several approaches are possible. Some surgeons prefer metachronous bilateral thoracotomy [18] with a 2–6 week interval between exploration. While this provides optimal exposure to the ipsilateral hemithorax, it has the disadvantage of requiring two surgeries, and subsequent delays in chemotherapy. To ensure optimal exposure and to minimise delays in chemotherapy, one of the authors (JK) routinely undertakes bilateral synchronous muscle sparing posterolateral thoracotomies together with epidural analgesia for post-operative recovery. As the epidural provides bilateral pain relief, the stay does not exceed that for a unilateral thoracotomy and patients are discharged on the fourth or fifth post-operative day. Two well-established alternative approaches are the median and the transverse sternotomy [19]. The latter improves the exposure (but still provides limited postero-superior access), but is infrequently performed. The sternotomy is a well-tolerated approach in children. In cases of sternotomy, some authors remark that exploration or exposure of the basal or posterior lung segments is difficult [1]. Access especially to the posterior aspect of the left lower lobe can be challenging due to cardiac compression, but can be improved by the transection of the pulmonary ligament and anaesthetic management [20]. Finally a bilateral synchronous thoracoscopic approach has been reported but has the limitation of finding deeper lesions [21].

## Tumour Specific Management

Wilms tumour (please refer to *Wilms Tumour Guidelines*)

Approximately 10% of Wilms tumour patients present with pulmonary metastasis [1, 2, 22]. Surgery at present has a limited role in the management of Wilms pulmonary metastasis with the standard of care including the addition of an anthracycline to chemotherapy and whole lung radiotherapy [23]. Both the Children’s Oncology Group (COG) and the International Society of Pediatric Oncology (SIOP) have recognised the long-term morbidity including pulmonary disease, cardiac disease and an increased risk of breast cancer especially in females [23, 24] to this additional treatment. To avoid these, both the SIOP protocol and the recently closed COG AREN 0533 protocol now avoid radiation in those patients who achieve a complete pulmonary response (CR) within 6 weeks of three-drug chemotherapy. Future trials will explore whether a similar approach may be utilised in patients achieving CR by either chemotherapy alone or a combination of chemotherapy and surgery. Early SIOP data suggest this may be feasible in around 88% of patients [25]. The role of surgery as part of the workup of Wilms may include a biopsy for small undetermined CT nodules at presentation, to confirm pulmonary metastatic disease, since the NWTS-5 study reported that 26% of patients with biopsied CT-only pulmonary lesions had benign nodules [26] emphasising the importance of differentiating between metastases and benign nodules.

Hepatoblastoma (please refer to *Hepatoblastoma and HCC Guidelines*)

Metastatic hepatoblastoma (approximately 20% of cases) has a significant impact on OS, decreasing it to about 50%–65% [27–30]. The mainstay of treatment for pulmonary metastasis is chemotherapy. Current dose intensive cisplatin regimens have about a 50% CR and 46% partial response (PR). Surgery has an important curative role for any remaining metastasis when there is no progressive disease [30].

In off-treatment pulmonary relapses, radical surgery may provide a durable cure in around 30% of patients [31–33].

The timing of metastasectomy for non-responder nodules depends on residual disease following chemotherapy and whether a partial hepatectomy or transplantation is required. Usually metastasectomy is delayed to after definitive local control for the patients requiring partial hepatectomies for surgical treatment, but should be done before definitive liver surgery, to clear metastatic disease prior to undertaking liver transplantation [29, 34]. There are groups which have demonstrated the feasibility of synchronous resection of both local primary tumour and the metastatic disease [35].

Rhabdomyosarcoma (please refer to *Rhabdomyosarcoma Guidelines*)

Rhabdomyosarcoma (RMS) is the most common paediatric soft tissue sarcoma [36]. Multimodal therapy consisting of chemotherapy, radiation therapy and/or surgery is used based on tumour extension, histology and tumour localisation and has improved survival of patients in low-risk localised disease with OS rates of more than 90% [37]. Patients with metastatic disease still have a poor prognosis [38, 39] with 5-year OS rates of 24% in the European Intergroup studies (MMT4-89 and MMT4-91) [40]. The risk stratification for metastatic RMS is based on the Oberlin score involving age, primary tumour site, number of metastases, histology and bone marrow involvement [37, 39].

Pulmonary metastases of RMS can be successfully treated by whole lung irradiation resulting in an improved survival [44]. Dantonello *et al* [40] from the Cooperative Weichteilsarkom Studiengruppe (CWS) group reported on 29 patients with embryonal RMS and lung metastases. Lung metastases were in remission in 22 children after induction chemotherapy, 3 had pulmonary metastasectomy and 9 underwent lung irradiation. Complete remission was achieved in 24/29 with a 5-year-OS of 48.7%. Local treatment of metastases did not improve the outcome in this group of patients [40]. Reports on pulmonary metastasectomy in RMS are rare [41, 42], which might be caused by the fact that pulmonary RMS metastases respond well to the chemotherapy and whole lung irradiation. Therefore, the role of surgery has been relegated to histological confirmation for undetermined CT nodules after induction chemotherapy or for selected cases not responding to chemotherapy.

Non-rhabdomyosarcoma (please refer to the *Non-Rhabdomyosarcoma Soft-tissue Sarcoma Guideline*)

Paediatric non-rhabdomyosarcoma soft tissue sarcoma (NRSTS) are a heterogeneous group of more than 50 different tumour entities [43] including such as synovial sarcoma, malignant peripheral nerve sheath tumour (MPNST), alveolar soft part sarcoma and epithelioid sarcoma. Pulmonary metastases might occur in 77% of these entities, often seen in high grade (Grade III) non-rhabdomyosarcoma [44]. Responses to multimodal treatment are dependent on histology. Synovial sarcoma is a relatively chemo-sensitive tumour while MPNST tended to be more chemo-resistant, same diversity to radiotherapy responses is shown in paediatric NRSTS [45].

Patients with primary metastatic synovial sarcoma treated within the CWS study group had the best prognosis in patients with oligometastatic lung metastases (5-year-OS: 85%) and a worse prognosis in those with multiple bilateral lung metastases (5-year-OS: 13%). Whole lung irradiation was not correlated with better outcomes [46]. Pulmonary metastasectomy can be helpful for the management of synovial sarcoma as in a series of 31 patients undergoing at least one pulmonary metastasectomy demonstrated a 2- and 5-year-OS-rate of 65% and 24%, whereas all other patients died within 2 years from diagnosis of pulmonary disease. The conclusion was that pulmonary metastasectomy may be associated with improved survival if a complete resection could be carried out [47].

Alveolar soft-part sarcoma is a rare tumour entity in which lung metastases might occur in a high volume of patients, but it is a slow growing pattern, allows a good OS, despite the high rate of pulmonary spread [48]. Pulmonary metastasectomy has been reported in some patients treated within the CWS study group [49].

In conclusion, non-rhabdomyosarcoma are a heterogeneous group of tumours, in which pulmonary metastases might occur. The numbers in children are low and therefore it is difficult to give general treatment advice for the management of pulmonary metastases, but surgery should be considered as an option when feasible.

Ewing sarcoma (please refer to *Osteosarcoma and Ewing Sarcoma Guidelines*)

Ewing sarcoma (EW) is the second most common bone tumour in children and adolescence, which can also arise in the soft tissue. Pulmonary metastases can be found in 25%, and the survival rate for metastatic patients is approximately 30% [50]. Irradiation of the lungs has been shown to improve survival in metastatic disease [51]. Therefore, radiation therapy has been included in several EW treatment protocols [52]. In smaller case series (n = 22), pulmonary metastasectomy has been shown to improve outcome compared to patients undergoing whole lung irradiation or chemotherapy alone and the outcome of those patients was also better than in those undergoing radiotherapy and pulmonary metastasectomy [52, 53]. As with other series, selection bias may have played a role given that the authors did not report for disease burden and did not explain their decision regarding choice of therapy. In conclusion, there is little evidence for improved survival in patients undergoing pulmonary metastasectomy for ES. The role of surgery is either to confirm the histology of undetermined CT-nodules at presentation (not recommended by the EURO Ewing Protocol), or to resect suspicious lung nodules after induction chemotherapy to confirm the diagnosis.

Osteosarcoma (please refer to *Osteosarcoma and Ewing Sarcoma Guidelines*)

Osteosarcoma is often associated with pulmonary metastases [54]. The outcome of patients with metastatic osteosarcoma is poor. Analysing the EURAMOS-1 trial, it could be shown that pulmonary metastases at diagnosis were one of the most adverse factors [55].

Besides modern imaging techniques using thoracic CT scan for pulmonary metastases and a preoperative consensus reading of the images prior to surgery by the radiologist and surgeon [19], it has been shown that there is a relevant difference in the number of preoperatively detected lung metastases on CT scan and the intraoperative findings [20]. Different authors have described that there is an underestimation of lung nodules on the CT scans compared to surgery of 30%–40% [19, 47]. The risk of underestimation increases with the number of nodules and there is a cut-off point for the exact correlation between five and ten metastases [1, 20, 42]. On the other hand, there is the discrimination problem between small lung metastases and small benign lesions, which sometimes leads to an overestimation of small metastases on imaging compared to intraoperative findings [54, 56].

Curative treatment approaches for primary metastatic osteosarcoma include removal of all lung nodules as more than 40% of metastatic patients achieving a complete surgical remission can become long-term survivors [57–59]. Survival is significantly correlated with age, site of the primary tumour, number and location of metastases, response to preoperative chemotherapy and completeness and time point of surgical resection of all tumour sites. The number of metastases at diagnosis and the completeness of surgical resection of all clinically detected tumour sites are of independent prognostic value [58]. Additionally, patients with unilateral lung metastases have a better outcome than patients with bilateral lung metastases [60]. Patients with solitary nodules have a better 5-year-OS (75%) than those with two to five metastases (26%) and more than five metastases (23%) [60].

In conclusion, patients seem to benefit from complete removal of lung metastases in osteosarcoma, that has been demonstrated to be best achieved by open approaches, that allows intraoperative diagnosis of all potential pulmonary nodules including those invisible to CT scan [19, 47]. The survival implications of these ‘invisible’ lesions remain unclear [61, 62]. Larger retrospective and even prospective randomised trials may be necessary to settle this controversy. Minimally invasive technique may be considered for later relapses of solitary pulmonary nodules diagnosed after ending osteosarcoma therapy, because ipsilateral disease is not likely to be found [63].

## Complications

After the resection of pulmonary metastases, up to 12% of patients may present with pulmonary complications, which prolongs the hospital-stay and result in an in-hospital mortality rate of up to 2%.

The vast majority of the thoracotomies performed in children consisted of wedge resections, performed in lung isolation. Lobectomies and segmentectomies should not be used, unless totally needed to manage secondary disease.

The most common complications are prolonged air leak; there is no relation demonstrated using sewing or stapler for this complication in adult or paediatric literature, but stapler size should be taken in consideration in children because of the range in sizes of the lung at different ages and the thickness of the parenchyma. Bleeding is also a known complication, being more frequent in segmentectomies and lobectomies, or patients with multiple thoracotomies. The third most common complication is pneumonia – which accounted for 25.0% of all complications in adults [64]. Patients with surgical complications increase two times its hospital stay than in those who had none.

Variables that are related to postoperative complications are intraoperative blood loss and blood transfusion status, the number of peripheral nodules and type of resection and bilateral synchronous thoracotomies. Santos Silva *et al* [65], in adult population, reported that any type of resection other than wedge resections increased the risk of pulmonary complication by 260%, and complications increased by 5% for every nodule resected.

The main complication is not to achieve complete surgical resection. The majority of patients in the paediatric population will be osteosarcoma patients, the calciﬁed metastases in osteosarcoma allow for identiﬁcation by palpation, not possible in non-calciﬁed histologies. After CT identiﬁcation of a pulmonary nodule, further difﬁculties may arise in localising the nodule for diagnostic or therapeutic resection. Superﬁcial lesions can be seen intraoperatively, on visual inspection and larger, ﬁrmer lesions can be palpated, but softer, smaller or deeper lesions can be easily missed. Many techniques, including pre-operative marking with wires, coils and dyes, and localisation with intraoperative ultrasound, have been used in an attempt to solve this problem, allowing a success rate of only 80%–90% for all techniques, other alternatives has been used lately, that may allow better localisation, increasing the role for minimally invasive surgery at least for diagnostic purposes.

### Tips and pitfalls

General anaesthesia and single lung ventilation are helpful to achieve a total lung collapse on the affected side in order to allow whole lung palpation for occult nodules. This can be achieved by the usage of a double-lumen tube. These tubes often need to be positioned by a bronchoscope. In younger children, these tubes are too large and therefore selective bronchial blocker such as Fogarty catheters or commercial bronchial blockers are required, which also need to be placed with the help of a bronchoscope [66] and have some risk of intraoperative dislocation. Endobronchial intubation of the contralateral lung is also an efficient technique to obtain single lung ventilation. A micro-cuffed endotracheal tube one size smaller than what would normally be used for endotracheal intubation.

Removal of lung nodules can either be performed by electrocautery or by laser resection to allow radical surgery with adequate margins as well as sufficient bleeding control. Parenchymal defects often may be left open. The usage of staplers can be helpful during wedge resections, but depend on the size of the child.

Preoperative CT-guided labelling of metastases by guidewires should be carried out directly prior to surgery in general anaesthesia and the patient should be immediately transferred to the OR in order to minimise the risk of displacement of the guidewire.

Intrapleural tumour dissemination after thoracoscopic metastasectomy seems to be a rare event, but might occur in some patients [67].

## Conclusion

Despite significant improvements in the survival of childhood cancer patients, those with metastatic disease have not shown the same equivalent outcomes. While chemotherapy and radiotherapy remain the main modalities in the treatment of metastatic disease, surgery is playing increasingly an important role in the treatment of several paediatric metastatic solid tumours. In some cases, this is diagnostic to confirm metastatic disease or alternatively to obtain tissue at the time of diagnosis or relapse for further analysis to guide a personalised targeted therapy. There is however a subset of cancers where surgery to remove all remaining metastasis can provide a survival benefit. Surgery should be effective, feasible and safe following these basic principles: control of the primary tumour is or could be achieved; there are no extrathoracic non-resectable metastases; no other treatment modality is deemed to be effective for pulmonary nodules; pulmonary function is compatible with the surgical treatment; clinical conditions are compatible with the surgical treatment; and clinical and radiological findings indicated that the metastasis is resectable.

## References

[1] Fuchs J, Seitz G, Handgretinger R (2012). Surgical treatment of lung metastases in patients with embryonal pediatric solid tumors: an update. Semin Pediatr Surg.

[2] Heij HA, Vos A, de Kraker J (1994). Prognostic factors in surgery for pulmonary metastases in children. Surgery.

[3] Heaton TE, Davidoff AM (2016). Surgical treatment of pulmonary metastases in pediatric solid tumors. Semin Pediatr Surg.

[4] Rosenfield NS, Keller MS, Markowitz RI (1992). CT differentiation of benign and malignant lung nodules in children. J Pediatr Surg.

[5] Kayton ML, Huvos AG, Casher J (2006). Computed tomographic scan of the chest underestimates the number of metastatic lesions in osteosarcoma. J Pediatr Surg.

[6] McCarville MB, Lederman HM, Santana VM (2006). Distinguishing benign from malignant pulmonary nodules with helical chest CT in children with malignant solid tumors. Radiology.

[7] Parsons AM, Detterbeck FC, Parker LA (2004). Accuracy of helical CT in the detection of pulmonary metastases: is intraoperative palpation still necessary?. Ann Thorac Surg.

[8] Karpelowsky J (2012). Paediatric thoracoscopic surgery. Paediatr Respir Rev.

[9] Choi BG, Kim HH, Kim BS (1998). Pulmonary nodules: CT-guided contrast material localization for thoracoscopic resection. Radiology.

[10] Nomori H, Horio H (1996). Colored collagen is a long-lasting point marker for small pulmonary nodules in thoracoscopic operations. Ann Thorac Surg.

[11] Wicky S, Mayor B, Cuttat JF (1994). CT-guided localizations of pulmonary nodules with methylene blue injections for thoracoscopic resections. Chest.

[12] Mogi A, Yajima T, Tomizawa K (2015). Video-assisted thoracoscopic surgery after preoperative CT-guided lipiodol marking of small or impalpable pulmonary nodules. Ann Thorac Cardiovasc Surg.

[13] Gaffke G, Stroszczynski C, Rau B (2005). CT-guided resection of pulmonary metastases. RoFo : Fortschritte auf dem Gebiete der Rontgenstrahlen und der Nuklearmedizin.

[14] Martin AE, Chen JY, Muratore CS (2009). Dual localization technique for thoracoscopic resection of lung lesions in children. J Laparoendosc Adv Surg Tech.

[15] Keating J, Newton A, Venegas O (2017). Near-infrared intraoperative molecular imaging can locate metastases to the lung. Ann Thorac Surg.

[16] Yamamichi T, Oue T, Yonekura T (2015). Clinical application of indocyanine green (ICG) fluorescent imaging of hepatoblastoma. J Pediatr Surg.

[17] Kitagawa N, Shinkai M, Mochizuki K (2015). Navigation using indocyanine green fluorescence imaging for hepatoblastoma pulmonary metastases surgery. Pediatr Surg Int.

[18] Hacker FM, von Schweinitz D, Gambazzi F (2007). The relevance of surgical therapy for bilateral and/or multiple pulmonary metastases in children. Eur J Pediatr Surg.

[19] Abbo O, Guatta R, Pinnagoda K (2014). Bilateral anterior sternothoracotomy (clamshell incision): a suitable alternative for bilateral lung sarcoma metastasis in children. World J Surg Oncol.

[20] Fuchs J, Seitz G, Ellerkamp V (2008). Analysis of sternotomy as treatment option for the resection of bilateral pulmonary metastases in pediatric solid. Surg Oncol.

[21] Han KN, Kang CH, Park IK (2014). Thoracoscopic approach to bilateral pulmonary metastasis: is it justified?. Interact Cardiovasc Thorac Surg.

[22] Green DM, Breslow NE, Ii Y (1991). The role of surgical excision in the management of relapsed Wilms’ tumor patients with pulmonary metastases: a report from the National Wilms’ Tumor Study. J Pediatr Surg.

[23] Green DM, Lange JM, Qu A (2013). Pulmonary disease after treatment for Wilms tumor: a report from the national wilms tumor long-term follow-up study. Pediatr Blood Cancer.

[24] Lange JM, Takashima JR, Peterson SM (2014). Breast cancer in female survivors of Wilms tumor: a report from the national Wilms tumor late effects study. Cancer.

[25] Verschuur A, Van Tinteren H, Graf N (2012). Treatment of pulmonary metastases in children with stage IV nephroblastoma with risk-based use of pulmonary radiotherapy. J Clin Oncol.

[26] Ehrlich PF, Hamilton TE, Grundy P (2006). The value of surgery in directing therapy for patients with Wilms’ tumor with pulmonary disease. A report from the National Wilms’ Tumor Study Group (National Wilms’ Tumor Study 5). J Pediatr Surg.

[27] Emre S, Umman V, Rodriguez-Davalos M (2012). Current concepts in pediatric liver tumors. Pediatr Transplant.

[28] Towbin AJ, Meyers RL, Woodley H (2018). 2017 PRETEXT: radiologic staging system for primary hepatic malignancies of childhood revised for the Paediatric Hepatic International Tumour Trial (PHITT). Pediatr Radiol.

[29] Wanaguru D, Shun A, Price N (2013). Outcomes of pulmonary metastases in hepatoblastoma - the prognosis always poor?. J Pediatr Surg.

[30] Zsiros J, Brugieres L, Brock P (2013). Dose-dense cisplatin-based chemotherapy and surgery for children with high-risk hepatoblastoma (SIOPEL-4): a prospective, single-arm, feasibility study. Lancet Oncol.

[31] Meyers RL, Czauderna P, Otte JB (2012). Surgical treatment of hepatoblastoma. Pediatric Blood Cancer.

[32] Meyers RL, Tiao GM, Dunn SP (2012). Liver transplantation in the management of unresectable hepatoblastoma in children. Front Biosci (Elite edition).

[33] Angelico R, Grimaldi C, Gazia C (2019). How do synchronous lung metastases influence the surgical management of children with hepatoblastoma? An update and systematic review of the literature. Cancers.

[34] Seng MS, Berry B, Karpelowsky J (2019). Successful treatment of a metastatic hepatocellular malignant neoplasm, not otherwise specified with chemotherapy and liver transplantation. Pediatric Blood Cancer.

[35] Urla C, Seitz G, Tsiflikas I (2015). Simultaneous resection of high-risk liver tumors and pulmonary metastases in children. Ann Surg.

[36] McDowell HP (2003). Update on childhood rhabdomyosarcoma. Arch Dis Childhood.

[37] Chen C, Dorado Garcia H, Scheer M (2019). Current and future treatment strategies for rhabdomyosarcoma. Front Oncol.

[38] Breneman JC, Lyden E, Pappo AS (2003). Prognostic factors and clinical outcomes in children and adolescents with metastatic rhabdomyosarcoma--a report from the Intergroup Rhabdomyosarcoma Study IV. J Clin Oncol.

[39] Oberlin O, Rey A, Lyden E (2008). Prognostic factors in metastatic rhabdomyosarcomas: results of a pooled analysis from United States and European cooperative groups. J Clin Oncol.

[40] Dantonello TM, Winkler P, Boelling T (2011). Embryonal rhabdomyosarcoma with metastases confined to the lungs: report from the CWS Study Group. Pediatric Blood Cancer.

[41] Erginel B, Gun Soysal F, Keskin E (2016). Pulmonary metastasectomy in pediatric patients. World J Surg Oncol.

[42] Kayton ML (2006). Pulmonary metastasectomy in pediatric patients. Thorac Surg Clin.

[43] Dasgupta R, Rodeberg D (2016). Non-rhabdomyosarcoma. Semin Pediatr Surg.

[44] Pappo AS, Devidas M, Jenkins J (2005). Phase II trial of neoadjuvant vincristine, ifosfamide, and doxorubicin with granulocyte colony-stimulating factor support in children and adolescents with advanced-stage nonrhabdomyosarcomatous soft tissue sarcomas: a Pediatric Oncology Group Study. J Clin Oncol.

[45] Mancini B, Roberts K (2012). Pediatric non-rhabdomyosarcoma soft tissue sarcomas. J Radiat Oncol.

[46] Scheer M, Dantonello T, Hallmen E (2016). Primary metastatic synovial sarcoma: experience of the CWS study group. Pediatric Blood Cancer.

[47] Stanelle EJ, Christison-Lagay ER, Wolden SL (2013). Pulmonary metastasectomy in pediatric/adolescent patients with synovial sarcoma: an institutional review. J Pediatr Surg.

[48] Kayton ML, Meyers P, Wexler LH (2006). Clinical presentation, treatment, and outcome of alveolar soft part sarcoma in children, adolescents, and young adults. J Pediatr Surg.

[49] Sparber-Sauer M, Seitz G, Kalle T (2018). Alveolar soft-part sarcoma: primary metastatic disease and metastatic relapse occurring during long-term follow-up: Treatment results of four Cooperative Weichteilsarkom Studiengruppe (CWS) trials and one registry. Pediatric Blood Cancer.

[50] Grünewald TGP, Cidre-Aranaz F, Surdez D (2018). Ewing sarcoma. Nat Rev Dis Prim.

[51] Briccoli A, Rocca M, Ferrari S (2004). Surgery for lung metastases in Ewing’s sarcoma of bone. Eur J Surg Oncol.

[52] Dirksen U, Brennan B, Le Deley MC (2019). High-dose chemotherapy compared with standard chemotherapy and lung radiation in Ewing sarcoma with pulmonary metastases: results of the European Ewing Tumour Working Initiative of National Groups, 99 Trial and EWING 2008. J Clin Oncol.

[53] Letourneau PA, Shackett B, Xiao L (2011). Resection of pulmonary metastases in pediatric patients with Ewing sarcoma improves survival. J Pediatr Surg.

[54] Bielack SS, Hecker-Nolting S, Blattmann C (2016). Advances in the management of osteosarcoma. F1000 Res.

[55] Smeland S, Bielack SS, Whelan J (2019). Survival and prognosis with osteosarcoma: outcomes in more than 2000 patients in the EURAMOS-1 (European and American Osteosarcoma Study) cohort. Eur J Cancer (Oxford, England : 1990).

[56] Dix DB, Seibel NL, Chi YY (2018). Treatment of stage IV favorable histology Wilms tumor with lung metastases: a report from the Children’s Oncology Group AREN0533 Study. J Clin Oncol.

[57] Ciccarese F, Bazzocchi A, Ciminari R (2015). The many faces of pulmonary metastases of osteosarcoma: Retrospective study on 283 lesions submitted to surgery. Eur J Radiol.

[58] Kager L, Zoubek A, Pötschger U (2003). Primary metastatic osteosarcoma: presentation and outcome of patients treated on neoadjuvant Cooperative Osteosarcoma Study Group protocols. J Clin Oncol.

[59] Ritter J, Bielack SS (2010). Osteosarcoma. Ann Oncol.

[60] Kager L, Kempf-Bielack B, Bielack S (2010). Synchronous and metachronous lung metastases in high-grade osteosarcoma. Jpn J Clin Oncol.

[61] Su WT, Chewning J, Abramson S (2004). Surgical management and outcome of osteosarcoma patients with unilateral pulmonary metastases. J Pediatr Surg.

[62] Karplus G, McCarville MB, Smeltzer MP (2009). Should contralateral exploratory thoracotomy be advocated for children with osteosarcoma and early unilateral pulmonary metastases?. J Pediatr Surg.

[63] Fernandez-Pineda I, Daw NC, McCarville B (2012). Patients with osteosarcoma with a single pulmonary nodule on computed tomography: a single-institution experience. J Pediatr Surg.

[64] Casiraghi M, De Pas T, Maisonneuve P (2011). A 10-year single-center experience on 708 lung metastasectomies: the evidence of the international registry of lung metastases. J Thorac Oncol.

[65] Santos Silva R, Beraldo PS, Santiago FF (2010). Risk factors for pulmonary complications in patients with sarcoma after the resection of pulmonary nodules by thoracotomy. J Bras Pneumol.

[66] Scanagatta P, Girelli L (2017). Metastasectomy in pediatric patients: indications, technical tips and outcomes. J Thorac Dis.

[67] Ang KL, Tan C, Hsin M (2003). Intrapleural tumor dissemination after video-assisted thoracoscopic surgery metastasectomy. Ann Thorac Surg.

# Discussion: Surgical strategies in pelvic tumours

**Published:** 2022

**Authors:** Timothy Rogers, Pablo Lezama, Erica Fallon, Bibekanand Jindal, Jan Godzinski

## Evaluation

### Epidemiology

Pelvic tumours in children are a heterogeneous group comprised of different histopathological types that arise from organs in the pelvis, sacral neural structures or the musculoskeletal structures of the pelvis and perineum [1]. Included are presacral tumours that arise between the sacrum and posterior rectal wall; an area of complex embryology that is reflected in the varied types of masses that arise in this location. Pelvic masses can be neoplastic or non-neoplastic, benign or malignant, congenital or acquired [2]. Pelvic anatomy is gender-dependent, consequently expanding the list of possible tumour pathologies in both sexes, but especially in females.

### Clinical presentation

Patients with pelvic tumours present with a mass and/or signs and symptoms affecting the structures of the pelvis or pelvic side wall. The viscera can obstruct or get compressed resulting in pain, outflow obstruction and decreased visceral capacity. Bleeding can occur with visceral surface involvement, and it should not be mistaken for menstruation in females. Musculoskeletal and neural structures can also be affected. A pelvic mass should be considered for patients with any of the clinical features noted in Table 1.

Any patient presenting with the signs and symptoms described in Table 1 should undergo quick investigation. Delays in diagnosis may lead to survival compromise and/or permanent functional impairment. This is the reason why all paediatric and surgical communities should be aware of differential diagnosis. It is important to recognise that delay in diagnosis of intra-pelvic tumours is not uncommon and therefore a high index of suspicion is required because delay can make treatment more complex and can worsen prognosis. It is well recognised for sacrococcygeal tumours, that there is an increasing risk of malignancy with increasing age at diagnosis.

Age at presentation and sex are important variables that determine the type and presentation of pelvic tumours, with females more commonly affected.

In the antenatal and perinatal period, the commonest causes include sacrococcygeal teratomas (SCT), ovarian cysts formed under the influence of maternal hormones and congenital/developmental lesions. Perineal hamartoma, pelvic neuroblastoma, vascular malformations and rhabdomyosarcoma (RMS) do rarely occur. Presacral masses can occur in the context of congenital anomalies such as with anorectal malformations and the Currarino triad and should be actively sought for or excluded [3]. Antenatal ultrasound complemented with magnetic resonance imaging (MRI) usually differentiates pelvic lesions and allows planning of foetal and obstetric care [4].

In toddlers and young children, pelvic tumours usually present with a palpable mass or any combination of signs and symptoms shown in Table 1. Urogenital RMS, pre-sacral teratoma or pelvic neuroblastoma can masquerade as dysfunctional voiding or constipation, potentially causing a delay in diagnosis. Vaginal bleeding in premenarchal girls needs investigation to exclude RMS or germ cell tumour.

In older children and adolescents, a benign pelvic mass is more likely to be an appendix mass/abscess, or inflammatory bowel disease phlegmon, but ovarian cyst pathology in females is common [5]. A pelvic tumour will more frequently be an ovarian teratoma, ovarian epithelial cystic tumour or pelvic sarcoma.

Pelvic tumours are also diagnosed during routine surveillance for patients with known predisposition syndromes: Gorlin syndrome (Bilateral ovarian fibroma) [6], Peutz–Jeghers syndrome (ovarian sex cord tumours), Olliers disease and Maffucci syndrome (Juvenile granulosa cell tumour), DICER syndrome (ovarian Sertoli–Leydig cell tumour) [7].Gonadal dysgenesis can result in ovarian gonadoblastoma formation [5]. Rarely an ectopic pelvic kidney may present as a palpable pelvic mass, or nephroblastoma within a pelvic kidney [8].

Osteosarcoma and Ewing sarcoma arising from the pelvic side-wall tend to present to the paediatric surgeon in pre-adolescent and adolescent age-groups [9].Examples of rare tumours in this region include chordoma [10], neurofibroma, schwannoma, lymphoma and fibroma.

## Workup

A comprehensive clinical assessment (sometimes under general anaesthesia) should be completed, which includes a digital rectal examination (with consent) that ascertains whether the lesion is presacral or arises in front of the rectum. Bimanual palpation determines tumour mobility and may also establish whether the lesion is solid or cystic.

**Lab:** Blood

Complete blood count, complete metabolic profile and coagulation profile

Tumour markers: alpha-fetoprotein, beta-human chorionic gonadotropin, cancer antigen-125, Inhibin

Additional markers such as lactate dehydrogenase, carcinoembryonic antigen and CA19.9 may be useful but are not routinely recommended.

: Urine catecholamines

**Imaging:**

An X-ray of the sacrum (AP and lateral view) may demonstrate widening of the presacral space with sacral bony destruction (chordoma), the ‘scimitar sign’ (anterior meningocele) or retro-rectal calcification (teratoma). Preliminary ultrasound of the pelvis and abdomen determines whether the lesion is solid or cystic, the relationship of the lesion to surrounding structures and may determine the tumour organ of origin. Further imaging includes MRI or computed tomography (CT) scan. Chest X-ray and chest CT scan are performed in cases of malignancy.

The ability to interpret cross-sectional imaging is essential for surgeons managing patients with pelvic tumours. Differentiating between inflammatory and neoplastic disease, determining the organ of origin, examining the vascular anatomy in relation to the tumour and determining the extent of disease (contralateral ovarian involvement) are some competencies required for image interpretation. Imaging also helps in deciding the best surgical approach for resection of the lesion.

In the case of a pre-sacral mass where there is a possible dural connection, consultation with neurosurgery before resection is advised as removal without addressing a dural connection may result in leakage of cerebrospinal fluid and central nervous system infection.

## Indications and Principles of Biopsy

A patient with suspected SCT or ovarian tumour will typically NOT require a diagnostic biopsy as tumour markers and imaging are sufficient before proceeding to definitive resection. Needle biopsy should not be performed for cystic lesions, so as to avoid meningitis after inappropriate puncture of a meningocele, or infection and bleeding of a cystic mass. Most other tumours require biopsy for tissue diagnosis and biological information as their treatment typically involves induction chemotherapy before definitive resection (+/− radiotherapy) or definitive radiotherapy [11]. A small number of patients may present with primarily resectable tumours, but this approach requires prior agreement by the solid tumour board.

When primary resection is not indicated, examination under anaesthesia at the time of biopsy is very helpful in assessing the site of origin and extent of the tumour. Cystoscopy, vaginoscopy (female) and endoscopic biopsy are appropriate for bladder, prostate (male) and vaginal tumours. Bimanual rectal/abdominal palpation can assist this evaluation. Sufficient biopsy material should be obtained for diagnosis, biological studies and tumour banking. Adequate biopsies of pelvic side wall tumours are obtained with minimally-invasive techniques, namely US or CT-guided core-needle biopsy, or laparoscopic biopsy; invasive open incisional biopsy is not usually required. In the case of perineal/perianal RMS, inguinal lymph node sampling should be performed before commencement of chemotherapy [12].

## Perioperative Management

### Role and timing of multimodality therapy

SCT and ovarian tumours typically undergo primary resection and depending on histopathological findings and staging, require chemotherapy if malignant. Patients with localised pelvic Neuroblastoma (L1) that do not encase neurovascular structures (iliac vessels and sciatic notch), do not infiltrate adjacent structures and do not have intraspinal extension (absence of Image-Defined Risk Factors (IDRFs)), may undergo gross total resection [13].

However, patients who have neuroblastoma with IDRFs (L2) should be managed with neoadjuvant chemotherapy, followed by function-preserving partial resection and adjuvant therapy. Most other tumours including bladder, prostatic, uterine, vaginal, vulval, perineal/perianal and pelvic side-wall tumours require biopsy and tissue diagnosis, as they are typically treated with chemotherapy first and subsequently have delayed definitive surgery or radiotherapy. For further detail, refer to the guidelines on specific tumour-types (*Germ cell tumours, Rhabdomyosarcoma, Ewing sarcoma Guidelines*).

Fertility preservation procedures such as sperm-banking or gonadal cryopreservation should be considered as early as possible during the patient’s treatment journey [14].

### Preoperative considerations

Preoperative multidisciplinary planning should include the assessment of comorbidities, magnitude of the operation, capacity of the anaesthesia team, intraoperative monitoring, reliable upper extremity vascular access, urinary catheter, availability of blood, appropriate allocation of postoperative level of care and monitoring and postoperative pain control. If a neoadjuvant chemotherapy protocol is used, surgery should follow blood count recovery, and the planned timing of surgery should not be delayed. If a stoma is anticipated, consultation with a stoma-therapist will ensure counselling and optimal abdominal marking.

Informed consent for surgery should include a comprehensive discussion about possible complications and the need for surveillance to identify possible recurrence and/or long-term bladder/bowel functional problems. Management of these problems is included and documented in the consent process.

## Surgery

### Surgery goals

***Ovarian tumours:*** (please also refer to *GCT guidelines*)

Most ovarian tumours are benign, therefore ovarian-preserving resection is indicated in most patients with ovarian pathology [15]. Oophorectomy is indicated for malignant tumours or where it is not feasible to obtain a complete resection and spare ovarian tissue.

***Sacrococcygeal teratoma***: (please also refer to *GCT guidelines*)

**Foetal**

Large vascular SCT can cause high-output cardiac failure and non-immune hydrops through vascular shunting [16]. These patients should be referred for consideration of pre-natal intervention such as foetal surgery, radiofrequency ablation and Ex Utero Intrapartum Treatment (EXIT)-to-resection.

**Neonatal**

Foetuses with large SCTs should be delivered by cesarean section to avoid dystocia and minimise rupture and bleeding of the tumour. The neonatal and surgical teams should be pre-warned. Pre-delivery MRI should be considered particularly if the baby shows signs of cardiac failure and is likely to be unstable after delivery.

The newborn is treated on the neonatal intensive care unit, and MRI obtained to assess the extent and vascularity of the tumour to facilitate the operative approach. Most tumours can be safely removed in the first few days of life.

Special care should be taken to protect the SCT from trauma and catastrophic bleeding. Vitamin K should be given and blood products should be immediately available. In the event of tumour bleeding, direct pressure should be applied whilst surgical consultation is sought. A temporary tourniquet may be applied around the base of the tumour to tamponade the bleeding whilst the neonate is transferred to the operation room for definitive haemorrhage control.

In the case of Currarino Triad, the pre-sacral mass should be treated at the time of ano-rectal malformation repair [17]. Patients with congenital anal stenosis or funnel anus should all undergo MRI of the pelvis and spine because of the high association (30%) with presacral masses and spinal cord abnormalities such as tethered cord in this subtype of anorectal malformation.

***Bladder/prostate RMS*** (please also refer to *RMS guideline*)

Ensuring urinary tract drainage is essential to avoid or treat obstructive uropathy and minimise nephrotoxicity from induction chemotherapy. A transurethral bladder catheter is preferred to suprapubic catheterisation in view of the risk of tumour contamination along the suprapubic catheter tract. Temporary percutaneous nephrostomy/nephrostomies should be used if internal stenting (JJ stents) is not possible to relieve obstruction to the upper tracts prior to commencement of chemotherapy. Vesicostomy is not recommended.

Primary resection is only indicated for small tumours in the dome of the bladder that are well away from the bladder trigone. Tumour volume reduces with chemotherapy; initial chemotherapy permits less aggressive local treatment with equal survival to radical surgery. The local therapy plan should be made by a multidisciplinary team (MDT) with experience in treating these tumours, with the goal of obtaining disease control whilst minimising loss of function and morbidity [18]. Where the tumour involves the bladder trigone, bladder-neck and/or prostate, the decision needs to be made about the appropriateness of conservative resection (R1 or R2) with brachytherapy (BT), radiotherapy alone or mutilating surgery to obtain a complete resection (R0). Incomplete resection by conservative surgery is only indicated if the remaining tumour can be adequately treated with planned BT in prepubertal boys. Partial prostatectomy without radiotherapy carries a high risk of local relapse. Where a conservative approach is not feasible, and in adolescents, the choice is between radical surgery and external beam/proton radiotherapy.

***Uterine/vaginal/vulval RMS***

Tumours at this site are very chemo-sensitive. Patients with favourable histology and biopsy proven complete response to chemotherapy, may not require any local therapy. For those with residual disease after chemotherapy, local treatment is necessary. BT has generally replaced surgery for local control. Patients with unfavourable histology must receive radiotherapy. Partial vaginectomy must only be considered if a R0 resection can be achieved without mutilation. Intracavity BT is used in all other cases with temporary ovarian transposition. For RMS of the cervix uteri, the same principles should be applied [19].

***Pelvic neuroblastoma*** (please also refer to *neuroblastoma guideline*)

Preoperative anorectal manometry and urodynamic studies may be warranted to assess for occult sphincter dysfunction. Preoperative MRI delineates the neural and sacral involvement. The surgical strategy and risk of complications are determined by the tumour location and stage at the time of diagnosis. Tumours arising in the pelvis are generally associated with excellent long-term survival, even when macroscopic disease is left *in-situ* in order to preserve major nerves or vascular structures [20]. In addition, the morbidity of complete resection in this anatomic area is very high (15%–35%) due in large part to injuries of the lumbosacral plexus or denervation to the bowel or bladder, resulting in urinary and faecal incontinence; therefore, incomplete resection should be considered to preserve function [21]. The surgical strategy for advanced disease should avoid the sacrifice of important structures as there is a lack of survival advantage with radical resection. L1 tumours can safely undergo complete resection.

Surgical approach includes laparotomy, laparoscopy, posterior sagittal approach or a combination of these to obtain the best tumour exposure for resection.

***Pelvic side wall tumours***

The pelvis is composed of three pairs of bones connected posteriorly by the sacrum and anteriorly by the pubic symphysis to complete the ring. A stable pelvic ring and hip joint are needed to support weight-bearing on the lower limb and inform the options for surgical resection and reconstruction [22]. Bone tumours are covered in a separate guideline but included is a summary of tumours arising from pelvic bones and the muscles of the inner pelvis.

Ewing sarcoma and osteosarcoma are the two most common bony pelvic malignancies in childhood and adolescence. Ewing sarcoma occurs at this site in a quarter of cases and is the most common bony malignancy of the pelvis; pelvic osteosarcoma is rare childhood and adolescence.

Treatment of tumours at this site depends on their histopathology but typically are not primarily resectable. Induction chemotherapy is followed by an assessment to determine optimal local therapy which usually requires radiotherapy or a combination of radiotherapy and delayed surgery to achieve a non-mutilating complete resection [23]. Modifications of internal hemipelvectomy aim to achieve a complete excision and preserve a stable pelvic ring and vertebral column, and the ability to walk2. New 3D technology allows custom-made implants.

Tumours arising from muscles of the inner pelvis can be divided into either chemotherapy and radiotherapy sensitive (RMS and RMS-like sarcomas) tumours, or resistant tumours that comprise the others. Knowledge of which group the tumour falls into, informs the local therapy decisions. Only in exceptional circumstances is a primary non-mutilating R0 resection feasible. Most frequently, when surgery is performed after induction chemotherapy, a macroscopic resection (R0/R1) attempting to preserve important functional structures like pelvic nerves, a functional anal sphincter complex, iliac vessels and ureters, is combined with radiotherapy delivered either pre- or post-operatively. In radiotherapy sensitive tumours, surgery may be omitted with definitive radiotherapy. Radiotherapy markedly decreases the risk of recurrence and can rarely be omitted [24].

## Key Steps

### Ovarian tumours

Before embarking on ovarian surgery, it is important to perform an exploration (laparoscopic or open) looking for evidence of tumour spread in the peritoneal cavity, omentum, liver and retroperitoneal lymph nodes. It is also important to assess the contralateral ovary for synchronous tumours. Benign cysts can be decompressed, avoiding spillage before removal. Benign solid tumours and most especially malignant tumours should be removed intact to avoid tumour-spillage and consequently up-staging the patient. Peritoneal fluid or irrigation fluid should be sent for cytology, and abnormal omentum and peritoneum should be removed (or biopsied if removal is not possible). Abnormal lymph nodes should be sampled [25] (See guideline on Ovarian Germ cell tumours).

### Sacrococcygeal teratoma

- Ensure that vitamin K has been given
- Blood products should be in theatre in-case of massive haemorrhage
- Preoperative bowel preparation is considered important by some surgeons
- Always place a urethral catheter before positioning the patient
- Approach is dependent on the location and extent of the tumour (Altman classification).

Usually the approach is trans-perineal with the patient in the prone ‘Jack-knife’ position. The perineal incision must consider the post-resection reconstruction options and typically involves a ‘chevron’ incision so as not to disrupt the perianal skin.

For massive SCTs or Altman classification 3–4 tumours, consider an initial abdominal approach to obtain proximal vascular control, ligation of the median sacral artery and initial tumour dissection [26]. The whole abdomen and lower body should be cleaned and draped to facilitate turning the patient in the operative field.

For large vascular tumours, upper body central venous assess and an arterial line should be inserted. Close intra-operative communication between surgeon and anaesthetist is required to ensure physiological stability and if the patient requires re-positioning, the endotracheal tube position is carefully maintained. Dissection proceeds around the surface of the tumour, preserving the sphincter mechanism as far as possible and includes en-bloc removal of the coccyx with the mass. Dissection of the anterior tumour surface is facilitated by the placement of a rectal Hagar dilator to avoid rectal injury. Once the tumour has been removed and haemostasis assured, care is taken to reconstruct the levator ani complex and pelvic floor during wound closure.

### Presacral tumours

The surgical approach to presacral tumours is similar to that for Altman classification 3-4 SCTs. Selection of the approach is the key to successful resection, provides optimal exposure of the tumour and minimises complications. The relationship of the tumour within the pelvis to sacral vertebra S4 is key when selecting an abdominal (anterior), perineal (posterior) or combined abdomino-perineal approach.

An abdominal approach is indicated for lesions with the lowest extent of the tumour above the S4 vertebra. A midline or Pfannenstiel incision achieves excellent exposure of pelvic structures, iliac vessels and ureters. The sigmoid colon and rectum are mobilised anteriorly, taking care not to cause injury to the ureters, sacral nerve roots and presacral venous plexus. The laparoscopic and robotic approaches are being increasingly used to resect tumours above the S4 vertebral level.

The perineal (posterior) approach is suitable for low lying tumours below S4 or those with a superior margin that can be felt by digital rectal examination. The patient is placed in the prone ‘Jack knife’ position and a Chevron or a longitudinal incision is made. Resection of the coccyx and distal sacrectomy may be performed depending upon the pathology and extent of the lesion.

A combined abdominal-perineal approach is used when a large tumour extends both below and above the S4 vertebral level. The abdominal approach (open or laparoscopic) is performed first with mobilisation of the tumour off the rectum and ligation of the median sacral artery before extensive tumour mobilisation. The laparoscopic approach may more easily identify the vessel for ligation to minimise the risk of intra-operative bleeding.

### Bladder/prostate RMS

Selection of the correct local control treatment options are discussed above. When indicated, conservative surgery and BT should be performed in a centre with this expertise. Refer to *Bladder/Prostate Rhabdomyosarcoma Guidelines* for organ-preserving techniques.

When radical cystectomy or cysto-prostatectomy is performed, the patient is positioned in the supine position and the incision made through the midline of the lower abdomen. A systematic abdominal examination is performed before starting the resection. An extra-peritoneal approach can be used if the tumour is not adjacent to the peritoneum, as this allows the subsequent urinary reconstruction to be kept extra-peritoneal and minimises the risk of peritoneal urine leak. If the tumour is adjacent to the peritoneum, the en-bloc resection should include the overlying peritoneal surface to ensure clear margins. Key points of the resection include distal mobilisation and ligation of the ureters which allows for their passive dilatation and facilitates subsequent ureteral anastomosis to the urinary diversion. It is important to define the lateral and posterior pedicles of the bladder and doubly ligate the vessels, whilst preserving nerves for anal continence. Dissection should continue in the plane posterior to Denonvillier’s fascia that lies anterior to the rectum. When dissecting the anterior bladder neck, the dorsal vein complex needs to be ligated and divided. Care is taken when transecting the urethra posteriorly to avoid rectal injury [27].

After removal of the tumour specimen, reconstruction can be with an incontinent ileal conduit, or a continent pouch [28].

### Uterine/vaginal/vulval RMS

BT is a preferred form of local treatment for vaginal tumours and should be performed in a centre with this expertise. It can be applied as intracavitary and/or interstitial BT. The impact on future fertility should be considered, and temporary transposition of the ovaries, either laparoscopic or open, may be required for patients undergoing BT if the anticipated ovarian doses exceed tolerance.

In patients with persistent tumours at the corpus uteri after induction chemotherapy, hysterectomy should be performed.

### Pelvic side wall tumours

Depending on the tumour, a multi-speciality surgical team should include a paediatric surgeon, neurosurgeon, orthopaedic surgeon, as well as a neurophysiologist [29]. Electrophysiologic monitoring should be used during resection of these tumours to mitigate against nerve injury. Potential for bleeding should be appreciated and blood products for transfusion should be available.

Access to the tumour depends on its location and the surrounding critical structures. Surgical approaches include low midline, Pfannenstiel or para-iliac incisions. Peri-anal tumours can be approached through a posterior-sagittal incision, and exposure optimised when combined with a coccygectomy. Minimal invasive techniques have limited value for definitive resection, but in two-access surgery the abdominal step may be approached laparoscopically.

### Pelvic neuroblastoma

A lower midline or transverse incision can be used. The iliac arteries and veins should be controlled early to avoid vascular injury and minimise bleeding. Care is taken to prevent injury to the ureters and sacral nerve roots. Electrophysiologic monitoring should be used during resection of these tumours. Often the obturator nerve can be visualised distally near the obturator foramen and traced proximally to the area of the lumbosacral plexus. When a tumour fills the pelvis, access to the internal iliac vessels may be impossible. Under this circumstance, division of the symphysis pubis allows more space for dissection [30]. An attempt should be made to preserve at least one hypogastric nerve and the anterior division of one internal iliac artery in order to preserve sexual function. A conservative surgical approach leaving some tumour behind is preferable to an attempt at complete resection if this approach risks significant morbidity.

## Tips, Pitfalls and Complications

### Tips

- Except for ovarian tumours and SCTs, pelvic tumours should undergo biopsy rather than resection in the first instance.
- Benign ovarian tumours in children require an ovarian-preserving procedure.
- Malignant ovarian tumours require MDT discussion followed by oophorectomy.
- SCT can usually be primarily resected, although when presenting late beyond the neonatal period with malignant transformation, will need induction chemotherapy before delayed resection.
- Massive vascular SCTs may benefit from pre-operative vascular embolisation.

### Pitfalls

- Beware of the pelvic tumour that masquerades as constipation or dysfunctional urine voiding.
- Don’t forget to obtain tumour-markers before resection of ovarian tumours and SCTs.
- Don’t forget to check the contra-lateral ovary before operating on an ovarian mass.
- Avoid upfront major resection of pelvic organs for pelvic tumours; most will respond to chemotherapy, allowing for either delayed conservative surgery and preservation of function, or definitive radiotherapy.
- For Bladder/prostate and utero-vaginal RMS, consult a centre of excellence early to formulate the primary tumour treatment plan.

### Complications

Continue long-term follow-up after resection of pelvic tumours to identify and manage bladder and bowel functional problems. In patients who have had ovarian tumours need to continue long-term follow-up due to the risk of developing metachronous or recurrent tumours.

## Postoperative Considerations

The postoperative period requires attention to maintaining homoeostasis, analgesia, adequate urinary drainage and nutritional intake. Babies should have soiled nappies replaced frequently to keep the incision sites clean. In older children and adolescents, venous thrombo-prophylaxis and early mobilisation should occur.

The place of post-resection chemotherapy and locoregional radiation therapy is determined at the solid tumour board meeting with knowledge of the histopathological report that describes the quality of resection and the pathological diagnosis.

## Prognosis and Follow-up

Overall survival of patients is determined by pathological diagnosis, stage of disease and quality of resection.

SCT identified and treated in the neonatal period have an excellent overall survival with 95% being benign. However, massive SCTs can cause pre-natal death, or catastrophic haemorrhage with attempted resection. Neurogenic bladder and bowel dysfunction occur in approximately 30% of patients and therefore all these patients require long-term follow-up [31].

Malignant germ cell tumours are usually highly responsive to platinum-based chemotherapy and surgery with survival rates above 80% in patients with metastases.

Ovarian teratomas risk development of metachronous tumours in up to 23%, therefore these patients require interval ultrasound with long-term follow-up into adulthood [32].

A national consensus guideline in preparation, for benign ovarian tumours, recommends 2-yearly follow-up with US until the child reaches the age of 16 years. The young person should then be referred to the adolescent gynaecologist for fertility assessment. This approach allows identification of recurrence and metachronous disease early, when tumours are still small, and more amenable to repeat ovarian-preserving surgery (Braungart – unpublished).

Prognosis in patients with pelvic RMS is dependent on several factors including; tumour site of origin, patient age, tumour biology, stage, tumour size and extent that are used to place patients into different risk-groups that define treatment intensity (See *IPSO RMS guideline, EpSSG guideline, INSTRuCT consensus documents*).

Pelvic neuroblastomas typically have favourable biology with excellent overall survival, however functional outcomes may be affected (See *Neuroblastoma Guidelines* for follow-up).

Prognosis and follow-up of pelvic side wall tumours depends on the histopathological tumour-type, stage and treatment delivered. Surgery for local relapse is extremely difficult and is generally not indicated for progressive disease or relapsed disease when already on chemotherapy. Radiotherapy in radiotherapy-responsive tumours may be indicated, even if radiotherapy was previously used. Mutilating surgery should be considered, but can be avoided if there is response to chemotherapy (See IPSO Bone tumour guideline).

## Figures and Tables

**Table 1.** Clinical presentations of a pelvic mass.

Organ affected (directly or indirectly) by pelvic mass	Clinical presentation
Bladder/prostate	Dysuria Frequency Stranguria Haematuria Drippling Incontinence Infection Retention Renal tract obstruction/dysfunction
Ovary/uterus/vagina	Antenatal diagnosis of mass/cyst Pain from compression Pain from ovarian/para-ovarian torsion Vaginal bleeding Vaginal mass/polyp Vulval nodule
Rectum/anus	Constipation Intestinal obstruction Soiling
Pelvis/sacrum/nerves	Limp Weakness Sensory loss

## References

[1] Groff DB (2001). Pelvic neoplasms in children. J Surg Oncol.

[2] Hosalkar H, Dormans J (2005). Surgical management of pelvic sarcomas in children. Pediatr Blood Cancer.

[3] Halleran DR, Vilanova-Sanchez A, Reck CA (2019). Presacral masses and sacrococcygeal teratomas in patients with and without anorectal malformations: a single institution comparative study. J Pediatr Surg.

[4] Kazan-Tannus JF, Levine D (2007). Imaging of fetal tumors. Ultrasound Clin.

[5] Heo SH, Kim JW, Shin SS (2014). Review of ovarian tumors in children and adolescents: Radiologic- pathologic correlation. Radiographics.

[6] Ball A, Wenning J, Van Eyk N (2011). Ovarian fibromas in pediatric patients with basal cell nevus (Gorlin) syndrome. J Pediatr Adolesc Gynecol.

[7] Scollon S, Anglin AK, Thomas M (2017). A comprehensive review of pediatric tumors and associated cancer predisposition syndromes. J Genet Couns.

[8] Oyinloye AO, Wabada S, Abubakar AM (2020). Wilms tumor in a left pelvic kidney: a case report. Int J Surg Case Rep.

[9] Kadhim M, Womer RB, Dormans JP (2017). Surgical treatment of pelvic sarcoma in children: outcomes for twenty six patients. Int Orthop.

[10] Kayani B, Hanna SA, Sewell MD (2014). A review of the surgical management of sacral chordoma. Eur J Surg Oncol.

[11] Wang H, Li F, Liu J (2014). Ultrasound-guided core needle biopsy in diagnosis of abdominal and pelvic neoplasm in pediatric patients. Pediatr Surg Int.

[12] Casey DL, Wexler LH, Laquaglia MP (2014). Patterns of failure for rhabdomyosarcoma of the perineal and perianal region. Int J Radiat Oncol Biol Phys.

[13] Brisse HJ, McCarville MB, Granata C (2011). Guidelines for imaging and staging of neuroblastic tumors: Consensus report from the international neuroblastoma risk group project. Radiology.

[14] Lautz TB, Burns K, Rowell EE (2021). Fertility considerations in pediatric and adolescent patients undergoing cancer therapy pediatric cancer survivorship infertility fertility preservation. Surg Oncol Clin NA.

[15] Renaud EJ, Sømme S, Islam S (2019). Ovarian masses in the child and adolescent: an American Pediatric Surgical Association Outcomes and Evidence-Based Practice Committee systematic review. J Pediatr Surg.

[16] Hambraeus M, Arnbjörnsson E, Börjesson A (2016). Sacrococcygeal teratoma: a population-based study of incidence and prenatal prognostic factors. J Pediatr Surg.

[17] AbouZeid AA, Mohammad SA, Abolfotoh M (2016). The Currarino triad: what pediatric surgeons need to know. J Pediatr Surg.

[18] Martelli H, Haie-Meder C, Branchereau S (2009). Conservative surgery plus brachytherapy treatment for boys with prostate and/or bladder neck rhabdomyosarcoma: a single team experience. J Pediatr Surg.

[19] Lautz TB, Martelli H, Fuchs J (2020). Local treatment of rhabdomyosarcoma of the female genital tract: Expert consensus from the Children’s Oncology Group, the European Soft-Tissue Sarcoma Group, and the Cooperative Weichteilsarkom Studiengruppe. Pediatr Blood Cancer.

[20] Zobel M, Zamora A, Sura A (2020). The clinical management and outcomes of pelvic neuroblastic tumors. J Surg Res.

[21] Cruccetti A, Kiely EM, Spitz L (2000). Pelvic neuroblastoma: low mortality and high morbidity. J Pediatr Surg.

[22] DeSilva JM, Rosenberg KR (2017). Anatomy, development, and function of the human pelvis. Anat Rec.

[23] Ludwig J (2015). Ewing Sarcoma Family of Tumors.

[24] DuBois SG, Grier HE, Lessnick SL (2015). Chapter 61 – Ewing Sarcoma.

[25] Gershenson DM, Frazier AL (2016). Conundrums in the management of malignant ovarian germ cell tumors: toward lessening acute morbidity and late effects of treatment. Gynecol Oncol.

[26] Gangadharan M, Panda S, Almond PS (2014). Management of preterm giant sacrococcygeal teratoma (GSCT) with an excellent outcome. J Surg Case Rep.

[27] Yuk HD (2019). Radical cystectomy. Manag Urothelial Carcinoma.

[28] Minevich E, Sheldon CA (2012). Reconstruction of the Bladder and Bladder Outlet.

[29] Wirbel RJ, Schulte M, Mutschler WE (2001). Surgical treatment of pelvic sarcomas. Clin Orthop Relat Res.

[30] Kiely E (2007). A technique for excision of abdominal and pelvic neuroblastomas. Ann R Coll Surg Engl.

[31] Hambraeus M, Al-Mashhadi A, Wester T (2018). Functional outcome and health-related quality of life in patients with sacrococcygeal teratoma – a Swedish multicenter study. J Pediatr Surg.

[32] Taskinen S, Urtane A, Fagerholm R (2014). Metachronous benign ovarian tumors are not uncommon in children. J Pediatr Surg.

# Discussion: Rare tumours

**Published:** 2022

**Authors:** Hany Gabra, Pablo A Lobos

## Introduction

In general, paediatric tumours are relatively rare when compared to tumours in adults and represent less than 1% of all cancer diagnoses [1]. Rare tumours in children are a group which are infrequently encountered and accounts for 5%–10% of all childhood cancers which themselves are rare diseases [2]. Interestingly when reviewing the epidemiology of those tumours, the majority (75%) occur in patients who are aged between 15 and 19 years [3]. They form a diverse group of pathology and clinical presentations and represent a challenge in their management.

To define what is a Rare Paediatric Tumour is difficult. In addition, the definition of a rare tumour is not uniform among paediatric and adult groups. However, it is currently acceptable that these are the tumours are considered rare when: which are not captured by a particular treatment protocol or they are known tumours but occur either in unusual age or location; for instance, colorectal carcinoma, renal cell carcinoma are often encountered in the adult population but are exceptionally rare in children [1]. In addition if they are generally rare independent of age, or they exhibit rare histologic features or they are relatively common tumours in rare locations, e.g. Neuroblastoma of the urinary bladder [4].

The definition of rare tumours has also been interpreted differently by various investigator groups. For instance, The European Cooperative Study Group for Pediatric Rare Tumors (EXPeRT) defines a rare childhood cancer as one that has an incidence rate of less than 2 per million per year, is not considered in clinical trials or both [5]. In general, most paediatric clinical trials involve childhood cancers that are relatively more common than other childhood cancers. Interestingly some rare paediatric cancer, e.g., hepatoblastoma are good examples of rare tumours which have been studied well and have a well-recognised standard treatment protocols whereas other rare, infrequent cancers are often not registered or reported. There are epidemiological patterns for rare childhood cancers:

1. Low incidence tumour entity occurring exclusively in children, e.g. Pancreatoblastoma (PBL), mesoblastic nephroma.
2. A tumour entity with bimodal age distribution and age dependent biology, e.g. germ cell tumours.
3. An adult-type tumour entity with rare occurrence during childhood and adolescence, e.g. breast cancer and malignant melanomas (MMs).
4. An adult-type tumour entity with rare occurrence during childhood and adolescence but with distinct biology.

Currently there are various groups attempting to rationalise rare paediatric tumours (see Table 1). In 2000, the Rare Tumors in Pediatric Age Project (TREP) was established in Italy followed by the European EXPeRT group in 2008 which represent a collaboration from groups from Italy, France, Poland, the United Kingdom and Germany. Their aim to enhance collaborative clinical and biologic research in rare paediatric cancers [5]. Both the TREP and EXPeRT groups have developed treatment and staging recommendations for selected rare cancers and have identified a group of experts who assist in consultations and clinical decisions. The EUROCARE (European Cancer Registry) project is a population-based cancer database that reports the survival rates from 74 population-based registries in 29 European countries, this has served as an important resource for investigators and offers opportunities for improved collaboration [6].

The Children’s Oncology Group (COG) Rare Tumor Committee is another group which used The COG registry to explore the epidemiology of rare childhood cancers. The COG has developed the Children’s Cancer Research Network, which uses a process to register all patients with childhood cancer who are under the age of 20 years and treated at COG institutions in the United States or Canada. There are other dedicated registries for individual rare cancer is, e.g., the International Pleuropulmonary Blastoma Registry (IPBR) and the Pediatric and Wild-Type GIST Clinic (see Table 1 for web links).

In the forthcoming topics, some of the rare tumours will be discussed in view of the latest literature and most agreeable guidelines by IPSO.

## Figures and Tables

**Table 1.** Some of the resources for paediatric rare cancers.

Name	Website
**COG**	http://www.childrensoncologygroup.org/
Cancer.Net	http://www.cancer.net/coping-and-emotions/managing-emotions/finding-support-and-information/finding-information-and-support-resources-rare-cancers
National Cancer Institute (NCI) Physician Data Query (PDQ)	http://www.cancer.gov/cancertopics/pdq
American Cancer Society	http://www.cancer.org/
NORD	http://www.rarediseases.org/
Rare Cancer Alliance	http://www.rare-cancer.org/
NCI	http://www.cancer.gov/
Association of Cancer Online Resources (ACOR)	http://www.acor.org/
Orphanet	http://www.orpha.net/
International Pleuropulmonary Blastoma Registry	http://www.ppbregistry.org/
EXPeRT	https://www.raretumors-children.eu/
Italian TREP Project	http://www.trepproject.org/
German Childhood Cancer Registry	http://www.kinderkrebsregister.de/
International Pediatric Adrenocortical Tumors Registry (IPACTR)	http://www.stjude.org/ipactr
Pediatric and Wildtype GIST Clinic	http://www.pediatricgist.cancer.gov/
CCLG (Children’s Cancer and Leukaemia Group, UK)	https://www.cclg.org.uk/member-area/treatment-guidelines/rare-tumours
Eurocare	

## References

[1] Brecht IB, Kaatsch P, Schneider DT, Brecht IB, Olson TA, Ferrari A (2012). Epidemiology. Rare Tumors in Children and Adolescents.

[2] Pappo AS, Furman WL, Schultz KA (2015). Rare tumors in children: progress through collaboration. J Clin Oncol.

[3] Shehata B, Shulman S, Schneider DT, Brecht IB, Olson TA (2012). Rare tumors: pathology and biology perspectives. Rare Tumors in Children and Adolescents.

[4] Mohamed A, Campbell-Hewson Q, Gabra HOS (2019). Neuroblastoma of the Urinary Bladder in an Infant. Eur J Pediatr Surg Rep.

[5] Ferrari A, Schneider DT, Bisogno G (2013). The founding of the European Cooperative Study Group on Pediatric Rare Tumors-EXPeRT. Expert Rev Anticancer Ther.

[6] Gatta G, Mallone S, Zwan JM (2014). Cancer survival in Europe 1999–2007 by country and age: results of EUROCARE-5 – A population-based study. Lancet Oncol.

# Discussion: Pancreatic Tumours

**Published:** 2022

## Background

Malignant pancreatic tumours are rare in children and adolescents, with an incidence of 0.46 cases per 1 million individuals younger than 30 years [1–4]. Limited series have been reported, and even the largest children’s hospitals only reported a handful of cases over a number of decades [5–8].

In children, a wide variety of tumours are encountered, which include both benign and malignant lesions [9]. These neoplasms can be classified as exocrine, endocrine, epithelial, non-epithelial, cystic and solid. The malignant tumours encompass a wide range of histologies that includes:

- Solid-cystic papillary tumour of the pancreas
- Pancreatoblastoma
- Neuroendocrine tumours
- Pancreatic carcinoma

Current treatment options for pancreatic tumours include surgical management and medical treatments; however, a surgical approach is preferred as it is associated with better long-term survival [5].

Because of the rarity and heterogeneity, there is a lack of standardised guidelines, and treatment is extremely challenging. In the United States, some data were provided by the Surveillance, Epidemiology and End Results (SEER) database and the National Cancer Database [5], a nationwide hospital-based cancer registry sponsored by the American College of Surgeons Commission on Cancer and the American Cancer Society. In Europe, treatment guidelines were developed inside the European Cooperative Study Group for Pediatric Rare Tumors (EXPeRT), on the basis of previous European National Studies [10, 11].

## Solid-Cystic Papillary Tumour (SCPT) of The Pancreas

### Epidemiology

This tumour, also known as Frantz tumour, is a rare neoplasm that mostly affects women and adolescents, with a predilection for blacks and East Asians [12].

The World Health Organization (WHO) has reviewed the classification of these lesions as epithelial low-grade malignant neoplasm [13]. For this reason, a complete surgical resection is the mainstay of treatment to achieve excellent long-term outcomes [14]. Local recurrence and metastases are rare, mostly recognised in adult women, and often reported after incomplete resection, peripancreatic tissue infiltration, neural/vascular invasion or lymph node spread [15].

SCPT is a very friable tumour, and tumour rupture and haemoperitoneum have been reported [16].

### Clinical presentation

Usually, patients present with non-specific clinical features, like abdominal discomfort and pain. Large SCPT may cause nausea, and/or vomiting, possibly due to compression of adjacent viscera by the tumour. Jaundice is rare even for tumours originating from the head of the pancreas. Sometimes, tumours are incidentally discovered after an abdominal trauma or on routine physical examination.

### Workup

The workup must include:

- Complete blood count, complete metabolic profile, coagulation profile
- AFP, CA19-9, CA125
- Urinary metanephrines and catecholamines

### Imaging

Tumours must be evaluated by

- Abdominal ultrasound (US)
- Magnetic resonance imaging (MRI) or
- Computed tomography (CT) scan

Imaging studies reveal classic imaging characteristics of SCPT: large size, mixed solid and cystic nature, encapsulation and haemorrhage.

### Differential diagnosis

The differential diagnosis includes many non-neoplastic and neoplastic cystic lesions like inflammatory pseudo-cyst, mucinous cystic tumours, microcystic adenoma and mucinous cystadenocarcinoma.

### Biopsy

The impact of tumour spillage is still controversial, explaining some degree of reluctance to perform preoperative biopsies. The pre-operative diagnosis of SCPT, however, is possible by means of fine needle aspiration cytology, which reveals loose aggregates of small, monotonous cells with scant cytoplasm surrounding thin-walled capillaries. Despite the small number of preoperative biopsies performed in the series from Crocoli *et al* [14], these procedures did not seem to influence the outcome.

As it happens to adult patients, an increasing number of endoscopy-guided fine-needle biopsy can be performed in paediatric patients. The reported diagnostic yield of this technique for pancreatic lesions to date is good, with a diagnostic accuracy of 78%–95%, sensitivity 78%–95% and specificity 75%–100% [15–17].

### Surgery – Resection of primary tumour

Surgery is the cornerstone of treatment for SCPT of pancreas, with a good prognosis expected after radical resection of the primary lesion [12, 14].

Laparoscopy, as well as robotic surgery, has recently been described as a feasible approach for pancreatic lesions, without an increase in postoperative complications [18–20].

Complete resections can be achieved through different surgical procedures, depending on the location of the tumour: central/distal resection for tumours located in the body and tail region and Whipple pancreaticoduodenectomy for tumours located in the head of the pancreas [21, 22]. The enucleation of the tumour has been also described, but it should be discouraged because of the risk of local relapse [12].

Distant metastases are rare in children with an incidence of less than 5%. They are usually located in the liver and, once resected do not seem to affect long-term prognosis. Therefore, the surgical resection of metastases is warranted, if feasible.

In the last years, ablative therapies have been developing for the treatment of solid tumours in children. The experience is still limited, however, there are some reports on the use of these techniques either for primary pancreatic tumours or liver metastases [23, 24].

### Postoperative considerations

Surgical procedures on pancreas are burdened by a high rate of early complications and late morbidity. Therefore, an accurate short-term and long-term follow-up is of utmost importance [21, 22] and should be the focus of forthcoming clinical investigations, due to the rarity of patients [7, 21].

### Histology

The fragility of the vascular supply leads to secondary degenerative changes and cystic areas of haemorrhage and necrosis [25].

Grossly, tumours are usually well-circumscribed, often encapsulated, ranging in size from 3 to 18 cm in diameter. The cut surface has a variegated appearance with solid, cystic and papillary areas with necrosis and haemorrhages [1].

Microscopically, extensive necrosis and degenerative changes are common. The tumour cells are arranged mostly in pseudopapillary and with occasional monomorphic pattern. The nuclei are uniform and round with an even chromatin pattern and small nucleoli. Often, nuclear grooves are seen. Hyaline globules are also noted in many cases. They have low mitotic activity, and usually do not have perineural and vascular invasion [26].

The cells surrounding the hyalinised fibrovascular stalks form the pseudopapillae. A highly specific paranuclear dot-like immunoreactivity pattern for CD99 has been described [25, 27]

### Role and timing of multimodality therapy

The management in case of metastases or invasion of adjacent structures is difficult and it has not been homogenous. The use of chemotherapy and radiotherapy has been sporadic with controversial results [28]. In some cases, the decision to avoid giving any treatment was based on the slow progression of SCPT, characterised by a long survival [29], however, some studies have demonstrated a possible sensibility to gemcitabine, epirubicine, docetaxel, paclitaxel and mitomycin C [30]. There are no specific chemotherapy guidelines and each case needs to be discussed in the presence of a Multidisciplinary Team (MDT).

## Pancreatoblastoma (PBL)

Pancreatoblastoma (PBL), although rare, is one of the most common pancreatic exocrine tumours in Childhood [31]: It accounts for 10%–20% of all pancreatic tumours and typically presents in the first decade of life, with a median age at diagnosis of 5 years [11, 25] The congenital form, defined as tumour detected before 3 months of age, is even less common, with the description of 15 cases until 2015 [31].

Patients with Beckwith–Wiedemann syndrome have an increased risk of developing this tumour; the syndrome is identified in up to 60% of cases of PBL developing during early infancy and in 5% of children developing the tumour later in life [32]. In the review presented by Ruol *et al* [31], 7 out of 15 cases of congenital PBL were affected by Beckwith Wiedemann Syndrome (BWS). PBL has also been associated with familial adenomatous polyposis syndromes [31].

These tumours tend to be diagnosed at an advanced stage. More than half of all patients were diagnosed with large tumours that either locally extended beyond the pancreas or were metastatic. This observation illustrates their aggressive biology. In addition, a diagnostic delay may occur as a result of often nonspecific clinical symptoms and the extreme rarity of this disease. Metastases usually involve liver, lungs and lymph nodes [11]

PBL was chosen by the EXPeRT as one of the first tumour types to review. Data collected by the different national groups on clinical findings and treatment modalities were exchanged and analysed [11].

### Clinical presentation

In most patients, symptoms comprise abdominal pain, palpable mass in epigastrium, vomiting, jaundice and weight loss. The lesion may also be discovered incidentally. Congenital PBL may be found during the prenatal US.

Tumours originate from the head, body or tail of the pancreas with similar frequency.

Close to 80% of the tumours secrete alpha-fetoprotein, which can be used to measure response to therapy and monitor for recurrence [11]. In some cases, the tumour may secrete adrenocorticotropic hormone (ACTH) or antidiuretic hormone, and patients may present with Cushing syndrome and syndrome of inappropriate antidiuretic hormone secretion [32].

### Workup

The workup should include:

- Complete blood count, complete metabolic profile (pancreatic enzymes) and coagulation profile
- AFP (to be monitored at diagnosis and during treatment)
- beta-HCG, CEA, CA19-9, CA125

### Imaging

Tumours must be evaluated by

- Abdominal ultrasound (US)
- Magnetic resonance imaging (MRI) or
- Thorax and abdominal Computed tomography (CT) scan

Imaging studies reveal a solid or dishomogeneous mass, sometimes well circumscribed, with septa and calcifications.

An initial biopsy, preferably a core needle biopsy, is acceptable in some cases.

### Staging

Since a tailored shared staging system did not exist for PBL, the ExPERT group decided to classify patients according to a surgical staging system based on the results of initial surgery, as follows [11]:

### Treatment

Even if surgery represents the mainstay of treatment for the cure of these children, chemotherapy is also necessary. Radiotherapy has been suggested for selected inoperable tumours. Using a multimodality approach, close to 80% of patients can be cured [11].

### Surgery

Biopsy for histology and molecular studies is essential, and it is usually done as a first step since many patients are unresectable at diagnosis and require neoadjuvant chemotherapy.

Surgery remains the mainstay of treatment of PBL, and a complete surgical resection is required to cure the patient. Procedures include distal pancreatectomy and Whipple pancreaticoduodenectomy in case of tumour of the head [14, 33]. The resection of metastases after chemotherapy is acceptable, if feasible.

### Chemotherapy

For large, unresectable or metastatic tumours, preoperative chemotherapy is indicated; PBL commonly responds to chemotherapy, and a cisplatin-based regimen is usually recommended. The PLADO regimen, which includes cisplatin and doxorubicin, is the most commonly used regimen: this treatment, in the European studies, was modelled on the base of molecular similarity with hepatoblastoma [3, 11, 34]. Although radiation therapy has been used in unresectable or relapsed cases, its role in the treatment of microscopic disease after surgery has not been defined [11, 34]. Response has been seen for patients with relapsed or persistent PBL treated with gemcitabine [35] and vinorelbine and oral cyclophosphamide [36]. High-dose chemotherapy with autologous haematopoietic stem cell rescue has been reported to be effective in selected cases [3].

Despite there are some internationally agreed recommendations for the first-line treatment, very little is known about management of relapse and role of high-dose chemotherapy. The most often used combinations included etoposide, cyclophosphamide/ifosfamide and cisplatin/carboplatin. A review from Reggiani *et al* [37] showed that the outcome for patients with recurrent PBL was not always dismal, especially when surgery is possible.

### Histology

This tumour is thought to arise from the persistence of the foetal analogue of pancreatic acinar cells. Pathology shows an epithelial neoplasm with an arrangement of acinar, trabecular or solid formations separated by dense stromal bands [25]. CTNNB1 gene mutations have been described in some cases, suggesting that PBL might result from alterations in the normal pancreas differentiation [38].

## Neuroendocrine Tumours (NET)

Extra-appendicular NET are very rare tumours arising from the chromaffin cells, present in several sites and organs. They are slow-growing malignancies included in the group of the so-called ‘orphan diseases’ [39].

Neuroendocrine tumours (NET) of the gastrointestinal tract and pancreas are extremely rare in the paediatric population and limited data are available. In children, the incidence is estimated to be around 0.5 cases per million/year [39].

NETs are sporadic in most cases, but may also be part of a hereditary syndrome: pancreatic NETs are associated with tuberous sclerosis (TS), multiple endocrine neoplasia type 1 (MEN1), von Hippel–Lindau syndrome and neurofibromatosis type 1 (NF1).

In most cases, NET of the gastrointestinal tract in children are located in the appendix. Pancreatic NET are a small but partially distinct group of the gastrointestinal neuroendocrine neoplasms. The most common in this group are insulinomas; however, in some research, the gastrinoma type neoplasms are perceived to be most common in children.

### Histology

The recent 2010 classification for gastroenteropancreatic NETs introduced a grading system based on mitotic count and Ki67 proliferation index, recognising three grades of malignancy: NET grade 1: ≤2% well differentiated; NET grade 2: 3%–20% moderately differentiated and NET grade 3: >20% neuroendocrine carcinoma [40].

### Symptoms

The symptoms are mostly unspecific (pain and weight loss); less frequently they are due to tumour’s secretion (diarrhoea, hypoglycaemia and Zollinger–Ellison syndrome). Sometimes, pancreatic NET are hormonally active. Hypoglycaemia is the dominant symptom of insulinoma. In children, hypoglycaemia usually manifests as behavioural disorders, convulsions or coma.

Gastrinoma secretes gastrin and typical symptoms include gastrointestinal ulcers (Zollinger–Ellison syndrome), chronic abdominal pain or symptoms of reflux disease; less common are diarrhoea and weight loss. Another symptom that may also be observed is anaemia caused by abnormal iron absorption. Since these symptoms are non-specific, diagnosis most frequently is made with considerable delay, even up to 4–6 years after the occurrence of the first symptoms.

Determining the level of chromogranin A (Cg A) in the blood is of essential significance in the laboratory diagnostics of NET, associated to the hormonal evaluation on the base of suspected tumour [41].

Most of the recommendations for imaging follow the guidelines accepted for adult patients. Computed tomography (CT) and magnetic resonance imaging (MRI) are highly sensitive in detecting both the primary tumour and potential metastases [42].

Functional imaging techniques, such as 111In-pentreotide scintigraphy, positron emission tomography/computed tomography (PET/CT) with 11C-5-hydroxytryptophan (11C-5-HTP), bone scintiscan, are also important for diagnosis [39].

Diagnostic and therapeutic guidelines in adult patients are well standardised [43]. The mainstay of treatment in localised tumours is the complete surgical resection, but in case of locoregional invasiveness or distant metastasis, the treatment options are challenging, because the response to conventional chemotherapy is poor [39, 41].

Traverso–Longmire pancreaticoduodenectomies, splenopancreatectomies, distal pancreatic resection and enucleation have been described in the series of Virgone [39], as well as hepatic transcatheter arterial chemoembolisation (TACE) in a patient with multiple liver metastases before being treated with an orthotopic liver transplantation.

## Pseudopapillary Tumours

Solid pseudopapillary tumour (SPT) is most commonly observed in women who are in 20s to 30s of their life and it accounts for 2%–3% of all pancreatic malignancies with 22% of SPT happens in childhood [47]. It has various synonyms like papillary cystic neoplasm/tumour, papillary epithelial neoplasm, solid and cystic papillary epithelial neoplasm and Frantz tumour. Its origin is not clear but pluripotent stem cells are suspected to be a likely source of this tumour. In children, it most commonly appears as a palpable mass and then followed by abdominal pain as initial symptom. Ultrasound and CT are common imaging studies performed and recently the application of fine-needle aspiration has been advocated for cytologic confirmation but this is still under debate [48]. In contrast to adult where the tumour is commonly located in the tail of pancreas, in paediatric cases, the tumour tends to be situated in head of pancreas. Complete surgical resection with negative margin is a mainstay of treatment to prevent recurrence as the role of chemotherapy or radiotherapy for this tumour has not been proven. SPT has a potential to metastasise or recur after the initial treatment and their rate have been reported as 19.5% for metastasis and 6.6% for recurrence in adult cases [49]. However, the report of recurrence or metastasis in paediatric cases is far less common. Outcome of this tumour is excellent with 95% 5-year survival [48].

## Pancreatic Carcinoma

Pancreatic carcinomas are extremely rare in children, representing less than 5% of paediatric pancreatic tumours [12, 16].

- Acinar cell carcinoma is a particularly rare neoplasm, accounting for approximately 1% of all exocrine tumours of the pancreas in all ages [44]. Although rare in paediatrics, acinar cell carcinoma is more common than ductal cell adenocarcinoma, the most common pancreatic carcinoma in adults [25]. Acinar cell carcinoma may closely simulate neuroendocrine lesions and has overlapping features with PBL and SCPT. The clinical evolution of this tumour in children is better than that observed in adults. Paediatric pathologists should include this entity in the differential diagnosis of primary pancreatic masses in children [44].
- Ductal adenocarcinoma is extremely rare in the first four decades of life. However, ductal adenocarcinoma is associated with several cancer predisposition syndromes, such as hereditary pancreatitis (PRSS1 mutations), familial atypical mole and multiple melanoma (CDKN2 mutations), Peutz–Jeghers syndrome and other hereditary nonpolyposis colon carcinomas (STK11 and germline mismatch repair genes) and syndromes associated with DNA repair gene mutations (such as BRCA2 and ATM) [45]. Age at presentation may be younger in these patients, although occurrence during childhood and adolescence is extremely rare [46].

Presenting symptoms are nonspecific, such as abdominal pain and vomiting, or they are related to local tumour growth.

For the diagnostic/therapeutic guidelines of carcinoma of pancreas, it is worth referring to experts of adults.

## Figures and Tables

**Table:** 

Stage	Definition
I	Completely excised tumour with negative microscopic margins
II	Grossly resected tumours with suspected microscopic residual disease
III	Gross/macroscopic residual disease after initial incomplete resection or biopsy
IV	Distant metastases

## References

[1] Mohan H, Bal A, Punia R (2006). Solid and cystic papillary epithelial neoplasm of the pancreas. J Postgrad Med.

[2] Perez EA, Gutierrez JC, Koniaris LG (2009). Malignant pancreatic tumors: incidence and outcome in 58 pediatric patients. J Pediatr Surg.

[3] Dall’Igna P, Cecchetto G, Bisogno G (2010). Pancreatic tumors in children and adolescents: the Italian TREP project experience. Pediatr Blood Cancer.

[4] Brecht IB, Schneider DT, Klppel G (2011). Malignant pancreatic tumors in children and young adults: evaluation of 228 patients identified through the Surveillance, Epidemiology, and End Result (SEER) database. Klin Padiatr.

[5] Picado O, Ferrantella A, Zabalo C (2020). Treatment patterns and outcomes for pancreatic tumors in children: an analysis of the National Cancer Database. Pediatr Surg Int.

[6] Ellerkamp V, Warmann SW, Vorwerk P (2012). Exocrine pancreatic tumors in childhood in Germany. Pediatr Blood Cancer.

[7] Yu DC, Kozakewich HP, Perez-Atayde AR (2009). Childhood pancreatic tumors: a single institution experience. J Pediatr Surg.

[8] Nasher O, Hall NJ, Sebire NJ (2015). Pancreatic tumours in children: diagnosis, treatment and outcome. Pediatr Surg Int.

[9] Perez EA, Gutierrez JC, Koniaris LG Malignant pancreatic tumors: incidence and outcome in 58 pediatric patients. J Pediatr Surg.

[10] Brennan B (2010). Pediatric pancreatic tumors: the orphan looking for a home. Pediatr Blood Cancer.

[11] Bien E, Godzinski J, Dall’Igna P (2011). Pancreatoblastoma: a report from the European cooperative study group for paediatric rare tumours (EXPeRT). Europ J Cancer.

[12] Dall’Igna P, Cecchetto G, Bisogno G (2010). Pancreatic tumors in children and adolescents: the Italian TREP project experience. Pediatr Blood Cancer.

[13] Basturk O, Hong SM, Wood LD (2015). A revised classification system and recommendations from the Baltimore Consensus Meeting for Neoplastic Precursor Lesions in the Pancreas. Am J Surg Pathol.

[14] Crocoli A, Grimaldi C, Virgone C (2018). Outcome after surgery for solid psudopapillary pancreatic tumors in children: report from the TREP project—Rare Tumors Study Group. Pediatr Blood Cancer.

[15] Irtan S, Galmiche-Rolland L, Elie C (2016). Recurrence of solid pseudopapillary neoplasms of the pancreas: results of a nationwide study of risk factors and treatment modalities. Pediatr Blood Cancer.

[16] Rojas Y, Warneke CL, Dhamne CA (2012). Primary malignant pancreatic neoplasms in children and adolescents: a 20-year experience. J Pediatr Surg.

[17] Yamabe A, Irisawa A, Bhutani MS (2016). Efforts to improve the diagnostic accuracy of endoscopic ultrasound-guided fine-needle aspiration for pancreatic tumors. Endosc Ultrasound.

[18] Sokolov YY, Stonogin SV, Donskoy DV (2009). Laparoscopic pancreatic resections for solid pseudopapillary tumor in children. Eur J Pediatr Surg.

[19] Namgoong JM, Kim DY, Kim SC (2014). Laparoscopic distal pancreatectomy to treat solid pseudopapillary tumors in children: transition from open to laparoscopic approaches in suitable cases. Pediatr Surg Int.

[20] Cecchetto G, Riccipetitoni G, Inserra A (2010). Minimally-invasive surgery in paediatric oncology: proposal of recommendations. Pediatr Med Chir.

[21] Mansfield SA, Mahida JB (2016). Pancreaticoduodenectomy outcomes in the pediatric, adolescent, and young adult population. J Surg Res.

[22] Dasgupta R, Kim PC (2005). Relationship between surgical volume and clinical outcome: should pediatric surgeons be doing pancreaticoduodenectomies?. J Pediatr Surg.

[23] Gomez FM, Patel PA, Stuart S (2014). Systematic review of ablation techniques for the treatment of malignant or aggressive benign lesions in children. Pediatr Radiol.

[24] Zambaiti E, Hinojosa AS, Montano V (2020). Interventional Radiology-guided Procedures in the treatment of pediatric Solid Tumor: a Systematic Review and Meta-Analysis. Eur J Pediatr Surg.

[25] Chung EM, Travis MD, Conran RM (2006). Pancreatic tumors in children: radiologicpathologic correlation. Radiographics.

[26] Patnayak R, Jena A, Parthasarathy S (2013). Solid and cystic papillary neoplasm of pancreas: A clinic-pathological and immunohistochemical study: a tertiary care center experience. South Asian J Cancer.

[27] Laje P, Bhatti TR, Adzick NS (2013). Solid pseudopapillary neoplasm of the pancreas in children: a 15-year experience and the identification of a unique immunohistochemical marker. J Pediatr Surg.

[28] Soloni P, Cecchetto G, Dall’igna P (2010). Management of unresectable solid papillary cystic tumor of the pancreas. A case report and literature review. J Pediatr Surg.

[29] Nakahara K, Kobayashi G, Fujita N (2008). Solid-pseudopapillary tumor of the pancreas showing a remarkable reduction in size over the 10- year follow-up period. Intern Med.

[30] Shimizu T, Murata S, Mekata E (2007). Clinical potential of an antitumor drug sensitivity test and diffusion-weighted MRI in a patient with a recurrent solid pseudopapillary tumor of the pancreas. J Gastroenterol.

[31] Ruol M, Dall’Igna P, Alaggio R (2015). Congenital pancreatoblastoma: a case report. J Ped Surg Case Report.

[32] Chisholm KM, Hsu CH, Kim MJ (2012). Congenital pancreatoblastoma: report of an atypical case and review of the literature. J Pediatr Hematol Oncol.

[33] Lindholm EB, Alkattan AK, Abramson SJ (2017). Pancreaticoduodenectomy for pediatric and adolescent pancreatic malignancy: a single-center retrospective analysis. J Pediatr Surg.

[34] Glick RD, Pashankar FD, Pappo A (2012). Management of pancreatoblastoma in children and young adults. J Pediatr Hematol Oncol.

[35] Belletrutti MJ, Bigam D, Bhargava R (2013). Use of gemcitabine with multi-stage surgical resection as successful second-line treatment of metastatic pancreatoblastoma. J Pediatr Hematol Oncol.

[36] Dhamne C, Herzog CE (2015). Response of relapsed pancreatoblastoma to a combination of vinorelbine and oral cyclophosphamide. J Pediatr Hematol Oncol.

[37] Reggiani G, Affinita MC, Dall’Igna P (2020). Treatment strategies for children with relapsed pancreatoblastoma: a literature review. J Pediatr Hematol Oncol.

[38] Honda S, Okada T, Miyagi H (2013). Spontaneous rupture of an advanced pancreatoblastoma: aberrant RASSF1A methylation and CTNNB1 mutation as molecular genetic markers. J Pediatr Surg.

[39] Virgone C, Ferrari A, Chiaravalli S (2021). Extra-appendicular neuroendocrine tumors: a report from the TREP project (2000–2020). Pediatr Blood Cancer.

[40] De Caro MLDB, Guadagno E, De Rosa G (2018). Pathological classification: GEP, TNET, and rare forms. Neuroendocrine Tumors in Real Life.

[41] Stawarski A, Maleika P (2020). Neuroendocrine tumors of the gastrointestinal tract and pancreas: is it also a challenge for pediatricians?. Adv Clin Exp Med.

[42] Kumbasar B, Kamel IR, Tekes A (2004). Imaging of neuroendocrine tumors: accuracy of helical CT versus SRS. Abdom Imaging.

[43] Kunz PL, Anthony LB (2013). Consensus guidelines for the management and treatment of neuroendocrine tumors. Pancreas.

[44] Tapia B, Ahrens W, Kenney B (2008). Acinar cell carcinoma versus solid pseudopapillary tumor of the pancreas in children: a comparison of two rare and overlapping entities with review of the literature. Pediatr and Develop Pathology.

[45] Rustgi AK (2014). Familial pancreatic cancer: genetic advances. Genes Dev.

[46] Lüttges J, Stigge C, Pacena M (2004). Rare ductal adenocarcinoma of the pancreas in patients younger than age 40 years. Cancer.

# Discussion: Pleuropulmonary Blastoma

**Published:** 2022

**Authors:** Calogero Virgone, Patrizia Dall’Igna

## Evaluation

### Epidemiology

Pleuropulmonary blastoma (PPB) is a very rare and highly aggressive neoplasm arising in the lungs and presenting in early childhood, with most cases diagnosed in children less than 6 years of age. It is a dysembryonic malignancy believed to arise from the pleuropulmonary mesenchyme. PPB is classified into three interrelated clinical-pathologic subtypes, which represent a developmental continuum, according to its macroscopic appearance: Type I (cystic), Type II (solid and cystic) and Type III (solid). The solid component of both type II and III has mixed pattern including high grade sarcoma elements [3–5]. Type I cystic PPB may progress to the aggressive Type II and III PPB but can often regress by losing malignant elements to the Type Ir [1, 2].

### Clinical presentation

PPB should be suspected in young children presenting with a pulmonary lesion with cystic, cystic and solid or completely solid appearance. A PPB diagnosis is challenging in the presence of pure cystic lesions that resemble other congenital cystic lesions of the lung.

PPB in children is characterised by symptoms mimicking a respiratory infection, pneumothorax or lung malformation. The tumour is usually located in the lung, but it may extend to the mediastinal structures, diaphragm and/or parietal pleura. Type I PPB is a localised tumour [3]; metastasis may be present at diagnosis in less than 10% of types II-III PPB, most frequently located in the brain, bones and liver [4].

PPB can be part of the DICER1 syndrome in about 2/3 of patients. Genetic counselling should be proposed to all patients and their families so as to screen for diseases associated with DICER1 mutation [5–7].

### Workup

Laboratory tests include complete blood count, complete metabolic profile and coagulation profile.

Imaging includes chest computed tomography (CT scan) and/or thoracic MRI, brain MRI, radionuclide bone scan, echocardiography. CT scan with contrast enhancement evaluates the primary tumour, its loco-regional extension, the possible involvement of lymph nodes, mediastinum and heart, as well as the diaphragm and liver.

Basic information for surgical planning includes the following:

- Relation of the tumour with surrounding organs and vascular structures
- Evaluation of bilateral disease or coexisting pulmonary malformations
- Evaluation of intravascular extension
- Evaluation of tumour response

### Indications and principles of biopsy

A surgical procedure is necessary to obtain a tumour sample and establish the diagnosis of PPB. If an upfront complete resection is not feasible, an initial biopsy is recommended to obtain a histological diagnosis before neoadjuvant therapy. Core-needle (18 or 16 Gauge) or open surgical biopsies are both possible options: a sufficient amount of tissue should be collected to allow histological, biological and genetic tests. Cytology of pleural fluid is not recommended to be used for diagnosis.

## Perioperative Management

### Role and timing of multimodality therapy

There are three main strategies to treat patients with PPB: Children’s Oncology Group (COG)/International Pleuropulmonary Blastoma Registry (IPBR), Cooperative Weichteilsarkom Studiengruppe (CWS) and International Society of Pediatric Oncology (EXpERT) [3, 8–11]. All these strategies reported similar survival results.

### Preoperative considerations

Surgery planning should consider the respiratory distress experienced by the patients affected by PPB. Special attention is required in patients with mediastinal compression when general anaesthesia is needed to obtain a diagnosis. Any pneumothorax or pleural effusion should be managed promptly.

### Surgery

Surgery represents the mainstay of treatment, to establish the diagnosis and cure the patient. Although limited by number of patients, published reports have shown that complete tumour resection is a major prognostic factor [3, 9–12].

#### Type I PPB

Since these tumours are purely cystic, such lesions should be considered suspicious, especially if there is known familial history of DICER1 disease. A complete upfront resection is the treatment of choice, whenever it could be possible without performing a pneumonectomy, and it should be performed through a thoracotomy. A thoracoscopic approach is discouraged.

If the number and site of cystic lesions make it impossible to perform a complete resection, the operation should be limited to the removal of the largest cysts, and the remaining lesions should be followed-up closely. In these cases, adjuvant chemotherapy might be considered to avoid the progression of a possible type I PPB to type II or III. In case of macroscopic incomplete surgery (R2 resection), a new resection should be planned.

#### Types II-III PPB

Upfront primary tumour resection should only be considered in small tumours, amenable to complete resection without any kind of demolitive surgery: this implies the lack of lymph node or metastatic disease. In all other cases, tumour resection should be considered after neo-adjuvant chemotherapy and, as in type I, upfront pneumonectomies are discouraged.

In the case of a life-threatening situation, upfront surgery may be discussed, but with the aim of a complete resection of the tumour. In the rarer situation when a total pneumonectomy could be necessary to remove the tumour completely, an initial debulking surgery is preferred and a second surgery, after adjuvant chemotherapy, can be planned to remove all residual disease. After neoadjuvant chemotherapy, a second look surgery is highly recommended. In case of no residual lesion visible at imaging, delayed surgery or thoracoscopy is recommended and a biopsy/resection of suspicious lesions or fibrotic remnants are needed.

A careful inspection of the pleural cavity and a palpation of the pulmonary parenchyma are needed: all remaining pleural or lung nodules should be resected or biopsied (the positioning of guidewire by an interventional radiologist right before the operation may be sometimes needed). A complete resection may imply non-anatomical lung resections and, sometimes, the additional removal of mediastinal tissues (pericardium, pleura, diaphragm) may be required due to presence of suspicious lesions. Possible pleural effusion should be collected for cytological analysis.

A total pleuro-pneumonectomy is accepted in case the tumour both showed no regression after chemotherapy, and seemed unresectable otherwise. This choice should be balanced with the possibilities of local treatment with radiotherapy.

After a second look surgery had ended in an incomplete resection, a third surgical look consisting in a pneumonectomy or lung irradiation should be discussed, considering the long-term effects of the multiple procedures.

All removed or biopsied tissues should be sent for histological assessment in order to define the adjuvant radiotherapy fields. The timing of delayed surgery is not defined and can depend on the regimen used: tumour resectability should anyway be evaluated after multidisciplinary board discussion.

Surgery goals

To perform and document a thorough surgical staging

To achieve R0 resection

To prevent tumour spillage

To mitigate complications and resection of other organs.

## Advanced Stages and Relapsed Disease

There is no clear therapeutic need for an upfront resection of metastatic sites. Examination of viable tumours in persistent metastasis after chemotherapy may help in guiding therapy (i.e. targeted therapy) and prevent or confirm rare tumour (RT). Outcomes of patients with recurrent disease remain poor, and the role of surgery in recurrent disease is not defined.

## Postoperative Considerations

Some authors recommend to avoid leaving a chest tub in order to minimise the contamination of the pleura [8].

## Prognosis, Prognostics and Follow-up

When a DICER gene mutation is found, specific screening studies may be recommended to look for PPB and other conditions known to be related to DICER1. Chest screening (by chest X-ray or chest CT) for young children known to have the DICER1 gene mutation but no symptoms have in some cases allowed PPB to be diagnosed when it is at the earliest and most curable stage.

Prognosis is strictly dependent on the PPB type, stage and feasibility of a satisfying local control, but also on the neo-adjuvant and adjuvant treatments available [9–14]. Overall, Type 1 pleuropulmonary blastoma has a favourable prognosis, with 5-year overall survival rate of 91%. However, 10% of cases may ultimately progress to type II or type III disease [1].

## References

[1] Messinger YH, Stewart DR, Priest JR (2015). Pleuropulmonary blastoma: a report on 350 central pathology-confirmed pleuropulmonary blastoma cases by the International Pleuropulmonary Blastoma Registry. Cancer.

[2] Priest JR, McDermott MB, Bhatia S (1997). Pleuropulmonary blastoma: a clinicopathologic study of 50 cases. Cancer.

[3] Priest JR, Hill DA, Williams GM (2006). Type I pleuropulmonary blastoma: a report from the International Pleuropulmonary Blastoma Registry. J Clin Oncol.

[4] Priest JR, Magnuson J, Williams GM (2007). Cerebral metastasis and other central nervous system complications of pleuropulmonary blastoma. Pediatr Blood Cancer.

[5] Boman F, Hill DA, Williams GM (2006). Familial association of pleuropulmonary blastoma with cystic nephroma and other renal tumors: a report from the International Pleuropulmonary Blastoma Registry. J Pediatr.

[6] Hill DA, Ivanovich J, Priest JR (2009). DICER1 mutations in familial pleuropulmonary blastoma. Science.

[7] Schultz KA, Yang J, Doros L (2014). DICER1-pleuropulmonary blastoma familial tumor predisposition syndrome: a unique constellation of neoplastic conditions. Pathol Case Rev.

[8] Bisogno G, Sarnacki S, Stachowicz-Stencel T (2021). Pleuropulmonary blastoma in children and adolescents: the EXPeRT/PARTNER diagnostic and therapeutic recommendations. Pediatr Blood Cancer.

[9] Bisogno G, Brennan B, Orbach D (2014). Treatment and prognostic factors in pleuropulmonary blastoma: an EXPeRT report. Eur J Cancer.

[10] Sparber-Sauer M, Seitz G, Kirsch S (2017). (CWS Study Group) The impact of local control in the treatment of type II/III pleuropulmonary blastoma. Experience of the Cooperative Weichteilsarkom Studiengruppe (CWS). J Surg Oncol.

[11] Childhood Pleuropulmonary Blastoma Treatment (PDQ®)–Health Professional Version.

[12] Grigoletto V, Tagarelli A, Atzeni C (2020). Pleuropulmonary blastoma: a report from the TREP (Tumori Rari in Età Pediatrica) Project. Tumori.

[13] Grigoletto V, Tagarelli A, Sparber-Sauer M (2020). Inequalities in diagnosis and registration of pediatric very rare tumors: a European study on pleuropulmonary blastoma. Eur J Pediatr.

[14] Indolfi P, Bisogno G, Casale F (2007). Prognostic factors in pleuro-pulmonary blastoma. Pediatr Blood Cancer.

# Discussion: Phaeochromocytoma and Paraganglioma

**Published:** 2022

**Authors:** Lucas E. Matthyssens, Imran Mushtaq

## Introduction

Phaeochromocytoma (PCC) and Paraganglioma (PGL) are rare neural crest-derived neuroendocrine tumours, jointly abbreviated as ‘PPGL’ (Orphanet: 29072, 324299, 94080, 276621, 276627; ICD-10: C74.1, C75.5, D35.0, D35.6, D44.7; ICD-O-3: 8700, 8680; OMIM: 171300, 115310, 168000, 601650, 605373, 614165). PCC arise from chromaffin cells of the adrenal medulla and usually secrete catecholamines. PGL originate outside the adrenal gland from the paraganglia of the autonomic nervous system: PGL involving the sympathetic nervous system are usually located in the abdominal/pelvic retroperitoneum (para-aortic, near the renal hilum, within the organ of Zuckerkandl, wall of the urinary bladder) or mediastinum and secrete catecholamines. PGL involving the parasympathetic nervous system are frequently located in the head and neck (skull base, carotid body chemodectoma, glomus jugulare/tympanicum tumour) or upper mediastinum and are mostly non-secreting.

Although PCC and PGL have a distinctly different location, presentation, malignant potential and genetic background, they are frequently combined under the term ‘PPGL’. The estimated incidence of PPGL in adults is 2–8 per million per year [1]. Around 1/5–1/10 of PPGL present in children and adolescents [2] with an estimated incidence of 0.11–2 per million children per year [3–5].

### Symptomatology

Hormone-secreting PPGL may present with headache, sweating, flushing, palpitations, blurred vision, syncope, tremor, gastrointestinal disturbance (including diarrhoea), weight loss and arterial hypertension (sustained and without paroxysms in 63%). In adults, hormone producing PPGL are associated with increased cardiovascular morbidity and mortality [3]. In children, PPGL are a relatively rare cause of secondary arterial hypertension and account for 1%–2% of all childhood hypertensive cases. Around 80% of catecholamine-secreting tumours are PCC and 20% are PGL [6]. Non-secreting PPGL may present as a palpable mass or cause symptoms due to pressure on surrounding structures (blood vessels, nerves) such as hearing loss, pulsatile tinnitus, cough, hoarseness, dysphagia, facial palsy, abnormal tongue motility or pain [3].

In adult practice, as a rule of thumb, ‘10% of PPGLs are extra-adrenal, 10% are bilateral and 10% are malignant’. Around 10%–20% of PPGLs are diagnosed in children and adolescents [2] and these are more frequently extra-adrenal (30%–60%), bilateral or multifocal (up to 32%), malignant (up to 50%) and also more often familial (up to 80%), as Paediatric PPGL patients carry more often a germline mutation (see ‘diagnostic investigations’ below) [7].

### Malignancy

Because of the higher rate of genetic mutations (especially in SDHB and Von Hippel–Lindau (VHL) genes), malignancy in paediatric PPGL may be more frequent than in adults. Malignancy in PPGL is however notoriously difficult or almost impossible to diagnose histopathologically. The use of histological scoring systems such as the ‘PASS score’ may help the pathologist in differentiating/predicting malignancy [8], but the definitive diagnosis is by the presence of metastatic disease in non-chromaffin organs (such as the lung, kidney, bone, liver, spleen and lymph nodes) at presentation or during follow-up. As metastasis may only appear many years after primary tumour excision, the follow-up of all patients with resected PPGL is very important. According to the current World Health Organization – classification, every PPGL should be considered to have some malignant potential [1, 7].

In adults, malignancy is believed to occur ‘in 10%’ (range: 2–26) of all PPGL, especially in sympathetic PPGL. Malignancy is a major cause of mortality, with a 5-year overall survival of metastasised PPGL of 50%–60% [3] The incidence of malignant PPGL in children is higher, ranging from 12% to 56% of reported cases and around 0.02 per million children per year [9].

Markers and risk factors for malignancy are limited and include germline SDHB mutations, ATRX somatic mutations, Ki67 protein expression on immunohistochemical analysis (PASS score), any extra-adrenal location (PGL), tumour size greater than 5 cm diameter and elevated plasma levels of methoxytyramine [1, 9].

## Diagnostic Investigations

### Laboratory Tests:

- Urine tests:
  - 24-hour urinary collection (ideally on two occasions) for the analysis of catecholamines (noradrenaline, adrenaline) and metanephrines (90% sensitivity and 98% specificity).
- Blood tests:
  - Plasma catecholamines (noradrenaline and adrenaline)
  - Plasma metanephrines (97% sensitivity and 85% specificity)
  - Plasma methoxytyramine.

### Imaging:

Radiological investigations:

- Abdominal ultrasound (adrenal, paravertebral, pelvic masses)
- Neck ultrasound in selective patients for associated tumo.rs as pre-operative screening (e.g. children with succinate dehydrogenase gene mutations)
- Cross-sectional imaging: MRI or CT scans of the abdomen and chest, focusing on local invasion or tumour extension into adjacent vessels (e.g. inferior vena cava), as well as lymph nodes or other metastases [1]
- ^123^I-labelled meta-iodo-benzyl-guanidine (MIBG) and/or 18F-FDG-positron emission tomography (PET)/CT scan for confirmation, accurate staging and pre-operative planning in selective cases. (N.B. Routine selective venous sampling for localisation is not usually required. Biopsy should only be undertaken with caution after multidisciplinary tumour board discussion.)

### Genetic Analysis:

Despite their low incidence, more than one third of PPGL are associated with inherited cancer susceptibility syndromes, which is the highest rate among all tumour types [11]. PPGL in children can be sporadic or familial, with almost 60% of apparently sporadic PPGL in children under 18 years old carry germline pathogenic variants in susceptibility genes. In children under 10 years old, 70%–85% were found to have pathogenic variants [12, 13].

*Genetic testing is therefore recommended in every paediatric PPGL patient*: the diagnosis of an inherited form of PPGL drives the clinical management and surveillance [1, 3, 7, 15]. At present, PPGL-related germline mutations have been found in more than 20 genes [7] Transcriptome analysis categorises susceptibility genes by biochemical phenotype in four molecular mRNA subtypes: the ‘pseudohypoxia’ subtype, with a noradrenergic phenotype (SDHA, SDHB, SDHC, SDHD, SDHAF2, VHL, FH, HIF2alpha, EGLN1, EGLN2, KIF1B, EPAS1, ANRT), the ‘kinase signaling’ subtype, with an adrenergic phenotype (RET, NF1, TMEM127, HRAS, BRAF, NGFR, FGFR, PKA), a ‘WNT-altered’ subtype (WNT4, DVL3, driven by MAML3 and CSDE1) and a ‘cortical admixture’ subtype (MAX) [9]. Especially SDHB germline mutations and ATRX somatic mutations may be markers of metastatic disease and poor clinical outcome [7].

Although children with genetically determined predisposition (Von Hippel–Lindau (VHL) disease, Neurofibromatosis (NF) type 1, Multiple Endocrine Neoplasia (MEN) types 1, 2a, 2b, Hereditary Leiomyomatosis and Renal Cell Carcinoma syndrome (HLRCC)) are at risk of developing multiple and bilateral lesions, generally the prognosis in childhood is good. For patients with hereditary PPGL, ‘The Endocrine Society’ clinical practice guidelines for PPGL recommend personalised management by a specialist referral centre with a multidisciplinary team [16].

### Perioperative Management

For patients with PPGL, this should take place within a specialised multidisciplinary paediatric team setting, involving paediatrician (nephrologist/endocrinologist/oncologist), paediatric anaesthetist, paediatric surgeon (general and/or urologist), paediatric nurse specialists and intensive care. Communication between members of the team is vital and should be established at the earliest opportunity to allow adequate planning for operative treatment.

#### Pre-operative management

*Management of arterial hypertension:*

- 2–3 weeks before surgery
  - α-blockade
    - Is usually given as oral phenoxybenzamine, at 0.25–1.0 mg/kg twice daily adjusted according to response (depending on weight but starting dose of 1 capsule (10 mg/dose) can be increased by 10 mg/day)
    - Intravenous phenoxybenzamine can be given if surgery is less than 2 weeks, and if used, should be given for at least 3 days as once daily intravenous infusion of 1 mg/kg, over 2 hours made in 200 mL 0.9% saline, giving a third of the dose over the first hour, the remaining two-thirds over the second hour
    - Adequate α-blockade is indicated by normotension, development of side effects (~10% orthostatic hypotension, tachycardia, nasal congestion).
  - β-blockade
    - Is usually given as oral propranolol, standard dosing regimen at 0.5–1.0mg/kg three times daily
    - If persistent tachycardia or dysrhythmia (and no cardiomyopathy)
    - Administration of β-blocker prior to adequate α-blockade, may compound hypertension secondary to unopposed vasoconstriction and is contra-indicated.
- 24 hours before surgery
  - Commence intravenous fluids (full maintenance) to maintain hydration and ensure adequate blood volume.
  - Omit dose of phenoxybenzamine on morning of surgery and dose of propranolol can be given pending blood pressure.

*Anaesthetic management*

- It is strongly recommended that anaesthesia is supervised by an experienced paediatric anaesthesiologist alert to the diagnosis of a catecholamine-producing tumour [1].
- If indicated: premedication (midazolam or temazepam)
- Intravenous induction (propofol or thiopentone)
- Vecuronium
- Invasive blood pressure monitoring prior to or immediately after induction
- Lidocaine 1% to vocal cords prior to intubation
- Fentanyl or remifentanil (0.15–0.25 micrograms/kg/minute)
- Isoflurane or sevoflurane: O_2_ : air
- Central venous line (IJV)
- Monitoring: ECG, sao_2_, etco_2_, arterial blood pressure (ABP), central venous pressure (CVP), urine output, Temp, arterial blood gas
- Epidural for open surgical procedures
- Pharmacology
  - Hypertension – during tumour manipulation
    - Sodium nitroprusside (1 mg/mL) by infusion (0.1–2.0 micrograms/kg/minute)
    - Phentolamine (100 micrograms/kg)
    - Esmolol (5 mg loading over 2 minutes then 50 microgram/kg/minute)
  - Hypotension – following adrenal vein ligation and tumour isolation.
    - Stop hypotensive agents
    - Gelofusin 15 mL/kg
    - Noradrenaline infusion if necessary
    - Phenylephrine
    - Vasopressin

## Assessment of Patients on Admission

Admission assessment

- Clinical evaluation, including resting heart rate and postural blood pressure measurements.
- Book post-operative bed in paediatric intensive care unit (PICU)

Admission investigations

- Blood tests:
  - Full blood count, including haematocrit.
  - Coagulation screen
  - Crossmatch 1 unit
  - U&E’s (C127), liver function tests, glucose, ionised calcium, parathyroid hormone
- Urine tests:
  - Urine dipstick & urine albumin: creatinine ratio if proteinuria
  - U&E’s
  - 24-hour urinary metanephrines and catecholamines
- Cardiac investigations:
  - ECG (for evidence of tachycardia and/or arrhythmias)
  - Echocardiography (left ventricular hypertrophy & function)

## Surgical Technique

### Surgical biopsy

***There is no place for routine surgical biopsy in PPGL***, because of the risk of tumour spillage, poor diagnostic power to discriminate between benign from malignant tumours and as biopsy and manipulation of the tumour may provoke unnecessary hypertensive crises and haemodynamic instability [1].

### Tumour resection

Surgical excision after appropriate medical preparation is the first-line and principal treatment of PPGL. The core principle is complete tumour excision without rupture or breach of the tumour capsule and with minimal manipulation of the tumour, to minimise fluctuations in blood pressure. Intra-operative tumour spillage should be prevented at every cost. PPGL are often vascular tumours and the risk of intra-operative bleeding is not insignificant.

Minimal Invasive Surgical (MIS) techniques (laparoscopy, retroperitoneoscopy, thoracoscopy) are now widely regarded as the optimal surgical procedure for removing small to moderate sized PPGL (3–8 cm diameter). Due to potential bleeding risk, advanced MIS skills are a pre-requisite.

**Contraindications to a MIS approach include:**

- Previous abdominal or thoracic surgery
- Evidence of tumour thrombus within the adrenal vein and/or IVC
- Coagulation disorders
- A suspected diagnosis of adrenal carcinoma.

### Approach

#### Unilateral tumours

The installation of the patient and the surgical approach depend upon the specific location of the PPGL. For abdominal and especially adrenal PPGL, different MIS approaches have been described: the laparoscopic lateral transperitoneal (TP) and the retroperitoneoscopic (RP) approaches. The size and location of the tumour relative to the kidney and renal vessels should be studied carefully preoperatively and will serve as a guide to which approach, TP or RP, would be more favourable.

*1. The laparoscopic lateral transperitoneal (TP) approach:* used most frequently by general paediatric surgeons, it allows for intra-abdominal exploration, offers great working space and is especially useful in case of larger tumours. The modified lateral decubitus position provides excellent exposure as the abdominal contents are pulled away by gravity.

*Technique:*

The child is positioned in semi-lateral position at 60° angle with the tumour side up. The table is flexed to open the space between the 12th rib and the iliac crest. Port placement is open (under direct vision) with positioning depending on the side operated on: three ports are used for a left adrenalectomy and an additional port is used on the right side for liver retraction. Procedural steps include taking down the lateral colonic fixation, dissecting in the avascular plane between the tail of the pancreas and kidney on the left, and under the liver up to the inferior caval (IVC) and renal vein on the right. On the left, the adrenal vein is controlled with titanium or polymer clips just before it enters the left renal vein and divided. In a similar fashion, the shorter right adrenal vein is controlled before it enters the IVC and is divided. The arterial branches are controlled by clips or energy device. The tumour is extracted in an Endobag®.

*2. The retroperitoneoscopic (RP) approach:* (in lateral or prone position, used by paediatric urologists already familiar with this approach for renal surgery) may be advantageous particularly in smaller tumours, in case of prior intra-abdominal surgery.

*Technique:*

The child is positioned fully prone in a similar manner to a RP nephrectomy and the same landmarks and access technique are used to enter the retroperitoneum [30]. The dissection commences around the kidney and continues until the inferior margin of the tumour is visualised at the superomedial border of the kidney. The arterial blood supply to the adrenal is then identified and divided. To minimise bleeding from the surface of the gland, dissection is performed in a plane within the surrounding adipose tissue. Right sided tumour resection is more difficult than left side resection due to the proximity to the IVC and the short adrenal vein. Once the veins have been divided and tumour has been fully mobilised, it is placed within an Endobag® and removed through the camera port incision.

Opinion remains divided on whether the (L)TP or (P)RP approach is preferable. There are no reliable comparative data in children and most reports have consisted of small series. Tumour diameters exceeding 5 cm and also extra-adrenal locations (PGL) carry a higher risk for malignancy. This is not an absolute contraindication for a MIS approach. But, especially in this situation, if there is any risk that complete excision without tumour breach cannot be achieved by MIS, an open approach should be carried out [1].

*1. Special Considerations:*

- Bilateral tumours and partial adrenalectomy: Partial adrenalectomy or ‘adrenal preserving/cortical-sparing surgery’ may be considered for selected patients with bilateral tumours, following a personalised approach. If macroscopic complete and intact excision of the tumour can be safely performed without tumour breach, it is not always necessary to remove the entire adrenal gland. This may be indicated especially in patients who already underwent contralateral adrenalectomy. In MEN2 patients with bilateral adrenal PCC, bilateral total adrenalectomy should be performed, by open or MIS approach. In non-MEN2 patients, bilateral (or at least unilateral) cortical-sparing adrenalectomy should be considered [14]. Patients with hereditary/familial PPGL presenting with a unilateral PPGL should be treated by unilateral adrenalectomy (although at risk for metachronous disease contralaterally) [17].
- Malignant disease: The therapeutic strategy for metastatic PPGL primarily aims to control excessive catecholamine secretion and tumour burden, as there are no curative treatment options [1]. In all patients with metastatic PPGL (debulking) surgical resection of the primary tumour and/or metastatic lesions should be considered, on a case-by-case basis [1]. Cytoreductive debulking surgery (R2) may improve symptoms, quality of life and survival by reducing the tumour burden and controlling hormonal arterial hypertension [1, 18]. Systemic chemotherapy (cyclophosphamide- and dacarbazine-based regimens combined with vincristine and/or doxorubicin) may be indicated in patients with important tumour burden and progressive disease [1, 18, 19]. Targeted therapies with sunitinib and pazopanib are under investigation [1]. In selected patients with distant metastases, there may also be a place for systemic MIBG-treatment, radiation therapy or ablational techniques (radiofrequency, cryoablation, chemoembolisation) [1, 20, 21].

## Post-operative Management

- The aim is to extubate the patient at the end of the procedure
- Admission to PICU for haemodynamic monitoring
- Blood pressure usually returns to normal levels by the second postoperative day.

- Potential problems:
  - Hypotension
  - Fluid overload
  - Hypertension
  - Hypoglycaemia
- Analgesia: epidural or intravenous patient- or nurse-controlled analgesia

## Postoperative Outcome & Follow-up

### Cardiovascular risk

The increased cardiovascular morbidity and mortality associated are normalised or decreased after radical surgery.

Potential complications

- The surgical excision of PPGL may be complicated by blood loss, trauma to surrounding tissues (renal vessels, pancreas, IVC, other vessels or viscera) and wound infection. It is highly recommended to record all surgical details and intra- and postoperative complications, especially within the first 30 days after surgery. Adherence to the framework of the ‘International Neuroblastoma Surgical Report Form
- (INSRF)’ [22], a multi-committee standardised reporting system for the surgery of neuroblastic tumours is encouraged because of the analogy in localisation (adrenal, retroperitoneal, pelvic, mediastinal) between PPGL and neuroblastic tumours.
- Surgical manipulation of the PPGL may cause intra-operative hypertensive crises and arrhythmias due to hormone release: avoiding excessive tumour manipulation by minimal touch technique is essential.
- Postoperative hypotension may be due to chronic vasoconstriction.
- Patients with tumours of > 5 cm diameter are particularly at risk for malignancy, haemodynamic problems and more severe postoperative complications [1, 23]. Low-threshold conversion is necessary if MIS dissection cannot be performed safely or if complete resection cannot be performed without undue trauma to the tumour or gland [1, 24].
- Adrenal insufficiency in case of bilateral adrenalectomy may be avoided by adrenal sparing surgery upon indication (see above).

### Recurrence

There is a recurrence rate of 10%–12%, although in some cases this is due to inadequate primary clearance: especially after cortical sparing/adrenal preserving adrenalectomy, recurrence rates may be between 10% and 38% [9, 25, 26]. Surgical excision of the recurrent tumour may give at least temporary cure – as some children may have multiple recurrences, especially in case of hereditary/familial disease [27, 28].

### Surveillance

Every paediatric PPGL patient should have genetic analysis performed.

- All patients with resected PPGL should be followed at regular intervals for at least 10 years and lifelong in case of germline mutation [1].
- If a pathogenic variant is found in SDHA, SDHB, SDHC, TMEM127 or MAX, all adult first-degree relatives are recommended targeted testing of the family’s mutation on DNA. Other children are only offered pre-symptomatic genetic testing if they would be recommended surveillance [3].
- Healthy first-degree relatives (and second-degree relatives in the case of SDHD and SDHAF2, which are maternally imprinted) should be offered carrier testing.
- Carriers of pathogenic variants should be offered surveillance with annual biochemical measurements of methoxy-catecholamines and bi-annual rapid whole-body MRI and clinical examination. Surveillance should start 5 years before the earliest age of onset in the family. The surveillance of children younger than 15 years needs to be individually designed [3].
- For children with VHL and Hereditary paraganglioma and pheochromocytoma syndrome, detailed surveillance guidelines have been published in 2017 [29].

## References

[1] Fassnacht M, Assie G, Baudin E (2020). Adrenocortical carcinomas and malignant phaeochromocytomas: ESMO-EURACAN Clinical Practice Guidelines for diagnosis, treatment and follow-up. Ann Oncol.

[2] Linet MS, Ries LA, Smith MA (1999). Cancer surveillance series: recent trends in childhood cancer incidence and mortality in the United States. J Natl Cancer Inst.

[3] Muth A, Crona J, Gimm O (2019). Genetic testing and surveillance guidelines in hereditary pheochromocytoma and paraganglioma. J Intern Med.

[4] Babic B, Patel D, Aufforth R (2017). Pediatric patients with PPGL should have routine preoperative genetic testing for common susceptibility genes in addition to imaging to detect extra-adrenal and metastatic tumours. Surgery.

[5] Ciftci AO, Tanyel FC, Şenocak ME (2001). Pheochromocytoma in children. J Pediatr Surg.

[6] Lenders JW, Eisenhofer G, Mannelli M (2005). Phaeochromocytoma. Lancet.

[7] Kim HJ, Kim MJ, Kong SH (2020). Characteristics of germline mutations in Korean patients with pheochromocytoma/paraganglioma. J Med Genet.

[8] Thompson LDR (2002). Pheochromocytoma of the adrenal gland scaled score (PASS) to separate benign from malignant neoplasms: a clinicopathologic and immunophenotypic study of 100 cases. Am J Surg Pathol.

[9] Pham TH, Moir C, Thompson GB (2006). Pheochromocytoma and paraganglioma in children: a review of medical and surgical management at a tertiary care centre. Pediatrics.

[10] Fishbein L, Leshchiner L, Walter V (2017). Cancer Genome Atlas Research Network Comprehensive molecular characterization of pheochromocytoma and paraganglioma. Cancer Cell.

[11] Dahia PL (2014). Pheochromocytoma and paraganglioma pathogenesis: learning from genetic heterogeneity. Nat Rev Cancer.

[12] Neumann HPH, Bausch B, McWhinney SR (2002). Germ-line mutations in nonsyndromic pheochromocytoma. N Engl J Med.

[13] Neumann HPH, Young WF, Eng C (2019). Pheochromocytoma and paraganglioma. N Engl J Med.

[14] Neumann HPH, Tsoy U, Bancos I (2019). Comparison of Pheochromocytoma specific morbidity and mortality among adults with bilateral pheochromocytomas undergoing total adrenalectomy vs cortical-sparing adrenalectomy. JAMA Netw Open.

[15] Favier J, Amar L, Gimenez-Roqueplo AP (2015). Paraganglioma and phaeochromocytoma: from genetics to personalized medicine. Nat Rev Endocrinol.

[16] Lenders JW, Duh QY, Eisenhofer G (2014). Pheochromocytoma and paraganglioma: an endocrine society clinical practice guideline. J Clin Endocrinol Metab.

[17] Evans DB, Lee JE, Merrell RC (1994). Adrenal medullary disease in multiple endocrine neoplasia type 2 Appropriate management. Endocrinol Metab Clin North Am.

[18] Ein SH, Weitzman S, Thorner P (1994). Pediatric malignant pheochromocytoma. J Pediatr Surg.

[19] Young WF (2020). Metastatic pheochromocytoma: in search of a cure. Endocrinology.

[20] Mcbride JF, Atwell TD, Charboneau WJ (2011). Minimally invasive treatment of metastatic pheochromocytoma and paraganglioma: efficacy and safety of radiofrequency ablation and cryoablation therapy. J Vasc Interv Radiol.

[21] Shapiro B, Sisson JC, Wieland DM (1991). Radiopharmaceutical therapy of malignant pheochromocytoma with 131I-MIBG: results from ten years of experience. J Nucl Biol Med.

[22] Matthyssens LE, Nuchtern JG, Van De Ven CP (2020). A novel standard for systematic reporting of neuroblastoma surgery: the international neuroblastoma surgical report form (INSRF): a joint initiative by the pediatric oncological cooperative groups SIOPEN*, COG** and GPOH***. Ann Surg.

[23] Butz JJ, Yan Q, McKenzie TJ (2017). Perioperative outcomes of syndromic paraganglioma and pheochromocytoma resection in patients with von Hippel-Lindau disease, multiple endocrine neoplasia type 2, or neurofibromatosis type. Surgery.

[24] Stefanidis D, Goldfarb M, Kercher KW (2013). SAGES Guidelines for minimally invasive treatment of adrenal pathology. Surg Endosc.

[25] Beltsevich DG, Kuznetsov NS, Kazaryan AM (2004). Pheochromocytoma surgery: epidemiologic peculiarities in children. World J Surg.

[26] Asari R, Scheuba C, Kaczirek K (2006). Estimated risk of pheochromocytoma recurrence after adrenal-sparing surgery in patients with MEN type 2A. Arch Surg.

[27] Ein SH, Shandling B, Wesson D (1990). Recurrent pheochromocytomas in children. J Pediatr Surg.

[28] Ein SH, Pullerits J, Creighton R (1997). Pediatric Pheochromocytoma A 36-year review. Pediatr Surg Int.

[29] Rednam SP, Erez A, Druker H (2017). Von Hippel-Lindau and Hereditary Pheochromocytoma/paraganglioma syndromes: clinical features, genetics, and surveillance recommendations in childhood. Clin Cancer Res.

[30] Mushtaq I, Spitz L, Coran A (2013). Nephrectomy and partial nephrectomy. Operative Pediatric Surgery.

# Discussion: Non-Germ Cell Gonadal Tumours

**Published:** 2022

**Authors:** Patrizia Dall’Igna, Calogero Virgone

## Evaluation

### Epidemiology

Sex cord stromal tumours (SCSTs) represent a heterogeneous group of rare gonadal tumours. Overall, SCSTs represent approximately 10% of all gonadal tumours during childhood [1]. They develop from the non-germ cell component of the ovary or testis. The rarity of these tumours associated with the heterogeneity and difficulty in the correct histopathologic classification has left a significant uncertainty with regard to the correct clinical approach to patients with those tumours. Of note, some subtypes of SCSTs are associated with constitutional genetic aberrations (e.g. *DICER-1* mutations) and thus, they are part of an underlying cancer predisposition [2].

Ovarian epithelial tumours are rare in childhood, accounting for less than 1% of all paediatric cancers and their incidence increases after menarche. Various histological types are recognised and classified according to the predominant histologic features (serous, mucinous, clear cell, endometrioid or undifferentiated), and serous and mucinous tumours are the most frequent in the paediatric age group [3]. In this group, the small cell carcinoma of the hypercalcaemic type (SCCOHT) represents the rarest form [4].

### Clinical presentation

Boys: Patients with testicular SCSTs characteristically present with indolent intra-testicular mass. Apart from benign disorders such as varicocele, cysts, etc., malignant germ cell tumours and teratomas present the most relevant differential diagnosis. However, a hormonal secretion may be present, and some prepubertal boys may present an isosexual precocious puberty. Genetic counselling should be performed based on individual and familial history: in these cases, Peutz–Jeghers syndrome (large cell-calcifying Sertoli Tumours) or DICER1 syndrome (SLCTs) should be ruled out [5–7].

Girls: Patients with ovarian SCSTs characteristically present with indolent clinical tumour/abdominal distension and may often suffer from abdominal discomfort and pain. In addition, hormonally active tumours may present with signs of precocious puberty such as breast swelling, pubic hair, vaginal bleeding – characteristic of oestrogen secreting granulosa cell tumours – or virilisation and hirsutism – characteristic of androgen secreting SLCTs. Some patients may present with acute abdomen, caused by adnexal/ovarian torsion. Malignant germ cell tumours and teratomas present the most relevant differential diagnosis. SCST are different from germ cell tumours because of their clinical presentation and their biology including associated genetic tumour predisposition syndromes. Ovarian SLCTs may be part of a DICER1 syndrome; in these cases, genetic counselling and thereafter an additional screening aimed to exclude/detect a thyroid tumour are recommended [7–12]. Patients with ovarian cystadenoma, cystadenocarcinoma or SCCOHT usually present with indolent clinical tumour/abdominal distension and may often suffer from abdominal discomfort and pain. Some patients may present with acute abdomen, caused by adnexal/ovarian torsion. Girls affected by SCCOHT may present a mutation in the *SMARCA4* gene: in these cases, a genetic testing should always be performed [3, 4, 12–14].

### Workup

**Lab:**

Complete blood count, complete metabolic profile and coagulation profile.

Markers:

- Testis SCST: Serum AFP, Serum β-HCG, human alkaline placenta like phosphatase, Serum inhibin (R), oestrogen/testosterone (in case of endocrine symptoms)
- Ovarian SCST, epithelial ovarian carcinoma, SCCOHT: CA125, anti-Mullerian hormone, Dehydroepiandrosterone sulphate (DHEAS)/testosterone/luteinizing hormone/folicule stimulating hormone/oestradiol, calcaemia, chromogranin-A and neuron specific enolase.

**Imaging:**

- Testis SCST: scrotal US scan, abdominal US and MRI.
- Ovarian tumours: abdominal US, abdominal MRI, chest CT scan.

## Perioperative Management

### Role and timing of multimodality therapy

Neo-adjuvant and adjuvant treatment such as chemotherapy or radiotherapy are indicated in advanced stages. While some recommendations exist for SCST in children and adolescents [10], epithelial carcinomas and SCCOHT are usually treated according to the guidelines delineated by adult gynaecologist-oncologists or with their collaboration [2, 4].

### Preoperative considerations

Preoperative multidisciplinary planning should include the assessment of co-morbidities, magnitude of the operation, capacity of the anaesthesia team, intraoperative monitoring, reliable upper extremity vascular access, urinary catheter, availability of blood, appropriate allocation of postoperative level of care and monitoring and postoperative pain control. If a neoadjuvant chemotherapy protocol is used, surgery should follow blood count recovery.

### Surgery

#### Testicular Sex Cord Stromal Tumours

Since virtually all testicular sex cord tumours in children and adolescents present as localised tumours, tumour resection will constitute the only therapy of these tumours. In principle, orchiectomy after high inguinal incision and first ligation of the spermatic cord constitutes the gold standard [8, 9]. Taking into consideration the patient’s potential wish, a testicular prothesis can be inserted during the same surgical session.

Despite the overall favourable prognosis, there has been some debate as to whether tumour excision after scrotal excision or organ sparing surgery (e.g. enucleation of the tumour) may also be appropriate. These strategies have not yet been validated prospectively, and it is unclear whether organ sparing surgery may indeed contribute to further reproductive function and quality of life [15]. However, in case of complete but organ sparing enucleation of a testicular SCST with an inguinal approach, a second look surgery and orchidectomy is not mandatory, at least in prepubertal children with non-metastatic tumours. Moreover, in the same group of patients a second look surgery in case of trans-scrotal surgery is not considered mandatory. Organ sparing surgery such as tumour enucleation should be attempted as an individual approach in non-metastatic bilateral tumours.

Biopsy of an unsuspicious (palpation and US) contralateral testis is not required.

Retroperitoneal lymph node dissection is not routinely recommended; however it should be considered in the rare cases of suspicious involvement based on US/MRI.

The extremely rare metastatic tumour should be treated according to the corresponding concept for stage IV ovarian SCST [10].

#### Ovarian Sex Cord Stromal Tumours

Even in large tumours, surgical resection is the first therapeutic step, and biopsy should be avoided. Diagnosis is mainly based on the clinico-imaging presentation and the elevated tumour markers [10].

Since most tumours present as localised stage Ia tumours, tumour resection by oophorectomy or adnexectomy will constitute the only therapy of these tumours [5, 10].

Median laparotomy constitutes the standard surgical approach in adults, but in children also a sub-umbilical transverse or a Pfannenstiel incisions can be used, depending on the size of the tumour. Both allow a good exposure and a better cosmetic result. In case of small tumours, laparoscopic resection may be performed by experienced hands [10], provided it follows an oncologically safe criteria, i.e. tumour rupture, spillage or any other violation of the tumour capsule must be avoided stringently [16-18].

Staging includes cytological evaluation of ascites and/or peritoneal fluid, inspection and palpation of the contralateral ovary, inspection of the peritoneal cavity, omental biopsy, careful evaluation of the pelvis and Douglas pouch and inspection and biopsy of any suspicious lymph nodes.

Oophorectomy should be performed in case of tumours confined to the ovary; in case of pelvic adhesion/infiltration ipsilateral fallopian tube, adnectomy has to be performed [16] (Figure 1).

In case of adhesions to the omentum, omentectomy is recommended; routine omentectomy is not required, if unsuspicious.

In case of bilateral tumours, ovary sparing tumour resection may be performed as an individual approach and performed by the appropriate expertise, since bilateral ovariectomy is mutilating and should be considered only in selected cases without response to chemotherapy [16]. Routine retroperitoneal lymph node dissection is not recommended and biopsy of an unsuspicious (palpation and MRI/US) contralateral ovary is not required.

#### Ovarian Serous/Mucinous Cystadenoma (benign and borderline tumours) (Figure 2)

Surgery is the cornerstone of treatment for benign and borderline tumours (BOTs) [3]. Moreover, taking into account the rarity of malignant neoplasms, in the absence of suspicion at preoperative investigations and intraoperative evaluation, the goals of the surgeon should be the preservation of as much as possible of the normal ovarian tissue and the prevention of adhesion, especially when a bilateral tumour is encountered. In the past years, fertility-sparing surgery has been considered as the gold standard of treatment for epithelial benign and borderline masses [19–22].

The need to perform a complete surgical staging in adults has been debated, being the contralateral ovary biopsy and the omental biopsy at risk for adhesion formation. In addition, some authors questioned the utility of a complete surgical staging, stating that it could be omitted for stage I BOTs, being at low risk for local or distant recurrences, when surgical treatment is performed in a centre where a frozen section analysis is available [23–25].

Fertility-sparing procedures in BOTs showed higher rate of relapses, but this does not seem to affect the OS. These data, together with the evidence that chemotherapy has a limited role in the management of these tumours, allows the avoidance of unjustified aggressive surgeries [26].

#### Ovarian Serous/Mucinous Cystadenocarcinoma

The treatment of malignant tumours, rarely encountered by both paediatric surgeon and oncologist, should follow guidelines and protocols in use for adult patients, and the referral of those patients to tertiary gynae-oncologic centres for further discussion should be encouraged [3, 15].

#### Ovarian Small Cell Carcinoma of the Hypercalcaemic Type (SCCOHT)

SCCOHT are very rare but highly malignant neoplasm. Even in children and adolescent, a more aggressive surgical approach is suggested by most authors [4].

*Early stages:* When the pathological diagnosis is ensured, surgical treatment includes total abdominal hysterectomy and bilateral salpingo-oophorectomy with peritoneal staging and full pelvic and para-aortic lymphadenectomy also for macroscopically stage I patients, because of poor prognosis, the high risk of extra-ovarian spread and the loss of function of the contralateral ovary after intensive adjuvant treatment [4].

*Advanced/metastatic/relapsed disease:* Removal of peritoneal disease (debulking surgery) including omentectomy and pelvic and para-aortic lymphadenectomy, if complete removal of the peritoneal disease can be achieved, is recommended (as initial surgery or after three to six cycles of chemotherapy; grade D) [4].

## Prognosis, Prognostics and Follow-up

Prognosis may vary according to histology and stage at diagnosis. Testicular SCST have an excellent outcome, as well as benign and borderline epithelial tumours. Among ovarian SCSTs, prognosis is excellent in the majority of juvenile granulosa cell tumour, while it is worse in advanced SLCT. Advanced cystadenocarcinoma and SCCOHT often have a dismal outcome. Survivors should be elected to long-term follow-up aimed to monitor the fertility and to detect contralateral metachronous tumours (reported to be up to 10%–20% in girls) as earlier as possible [3, 4, 8–10, 27].

## Figures and Tables

**Figure 1.** Treatment strategy for ovarian and testicular SCST proposed by the ExpERT group.

**Figure 2.** Strategy for benign and borderline epithelial ovarian neoplasm proposed by the Italian TREP project (Virgone et al [3]).

## References

[1] Schneider DT, Terenziani M, Cecchetto G, Schneider DT, Brecht IB, Olson TA (2012). Gonadal and extragonadal germ cell tumors, sex cord stromal tumors and rare gonadal tumors. Rare Tumors in Children and Adolescents.

[2] Schultz KA, Pacheco MC, Yang J (2011). Ovarian sex cord-stromal tumors, pleuropulmonary blastoma and DICER1 mutations: a report from the International Pleuropulmonary Blastoma Registry. Gynecol Oncol.

[3] Virgone C, Alaggio R, Dall’Igna P (2015). Epithelial tumors of the ovary in children and teenagers: a prospective study from the Italian TREP Project. J Pediatr Adolesc Gynecol.

[4] Sessa C, Schneider DT, Planchamp F (2020). ESGO-SIOPE guidelines for the management of adolescents and young adults with non-epithelial ovarian cancers. Lancet Oncol.

[5] Cecchetto G, Alaggio R, Bisogno G (2010). Sex cord-stromal tumors of the testis in children. A clinicopathologic report from the Italian TREP project. J Pediatr Surg.

[6] Fresneau B, Orbach D, Faure-Conter C (2015). Sex-cord stromal tumors in children and teenagers: results of the TGM-95 study. Pediatr Blood Cancer.

[7] Schultz KA, Schneider DT, Pashankar F (2012). Management of ovarian and testicular sex cord-stromal tumors in children and adolescents. J Pediatr Hematol Oncol.

[8] Schneider DT, Jänig U, Calaminus G Ovarian sex cord-stromal tumors--a clinicopathological study of 72 cases from the Kiel Pediatric Tumor Registry. Virchows Arch.

[9] Cecchetto G, Ferrari A, Bernini G (2011). Sex cord stromal tumors of the ovary in children: a clinicopathological report from the Italian TREP project. Pediatr Blood Cancer.

[10] Schneider DT, Orbach D, Ben-Ami T (2021). Consensus recommendations from the EXPeRT/PARTNER groups for the diagnosis and therapy of sex cord stromal tumors in children and adolescents. Pediatr Blood Cancer.

[11] Rio Frio T, Bahubeshi A, Kanellopoulou C DICER1 mutations in familial multinodular goiter with and without ovarian Sertoli-Leydig cell tumors. JAMA.

[12] Goudie C, Witkowski L, Vairy S Paediatric ovarian tumours and their associated cancer susceptibility syndromes.

[13] Nasioudis D, Alevizakos M, Holcomb K (2017). Malignant and borderline epithelial ovarian tumors in the pediatric and adolescent population. Maturitas.

[14] Pastorczak A, Krajewska K, Urbanska Z (2021). Ovarian carcinoma in children with constitutional mutation of SMARCA4: single-family report and literature review. Fam Cancer.

[15] Tröbs RB, Krauss M, Geyer C (2007). Surgery in infants and children with testicular and paratesticular tumours: a single centre experience over a 25-year-period. Klin Padiatr.

[16] Schneider DT, Orbach D, Cecchetto G (2015). Ovarian Sertoli Leydig cell tumours in children and adolescents: an analysis of the European Cooperative Study Group on Pediatric Rare Tumors (EXPeRT). Eur J Cancer.

[17] Schneider DT, Calaminus G, Göbel U (2004). Tumor rupture and mitotic index in pediatric sex cord-stromal tumors. J Clin Oncol.

[18] Schneider DT, Calaminus G, Wessalowski R (2003). Ovarian sex cord-stromal tumors in children and adolescents. J Clin Oncol.

[19] Tsai JY, Saigo PE, Brown C (2001). Diagnosis, pathology, staging, treatment, and outcome of epithelial ovarian neoplasia in patients age ! 21 years. Cancer.

[20] von Allmen D (2005). Malignant lesions of the ovary in childhood. Semin Pediatr Surg.

[21] Abdel-Hady el-S, Abdel-Hady HR, Gamal A (2012). Fertility sparing surgery for ovarian tumors in children and young adults. Arch Gynecol Obstet.

[22] Reddy J, Laufer MR (2009). Advantage of conservative surgical management of large ovarian neoplasms in adolescents. Fertil Steril.

[23] Cho YH, Kim DY, Kim JH (2006). Is complete surgical staging necessary in patients with stage I mucinous epithelial ovarian tumors?. Gynecol Oncol.

[24] Winter WE, Kucera PR, Rodgers W (2002). Surgical staging in patients with ovarian tumors of low malignant potential. Obstet Gynecol.

[25] Romeo M, Pons F, Barretina P (2013). Incomplete staging surgery as a major predictor of relapse of borderline ovarian tumor. World J Surg Oncol.

[26] Hazard FK, Longacre TA (2013). Ovarian surface epithelial neoplasms in the pediatric population: incidence, histologic subtype, and natural history. Am J Surg Pathol.

[27] Schultz KA, Harris AK, Schneider DT (2016). Ovarian sex cord-stromal tumors. J Oncol Pract.

# Discussion: Colorectal Carcinoma

**Published:** 2022

**Authors:** Aodhnait S. Fahy, Reto Baertschiger

## Incidence

Colorectal cancer is very uncommon in paediatric patients, in contrast to adults. It is the most common gastrointestinal malignancy in this population. Colorectal cancer has a very poor prognosis in children [1, 2].

The incidence in patients under 18 years is approximately 1 in 1 million [3], and prevalence approximately 0.2% [2, 4].

The predominant histologic subtype is adenocarcinoma but there is a high percentage of signet ring or anaplastic lesions, and an increased proportion of mucinous tumours [5, 6].

The polyposis syndromes including FAP syndrome as well as inflammatory bowel disease markedly increase paediatric patient risks [7], however overall do not represent the majority of cases. FAP syndrome is secondary to adenomatous autosomal dominant genetic disease in the APC gene on chromosome 5. For patients with FAP, a screening colonoscopy is indicated by the age of 10, and prophylactic total procto-colectomy with ileal-pouch anal anastomosis is recommended to reduce the almost 100% risk of these patients developing a colorectal cancer [7]. Children suffering from inflammatory bowel disease, more specifically ulcerative colitis, are the other subset of patients at high risk of developing colorectal cancer [8]. Patients with ulcerative colitis should undergo endoscopic screening within 8 years of diagnosis, or sooner if concerns, based on imaging or symptoms [9].

## Clinical Presentation

The patients’ symptoms are most frequently associated with anaemia secondary to haematochezia, and some patients present occasionally with a palpable mass, abdominal pain or present with intestinal obstruction as emergencies [10]. Unfortunately, patients often present with advanced disease because of late diagnosis secondary to ignoring initial symptoms or absence of screening [1, 2, 10].

## Diagnosis

A detailed history and physical exam including family history and rectal exam are recommended. Workup should include colonoscopy, axial imaging for staging and genetic testing particularly as the identification of high-risk individuals and families can allow for excellent outcomes [11].

## Management

Surgery is the first and crucial step in the management of paediatric colorectal cancer. This can either be undertaken prophylactically after the diagnosis of polyposis syndromes, or as segmental resections with lymphovascular control, with a similar approach to adult colorectal cancer surgery. Neo-adjuvant chemotherapy should be considered for stage 4 disease. Adjuvant therapy follows the National Comprehensive Cancer Network (NCCN) guidelines [12, 13].

Furthermore, education of families with pre-disposing syndromes and the importance of regular screening colonoscopy is cornerstone of management.

## Prognosis

Children and adolescents with colorectal cancer present with more advanced disease when compared to their adult counterparts. Their prognosis is significantly worse when compared with adult patients – with reports of 65%–100% 5-year mortality [2, 10]. Survival rates in the SEER database were 40% and 31% at 5 and 10 years, respectively, for children under 14. This contrasts significantly with adult patients whose outcomes are 60% and 54% at 5 and 10 years, respectively, in the same dataset [5]. More recent studies have replicated these outcomes with a 3-year OS and event-free survival (EFS) of 42% and 32 % in the paediatric population [2].

## References

[1] Attard TM, Lawson CE (2019). Comparison of the demographic characteristics of pediatric and adult colorectal cancer patients: a national inpatient sample- based analysis. World J Pediatr.

[2] Hayes-Jordan AA, Sandler G, Malakorn S (2020). Colon cancer in patients under 25 years-old: a different disease?. J Am Coll Surg.

[3] Blumer SL, Anupindi SA, Adamson PC (2012). Sporadic adenocarcinoma of the colon in children: case series and review of the literature. J Pediatr Hematol Oncol.

[4] Meyer JE, Narang T, Schnoll-Sussman SH (2010). Increasing incidence of rectal cancer in patients aged younger than 40 years: an analysis of the surveillance, epidemiology, and end results (SEER) database. Cancer.

[5] Sultan I, Rodriguez Galindo C, El-Taani H (2010). Distinct features of colorectal cancer in children and adolescents: a population-based study of 159 cases. Cancer.

[6] Ballester V, Rashtak S, Boardman L (2016). Clinical and molecular features of young-onset colorectal cancer. World J Gastroenterol.

[7] Fahy AS, Moir CR (2018). Current approaches to pediatric polyposis syndromes. Clin Colon Rectal Surg.

[8] Kayton ML (2007). Cancer and inflammatory bowel disease. Semin Pediatr Surg.

[9] Orlanski-Meyer E, Topf-Olivestone C, Ledder O (2020). Outcomes following pouch formation in pediatric ulcerative colitis: a study from the Porto Group of ESPGHAN. J Pediatr Gastroenterol Nutr.

[10] Kim G, Baik SH, Lee KY (2013). Colon carcinoma in childhood: a review of the literature with four case reports. Int J Colorectal Dis.

[11] Ferrari A, Rognone A, Casanova A (2008). Colorectal carcinoma in children and adolescents: the experience of the Instituto Nazionale Tumori of Milan, Italy. Pediatr Blood Cancer.

[12] Chan GHJ, Chee CE (2019). Making sense of adjuvant chemotherapy in colorectal cancer. J Gastrointest Oncol.

[13] Jeffery M, Hickey BE, Hider PN (2019). Follow-up strategies for patients treated for non-metastatic colorectal cancer. Cochrane Database Syst Rev.

# Discussion: Adrenocortical Tumours

**Published:** 2022

**Authors:** Patrizia Dall’Igna, Vilani Kremer, Ivan Dario Molina Ramirez, Calogero Virgone

## Background

Paediatric adrenocortical tumours (ACTs) are rare but potentially aggressive endocrine malignancies. They are frequently associated with Li–Fraumeni syndrome, a familial cancer predisposition disorder caused by germline pathogenic variant in the tumour suppressor gene TP53 [1–3].

The treatment guidelines for these tumours in Europe were developed inside the EXPeRT, but similar guidelines were also developed inside the major International Cooperative Groups [4].

ACTs comprise benign adenoma (ACA) and highly malignant adrenocortical carcinoma (ACC). Only about 20% of paediatric ACTs are classified as ACA and are associated with excellent prognosis. However, the distinction between adenoma and carcinoma is difficult both at the clinical and histopathologic level. The histologic Wienecke index has been reported to show a stronger prognostic predictive value compared to other histologic prognostic scores that used in adults [5–8]. Nevertheless, the prognostic stratification of paediatric ACTs is still challenging due to their rarity, variable presentation and difficulty in histologic definition. Overall, the 5-year survival for children with ACTs depends on the stage and histology. It may vary from more than 80% for patients with small localised resected ACT to less than 20% for patients with metastatic ACC (10%–33% of all cases) [5, 9, 10].

## Epidemiology

ACTs in childhood represent about 0.2% of all paediatric malignancies. The incidence varies across geographic regions and is remarkably high in southern Brazil [11, 12].

The higher incidence observed in the Brazilian population has been linked to a specific pathogenic variant of TP53 gene (unique founder TP53 pathogenic variant Arg337His) which leads to the onset of ACC limited to the paediatric age [1].

The female/male ratio is 2/1 and the age incidence curve is characterised by two peaks, the first under 3 years, the second during adolescence [5, 13].

## Clinical Presentation

Because paediatric ACTs are almost universally functional, they cause endocrine disturbances, and a diagnosis is usually made 5–8 months after the first signs and symptoms emerge [6, 10].

- Virilisation (pubic hair, accelerated growth, enlarged penis, clitoromegaly, hirsutism and acne) caused by an excess of androgen secretion is detected, alone or in combination with hypercortisolism, in more than 80% of patients
- Hyperestrogenism
- Cushing syndrome. Isolated Cushing syndrome is very rare (5% of patients), and it appears to occur more frequently in older children
- Pain and fatigue

Because of the hormone hypersecretion, it is possible to establish an endocrine profile for each particular tumour, which may facilitate the evaluation of response to treatment and monitor for tumour recurrence. Nonfunctional tumours are rare (<10%) and tend to occur in older children.

## Workup

- Complete blood count, complete metabolic profile, coagulation profile
- Hormonal assessment:
  - Glucocorticoid excess:
  - Dexamethasone suppression test
  - Free 24-hours cortisoluria (with creatininuria)
  - Basal ACTH (plasma)

Sex steroids and steroid precursors excess:

- DHEA-S (serum)
- 17-OH-Progesterone (serum)
- (Delta-4-) Androstenedione (serum)
- Testosterone (in both sex) (serum)
- 17-beta-oestradiol (in both sex) (serum)
- 11-Deoxycortisol
- Estrone (E1) and oestradiol (E2)
- Mineralocorticoid excess:
- Aldosterone
- Natraemia, Kalaemia (plasma), Natriuresis
- Aldosterone/renin ratio (in patients with hypertension and/or hypokalaemia)
- Urinary metanephrines and catecholamines

## Imaging

### Primary tumour and loco-regional tumour extension

All ACTs must be evaluated by:

- Pelvic and abdominal US with Doppler
- Pelvic/abdominal MRI or
- CT

The imaging finding should be discussed in a MDT meeting, including the following disciplines: paediatric oncology, endocrinology, pathology, radiology, surgery, in order to evaluate the possible local invasion and the regional nodes involvement. This is imperative for planning the surgical excision of the primary tumour or to choose a preoperative chemotherapy followed by delayed resection.

### Distant metastases

Considering that the most common sites of metastatic disease in paediatric ACC are liver and lungs, metastatic workup should be primarily focused on these sites to confirm or exclude the presence of the metastatic disease:

- Chest CT scan is necessary to identify or exclude possible lung metastases
- PET scan or PET-MRI to identify unusual sites of metastases
- Bone CT scan (in the absence of PET scan) should be limited to cases where the clinical suspicion of bone metastasis is present
- Brain MRI should be performed when cerebral metastases are clinically suspected or in cases with suspicious/proven Li–Fraumeni syndrome

## Additional Assessments

- It is reasonable to propose a genetic counselling preoperatively, but it could be delayed after surgical treatment if there is no high clinical suspicion of cancer predisposition disorder
- Cardiac US in case of vascular and diaphragmatic involvement seems useful

## Diagnosis

In most cases, clinical diagnosis can be established based on typical tumour site, hormone profile and exclusion of other tumours in differential diagnosis (neuroblastoma). However, final histopathological evaluation is mandatory after tumour resection to allow for confirmation of diagnosis and histological stratification of ACT.

### Biopsy

The initial biopsy should be strongly discouraged in all patients as it is considered as a violation of the capsule resulting in an upstaging up to stage IV, turning a potentially curable tumour into a fatal disease [8, 14–16].

## Surgery

### Primary tumour

Different series, although with a limited number of patients, have shown that complete tumour resection is a major prognostic factor. Therefore, an upfront complete ‘en-bloc’ resection is the cornerstone of treatment. Surgical resection of ACT should be performed by surgeons experienced in adrenal and oncological surgery aiming at a complete resection, considering that surgery alone may, in most cases, cure the patient.

The incidence of intraoperative tumour ruptures has been evaluated in paediatric series at 20% at the time of the initial excision, and more than 40% in the case of surgery on a locoregional recurrence [10, 11]. The rupture is responsible for tumour spread in the peritoneal cavity, increasing the risk of loco-regional recurrence, with dismal outcome [8, 10].

### Key steps of surgery

- The preferred surgical approach should be an open approach through a right or left transverse TP incision or a midline laparotomy in case of exceptionally huge masses [17]
- The tumour should be removed without rupture and a systematic regional node sampling should always be performed
- Nephrectomy is accepted when the tumour cannot be separated from the kidney with tumour free margins. The presence of a thrombosis of the vena cava (COG stage III) does not necessarily categorise the tumour as inoperable although it complicates the surgical procedure, because of the risk of tumour embolism during the manipulation of the vena cava and the increased risk of intraoperative tumour rupture.

Recently, a MIS, both laparoscopic and robotic MIS to ACA has been discussed for infants and pre-school children with small non-infiltrating, non-metastatic tumours, showing similar results both in terms of rate of complications and oncological outcome in some studies [18–21]. However, mini-invasive surgery is strongly discouraged when malignancy is suspected. In particular, tumours with a volume exceeding 200 cm3 (or >5 cm) and/or suspicious regional nodal involvement and/or signs of local invasion should always be resected using an open laparotomy, with no exceptions.

## Metastases

For what concerns metastatic sites, an aggressive approach combining aggressive (complete) surgery with neoadjuvant and adjuvant chemotherapy plus mitotane, is recommended and should be aimed at the clearance of the primary tumour and metastatic sites as much as possible. Surgery of metastatic sites should however be reserved to patients who are in good clinical condition and when a reasonable clearance of metastatic sites is deemed feasible. Metastases may be alternatively approached at the time of resection of the primary tumour or delayed to a second operation.

## Preoperative considerations

The anaesthetic management of patients with hypercortisolism at diagnosis or during hormone supplementation due to the adrenal insufficiency associated with mitotane must be carried out in collaboration with the paediatric endocrinology team. The risk of acute adrenal decompensation at the time of surgical tumour removal should be considered.

## Role and timing of multimodality therapy

The decision on immediate surgical resection should be evaluated in large tumours with a high risk of intraoperative rupture. In these, a preoperative chemotherapy (including mitotane) based on clinical diagnosis followed by delayed resection should be the preferred strategy. The most important risk factors are the completeness of resection and avoidance of spillage at diagnosis. In huge, advanced tumours and those with locoregional metastases, spillage also has to be avoided, which plead for preoperative chemotherapy in typical clinical and biological presentation, without any biopsy.

## Staging

Staging should follow the COG system.

The most widely used staging system in childhood has been proposed by Sandrini *et al* [11], and has been later modified. This is a post-surgical staging system, which relies on the possibility and the quality of resection, tumour size, regional node involvement and presence of metastatic disease.

The COG system (Table 1) has been adopted by the French VRT FRACTURE Group and, with some modifications, by the Italian TREP project. It is recommended to use the COG system to allow National and International referral, comparison of data and a proper paediatric stratification.

## Postoperative Considerations

Histology after tumour resection is mandatory, and revision of histological slides from pathologists with proven experience in paediatric tumours is highly recommended. Differentiating benign from malignant ACT is often difficult, and adult scores (Weiss, Hough, Van Slooten) have been demonstrated to be poorly predictive, especially in younger children. Later, the Wienecke index (first described in 2003) [6, 7] has been reported to have a prognostic value that is more reliable compared to other histologic prognostic scores used in adults [5, 7, 8, 12].

This index classifies ACTs in three prognostic groups (benign, malignant and of undetermined malignant potential) on the basis of the number of the following pathologic criteria: tumour size, tumour weight, extension into periadrenal soft tissues and/or adjacent organs, vena cava invasion, capsular invasion, necrosis, mitotic rate/atypical mitoses, vascular invasion. In case of ≤2 criteria, tumours are considered benign; in case of three criteria, tumours are considered undetermined malignant potential, in case of ≥4 criteria tumours are considered malignant.

## Treatment for Malignant ACT

The overall strategy treatment is related to stage.

For localised ACT (ST I, II and some III) an upfront surgery is recommended. Adjuvant chemotherapy + mitotane is proposed in case of ST III, tumour rupture in children older than 4 years or non-normalisation of hormonal markers. In children less than 4 years is of debate and should be discussed. The first line recommended regimen is CED (cisplatin-doxorubicin-etoposide) or NN1/NN2 (vincristine-ifosfamide-doxorubicin and carboplatin-etoposide) [24].

For unresectable tumours (ST III) or metastases (ST IV), neoadjuvant chemotherapy including mitotane is recommended, followed by complete resection of the primary tumours and metastases (combined or delayed).

For relapsed tumours, there are no specific second-line therapies. Repeated surgeries and re-use of mitotane are possible.

The role of external radiotherapy is uncertain, since ACCs are usually radio-resistant neoplasms. Limited data exist on the efficacy of radiotherapy and it has been mostly used only as salvage therapy. Radiation therapy should be discussed in some refractory stage III ACC (R2, unresectable tumours), stage IV or relapsed tumours [15].

## Follow-up

Patients with unfavourable clinical or histological risk factors should undergo periodical clinical, imaging and hormonal studies. For those who harbour a germline TP53 or had a diagnosis of Li–Fraumeni syndrome, a long-term follow-up for other tumours is recommended [22, 23].

## References

[1] Ribeiro RC, Sandrini F, Figueiredo B (2001). An inherited p53 mutation that contributes in a tissue-specific manner to pediatric adrenal cortical carcinoma. Proc Natl Acad Sci USA.

[2] Rodriguez-Galindo C, Figueiredo BC, Zambetti GP (2005). Biology, clinical characteristics and management of adrenocortical tumors in children. Pediatr Blood Cancer.

[3] Custódio G, Komechen H, Figueiredo FRO (2012). Molecular epidemiology of adrenocortical tumors in southern Brazil. Mol Cell Endocrinol.

[4] Ferrari A, Brecht IB, Gatta G (2019). Defining and listing very rare cancers of paediatric age: consensus of the Joint Action on Rare Cancers in cooperation with the European Cooperative Study Group for Pediatric Rare Tumors. Eur J Cancer.

[5] Dall’Igna P, Virgone C, De Salvo GL (2014). Adrenocortical tumors in Italian children: analysis of clinical characteristics and P53 status. Data from the national registries. J Pediatr Surg.

[6] Ribeiro RC, Sandrini Neto RS, Schell MJ (1990). Adrenocortical carcinoma in children: a study of 40 cases. J Clin Oncol.

[7] Ribeiro RC, Pinto EM, Zambetti GP (2012). The International Pediatric Adrenocortical Tumor Registry initiative: contributions to clinical, biological, and treatment advances in pediatric adrenocortical tumors. Mol Cell Endocrinol.

[8] Picard C, Orbach D, Carton M (2019). Revisiting the role of the pathological grading in pediatric adrenal cortical tumors: results from a national cohort study with pathological review. Mod Pathol.

[9] Teinturier C, Pauchard MS, Brugières L (1999). Clinical and prognostic aspects of adrenocortical neoplasms in childhood. Med Pediatr Oncol.

[10] Michalkiewicz E, Sandrini R, Figueiredo B (2004). Clinical and outcome characteristics of children with adrenocortical tumors: a report from the International Pediatric Adrenocortical Tumor Registry. J Clin Oncol.

[11] Sandrini R, Ribeiro RC, DeLacerda L (1997). Childhood adrenocortical tumors. J Clin Endocrinol Metab.

[12] Dehner LP, Hill DA (2009). Adrenal cortical neoplasms in children: why so many carcinomas and yet so many survivors?. Pediatr Dev Pathol.

[13] Wieneke JA, Thompson LDR, Heffess CS (2003). Adrenal cortical neoplasms in the pediatric population: a clinicopathologic and immunophenotypic analysis of 83 patients. Am J Surg Pathol.

[14] Cecchetto G, Ganarin A, Bien E (2017). Outcome and prognostic factors in high-risk childhood adrenocortical carcinomas: a report from the European Cooperative Study Group on Pediatric Rare Tumors (EXPeRT). Pediatr Blood Cancer.

[15] Redlich A, Boxberger N, Strugala D (2012). Systemic treatment of adrenocortical carcinoma in children: data from the German GPOH-MET 97 trial. Klin Padiatr.

[16] McAteer JP, Huaco JA, Gow KW (2013). Predictors of survival in pediatric adrenocortical carcinoma: a Surveillance, Epidemiology, and End Results (SEER) program study. J Pediatr Surg.

[17] Williams AR, Hammer GD, Else T (2014). Transcutaneous biopsy of adrenocortical carcinoma is rarely helpful in diagnosis, potentially harmful, but does not affect patient outcome. Eur J Endocrinol.

[18] Fascetti-Leon F, Scotton G, Pio L (2017). Minimally invasive resection of adrenal masses in infants and children: results of a European multi-center survey. Surg Endosc.

[19] Facetti-Leon F, Scotton G, Pio L (2017). Erratum to: minimally invasive resection of adrenal masses in infants and children: results of a European multi-center survey. Surg Endosc.

[20] Heloury Y, Muthucumaru M, Panabokke G (2012). Minimally invasive adrenalectomy in children. J Pediatr Surg.

[21] Cundy TP, Marcus HJ, Clark J (2014). Robot-assisted minimally invasive surgery for pediatric solid tumors: a systematic review of feasibility and current status. Eur J Pediatr Surg.

[22] Frebourg T, Bajalica Lagercrantz S, The European Reference Network GENTURIS (2020). Guidelines for the Li–Fraumeni and heritable TP53-related cancer syndromes. Eur J Hum Genet.

[23] Kratz CP, Achatz MI, Brugières L (2017). Cancer screening recommendations for individuals with Li–Fraumeni syndrome. Clin Cancer Res.

[24] Abib SCV, Weldon C (2021). Management of adrenal tumors in pediatric patients. Surg Oncol Clin.

# Discussion: Gastrointestinal Stromal Tumours (GIST)

**Published:** 2022

**Authors:** Michele Ilari, Giovanni Torino

## Epidemiology, Biology and Clinical Aspects

Gastrointestinal stromal tumours (GISTs) are the most important group of mesenchymal smooth muscle neoplasms that can arise anywhere within the gastrointestinal tract, being their most frequent locations the stomach (60%–70%) and the small bowel (25%–35%). Their incidence during childhood is about 0.02 cases per 1 million per year [1, 2].

All tumours of mesenchymal origin that express the membrane protein kit (CD117) or which have a mutation in platelet-derived-growth factor a (PDGFRA) should be considered as GISTs [3–6].

Mutation of the ‘Kit gene’, except for the case of ‘Wild-type’ GISTs, leads to dysregulated signal of the Kit tyrosine kinase receptor, which is involved in critical cellular pathways that control cell proliferation and differentiation [7–9].

The most frequently reported symptoms associated with GIST in paediatric patients are gastrointestinal bleeding, associated with ‘more or less’ severe anaemia, abdominal pain and/or palpable mass. More rare presentations are massive bleeding, gastric outlet obstruction or constipation [10–12].

Diagnostic workup should include complete blood count, liver and kidney function tests and tumour markers. Besides conventional cross-sectional imaging, PET-CT scan and specific methods for studying gastrointestinal masses as Contrast-Enhanced Ultrasound or Digestive EchoEndoscopy with fine needle aspiration may be useful.

Percutaneous biopsy is not recommended because of tumour rupture and/or peritoneal spread. Samples of tumour tissue should be stained with antibodies against KIT and PDGFRA. GISTs are histologically subtyped into three categories: spindle cell, epithelioid or mixed type.

## Treatment

The therapeutic approach is stage related [13, 14]. Patients with advanced disease, showing evidence of liver and/or peritoneal metastases, neoadjuvant chemotherapy should be administered which consists of a competitive inhibition by monoclonal antibodies that prevents the transfer of phosphate groups from adenosine triphosphate to tyrosine residues on substrate protein (phosphorylation) interrupting proliferative intracellular signal pathways [15, 16].

Treatment for localised GISTs is the surgical resection of the entire mass with preservation of the normal tissue (pseudo-capsule) around the tumour [17–18]. The abdominal cavity should be thoroughly explored by laparotomy, with close examination of the peritoneal surfaces and liver to identify possible metastases. In the case of tumours involving adjacent organs, complete resection of the entire tumour and adjacent organs is acceptable, to obtain negative margins. Removal of uninvolved tissue is not recommended. Lymphadenectomy is indicated only if there is evidence of nodal involvement (rare in GISTs). Laparoscopy and robotic-assisted surgery can be used to resect small to intermediate sized lesions (<10 cm.), especially from the stomach [19–27].

Postoperative staging by the TNM system is performed to guide the treatment strategy based on tumour prognosis. Tumour size, location and mitotic count are prognostic factors considered to indicate adjuvant therapy. A high mitotic index (more than 5/50 High Power Fields) and/or tumours greater than 10 cm have been associated with increased recurrence rate.

Follow-up: Patients who had a resection of a primary GIST, should be scheduled for a clinical exam and abdomen/pelvis CT scan with IV contrast every 3–6 months during the first 3–5 years and, thereafter, every year [25–28].

## References

[1] Ilari M, Nino F, Zangari A (2014). Paediatric gastrointestinal stromal tumours arising from the stomach: a clinicopathologic review of 85 cases. J Pediatr Neonatal Care.

[2] Vliagoftis H, Worobec AS, Metcalfe DD (1997). The protooncogene c-kit and c-kit ligand in human disease. J Allergy Clin Immunol.

[3] Willobee BA, Quiroz HJ, Sussman MS (2018). Current treatment strategies in pediatric gastrointestinal stromal cell tumor. Transl Gastroenterol Hepatol.

[4] Weldon CB, Madenci AL, Boikos AS (2017). Surgical Management of wild-type gastrointestinal stromal tumors: a report from the national institutes of health pediatric and wildtype gist clinic. J Clin Oncol.

[5] Rubin BP, Fletcher JA, Fletcher CD (2000). Molecular insights into the histogenesis and pathogenesis of gastrointestinal stromal tumors. Int J Surg Pathol.

[6] Kindblom LG, Remotti HE, Aldenborg F (1998). Gastrointestinal pacemaker cell tumor (GIPACT): gastrointestinal stromal tumors show phenotypic characteristics of the interstitial cells of Cajal. Am J Pathol.

[7] Maeda H, Yamagata A, Nishikawa S (1992). Requirement of c-kit for development of intestinal pacemaker system. Development.

[8] Ward SM, Burns AJ, Torihashi S (1994). Mutation of the proto-oncogene c-kit blocks development of interstitial cells and electrical rhythmicity in murine intestine. J Physiol.

[9] Huizinga JD, Thuneberg L, Klüppel M (1995). W/kit gene required for interstitial cells of Cajal and for intestinal pacemaker activity. Nature.

[10] Sabah M, Leader M, Kay E (2005). Gastrointestinal stromal tumours: an update. Curr Diag Pathol.

[11] Prakash S, Sarran L, Socci N (2005). Gastrointestinal stromal tumors in children and young adults: a clinicopathologic, molecular, and genomic study of 15 cases and review of the literature. J Pediatr Hematol Oncol.

[12] Miettinen M, Lasota J, Sobin HL (2005). Gastrointestinal stromal tumors of the stomach in children and young adults. A clinicopathologic, immunohistochemical, and molecular genetic study of 44 cases with long-term follow-up and review of the literature. Am J Surg Pathol.

[13] Kuroiwa M, Hiwatari M, Hirato J (2005). Advanced-stage gastrointestinal stromal tumor treated with imatinib in a 12-year-old girl with a unique mutation of PDGFRA. J Ped Surg.

[14] Chompret A, Kannengiesser C, Barrois M (2004). PDGFRA germline mutation in a family with multiple cases of gastrointestinal stromal tumor. Gastroenterology.

[15] Kinoshita K, Hirota S, Isozaki K (2004). Absence of c-kit gene mutations in gastrointestinal stromal tumours from neurofibromatosis type 1 patients. J Pathol.

[16] Dematteo RP, Lewis JJ, Leung D (2000). Two hundred gastrointestinal stromal tumors: recurrence patterns and prognostic factors for survival. Ann Surg.

[17] Fletcher CD, Berman JJ, Corless C (2002). Diagnosis of gastrointestinal stromal tumors: a consensus approach. Hum Pathol.

[18] Miettinen M, El Rifai W, Sobin HL (2002). Evaluation of malignancy and prognosis of gastrointestinal stromal tumors: a review. Hum Pathol.

[19] Blay JY, Bonvalot S, Casali P (2005). Consensus meeting for the management of gastrointestinal stromal tumors. Report of the GIST consensus conference of 20–21 March 2004, under the auspices of European Society for Medical Oncology. Ann Oncol.

[20] Dematteo RP, Heinrich MC, El-Rifai WM (2002). Clinical management of gastrointestinal stromal tumor (GIST) before STI-571. Hum Pathol.

[21] Eisemberg BI, Judson I (2004). Surgery and imatinib in the management of GIST: emerging approaches to adjiuvant and neoadjuvant therapy. Ann Surg Oncol.

[22] Rubin BP, Heinrich MC, Corless CL (2007). Gastrointestinal stromal tumour. Lancet.

[23] Bedard EL, Mamazza J, Schlachta CM (2006). Laparoscopic resection of gastrointestinal stromal tumors: not all tumora are created equal. Surg Endosc.

[24] Novitsky YW, Kercher KW, Sing RF (2006). Long-term outcomes of laparoscopic resection of gastric gastrointestinal stromal tumors. Ann Surg.

[25] von Mehren M, Watson JC (2005). Gastrointestinal stromal tumors. Hematol Oncol Clin North Am.

[26] Joensuu H, Fletcher C, Dimitrijevic S (2002). Management of malignant gastrointestinal stromal tumours. Lancet Oncol.

[27] Blackstein ME, Blay JY, Corless C (2006). Gastrointestinal stromal tumors: consensus statement on diagnosis and treatment. Can J Gastroenterol.

[28] Demetri G, Benjamin R, Blanke CD (2004). NCCN Task Force report: optimal management of patients with gastrointestinal stromal tumor (GIST)—expansion and update of NCCN Clinical Practice Guidelines. J Natl Compr Canc Netw.

# Discussion: Melanoma

**Published:** 2022

**Authors:** Michele Ilari, Elisa Chiarella, Giovanni Torino

## Evaluation

### Epidemiology

Malignant melanoma (MM) in the paediatric age is rare. A large series reports an incidence by age of 0.7/million/year at 0–9 years and 13.2/million/year at 10–15 years [1]. The increased risk of melanoma development in large congenital nevi is widely accepted. Melanoma arose from intermediate congenital nevi has been reported in prepubertal age, although there is no evidence that prophylactic excision is mandatory [2]. Risk of melanoma in small congenital nevi is still matter of controversy, although in some reports it seems not to be entirely insignificant [3]. The purpose of this study is to discuss surgical indications of the nevi and diagnosis and treatment of the melanoma.

### Clinical presentation

There are three subtypes of melanomas that are prevalent in the paediatric population: Spitzoid melanoma, melanoma arising from congenital melanocytic nevi and conventional melanoma (or adult-type melanoma) which can spread to the surface layer or extend deeply as the nodular type [4].

Indications for excision of the melanoma include the clinical appearance/modified ABCD criteria (asymmetry of the lesion, edges irregularity, irregular and/or dark colouring, size more than 5 mm), not circular expansion, other ‘warning’ signs of MM (rapid onset and growth, bleeding, itching, pain), site of lesion of difficult monitoring and/or in case of exposure of the lesion to frequent trauma.

#### Spitzoid Melanoma

These lesions usually present as pink or reddish nodules on the extremities or head and neck. The Spitzoid melanomas have a benign behaviour although they are constituted by melanocytic cells [5–7]. They more commonly display the modified ABCD criteria (asymmetry of the lesion, edges irregularity, irregular and/or dark colouring, size larger than 5 mm, not circular expansion) than non-Spitzoid nevi in which the modified ABCD criteria are met in only 40% of cases.

#### Melanoma Arising in Congenital Melanocytic Nevi

The congenital melanocytic nevi affect approximately 1% of newborn and they are classified according to their diameter in: small (<1.5 cm), medium (1.5–19.9 cm) and large or giant (≥20 cm). These nevi are slightly more common in females, but more likely to be associated with complications in males.

#### Conventional (adult-type) Melanoma

About 40–50% of paediatric melanomas are considered conventional. Among such cases, risk factors and baseline characteristics vary according to age. The conventional melanoma is more recurring in Caucasian race because the white skin is more sensitive to sunrays. The conventional melanoma that arise in the paediatric population are often nodular and amelanotic, moreover, atypical vascular or crystalline structures can be seen at the dermoscopy.

### Work up

The use of ‘total body photography’ in children and adolescents to identify new or ‘changing’ nevi is useful. The surgical indications based on change or onset of the nevi has poor specificity because of the volatility and variability of nevi during the growth of the child. Paediatric melanomas often fail to exhibit conventional ABCDE criteria, but the ‘change’ remains an important diagnostic ‘clue’, despite its poor specificity. Sequential digital ‘dermatoscopic imaging’, can be used to improve diagnostic accuracy in children’s melanoma.

PET-CT scan is indicated in confirmed cases for staging and evaluation of distant metastasis.

## Pre-Operative Management

Indications for surgical excision of ‘acquired nevi’ are mainly related to lesions resembling MM, such as atypical nevi, Spitz nevi and lesions presenting with clinical signs and symptoms as the modified ABCD criteria, ulceration, bleeding and/or itching [8]. Patients with atypical nevi have demonstrated to be at increased risk for developing melanoma. This risk is higher in familial atypical nevi subgroups, but it has been found even in patients with non-familial atypical nevi. The congenital nevi are at higher risk of developing malignancy, but their excision remains debatable.

## Surgical Guidelines

The surgical approach of the ‘primary lesion’ consists of a ‘wide local excision’, with 2-mm margins of normal skin and that should extend to the muscular fascia.

Recommended surgical margins, after diagnosis of MM, are based on ‘Breslow thickness’ [9, 10] (described in adults):

- Lesions ≤1.0 mm in depth require a margin of 1.0 cm.
- Lesions > 2 mm in depth require a margin of 2.0 cm.
- Intermediate thickness lesions (1.0–2.0 mm) are recommended to have a margin of 1.0–2.0 cm.

Similar to wide local excision margins, the indications for sentinel lymph node biopsy (SLNB) are actually based on ‘adult guidelines’ [11]:

- SLNB may be avoided for lesion depth < 0.8 mm without other concerning features such as ulceration.
- Lesions with thickness ≥ 1.0 mm should undergo SLNB.
- For lesions between 0.8 and 1.0 mm and those <0.8 mm with ulceration, SLNB should be offered with a discussion of risks and benefits with the patient and family.

SLNB must be performed using preoperative lymphoscintigraphy, intraoperative blue dye injection around the site of excision and handheld gamma probe for radiolocalisation.

Completion lymph node excision is recommended for patients with ‘positive SLNB’ (although it is controversial for patients with this situation only) and/or ‘high-risk features’ such as extracapsular extension, primary tumour microsatellitosis (1 or more discontinuous nests of melanoma cells with a diameter ≥ 0.3 mm and separated by >0.05 mm of normal dermis or subcutaneous tissue), >3 involved lymph nodes and patient affected by immunosuppression.

## Postoperative Management

Pathologic reports should be reviewed for standard pathologic variables including Breslow thickness, lymph node involvement and histologic diagnosis for lesions other than melanoma.

Surgical procedures can result not only in short-term discomfort, limitation of physical activity and risk of infection, but also in long-term complications such ‘scars’ that may restrict the mobility and function of the joints.

In particular, in case of ‘large or giant’ congenital melanocytic nevi where the excision is not feasible, techniques such as curettage, dermabrasion and ablative (e.g. carbon dioxide, erbium:YAG) or pigment-specific laser therapy may have an important benefit [12].

Systemic therapy is indicated for patients with regionally advanced disease or distant metastatic disease.

## References

[1] Zangari A, Bernardini ML, Tallarico R (2007). Indications for excision of nevi and melanoma diagnosed in a pediatric surgical unit. J Pediatr Surg.

[2] Ferrari A, Bisogno G, Cecchetto G (2014). Cutaneous melanoma in children and adolescents: the Italian rare tumors in pediatric age project experience. J Pediatr.

[3] Zangari A, Ilari M, Nino F (2013). Report of a malignant melanoma arising in a small congenital nevus in a 3-year-old child. Indian Assoc Pediatr Surg.

[4] Makkar HS, Frieden IJ (2002). Congenital melanocytic nevi: an update for the paediatrician. Curr Opin Pediatr.

[5] Pappo AS (2003). Melanoma in children and adolescents. Eur J Cancer.

[6] Swetter SM (2004). Malignant Melanoma.

[7] Sahin S, Levin L, Kopf AW (1999). Risk of melanoma in medium-sized congenital melanocytic nevi: a follow-up study. J Am Acad Dermatol.

[8] Yesudian PD, Parslew RAG (2003). A guide to the management of pigmented skin naevi in children. Curr Paediatr.

[9] Wong SL, Faries MB, Kennedy EB (2018). Sentinel lymph node biopsy and manage- ment of regional lymph nodes in melanoma: American Society of Clinical Oncology and Society of Surgical Oncology clinical practice guideline update. J Clin Oncol.

[10] Wong SL, Kennedy EB, Lyman GH (2018). Sentinel lymph node biopsy and management of regional lymph nodes in melanoma: American Society of Clinical Oncology and Society of Surgical Oncology clinical practice guideline update summary. J Oncol Pract.

[11] Neville HL, Andrassy RJ, Lally KP (2000). Lymphatic mapping with sentinel node biopsy in pediatric patients. J Pediatr Surg.

[12] Aldrink J, Polites S, Lautz TB (2019). What’s new in pediatric melanoma: an Update from the APSA Cancer Committee. J Pediatr Surg.

# Discussion: Salivary Gland Tumours

**Published:** 2022

**Authors:** Daniel Liberto, Pablo Lobos

## Evaluation

### Epidemiology

Malignant tumours of the salivary glands (salivary gland tumour) are rare, accounting for less than 10% of all paediatric head and neck malignancies, with an annual incidence less than 1 in 1 million. Children younger than 10 years are less frequently affected, and there is a slight predominance in females (1.4:1) [1, 2].

About 40%–60% of all paediatric SGT are malignant, much more than in adults. The parotid gland is the most frequently affected site (72%–88%), followed by minor salivary glands (7%) and the submandibular ones (5%).

SGT can arise from the epithelium (carcinomas), or they can have a mesenchymal (sarcoma) or lymphoid origin (lymphoma). The most frequent SGTs are low-grade mucoepidermoid carcinomas (MECAs), followed by acinar cells cystic carcinomas (both account for 80% of all carcinomas). Other malignancies include rhabdomyosarcoma, lymphoma and rarely, sialoblastoma. Histologic types are classified according to the WHO classification of malignant tumours of the major salivary glands (2005).

### Clinical presentation

The usual presentation of a SGT is an otherwise asymptomatic, slow-growing mass. A higher suspicion index for malignancy is raised when rapid growth, facial nerve palsy, hard fixed lesions and associated enlarged cervical lymph nodes are observed. Approximately 10% of paediatric patients present as the first clinical sign with satellite palpable cervical lymph nodes. Painful masses suggest adjacent nerves infiltration, indicating worse prognosis [3–5].

SGT affecting the minor glands usually presents as a non-painful non-ulcerated mass, palpable from the oral cavity. They are frequently localised at the hard or soft portion of the palate. Depending on their specific location, they can present with nasal obstruction, epistaxis, dysphonia, breathing complaints or dysphagia.

Complete physical exam of the head and neck region is indicated, with special focus on the size, location and mobility of the tumour. Bilaterality, multiplicity and/or synchronic tumours are very rare.

### Workup

There are no relevant tumour markers or specific analytic workup for this kind of tumours. Ultrasound and Doppler are the first imaging study usually indicated for SGT. This method will give information on tumour features as it is localised or diffuse, solid or cystic, poorly or highly-vascularised, how deep it is, infiltration of adjacent tissues and facial nerve. Ultrasound is capable of differentiating extraglandular lesions from intraglandular ones in 98% of cases.

Depending on the features displayed by Ultrasound, MRI and/or CT scan should be indicated for further investigation. Eventual resectability may be evaluated by one of these methods. We recommend the indication of MRI, as parotid lesions are well defined in T1 sequences. T1 images offer valuable information about tumour margins, deepness and infiltrative pattern; perineural, vascular and osseous invasion, as meningeal infiltration, can be adequately evaluated by this method. T2 sequences are useful to differentiate benign from malignant lesions in 73% of cases, being hyperintense masses more probably benign.

Indications of PET-CT scan with 18-fluro-deoxiglucose have not been well defined. Both malignant and benign tumours show a high concentration of the tracer, being less specific than MRI. This method is actually recommended for the follow-up of patients with distant metastases [6].

## Indications and Principles of Biopsy

Although cytology studies performed on samples obtained by fine-needle aspiration have been widely accepted for the diagnosis of SGT in adults, they are still not fully established for paediatric cases [7–9]. It is very important to have a preoperative diagnosis of SGT to plan an adequate surgical approach. Recent studies have reported a high diagnostic yield of fine-needle aspiration for SGT in paediatrics, with a sensibility of 92% and specificity of 86%, similar to previously reported for adults.

In cases presenting with satellite lymph nodes at diagnosis, an excisional biopsy may be preferred as malignancy is suspected. Incisional biopsies are not indicated for the risk of facial nerve damage and tumour spillage.

## Perioperative Management

### Role and timing of multimodal therapy

The treatment of paediatric SGT is mainly based on the surgical resection of the mass, like in adult cases. There are no prospective or retrospective studies available that compare the surgery-only treatment with multimodal therapies for paediatric malignant SGT.

Neoadjuvant chemotherapy and/or radiation may be considered for high-risk patients with large tumours, advanced stages, positive margins after resection or high-grade malignancies.

### Preoperative considerations

All patients who will be candidates for surgery should be screened, either pre and postoperatively, with the House–Brackmann scale for the function of the facial nerve.

## Surgery

### Surgery goals

The ultimate goal of surgery for paediatric SGT is the complete resection of the lesion, with intraoperative visualisation and control of the facial nerve. Malignant tumours affecting the major salivary glands with associated lymph node metastases require a total parotidectomy with cervical dissection, with further adjuvant chemotherapy and/or radiation, according to histologic type. Resectability of advanced tumours must be determined by preoperative imaging. When infiltration of adjacent structures is demonstrated, radical surgery with facial nerve sacrifice may be indicated.

Regarding cervical dissection in N0 patients, selective dissection of cervical regions I-III is suggested for: high-grade malignancies, T3-T4, extraglandular compromise and lymphatic invasion within the tumour [10]. For N+ cases, a modified radical cervical dissection versus selective cervical dissection of regions I-III or I-IV is recommended.

## Postoperative Considerations

The indication of adjuvant radiation therapy for paediatric parotid gland tumours is controversial. It is recommended in patients with high-grade tumours, perineural or vascular invasion, residual disease or cervical lymph node metastases [11]. A multidisciplinary discussion must be made, as long-term sequelae as trismus, cranio-facial deformities, osteoradionecrosis and second malignancies can occur. Recent reports have shown indication of radiation therapy in 27% of study cohorts, in contrast to 51% in adults. Approximately one half of the patients will present a Frey syndrome in the long term.

## Prognosis and Follow-up

Reported 5-year OS and EFS is 94%–96% and 83%, respectively [12, 13]. Factors contributing to this improved survival for children against adults are: more localised tumours, with less extensive lesions and less frequent cervical metastases (76% versus 50%), more frequent well or moderately differentiated tumours (88% versus 49%). MECA and acinar cells carcinoma account for 83.6% of paediatric tumours, against 35.4% in adults [14]. Risk factors predicting dismal outcome were tumour grade, tumour margin status and nerve invasion. A worst survival has been reported for children younger than 10 years (5-year OS 50%).

Local recurrence has been reported up to 20% of cases, within a median of 1.1 years after diagnosis. SGT affecting the minor salivary glands, particularly high-grade lesions, has been reported to have a worse prognosis, with survival around 50%.

Long term follow-up has been suggested, as late recurrences have been reported even 45 years after the initial treatment [15].

SGT of non-epithelial origin deserves a special comment. Although lymphomas and metastatic tumours have been reported to occur at the salivary glands, most of these cases have been published as case reports or small series. In fact, only the mesenchymal tumours and, in particular, the rhabdomyosarcoma is most frequently reported. About 40%–50% of them present at a parameningeal site. Almost all are treated with radiation. Intergroup Rhabdomyosarcoma Study (IRS) (Rhabdomyosarcoma Study Group) protocols are highly effective to obtain complete remission. Reported 5-year OS of 84% has been reported for IRS stages 1 and 2.

## References

[1] Yoshida YE, García J, Eisele D (2014). Salivary gland malignancies in children. Int J Pediatr Otorhinolaryngol.

[2] Ord RA, Carlson ER (2016). Pediatric salivary gland malignancies. Oral Maxillofacial Surg Clin N Am.

[3] Lennon P, Silvera V, Perez-Atayde A (2015). Disorders and tumors of the salivary glands in children. Otolaryngol Clin N Am.

[4] Aro K, Leivo I, Grénman R (2012). Paediatric salivary gland cancer in Finland. Paediatric salivary gland cancer in Finland. Int J Pediatr Otorhinolaryngolo.

[5] Ethunandan M, Ethunandan A, Macpherson D (2003). Parotid neoplasms in children: experience of diagnosis and management in a district general hospital. Int J Oral Maxillofac Surg.

[6] Kitajima K, Suenaga Y, Sugimura K (2015). Present and future role of FDG-PET/CT imaging in the management of head and neck carcinoma. Jpn J Radiol.

[7] Ronchi A, Montella M, Zito Marino (2019). Diagnostic accuracy of FNA cytology for diagnosis of salivary gland tumors in pediatric patients. Cancer Cytopathol.

[8] Agaimy A, Iro H, Zenk J (2017). Pediatric salivary gland tumors and tumor-like lesions.

[9] Oesterreich R, Udaquiola J, Lobos P (2016). Diagnóstico y tratamiento de los tumores de la región parotídea en Pediatría: cohorte. Cir Pediatr.

[10] Wenig B (2015). Chapter 20 (Elsevier) pp 861–1049. Neoplasms of Salivary Glands. Atlas of head and Neck Pathology.

[11] House JW, Brackmann DE (1985). Facial nerve grading system. Otolaryngol Head Neck Surg.

[12] Stevens E, Andreasen S, Bjørndal K (2015). Tumors in the parotid are not relatively more often malignant in children than in adults. Int J Pediatr Otorhinolaryngol.

[13] Zamani M, Grønhøj C (2019). Survival and characteristics of pediatric salivary gland cancer: a systematic review and meta-analysis. Pediatr Blood Cancer.

[14] Cockerill C, Gross B, Contag S (2016). Pediatric malignant salivary gland tumors: 60 year follow up. Int J Pediatr Otorhinolaryngol.

[15] Barnes L, Evesnon J, Reichart P (2005). World Health Organization Classification of Tumours: Pathology and Genetics of Head and Neck Tumours.

# Discussion: Neuroendocrine Tumours of the GI Tract

**Published:** 2022

**Authors:** Jennifer Aldrink, Calogero Virgone

## Evaluation

### Epidemiology

Although rare, NETs are the most common GI epithelial tumours in paediatric age [1]. Appendiceal NETs are usually an incidental finding at histology after appendectomy [1–3]. Several paediatric series have been reported, but the precise incidence in relation to the total number of appendectomies is still not available. The general incidence has been estimated in a range between 1:100,000 and 1.14:1 million children per year [1–4].

Extra-appendicular NETs are even rarer: in the paediatric age, the majority of them arise from the pancreas and bronchi. The current incidence in adults ranges from 3.24/100 000 in North Europe to 5.25/100 000 in the United States. In children, the incidence is estimated to be around 0.5 cases per million/year [5, 6].

Extra-appendicular NETs are sporadic in most cases, but they may also be part of a hereditary syndrome: pancreatic NETs are associated with TS, MEN1, VHN syndrome and NF1 [7–9].

### Clinical presentation

Symptoms may vary by location. Appendiceal NETs (previously known as carcinoid tumours of the appendix, although this term is no longer in the WHO classification) are typically diagnosed after appendectomy: their role in the onset of appendicitis is not clear. Extra-appendicular NET may present with symptoms related to mass-effect or to hormonal secretion (gastrine, glucagon, vasoactive intestinal peptide (VIP), Insulin). In metastatic cases, extremely rare in children, a carcinoid syndrome (flushing, diarrhoea, and, less frequently, heart failure) may be evident. Pancreatic NETs may present with symptoms related to hormonal secretion, or, when it is absent, fatigue, fever abdominal pain or an abdominal mass can be palpable.

### Workup

Appendiceal NET (post appendectomy) Lab: Complete blood count, complete metabolic profile, coagulation profile. Blood lactate dehydrogenase (LDH), neuron-specific enolase (NSE), Cromogranin A and urinary 24-hours 5-idrossiindolacetic acid.

Extra-appendicular neuroendocrine tumours (preoperative setting) Lab: Complete blood count, complete metabolic profile, and coagulation profile. Blood LDH, NSE, Cromogranin A and urinary 24-hours 5-hydroxyindolacetic (5-HIIA) acid. Dosage of gastrin, glucagone, peptide C, VIP (pancreatic tumours).

Appendiceal NET (post appendectomy) Imaging: abdominal US, and/or (depending on assessed size on histology) CT/MRI abdomen, and PET-CT (Figure 1 shows, as an example, the current recommendations from the Italian TREP group).

Extra-appendicular NET (preoperative setting) Imaging: abdominal US, and CT/MRI abdomen, and PET-CT (68Ga-DOTATATE, 68Ga-DOTATOC, 68Ga-DOTANOC), chest CT [10, 11].

It is worth to underline that some recent papers (one analysis on a large national registry and one meta-analysis) suggest that postoperative surveillance should be limited in patients found to have an appendiceal NET, avoiding the use of imaging and markers’ dosage when patients do not claim any symptom or clinical sign [12–15]. According their consideration, follow-up investigations could be omitted in most patients (with tumours < 1.6 or 2 cm and completely resected). However, these findings should be hopefully confirmed by large prospective series.

### Indications and principles of biopsy

Biopsy in appendiceal NETs is not indicated, even in those exceptional cases where the tumour is diagnosed preoperatively.

For what concerns extra-appendiceal NETs, a biopsy may be useful when instrumental and laboratory work-up do not allow to precisely define the diagnosis, or in inoperable tumours. Adequate sampling may be obtained through a laparotomy/laparoscopy, image-guided percutaneous tru-cut or with the help of endoscopic ultrasound guidance (endoscopic ultrasound-guided fine needle aspiration biopsy), in tumours arising from the head of the pancreas.

## Perioperative Management

### Role and timing of multimodality therapy

There are no ongoing protocols for children with GI NET. Adjuvant or neo-adjuvant strategies are derived from adult guidelines and are usually adopted on a case-by-case strategy in children [5, 10, 16–18].

### Preoperative considerations

Preoperative multidisciplinary planning should include the assessment of comorbidities, magnitude of the operation, capacity of the anaesthesia team, intraoperative monitoring, reliable upper extremity vascular access, urinary catheter, availability of blood, appropriate allocation of postoperative level of care and monitoring and postoperative pain control. If a neoadjuvant chemotherapy protocol is used, surgery should follow blood count recovery.

## Surgery

### Appendicular NET (after appendectomy)

Right hemicolectomy (RHC) or caecum resection has been traditionally recommended for tumours larger than 2 cm or with involved margins [19]; however, it is controversial whether this approach may offer more advantages in terms of OS and EFS rates. Recent studies exploring the SEER database both in adults [20] and in children [14] showed how a formal RHC does not offer any advantage in term of survival. However, the most recent guidelines for the treatment of NETs of the appendix in adults [16–18] recommend RHC not only for tumours larger than 2 cm but also for incompletely excised tumours of any size, for tumours located at the base of the appendix and for cases presenting vascular invasion or extension into periappendiceal fat. Some authors actually question the need of RHC in case of either vascular invasion or extension into the periappendiceal fat [19, 21–27] because in their series no correlation with the presence of distant metastases was observed.

Concerning tumour size, Moertel *et al* [19] found in their own series that 12 of 23 adults with tumours larger than 2 cm did not undergo RHC and just 1 had a relapse 29 years after diagnosis, that was successfully treated with RHC. Other authors recently suggested that RHC did not give any relevant benefit in terms of OS and cancer-specific survival in patients affected by NETs larger than 2 cm. According to them, appendectomy alone seems therefore sufficient for the local control of the disease [21].

The GPHO-MET study on appendiceal NETs in children and adolescents asserted that all of the patients with tumours larger than or equal to 1.5 cm, even if completely excised, should undergo secondary RHC with mesenteric lymph nodes sampling [4]. At the basis of their approach, there was a high incidence of micrometastatic nodal spread in 16% of patients (9/60); however, no local or metastatic relapse was observed.

Moreover, the real clinical significance of nodal micrometastases is still unclear; whether they represent a true metastatic localisation or not, should be determined on the basis of a larger and long-lasting series.

The other two most numerous paediatric series published supported a less aggressive strategy. Virgone *et al* [1] did not suggested RHC even for tumour larger than 2 cm, leaving a door open to re-operation only for incompletely excised tumours; while deLambert *et al* [12] recommended not to perform any kind of second surgery in any case, and also discouraged prolonged and detailed follow-up as described in the Italian recommendations.

In addition, a recent meta-analysis has also observed that no relapse or tumour-related death occurred in all published cases of paediatric appendiceal NET [28]. As far as we know, the only case of relapsed appendiceal NET, published in 2021, has size as only risk factor [29].

In the view of these suggestions and considering the benign clinical course of appendiceal NETs in children (which are well differentiated in almost all cases), we do not recommend and aggressive surgical approach according to the indication given in adults. This approach is currently shared by most authors, and described in the available national recommendations [1, 12, 13, 30].

In summary, we recommend that appendectomy alone should be considered curative in all completely resected NETs smaller than 2 cm. A more aggressive surgical approach, such as subtotal or total resection of the caecum, or even RHC, can be considered, but not mandatory, in those cases with incompletely excised tumours or when size exceed 2 cm: these cases, although rare, should be managed among local and national MDT.

For what concerns high-grade tumours (NET G3 – exceptional also in adults) or undifferentiated tumours (Neuroendocrine Carcinoma), or mixed epithelial-neuroendocrine neoplasm, our recommendation is to follow adult guidelines, which usually adopt a strategy similar to that for colon adenocarcinoma. These recommendations include also Goblet cell carcinoids, which are nowadays included in the epithelial neoplasms classification and shouldn’t be considered anymore together with other NETs.

Extra-appendicular GI NET

A complete surgical resection, when available, is the main prognostic factor for a good outcome [3, 5, 16–18, 31, 32].

Pancreatic NETs in children are extremely rare. While limited data is available and primarily retrospective in nature, the incidence is approximately 0.5 cases per million/year, and survival estimates demonstrate an OS and EFS around 80% and 90%, respectively [5]. Other sites (liver, unknown primary) usually have a worse outcome. NETs are sporadic in most cases but may also be part of a hereditary syndrome: pancreatic NETs are associated with TS, MEN1, VHN syndrome and NF1.

Pancreatic NETs are a distinct group of gastrointestinal neuroendocrine neoplasms. The most common types of pancreatic NETs in children are insulinomas and gastrinomas. Hypoglycaemia is the dominant symptom of insulinoma. In children, hypoglycaemia usually manifests as behavioural disorders, convulsions or coma. Gastrinomas secrete gastrin and typical symptoms include gastrointestinal ulcers (Zollinger–Ellison syndrome), chronic abdominal pain or symptoms of reflux disease; less common are diarrhoea and weight loss, or anaemia secondary to abnormal iron absorption. Since these symptoms are non-specific, diagnosis most frequently is made with considerable delay, even up to 4–6 years after the occurrence of the first symptoms. Considering the slow growing nature of these tumours, a long follow-up should be recommended (≥ 10 years) [33].

Diagnostic and therapeutic guidelines in adult patients are well standardised and discussed in a previous chapter on pancreatic tumours. The mainstay of treatment in localised tumours is the complete surgical resection, but in case of locoregional invasiveness or distant metastasis, the treatment options are challenging, because the response to conventional chemotherapy is poor [5, 8]. Surgical debulking can be taken into consideration, as long-term survivals have been described in adults with metastatic or inoperable disease when associated with adjuvant therapy (somatostatin analogues). This choice should be weighed according to the general conditions of the patients and after MDT discussion including adult oncologists and surgeons who are experts in the management of NETs.

Liver metastases may also benefit from a complete surgery at time of the resection of the primary tumour. In most cases, however, surgery is not feasible and other techniques should be considered, such as radiofrequency ablation and chemoembolisation, whose utilisation in children is becoming more and more attractive for various histotypes [8, 34].

## Postoperative Considerations

After major operations (RHC, Whipple or Traverso–Longmire operation), special attention should be addressed to the monitoring and treatment of possible complications (e.g. persistent pancreatic fistula, diabetes or diarrhoea).

## Prognosis, Prognostics and Follow-up

OS and EFS of patients with appendicular NET is 100%. No progression or death for disease has been reported regarding this subset of patients, and only one relapse successfully treated with surgery only has been reported so far. Given the fact that abnormal level of Chromogranin A (CgA) and 5-HIIA is detectable only when a big tumour load or a carcinoid syndrome are, respectively, present, it seems reasonable to limit their monitoring only for patients with tumours > 2 cm or incompletely resected and without a second surgery. For similar reasons, the use of cross sectional imaging (CT or MRI) and Somatostatin Receptor Imaging-Positron Emission Tomography (SRI-PET) studies may be reserved to a selected subgroup of appendicular NET (larger tumours, incompletely resected tumours, G2 or higher). In those patients with low risk appendicular NET (completely resected, < 2 cm, G1), the burden of follow-up should be reduced: in these cases, a strict follow-up can be omitted and post-appendectomy investigations may be limited to a US scan.

Considering the slow growing nature of these tumours, however, a long follow-up may be recommended (≥ 10 years) in those patients with risk factors who did not received any treatment after appendectomy [1, 4, 12].

Pancreatic NETs in children show an OS and EFS around 80% and 90%, respectively, but data are scarce and are derived mostly from retrospective series. Other sites (liver, unknown primary) usually have a more dismal outcome. Considering the slow growing nature of these tumours, a long follow-up should be recommended (≥ 10 years) [36].

## Figures and Tables

**Figure 1.** TREP guidelines for diagnostic workup and treatment after appendectomy. CT, Computed tomography; FU, Follow-up; 5-HIIA, 5-hydroxyindoleacetic acid; MRI, Magnetic resonance imaging; NET, Neuroendocrine tumour; US, Ultrasound (Virgone et al [1]).

## References

[1] Virgone C, Cecchetto G, Alaggio R (2014). Appendiceal neuroendocrine tumours in childhood: Italian TREP project. J Pediatr Gastroenterol Nutr.

[2] Moertel CL, Weiland LH, Telander RL (1990). Carcinoid tumor of the appendix in the first two decades of life. J Pediatr Surg.

[3] Navalkele P, O’Dorisio MS, O’Dorisio TM (2011). Incidence, survival, and prevalence of neuroendocrine tumors versus neuroblastoma in children and young adults: nine standard SEER registries, 1975–2006. Pediatr Blood Cancer.

[4] Boxberger N, Redlich A, Boger C (2013). Neuroendocrine tumors of the appendix in children and adolescents. Pediatr Blood Cancer.

[5] Virgone C, Ferrari A, Chiaravalli S (2021). Extra-appendicular neuroendocrine tumors: a report from the TREP project (2000–2020). Pediatr Blood Cancer.

[6] Cavalcoli F, Garrahy A, Castellaneta M (2018). Epidemiology of neuroendocrine tumours: by site of tumour and by geographical area. Neuroendocrine Tumors in Real Life.

[7] Sacco Casamassima MG, Gause CD, Goldstein SD (2016). Pancreatic surgery for tumors in children and adolescents. Pediatr Surg Int.

[8] Stawarski A, Maleika P (2020). Neuroendocrine tumors of the gastrointestinal tract and pancreas: is it also a challenge for pediatricians?. Adv Clin Exp Med.

[9] Lodish MB, Stratakis CA (2010). Endocrine tumours in neurofibromatosis type 1, tuberous sclerosis, and related syndromes. Best Pract Res Clin Endocrinol Metab.

[10] Boudreaux JP, Klimstra DS, Hassan MM (2010). The NANETS consensus guideline for the diagnosis and management of neuroendocrine tumors: well-differentiated neuroendocrine tumors of the jejunum, ileum, appendix, and cecum. Pancreas.

[11] Hicks RJ, Kwekkeboom DJ, Krenning E (2017). ENETS consensus guidelines for the standards of care in neuroendocrine neoplasia: peptide receptor radionuclide therapy with radiolabeled somatostatin analogues. Neuroendocrinology.

[12] de Lambert G, Lardy H, Martelli H (2016). Surgical management of neuroendocrine tumors of the appendix in children and adolescents: a retrospective french multicenter study of 114 cases. Pediatr Blood Cancer.

[13] Njere I, Smith LL, Thurairasa D (2018). Systematic review and meta-analysis of appendiceal carcinoid tumors in children. Pediatr Blood Cancer.

[14] Parikh PP, Perez EA, Neville HL (2018). Nationwide overview of survival and management of appendiceal tumors in children. J Pediatr Surg.

[15] Lobeck IN, Jeste N, Geller J (2017). Surgical management and surveillance of pediatric appendiceal carcinoid tumor. J Pediatr Surg.

[16] Pavel M, O’Toole D, Costa F (2016). ENETS consensus guidelines update for the management of distant metastatic disease of intestinal, pancreatic, bronchial neuroendocrine neoplasms (NEN) and NEN of unknown primary site. Neuroendocrinology.

[17] Pape UF, Niederle B, Costa F (2016). ENETS consensus guidelines for neuroendocrine neoplasms of the appendix (excluding goblet cell carcinomas). Neuroendocrinology.

[18] Kunz PL, Reidy-Lagunes D, Anthony LB (2013). Consensus guidelines for the management and treatment of neuroendocrine tumors. Pancreas.

[19] Moertel CG, Weiland LH, Nagorney DM (1987). Carcinoid tumor of the appendix: treatment and prognosis. N Engl J Med.

[20] Mehrvarz Sarshekeh A, Advani S, Halperin DM (2017). Regional lymph node involvement and outcomes in appendiceal neuroendocrine tumors: a SEER database analysis. Oncotarget.

[21] Rossi G, Valli R, Bertolini F (2003). Does mesoappendix infiltration predict a worse prognosis in incidental neuroendocrine tumors of the appendix? A clinicopathologic and immunohistochemical study of 15 cases. Am J Clin Pathol.

[22] Kulke MH, Mayer RJ (1999). Carcinoid tumors. N Engl J Med.

[23] Landry CS, Woodall C, Scoggins CR (2008). Analysis of 900 appendiceal carcinoid tumors for a proposed predictive staging system. Arch Surg.

[24] Mullen JT, Savarese DM (2011). Carcinoid tumors of the appendix: a population-based study. J Surg Oncol.

[25] Guzman C, Boddhula S, Panneerselvam N (2020). Appendiceal carcinoid tumors: is there a survival advantage to colectomy over appendectomy?. J Gastrointest Surg.

[26] Bamboat ZM, Berger DL (2006). Is right hemicolectomy for 2.0-cm appendiceal carcinoids justified?. Arch Surg.

[27] Alexandraki KI, Kaltsas G, Grozinsky-Glasberg S (2020). The effect of prophylactic surgery in survival and HRQoL in appendiceal NEN. Endocrine.

[28] Panek M, Szymczak M, Stepaniuk M (2021). Radical surgical treatment of neuroendocrine tumors of the appendix in children - a Polish multicenter study. Arch Med Sci.

[29] Mishra PR, Stringer MD (2020). Appendiceal carcinoid tumors: a plea for critical reporting. Pediatr Surg Int.

[30] Howell DL, O’Dorisio MS (2012). Management of neuroendocrine tumors in children, adolescents, and young adults. J Pediatr Hematol Oncol.

[31] Mylonas KS, Doulamis IP, Tsilimigras DI (2018). Solid pseudopapillary and malignant pancreatic tumors in childhood: a systematic review and evidence quality assessment. Pediatr Blood Cancer.

[32] Arnold R, Chen YJ, Costa F (2009). ENETS Consensus Guidelines for the Standards of Care in Neuroendocrine Tumors: follow-up and documentation. Neuroendocrinology.

[33] Gómez FM, Patel PA, Stuart S (2014). Systematic review of ablation techniques for the treatment of malignant or aggressive benign lesions in children. Pediatr Radiol.

[34] Zambaiti E, Siles Hinojosa A, Montano V (2019). Interventional radiology-guided procedures in the treatment of pediatric solid tumors: a systematic review and meta-analysis from the group of young pediatric surgeons of Europe. Eur J Pediatr Surg.

# Discussion: Surgery for lymphoma

**Published:** 2022

**Authors:** Sharon Cox, Abdelhafeez Abdelhafeez, Simone Abib

## Introduction

Lymphomas are a heterogeneous group of malignancies of the reticuloendothelial system and are pathologically classified into two main groups – Hodgkin’s lymphoma (HL) and non-Hodgkin’s lymphoma (NHL).

Lymphoma accounts for about 10%–12% of childhood malignancies, making it the third most common paediatric cancer, with NHL accounting for just less than half of the cases, and Burkitt’s Lymphoma predominating in this group [1].

Historically, the role of the surgeon was to perform staging laparotomy for biopsy of nodes and the liver as well as a splenectomy. Cross sectional imaging has replaced this staging need, but an adequate tissue diagnosis is imperative to achieve an accurate diagnosis and classification using various histological techniques [2].

The presentations, symptoms and signs of patients presenting with lymphoma are extremely variable. Patients can present with an obvious nodal or non-nodal mass and undergo a routine blood workup and biopsy or can present with various surgical diagnoses such as and abdominal mass, intussusception or appendicitis – where subsequent histology reveals the diagnosis. Other presentations include ongoing pain, abdominal distention and vomiting due to intestinal obstruction, obstructive uropathy, gastrointestinal bleeding or peritonitis from intestinal perforation [3, 4]. Surgeons thus need to be aware of the potential diagnosis which may influence surgical management in this heterogenous group of patients.

## Surgical Goals

- To provide adequate tissue sample for proper diagnosis (open biopsies/excision of accessible lymph nodes or mediastinal/abdominal masses)
- To treat surgical complications related to the disease (acute abdomen, for instance) or treatment
- To provide support:
  - venous access for chemotherapy (please refer to *the IPSO vascular access guideline*)
  - haemodialysis line insertion for tumour lysis syndrome
- Evaluation of abdominal distension after initiation of treatment (intestinal perforation, neutropenic enterocolitis, etc.)

## Preoperative: Evaluation, Images, Special needs, Biopsy Need?

**Patients may present to the surgeon in many different ways:**

- Referral for biopsy
- Referral for an indwelling line for chemotherapy
- Referral for Haemodialysis line insertion for tumour lysis syndrome
- As an incidental finding in a patient presenting with another typical childhood surgical condition, especially during surgery for an acute abdomen
- As a complication after initiation of chemotherapy

As with any patient, a thorough clinical assessment in the form of history and physical examination is required. Physical examination should include an assessment of the patient’s general condition, the location of any enlarged nodes, as well full assessment of the chest and abdomen, looking for masses or organomegaly. Other superficial areas include mandible swellings or patients presenting with suspicious mandibular masses and loose teeth. Uncharacteristic and uncommon skin lesions are also a rare form of lymphoma presentation.

Duration, characteristics and evolution of the mass, associated symptoms (fatigue, fever, night sweats, loss of weight, bruising and recurrent infections) may all be clues. In abdominal masses, associated pain, vomiting, constipation, GIT bleeding or urinary retention may suggest the nature of the disease process.

Full blood workup including blood counts, clotting profile, renal and liver functions as well as LDH need to be performed.

Imaging studies may include ultrasonography, CXR or cross-sectional imaging in the form of computed tomography (CT) Chest and abdomen or magnetic resonance imaging of the abdomen. Ultrasonography is the preferred initial investigation with the obvious advantages of lack of ionising radiation, and provides information on the size, shape and anatomy of the mass.

Mediastinal masses should have an initial chest X-ray with selected cases proceeding to CT scan for further information – with special emphasis on delineating the need for airway interventions during anaesthesia. If more than 50% of the tracheal lumen is compressed, needle or open biopsy should be performed under LOCAL anaesthesia only, as to avoid respiratory collapse. The use of steroids should be postponed until a biopsy is made, but in cases with severe impairment of the physiological status, with imminent risk of life, they should be considered, in agreement with the paediatric oncology staff.

Pre-operative consent is taken as per standard procedure, and in addition, surgeons should enquire whether further procedures such as line insertion, bone marrow biopsy, intrathecal chemotherapy or other relevant procedures should be included in the consent – this is particularly appropriate when frozen section is available – the results of which may inform decision and allow all of these procedures to happen in a single anaesthetic.

If the primary diagnostic work-up cannot identify the definitive cause of peripheral lymphadenopathy, a histopathological diagnosis is required. In general, children who present with a fast-growing tumour, or those who have a persistent lymphadenopathy despite being administered empirical antibiotics should be prepared to undergo a surgical biopsy. The possibility of malignancy usually drives surgeons to make this decision, and predictors include a fast-growing tumour, multiple levels of lymphadenopathy, supraclavicular location, hard or fixed nodes. Concomitant clinical symptoms such as persistent or unexplained fever, weight loss, organomegaly, abdominal pain associated with signs of intestinal obstruction (e.g. vomiting, abdominal distention, gastrointestinal bleeding) should be added to the aforementioned criteria for tumour biopsy.

An open biopsy to obtain adequate tissue samples for histopathological assessment is the preferred procedure. A significant lesion decided by clinical examination and preoperative imaging should be removed in part (incisional) or in total (excisional) for a precise pathological result. HL, NHL, neuroblastoma, leukaemia, rhabdomyosarcoma and metastatic diseases are the most frequent neoplasia’s associated with cervical lymph node enlargement.

Definitive diagnosis of Lymphoma requires histological evaluation and sufficient tissue needs to be obtained for immunophenotyping, cytogenetics and molecular biology testing in order to arrive at an accurate diagnosis. FNAC is generally not sufficient as the tissue yield is too low for a full pathological diagnosis. Core, incision or excision biopsy is preferred. Fine needle aspiration cytology (FNAC) can provide a minimally invasive diagnostic aid. However, interpretation of FNAC requires special cytopathology expertise. General anaesthesia is not needed for FNAC; therefore, FNAC may be used as the first-choice approach for patients with large mediastinal mass who are at risk of perioperative respiratory collapse.

The most superficial and easily accessible mass should be approached first – with the intention of minimising surgical morbidity. Thus, if a patient presents with a thoracic lesion as well as nodes in the neck, biopsy of the neck nodes would provide the least surgical morbidity.

The use of frozen section at the time of biopsy has the advantages of allowing sufficient histological assessment to make further decisions relating to line insertion, lumber puncture or bone marrow biopsy all in the same anaesthetic, thus streamlining management [3].

For deeper lesions, the use of core needle biopsy under radiological guidance is also an option – and in this instance surgical morbidity is avoided [3].

## Special Considerations for Lymph Node Sampling

1. Preoperative evaluation of mediastinum enlargement should be performed before a child is given general anaesthesia, due to the risk of ventilation problems and death during induction, when there is a significant mediastinal mass compressing the airway. In this situation, the surgeon should look for peripheral lymph nodes that can be biopsied under local anaesthesia or cavity fluid that can be obtained via thoracentesis or paracentesis to make a diagnosis.

Contraindications for general anaesthesia in patients with anterior mediastinal mass include more than 50% reduction of the predicted expiratory flow rate or more than 50% reduction of the predicted tracheal diameter. Orthopnoea is the most ominous predictor of respiratory collapse at the time of general anaesthesia [4].

If general anaesthesia is needed, the anaesthesiologist should be aware of the risks and adjust the sedation or anaesthesia accordingly. This may be by positional changes, performing the biopsy in a seated or lateral position, or with specific intubation techniques appropriate to the patient.

2. Sometimes, more than one biopsy may be needed to diagnose lymphoma. To avoid that situation, the surgeon should ensure that a representative size of tissue specimen is obtained. If the diagnosis is ‘lymph node reactive’ or ‘inconclusive’ on open biopsy, the patient should be followed up with further investigation until the case is resolved.

3. It is important to note that patients from countries where TB and HIV are prevalent may have concurrent different diagnoses.

(Please refer to *Management of Enlargement of Lymph Node Guideline*)

## Special Considerations for Abdominal Burkitt Lymphoma

Image guided procedures should be avoided when treating a child with suspected abdominal Burkitt lymphoma, due to the risk of intestinal perforation. In such cases, it is safer to perform an open biopsy by laparotomy or laparoscopy.

Endemic Burkitt lymphoma most commonly involves the jaw; however, abdomen primary is very common in both endemic and sporadic types. In the abdomen, the disease can preferably involve mesenteric lymph nodes (more common in high income countries) and/or preferably involve intestinal wall in various segments (frequent presentation in low- and middle-income countries (LMICs)). Patients with abdominal primary may present initially to surgeons, either with an asymptomatic abdominal mass or acute abdomen [5].

**Intussusception**

Intussusception is a well-known paediatric entity occurring in the majority of cases in patients under 2 years of age, and is usually idiopathic in nature. A lead point is found in less than 10% of cases, and these usually occur in older children and in patients over 4 years there needs to be a high index of suspicion that a lead point exists. While most lead points are benign, lymphoma of the bowel may well precipitate intussusception, with Burkitt’s lymphoma/mature B-cell NHL being the most common associated subtype, and post-transplant lymphoproliferative disease (PTLD) being an infrequent finding. In most instances, patients presenting with a lead point will progress to surgery either as a lead point was confirmed on ultrasound, the intussusception is not in the typical ileo-colic site, due to recurrence after reduction, or due to irreducibility on reduction enema attempts. Either way, the principles remain the same as for biopsy [7].

**Appendicitis**

Approximately just less than one third present with lower abdominal pain mimicking acute appendicitis. Therefore, patients may be explored in the acute setting and more commonly found to have a locally extensive disease involving the mesentery, retroperitoneal and intraperitoneal space.

**Surgical treatment**

The abdomen is the most frequent site of involvement in nonendemic Burkitt’s lymphoma [6]. For abdominal masses, the approach can be via open surgical techniques or laparoscopy, depending on the clinical situation as well as the facilities and expertise available. In all cases, it is important to remember that the goal is to safely sample representative lesions. Excision of complex masses is not indicated and destructive surgery should be avoided [3, 6]. If at all possible, biopsy of nodal masses or involved solid organs is preferred to biopsy of the bowel, where fistulation is a potential complication.

If the child presents with acute abdomen, the surgeon’s role is that of stabilisation of the abdominal condition.

If an unsuspected malignancy resulting in bowel obstruction or perforation is found, extensive resections and anastomosis should be avoided. Rather, a stoma to divert and control bowel contents and a biopsy of the lesion is appropriate – especially under emergent conditions. This can temporise the patient until referral to an appropriate centre and/or formal diagnosis is achieved.

The role of surgery should be diagnostic only and conduct of surgery should be as least invasive as possible to facilitate rapid recovery and starting of chemotherapy postoperatively. If obstruction and/or perforation is present, leading to the need of intestinal resection, stomas are safer until chemotherapy can be administered, for the tumour has rapid growth and involves the intestinal wall. If an anastomosis is performed, there is a high risk of anastomotic leak that leads to sepsis and death. Thus, stomas are safer in an acute setting. Less commonly the disease is limited to a short segment of bowel and mesentery, complete surgical resection of limited disease with end to end anastomosis is desirable because this result in de-escalation of therapy and excellent outcome. If that occurs, the surgeon must keep in close communication with the oncologist, in order to delay the initial chemotherapy, to avoid anastomotic leak.

Sometimes, perforation can occur after beginning of chemotherapy, for the intestinal wall melts with the tumour. In such cases, since the child will be neutropenic and with low platelet count, stomas should be indicated, instead of primary anastomosis.

Post chemotherapy abdominal disease is infrequent due to the efficacy of medication and thus the role of second look procedures is limited [6]. An infrequent complication after chemotherapy induces mucositis may be that of a bowel stricture [9]. Should this occur, resection and anastomosis are appropriate.

**Other conditions**

Primary gastric Burkitt lymphoma is very rare in childhood. Many gastric lymphomas including mucosa-associated lymphoid tissue lymphoma are associated with Helicobacter pylori infection or acute bleeding [8]. These patients would undergo endoscopic assessment and biopsy as per normal protocols.

PTLD: surgery may be needed to treat intestinal obstruction or severe gastrointestinal bleeding related to this situation.

Lymphomas can present infiltrating ovaries and testis. In such cases, gonadectomies should not be made, only biopsy for diagnostic confirmation. Renal infiltration can also occur. Image studies usually show bilateral involvement in gonadal and renal lymphomas.

**Support**

Surgery is of essence in providing adequate vascular access for chemotherapy and for dialysis, if needed.

Please refer to *IPSO guideline: Venous access for the paediatric cancer patient. Israel Fernandez-Pineda, Sharon Cox, Chan Hon Chui, Jörg Fuchs and Simone Abib*

## Post operation

In biopsy procedures, post operative period is usually uneventful.

In cases where intestinal resections with stomas were made, since usually an ileostomy is needed, care should be taken in order to prevent metabolic support and adequate fluid and electrolyte replacement. Nutritional support is of essence. The skin near the ileostomy should be early protected to avoid severe dermatitis.

Should the disease be localised and a primary anastomosis performed, close communication with the oncologist should be made, to postpone the initiation of chemotherapy.

## Complications

Infectious complications may be slightly higher in a population with bone marrow involvement as well as those on chemotherapy. Preoperative prophylactic antibiotics need to be given for surgical interventions to avoid these.

In case a stoma is made, care should be taken to prevent and treat metabolic, nutritional and fluid loss.

Dermatitis around the stoma is common. Early local and intensive care should be made.

## Tips

Be careful not to perforate the bowel while sampling the tumour for diagnosis.

This is a haemo-oncology disease that is treated by chemotherapy. Surgery is important for diagnosis and to treat complications. Avoid destructive surgery – chemotherapy is the mainstay of treatment in lymphoma.

Protect the skin as soon as the stoma is made in order to prevent severe dermatitis.

## Pitfalls

Sometimes more than one biopsy may be needed to diagnose lymphoma. To avoid that, be sure to biopsy a representative lymph node. It is important to remember that in countries where TB and HIV are prevalent, there could be concurrent different diagnoses.

Intestinal perforation can occur after the initiation of chemotherapy.

Intestinal perforation can occur while making a biopsy.

Should an intestinal resection be needed, avoid primary anastomosis, stomas are safer. In rare cases in which the disease is localised, resection and primary anastomosis can be made, but close communication with the oncologist in order to postpone chemotherapy is advised.

If there is gonadal involvement, refrain from doing gonadectomies, only biopsies are needed.

## References

[1] Foley RW, Aworanti OM, Gorman L (2016). Unusual childhood presentations of abdominal non-Hodgkin’s lymphoma. Pediatr Int.

[2] Morris-Stiff G, Cheang P, Key S (2008). Does the surgeon still have a role to play in the diagnosis and management of lymphomas?. World J Surg Oncol.

[3] Vural S, Baskin D, Dogan O (2010). Diagnosis in childhood abdominal Burkitt’s lymphoma. Ann Surg Oncol.

[4] Perger L, Lee E, Shamberger R (2008). Management of children and adolescents with a critical airway due to compression by an anterior mediastinal mass. J Pediatr Surg.

[5] Faizan M, Anwar S, Khan S (2018). Demographics and outome in paediatric non-Hodgkin Lymphoma: single centre experience at the children hospital Lahore, Pakistan. J Coll Physicians Surg Pak.

[6] Shamberger RC, Weinstein HJ (1992). The role of surgery in abdominal Burkitt’s lymphoma. J Pediatr Surg.

[7] Bussell HR, Kroiss S, Tharakan SJ (2019). Intussusception in children: lessons learned from intestinal lymphoma as a rare lead-point. Pediatr Surg Int.

[8] Kim SC, Hwang JW, Lee MK (2016). Rare case of primary gastric burkitt lymphoma in a child. Korean J Gastroenterol.

[9] Gupta G, Agarwala S, Thulkar S (2011). Jejunal stricture: a rare complication of chemotherapy in pediatric gastrointestinal B-cell non-Hodgkin lymphoma. J Pediatr Hematol Oncol.

# Discussion: Minimally invasive surgery in paediatric surgical oncology

**Published:** 2022

**Authors:** Rodrigo Chaves Ribeiro, Thomas Blanc, Hau D Le, Luca Pio, Max Pachl, Stefano Avanzini, Lucas E Matthyssens, Aurore Bouty, Piotr Czauderna

## 1. Introduction

Paediatric solid tumours include many different tumour types and can present in various locations throughout the body. Each type of solid tumour constitutes a different challenge and often requires a specific treatment protocol. Thus, the treating paediatric surgeon should work closely with the paediatric oncologist to develop a patient-tailored treatment plan, including surgical excision, based on the type of the tumour. This treatment plan should be discussed with a multidisciplinary team and include the timing of surgical resection and its approach. The benefits of minimally invasive surgery (MIS) are well established in non-oncologic surgery, but the role of MIS in paediatric solid tumours is still emerging and in many cases lacks proper evidence. To provide optimal outcomes for patients with solid tumours, the priority is to achieve an excellent quality of oncologic resection, regardless of the approach used. Therefore, surgeons who want to apply MIS to paediatric patients with solid tumours must have knowledge in both paediatric surgical oncology along with experience and expertise in paediatric MIS to provide optimal care and outcome for their patients. Selecting appropriate patients who can undergo MIS is vital and traditional criteria used are tumour size and location, patient’s condition and comorbidities, and local institutional expertise and support. Many advances in MIS have been developed in the last few decades that allow us to provide optimal surgical resection while minimising the pain and morbidity associated with open surgery.

Although MIS does carry some risks (such as air embolism, increased intraabdominal pressure during the procedure leading to decreased lung compliance and increased cardiac overload), a MIS approach offers well recognised advantages: reduced time to complete post-operative mobilisation and oral feeding, decreased use of narcotics, less bowel adhesion formation, decreased risk of wound infections and incisional hernias and earlier transition to postoperative adjuvant therapy. For all these reasons, in combination with the wide range of technical and ergonomic enhancements offered by robotic surgical technology, MIS is gaining more and more acceptance also in paediatric surgical oncology.

## 2. Incidence

The incidence of all paediatric neoplasms is estimated to be 100–164 new cases per million children under 15 years of age [1]. Solid tumours amendable for MIS approach are located mainly in abdomen or chest with applications that include biopsy and resection of primary and metastatic lesions. Common tumours that are potential candidates for MIS [2] resection are neuroblastoma (NBL) and ganglioneuroblastoma (7.5% of all tumours), renal tumours (6.3%), hepatic tumours (1.3%), gonadal tumours (1.9%) and others (see below). In addition, MIS approach can be applied to other types of cancer, like biopsy of thoracic and abdominal lymphoma, resection of pulmonary and hepatic metastatic lesions and ancillary surgical procedures such as ovarian sampling for fertility preservation and gastrostomy/enterostomy formation in case of bowel-related cancer complications.

## 3. Principles of Surgical Resection

The surgical treatment of many paediatric solid tumours is one component of a multi-disciplinary approach that is individualised based on the tumour staging and risk groups. The treating paediatric surgeon needs to work closely with the paediatric oncologist to develop an individualised treatment plan based on cooperative group treatment protocols or recommended therapies. The surgeon should be involved at the time of work-up to participate or to give input in the timing and method of tumour biopsy or to determine if early resection is feasible. In addition, there are differences in treatment protocols among different regions. Protocols are often tailored based on recommendations from the Children’s Oncology Group (COG) and International Pediatric Oncology Society (SIOP). In general, with few exceptions, a complete resection without spillage of the tumour is expected. Tumours located in abdomen or pelvis can be approached with laparoscopy, thoracic tumours by thoracoscopy.

The following criteria should be taken into account when considering MIS techniques for tumour resection: 1) Is MIS approach possible from technical standpoint? 2) Can the patient tolerate an MIS procedure? 3) Will the MIS approach achieve similar oncologic outcome compared to open approach?

The first criterion focuses on the tumour size and location. Since MIS needs working space around the tumour for visualisation and instrumentation, large tumours that occupy a large part of the abdomen may not be amendable to MIS. The second criterion focuses on patient factors. Since MIS requires pneumoperitoneum or pneumothorax (often with single lung ventilation), one must consider whether the patient’s physiology and comorbidities will allow this for the entire period of surgery. The third criterion focuses on the adherence to standard surgical oncology practices, irrespective of the MIS approach adopted. Proper surgical resection reduces the risk of local recurrence, may decrease the need for additional adjuvant therapy and alleviate perioperative complications. The last factor to be taken into account is the availability of a well-trained surgical and anaesthesiology team. One should keep in mind that other MIS limitations include higher costs and the need for special equipment and training.

## 4. Principles of MIS

MIS is considered a routine and safe surgical approach in most adult abdominal and thoracic procedures and many paediatric procedures, including neonatal surgery. In adult surgical oncology, MIS has also been shown to be an effective approach in abdominal oncological procedures such as colonic, gastric, prostate, kidney and gynaecology tumours. More advanced oncologic procedures such as liver and pancreatic resections have also been demonstrated.

MIS in paediatric oncology is still in the early stages. Its basis comprises surgery using specific equipment, adapted to the size of the patient. In laparoscopy, the procedure starts with the creation of a pneumoperitoneum, followed by the insertion of ports and the work with visualisation on a monitor.

Before starting a MIS procedure, the surgeon needs to reflect on the equipment needed, the patient’s position on the operating table, the patient’s fixation and the port positioning. The careful creation of a low-pressure pneumoperitoneum and placement of the first port are essential to avoid complications. In young children, we avoid the use of a Veress needle and prefer the first port placement under direct vision through a trans- or peri-umbilical incision (Hasson technique). The Veress needle can be helpful in adolescents if the tumour is not in the needle’s path, such as for pelvic, renal and adrenal surgery. Planning the port sites is essential for the smooth running of the surgery (Figure 1) and for this, we must remember the endoscopic principle of triangulation, where the optical port is in the centre, with a slightly posterior entrance, and the lateral ports with working angles between 60° and 90°. The surgeon should pay attention to the distance between the ports (avoid working with both arms too wide or too close) and to the table’s position and monitor, as ergonomics is important during lengthy procedures. The tumour extraction is done generally with a Pfannenstiel incision or enlargement of an abdominal or thoracic trocar incision.

Advanced laparoscopic energy devices, such as (Ultracision®/Harmonic®, Ethicon Endo-Surgery, Ohio, USA) and advanced bipolar forceps (Ligasure®, Covidien, Mansfield, MA, USA), are particularly useful in MIS oncology procedures.

## 5. MIS Approach to Renal Tumours in Children

Renal tumours represent 5%–11% of all paediatric malignancies. Their incidence is 7.1 cases per million children under 15 years. Nephroblastoma (Wilms tumour) is by far the most common tumour type in children, whereas clear cell sarcoma of the kidney, renal cell carcinoma, malignant rhabdoid tumour of the kidney and congenital mesoblastic nephroma are less frequent. The treatment of paediatric renal malignancies is multimodal (see IPSO Renal Tumor Guideline), depending on the histologic subtype, and structured in international protocols [3].

The major difference between the North American COG protocol and the SIOP protocol is the use of neoadjuvant chemotherapy for most cases, mainly without prior histology, in the latter. To date, the standard surgical treatment for unilateral Wilms tumours remains total nephrectomy with lymph node sampling via transverse transperitoneal laparotomy.

Since its first description in 2004 by Duarte *et al* [4], laparoscopic nephrectomy for renal tumours in children has gained in popularity with over 100 cases described [5–10]. However, controversies regarding this procedure persist [10].

One of the main questions raised is patient selection. Although not recommended in either protocol owing to lack of evidence, MIS is accepted in the UMBRELLA SIOP-Renal Tumor Study Group (SIOP-RTSG) 2016 protocol for small, central tumours surrounded by a rim of normal renal parenchyma, where lymph node sampling can still be performed [11]. It was initially accepted that only in tumours not extending beyond the lateral border of the spinal column should MIS be performed [12]. However, all authors now admit that indications can be expanded with experience [9, 13]. Another useful parameter to consider is the volume of the tumour [9, 11]. Wilms tumours usually present as large masses, often occupying at least half of the abdominal cavity, precluding the use of laparoscopic procedures. In this respect, the tumour-shrinking effect of chemotherapy might render MIS possible in a larger number of cases. Therefore, neoadjuvant chemotherapy might play a role in the development of MIS for the treatment of Wilms tumours [14]. Overall, laparoscopic nephrectomy is feasible in 20% of the cases after neoadjuvant chemotherapy, in experienced hands in both surgical oncology and MIS [8]. Currently accepted criteria for MIS nephrectomy in unilateral Wilms tumours are described in Table 1.

The surgical technique has been described by several authors [6, 13, 15]. Briefly, vessels are dissected first and ligated using Hem-O-Lok® (Teleflex). Care should be taken during dissection as large tumours can easily distort all anatomical landmarks. The goal of the surgery is to remove the kidney without intra-operative rupture, as this will upstage the tumour. Therefore, conversion should not be considered as a failure. Both trans- and retro-peritoneal approaches have been described and there is no evidence of superiority of any of those so far [10]. Robotic-assisted procedures have also been described with good results [16].

The other subject of debate is the ability to perform appropriate lymph node sampling using MIS. Although most studies report insufficient numbers of lymph nodes sampled during laparoscopic procedures [10], there was no difference in the number of lymph nodes sampled between open and laparoscopic procedures in a single-centre series [13].

Extraction in a specimen bag, through either a Pfannenstiel incision or an enlarged port site, has been described. Morcellation of the tumour is however formally contra-indicated as this precludes proper histologic staging [10].

Laparoscopic nephrectomy results in less postoperative pain and a shorter length of stay [17]. Although length of follow-up varies among series, the reported event-free survival after laparoscopic nephrectomy is 93%, which is comparable to open surgery for stage I tumours of favourable histology [10].

(see IPSO Wilms Tumour Tumour Guideline)

## 6. MIS Approach to Adrenal Tumours in Children

Adrenal tumours comprise a range of tumours of neuroectodermal origin, tumours such as neuroblastomas (NBLs), ganglioneuroblastomas and ganglioneuromas [18]. Rarer tumours include adrenocortical carcinoma (ACC) and pheochromocytoma (see IPSO Adrenal Tumour Guideline).

MIS may be an alternative in a relatively small number of NBL-patients due to this tumour’s tendency to adhere and involve large vessels. The presence of one or more Image Defined Risk Factors – especially those with vascular involvement – discourages the use of MIS, whereas their absence may suggest this approach [19–21]. Preoperative chemotherapy may significantly shrink large tumours; however, these may be very adherent to neighbouring tissues, leading to intraoperative difficulties or complications.

Adrenal tumours and carcinomas (ACC) can present with symptoms or signs of precocious puberty or Cushing’s syndrome. Despite some reports in the literature, MIS is not used routinely in paediatric ACC, as intraoperative rupture must be avoided, to prevent relapse risk [22].

Pheochromocytoma resection in children can be approached with MIS, provided an adequate operative blood pressure control is available, to contrast hyper- and hypotensive peaks due to tumour manipulation [23].

The operative approach to adrenal tumours differs according to the tumour side. A right adrenalectomy is technically more difficult than on the left side. The patient’s position will be oblique or lateral, similar to renal tumour surgery. On the right side, the first step is to release the liver’s triangular ligaments and free the liver from retroperitoneal adhesions. Usually, a fourth port is needed to separate the liver and achieve adrenal dissection. Great care must be taken with dissection of the adrenal vein, which is short and drains directly into inferior vena cava. On the left side, the need to mobilise the spleen and dissect it off the colonic flexure may increase the technical difficulties. In most cases, however, it easily suspended without significant dissection, and the adrenal vein is approached. Usually, the largest branch of the adrenal vein drains into the left renal vein. Adrenal tumours can be removed by slightly enlarging the umbilical incision or via a Pfannenstiel approach.

## 7. MIS Approach to Gonadal Tumours in Children

In ovarian tumours, primary tumour resection by MIS can be performed. However, a suprapubic Pfannenstiel incision will have to be made for the complete removal of the tumour (see IPSO GCT Guideline). For this reason, some authors prefer the open or hybrid technique. In these cases, laparoscopy allows a magnified view of the pelvis and a possibility to evaluate better the peritoneal cavity, such as evaluating diaphragmatic domes. Tumours that were resected in centres with no or little experience in paediatric surgical oncology and where information for proper staging is missing, may benefit from a MIS re-staging.

As for renal tumours, the surgeon must keep in mind that ovary sparing surgery criteria overcome those of MIS, the former being therefore the recommended approach, whenever there is no suspicion of malignancy.

In ovarian tumours, three ports are used. The surgeon and cameraperson stand on the tumour’s contralateral side, with the patient in a semi-oblique position (Figure 2). The first port is usually in the umbilicus, with the second close to the iliac crest and the third in-between the two, slightly away from their line to achieve an adequate triangulation. A vessel sealing device is helpful in sectioning of the wide, round ligament and the Fallopian tube. A Pfannenstiel incision is indicated to remove the piece if this cannot be retrieved through an enlarged umbilical incision. Peritoneal fluid collection should be sampled immediately before and after tumour resection, to provide adequate cytological diagnosis. Finally, inspection of the peritoneal cavity in search of implants or tumour invasion/metastasis completes the procedure.

Testicular tumours may benefit from MIS to complete retroperitoneal lymphadenectomy, which is usually performed after chemotherapy. In these cases, MIS is recommended only when the tumour does not involve or adhere to the large vessels. Lymphadenectomy may also be performed on a patient with an ovarian tumour that persists with a retroperitoneal mass after chemotherapy. In retroperitoneal lymphadenectomy, the patient’s position is crucial to access the space between the gonadal vessels and the aorta (on the left) or the vena cava (on the right side). The position is lateral, with trocars on the midline. The first step is a wide release of the colon until the large vessel’s visualisation, with exposure from the iliac vessels up to the renal hilum. A retroperitoneoscopic approach is also feasible for this procedure with the patient in full lateral position.

## 8. MIS Approach to Pancreatic Tumours in Children

Pancreatic tumours are rare in children (see IPSO Rare Tumour Guideline). Among the aetiologies, the main one is the pseudopapillary tumour of the pancreas (Frantz tumour). Surgical resection remains the primary treatment. Laparoscopic pancreatic surgery was developed and practiced in adult patients, bringing cosmetic benefits, less postoperative pain and shorter length of stay. There are only a few reports on laparoscopic pancreatic surgery in children due to its complexity and rarity [24, 25]. However, is it possible to attempt particular cases with an experienced team. Other pancreatic masses that may be amenable to MIS are neuroendocrine tumours such as insulinoma, although their intraoperative detection may be challenging and intraoperative ultrasonography may be helpful [25].

## 9. MIS Approach to Hepatic tumours in Children

Laparoscopic liver resection is one of the most challenging minimally invasive operations. For adults, a consensus has already been published on the subject [26]. Over the last decade, laparoscopic liver resections in paediatric hepatic tumours have been successfully performed, but most laparoscopic hepatectomies reported are case presentations and small case series of non-anatomical resections for small, peripheral and usually benign, lesions [27, 28]. Anatomic laparoscopic hepatic resections are uncommon and mostly deal with the so-called laparoscopic liver segments (2 to 6) and smaller tumours (<5 cm).

(Please also refer to *Hepatoblastoma and HCC Guidelines*)

## 10. Minimally Invasive Fertility Preserving Procedures in Children

(Please also refer to *Fertility Preservation Guidelines*)

With increasing overall survival, many paediatric cancer patients may be confronted with long-term morbidity, among which impaired gonadal function and fertility, with potentially important psychosocial consequences [29, 30]. It is therefore important and should be common practice, to consider and discuss gonadal function and fertility preservation for every paediatric cancer patient, before the start of gonadotoxic treatments (chemo-/radiotherapy). Depending on the gender of the patient and the treatment modalities needed, different interventions can be proposed.

Especially in girls, minimal invasive surgical techniques can offer a safe method for the preservation of fertility, with minimal postoperative morbidity and acceptable cosmesis.

When systemic gonadotoxic chemotherapy is indicated, ovarian tissue cryopreservation can be proposed in girls of any age. Depending on the size of the ovary, ovarian cortical strips can be harvested or an entire ovary may be excised (unilateral ovariectomy/oophorectomy) [31, 32]. This procedure can be performed by open approach (at the moment of tumour resection) or by laparoscopy. Scheduled a few days before the start of chemotherapy, the minimal invasive ovarian tissue harvesting procedure can be performed with one 5 mm port at the umbilicus (or 10 mm, depending on the patient size) for a 5 mm laparoscope, and two 3 mm working instruments. A low-pressure pneumoperitoneum is created by insufflating CO2 and the pelvis and internal genital organs are explored. In small children a unilateral laparoscopic ovariectomy (oophorectomy) is performed taking precautions not to injure the ipsilateral fallopian tube. The gonad is immediately extracted (by the umbilical incision), fixed in the appropriate solution and transported to the fertility laboratory for cryopreservation. After haemostatic control, the abdomen is desufflated and the fascia, subcutis and skin are closed with absorbable sutures. Most patients can go home 12–24 hours postoperatively and may start chemotherapy a few days later. Ovarian tissue cryopreservation in premenarchal girls is still considered experimental and application of the Edinburgh selection criteria is recommended [32]. In 2015, the first children born after autograft of cryopreserved ovarian tissue from childhood cancer survivors have been reported [33, 34].

In case of expected gonadotoxic abdominopelvic radiation treatment, one can also opt to reposition and fix one or both gonads outside the planned radiation field (laterally, or medially). This so-called ovarian transposition (oophoropexy) has been performed for over 50 years in adults by an open approach but has also been described by MIS and in paediatric patients [35–37]. The common laparoscopic approach uses one 5 mm umbilical port and two 3 mm working instruments to mobilise one or both gonads lateral and cephalad (as high as possible) with secure suture fixation to the peritoneum above the pelvic brim, outside the radiation target volume. It is recommended to mark each transposed gonad with metallic clips. This procedure has also been described by single incision and robotic approach [38, 39].

Upon indication, both minimal invasive procedures can also be combined [32].

For more details on gonadal function and fertility preservation options and outcomes in paediatric cancer patients, we refer to the separate ‘IPSO Guideline on Fertility Preservation in Children’.

## 11. MIS Approach to Thoracic Tumours in Children

Tumours in the chest can be categorised into mediastinal and pulmonary masses (see IPSO Thoracic Tumour Guideline). Common mediastinal tumours in children are thymoma (4%), germ cell tumours (19%), lymphoma (9%) and neurogenic tumours (46%), while the rest are cysts (8%) [40] Lung neoplasms in children are mostly either benign congenital lung lesions such as congenital pulmonary adenomatoid malformation, sequestration or metastatic disease in origin. Primary lung tumours in children are very rare [41] A single-institution 90-year review by Yu *et al* [42] reported 14 distinct histopathological types of primary lung tumours including carcinoid tumours, inflammatory myofibroplastic tumours, pleuropulmonary blastoma, small cell lung carcinoma, adenoma and pulmonary infantile haemangioma [42].

The application of a MIS approach for mediastinal and thoracic tumours needs to be individualised based on patient’s factors (age, size, conditions), tumour factors (size, location, invasiveness, etc.) and surgeon’s factors (experience, equipment, institutional support). As for abdominal tumours, the basic principles of MIS for oncology still apply here.

For mediastinal tumours such as thymomas, the patient is often positioned supine with the ipsilateral arm at 90° to allow port placement in the ipsilateral axilla and lateral chest wall. Visualisation is often enhanced with single-lung ventilation and angled optics (30°–70°). Energy devices such as electrocautery must be used with extreme caution to prevent injury to the phrenic, recurrent laryngeal (anterior mediastinum) or vagus nerve (middle mediastinum).

For primary pulmonary tumours, surgical resections are often performed via anatomical landmarks (lobectomy, bilobectomy or pneumonectomy). Patients are usually positioned in lateral decubitus or semi-prone and single-lung ventilation is often required. The use of oscillatory ventilation might be helpful in younger patients. Port placement is based upon tumour location. Surgeons need to follow oncological principles to prevent tumour rupture and spillage. Specimens should be placed in a spill-proof specimen bags for retrieval.

For metastectomy, MIS resections have been shown to be as effective as open surgery. A retrospective study from the Pediatric Surgical Oncology Research Collaborative Study shows that MIS metastasectomy patients have similar rates of overall survival and pulmonary recurrence when compared to open surgery [43]. Metastatic lesions are often detected on computed tomography scan or other cross-sectional imaging modalities. However, these lesions need to be localised prior to MIS resection because of the lack of tactile feedback and methods to localise these lesions will depend on local institution’s facilities. Some of the common localisation methods are: hookwires, dyes, microcoils, fiducial markers, contrast media and radiotracer. Metastectomy is usually performed using wedge resections and can be performed in both lungs at the same setting.

(see IPSO Pulmonary metastasis Guideline)

## 12. Upcoming Technologies

**Fluorescent guided surgery**

This technology is still in its infancy but is undergoing rapid evolution within adult and paediatric oncology surgery. It involves injecting a fluorochrome which is a molecule that emits photons when excited by laser or exposed to near-infrared light (NIR). During absorption of the light energy electrons are promoted from low to high levels and on return from their excited state energy is emitted as photons of light. The emissions are at a maximal wavelength of 832 nm and can penetrate up to 15 mm.

Indocyanine green (ICG) is the most commonly available fluorochrome and has been used in clinical practice in humans since 1956. Initially it was used to measure cardiac output, liver function and to study retinal vessels. It has an excellent safety profile although it can interfere with oxygen saturation readings because of the wavelength of light used to monitor oxygen saturation of haemoglobin. Once ICG is excited by NIR, fluorescence can be detected by specific cameras and the image transmitted to a screen for visualisation. ICG is cleared by the liver and excreted into the bile.

In oncology surgery, it has been used extensively for sentinel node mapping in adults for cervical, endometrial and breast cancer [44, 45]. The ICG is injected in the peri-tumoural area usually at four or more sites with a wait of around 15 minutes until it has penetrated into draining lymph nodes. Once this has occurred, the nodes can be resected.

Uses also include resection of liver tumours and identification of areas of ischaemia following bowel anastomoses.

In paediatric oncology [46], uses are still experimental and as an adjunct to standard surgery rather than as a replacement for it with most procedures being performed within clinical trials. However, it shows promise for use in pulmonary metastectomies, liver resections, sentinel node biopsies and non-hepatic resections.

**Robotic approach**

Robotic surgery (RS) is currently applied in almost all paediatric surgical diseases and in recent years was the more and more described for the management of selected solid tumours [47]. RS was most commonly applied to neuroblastic and renal tumours, with the main indications and limitation of conventional MIS [48]. To date there is no evidence of superiority of RS when compared to traditional minimally invasive approaches for paediatric solid tumours management [16]. Based on recent literature, the main recommendation of RS application to paediatric solid tumours is its use in a referral centre of paediatric surgical oncology with a high volume of robotic procedures in the same Institution.

## Figures and Tables

**Figure 1.** Trocar positioning of trocars for a laparoscopy nephrectomy.

**Figure 2.** Trocar positioning of trocars for a left salpingo-oophorectomy.

**Table 1.** Criteria for Laparoscopy Nephrectomy.

Tumour does not extend beyond the vertebral body
Possibility of hilum dissection
Without vascular extension
Without rupture
Team expertise in both oncological and MIS procedures

## References

[1] Stiller CA, Kroll ME, Eatock EM, Stiller C Incidence of childhood cancer 1191–2000. Childhood Cancer in Britain: Incidence, Survival, Mortality.

[2] Siegel RL, Miller KD, Fuchs HE (2021). Cancer statistics. CA Cancer J Clin.

[3] Fernandez C, Geller JI, Ehrlich PF, Pizzo PA, Poplack DG Renal tumors. Principles and practice. Pediatric Oncology.

[4] Duarte RJ, Denes FT, Cristofani LM (2004). Laparoscopic nephrectomy for Wilms tumor after chemotherapy. J Urol.

[5] Bouty A, Burnand K, Nightingale M (2018). What is the risk of local recurrence after laparoscopic transperitoneal radical nephrectomy in children with Wilms tumours? Analysis of a local series and review of the literature. J Pediatr Urol.

[6] Flores P, Cadario M, Lenz Y (2018). Laparoscopic total nephrectomy for Wilms tumor: towards new standards of care. J Pediatr Urol.

[7] Schmidt A, Warmanna SW, Urlaa C (2019). Patient selection and technical aspects for laparoscopic nephrectomy in Wilms tumor. Surg Oncol.

[8] Bouty A, Blanc T, Leclair MD (2020). Minimally invasive surgery for unilateral Wilms tumors: Multicenter retrospective analysis of 50 transperitoneal laparoscopic total nephrectomies. Pediatr Blood Cancer.

[9] Gavens E, Arul GS, Pachl M (2020). A single centre matched pair series comparing minimally invasive and open surgery for the resection of pediatric renal tumours. Surg Oncol.

[10] Malek MM, Behr CA, Aldrink JH (2020). Minimally invasive surgery for pediatric renal tumors: a systematic review by the APSA Cancer Committee. J Pediatr Surg.

[11] van den Heuvel-Eibrink MM, Hol JA, Pritchard-Jones K (2017). Position paper: rationale for the treatment of Wilms tumour in the UMBRELLA SIOP-RTSG 2016 protocol. International Society of Paediatric Oncology — Renal Tumour Study Group (SIOP–RTSG). Nat Rev Urol.

[12] Varlet F, Petit T, Leclair MD (2014). Laparoscopic treatment of renal cancer in children: a multicentric study and review of oncologic and surgical complications. J Pediatr Urol.

[13] Burnand K, Roberts A, Bouty A (2018). Laparoscopic nephrectomy for Wilms’ tumor: can we expand on the current SIOP criteria?. J Pediatr Urol.

[14] Warmann SW, Godzinski J, Tinteren H (2014). Minimally invasive nephrectomy for Wilms tumors in children—data from SIOP 2001. J Pediatr Surg.

[15] Duarte RJ, Dénes FT, Cristofani LM (2006). Further experience with laparoscopic nephrectomy for Wilms’ tumour after chemotherapy. BJU Int.

[16] Blanc T, Pio L, Clermidi P (2019). Robotic-assisted laparoscopic management of renal tumors in children: preliminary results. Pediatr Blood Cancer.

[17] Romao RL, Weber B, Gerstle JT (2014). Comparison between laparoscopic and open radical nephrectomy for the treatment of primary renal tumors in children: single-center experience over a 5-year period. J Pediatr Urol.

[18] Emre Ş, Özcan R, Bakır AC (2020). Adrenal masses in children: Imaging, surgical treatment and outcome. Asian J Surg.

[19] Shirota C, Tainaka T, Uchida H (2017). Resection of neuroblastomas in low- to high-risk patients without image-defined risk factors is safe and feasible. BMC Pediatr.

[20] Traynor MD, Sada A, Thompson GB (2020). Pediatr Surg Int.

[21] Fascetti-Leon F, Scotton G, Pio L (2017). Minimally invasive resection of adrenal masses in infants and children: results of a European multi-center survey. Surg Endosc.

[22] Lopes RI, Dénes FT, Bissoli J (2012). Laparoscopic adrenalectomy in children. J Pediatr Urol.

[23] Malkan AD, Loh AH, Sandoval JA (2014). Minimally invasive surgery in the management of abdominal tumors in children. J Pediatr Surg.

[24] Sokolov YY, Stonogin SV, Donskoy DV (2009). Laparoscopic pancreatic resections for solid pseudopapillary tumor in children. Eur J Pediatr Surg.

[25] Esposito C, De Lagausie P, Escolino M (2017). Laparoscopic resection of pancreatic tumors in children: results of a multicentric survey. J Laparoendosc Adv Surg Tech A.

[26] Wakabayashi G, Cherqui D, Geller DA (2015). Recommendations for laparoscopic liver resection: a report from the second international consensus conference held in Morioka. Ann Surg.

[27] Murawski M, Gołębiewski A (2021). Laparoscopic resection of liver tumors in children. J Pediatr Surg.

[28] Wang J, Jin S, Zhang Y (2020). A report of 21 cases of laparoscopic liver resection in children. J Laparoendosc Adv Surg Tech A.

[29] Chemaitilly W, Li Z, Krasin MJ (2017). Premature ovarian insufficiency in childhood cancer survivors: a report from the St. Jude Lifetime Cohort. J Clin Endocrinol Metab.

[30] van den Berg MH, Dijk M, Byrne J (2021). Treatment-related fertility impairment in long-term female childhood, adolescent and young adult cancer survivors: investigating dose-effect relationships in a European case-control study (PanCareLIFE). Hum Reprod.

[31] Imhof M, Hofstetter G (2004). Cryopreservation of a whole ovary as a strategy for restoring ovarian function. J Assist Reprod Genet.

[32] Wallace WH, Smith AG, Kelsey TW (2014). Fertility preservation for girls and young women with cancer: population-based validation of criteria for ovarian tissue cryopreservation. Lancet Oncol.

[33] Demeestere I, Simon P, Dedeken L (2015). Live birth after autograft of ovarian tissue cryopreserved during childhood. Hum Reprod.

[34] Donnez J, Dolmans MM, Diaz C (2015). Ovarian cortex transplantation: time to move on from experimental studies to open clinical application. Fertil Steril.

[35] Williams RS, Mendenhall N (1992). Laparoscopic oophoropexy for preservation of ovarian function before pelvic node irradiation. Obstet Gynecol.

[36] Thibaud E, Ramirez M, Brauner R (1992). Preservation of ovarian function by ovarian transposition performed before pelvic irradiation during childhood. J Pediatr.

[37] Morice P, Castaigne D, Haie-Meder C (1998). Laparoscopic ovarian transposition for pelvic malignancies: indications and functional outcomes. Fertil Steril.

[38] Ben Dhaou M, Zouari M, Jallouli M (2015). Single-port laparoscopic ovarian transposition in an 11-year-old girl. Arch Pediatr.

[39] Molpus KL, Wedergren JS, Carlson MA (2003). Robotically assisted endoscopic ovarian transposition. JSLS.

[40] Takeda S, Miyoshi S, Akashi A (2003). Clinical spectrum of primary mediastinal tumors: a comparison of adult and pediatric populations at a single Japanese. J Surg Oncol.

[41] Lichtenberger JP, Biko DM, Carter BW (2018). Primary lung tumors in children: radiologic-pathologic correlation from the radiologic pathology archives. Radiographics.

[42] Yu DC, Grabowski MJ, Kozakewich HP (2010). Primary lung tumors in children and adolescents: a 90-year experience. J Pediatr Surg.

[43] Lautz TB, Farooqui Z, Jenkins T (2021). Thoracoscopy vs thoracotomy for the management of metastatic osteosarcoma: a Pediatric Surgical Oncology Research Collaborative Study. Int J Cancer.

[44] Jung SY, Han JH, Park SJ (2019). The sentinel lymph node biopsy using Indocyanine green fluorescence plus radioisotope method compared with the radioisotope-only method for breast cancer patients after Neoadjuvant chemotherapy: a prospective, randomized, open-label, single-center phase 2 trial. Ann Surg Oncol.

[45] Vermersch C, Raia-Barjat T, Chapelle C (2019). Randomized comparison between indocyanine green fluorescence plus (99m) technetium and (99m) technetium alone methods for sentinel lymph node biopsy in breast cancer. Sci Rep.

[46] Goldstein SD, Heaton TE, Bondoc A (2021). Evolving applications of fluorescence guided surgery in pediatric surgical oncology: a practical guide for surgeons. J Pediatr Surg.

[47] Meehan JJ (2013). Robotic surgery for pediatric tumors. Cancer J.

[48] Meignan P, Ballouhey Q, Lejeune J (2017). Robotic-assisted laparoscopic surgery for pediatric tumors: a bicenter experience. J Robot Surg.

# Discussion: Surgical emergencies and complications in paediatric oncology

**Published:** 2022

**Authors:** Sharon Cox, Ahmed Elgendy, Paul D Losty, Chan Hon Chui, Abdulrasheed Nasir

More than 12,000 new malignancies are diagnosed annually among children and adolescents in the USA [1].

Although the overall outcome in the majority of paediatric tumours is favourable, life-threatening emergencies can occur which add significant morbidity and mortality [2]. Multidisciplinary team co-operation is fundamental in such emergencies to establish a prompt diagnosis and formulate a management plan. This may result in urgent, lifesaving operative procedures. These surgical emergencies can be attributed to the tumour itself or as a consequence of its treatment. This article aims to discuss clinical criteria and management of surgical emergencies and complications in paediatric oncology patients.

## Classification of commonly encountered surgical emergencies:

### Emergencies Related to Chemotherapy: Part 1 (page 219)

- Typhlitis/bowel perforation
- Pancreatitis
- Cholelithiasis
- Gastrointestinal (GI) haemorrhage
- Haemorrhagic cystitis
- Anorectal complications
- Invasive fungal infections
- Extremities

### Emergencies Related to Tumour Bulk or Mass Effect: Part 2 (page 232)

- Bowel obstruction, perforation and gangrene
- Intussusception
- Torsion of ovarian tumour
- Ruptured solid tumours/malignant haemoperitoneum
- Urinary tract obstruction
- Spinal cord compression

### Emergency Access Procedures: Part 3 (page 248)

- Chest tube or pigtail insertion: pleural effusion, haemothorax or tension pneumothorax
- Removal of indwelling line: line sepsis
- Dialysis catheter insertion: tumour lysis syndrome (TLS)
- Vascular access: leukaemia with hyperleukocytosis
- Tracheostomy insertion: airway obstruction

### Surgical Biopsies in the Management of BMT Patients: Part 4 (page 256)

# Discussion: Emergencies related to chemotherapy

**Published:** 2022

**Authors:** Sharon Cox, Ahmed Elgendy, Chan Hon Chui, Abdulrasheed Nasir, Paul D Losty, Humberto Enrique Mejia Alvarez, Jaime Shalkow, Giorgio Persano, Joyce Freitas, Alessandro Crocoli

(Please also refer to *Pediatric Surgery in the Immunocompromised patient/BMT guideline* – page 259)

## Introduction

The systemic effects of the primary disease and the multimodal therapy of cancers often cause malnutrition and myelosuppression, in particular, thrombocytopenia and immunosuppression. The clinical presentations are often modified by immunosuppression, rendering initial diagnosis difficult. Any invasive surgical procedure is associated with high risk of morbidity and mortality. Surgeries undertaken may potentially be affected by delayed wound healing, bleeding diathesis and infection risks, leading to subsequent delay in anti-neoplastic therapy that may adversely affect the overall treatment efficacy [3].

The immunosuppressed patients, including those who underwent bone marrow transplantation (BMT), may also develop complications that require surgical management. These may include typhlitis, pancreatitis, cholelithiasis, GI haemorrhage, Haemorrhagic cystitis, anorectal complication, invasive fungal and extremity infections [3].

In addition, the BMT patient may also develop graft-versus-host disease (GvHD). Clinical manifestations depend on the degree of donor/recipient human leucocyte antigen (HLA) incompatibility and graft alloreactivity to major host antigens. Primary affected organs are skin, liver and GI tract, although other sites may be affected [4].

## Neutropenic Colitis (Typhlitis)

### Definition:

- Neutropenic colitis (NC) or typhlitis is defined as a necrotising inflammation of the caecum and colon, in the presence of neutropenia that could be life-threatening if not recognised and treated early [5].
- It was first described as a complication of chemotherapy agents used in childhood leukaemia patients, but now it is known to have other causes as well (Table 1) [5–8].

### Pathophysiology

- Pathophysiology concepts underlying the development of NC are not fully characterised but it is thought that mucosal damage is the first step on this cascade [9, 10]. Stasis, shock, sepsis, bacterial translocation and delay in mucosal healing occur. The typical lesions described are marked haemorrhagic necrosis, mucosal ulceration, extensive oedema in the submucosa and lamina propria, congestion and mural necrosis with a paucity of infiltrative inflammatory cells, with or without infiltrative bacteria [11]. It may involve any segment of bowel but usually involves the caecum, ascending colon and less frequently terminal ileum and jejunum [9–12].

### Microbiology

- Many microorganisms have been associated to the development of sepsis in patients with NC. Blood cultures may show *Streptococcus* species, *Escherichia coli, Enterococcus faecium, Candida albicans, Candida glabrata, Klebsiella* species, *Bacteroides fragilis* [13–15], *Pseudomonas aeruginosa* [6, 16] and less frequently mucormycosis [17] or even influenza [18].

### Epidemiology and risk factors:

- The incidence of NC can be estimated at 2.6%–5% of paediatric patients hospitalised for leukaemia, lymphoma, AIDS or patients maintained on immunosuppressive agents [2, 14, 15], and has a mortality rate of 2.5%–5% [9].
- It occurs more often in haematological malignancies than solid tumours [16], especially in acute myeloid leukaemia and Burkitt’s lymphoma.
- Intensive chemotherapy and BMT are contributory factors.
- Drugs that may be implicated include anthracyclines and cytosine arabinoside which are known to cause mucosal ulcers, and vincristine which causes bowel hypomotility, though others may be associated.
- The most frequent mentioned risk factors are the presence of mucositis, neutropenia <500 cells/µL, chemotherapy in the 2 weeks before the onset of typhlitis, history of haematopoietic stem cell transplantation (HSCT), corticosteroid administration and the presence of a central venous catheter (CVC) [10, 12, 19].

### Clinical findings:

- The most commonly described symptoms of NC are the triad of abdominal pain, fever above 38°C and neutropenia (ANC < 1,000/µL) [20], but abdominal tenderness, diarrhoea, bloody diarrhoea, mucositis, emesis, abdominal bloating and nausea have also been described [14, 15, 19, 21].
- Clinical findings of hypotension, peritonitis, septic shock unresponsive to intravenous fluid resuscitation, septic emboli, intussusception and signs of perforation are all associated with a more severe course [12, 21, 22].

### Imaging:

- Imaging is indicated in neutropenic patients with abdominal pain and fever. Initial diagnostic work-up includes plain abdominal X-ray to rule out pneumoperitoneum [8]. Other radiologic findings vary from a nonspecific bowel gas pattern to frank, right colonic pneumatosis intestinalis [7], however, such findings are rarely diagnostic [6].
- Ultrasound scan (US) is helpful in the diagnosis and may reveal thickening of the caecum, transverse colon or terminal ileum of >3 mm, which is highly suggestive of NC in association with typical symptoms [5, 16]. Thickening above 0.5 cm is related to poor prognosis.
- Computed tomography (CT) imaging may confirm bowel wall thickening and reveal pneumatosis intestinalis and/or pneumobilia that may help differentiate NC from other conditions with similar clinical presentation such as *Clostridium difficile* colitis. Another tomographic sign of NC includes fatty streaks on the affected bowel segment [23].
- To date, there is not sufficient evidence supporting the preferential use of US or CT, and imaging is accordingly utilised to local availability of the modality.
- Some studies report a mortality of up to 60% of patients with bowel wall thickening > 10 mm [24].

### Management:

- Initial non-surgical management of NC is the treatment of choice and includes bowel rest, naso-gastric (NG) tube placement, total parenteral nutrition, administration of appropriate blood products [6] and broad spectrum antibiotics with Gram-negative coverage, such as antipseudomonal β-lactam, a fourth-generation cephalosporin or a carbapenem, with the addition of a second Gram-negative agent or a glycopeptide for patients who are clinically unstable, when a resistant infection is suspected, or for centres with a high rate of resistant pathogens [25].
- Metronidazole as a first-line agent for pseudomembranous enterocolitis coverage and empiric antifungal coverage is also recommended, especially in patients who are neutropenic for over 96 hours [6, 25, 26].
- In patients who are hypotensive at first presentation, administration of an initial fluid bolus of 20 mL/kg of crystalloids is advisable [27]. Patients who fail to respond to initial bolus fluids should be evaluated for admission to Intensive Care Unit. Most patients will respond to medical therapy [6, 16, 21, 28].
- Feeding can recommence in the absence of abdominal pain, when bowel transit is restored, and leucocytes are above 1,500.

### Indications for surgery [18]:

- Persistent GI bleeding after resolution of neutropenia, thrombocytopenia and correction of clotting abnormalities, especially if the site of bleeding is known. It can, however, be difficult to find the source of bleeding looking at the bowel from its serosal surface.
- Evidence of free intraperitoneal perforation. Perforation frequently occurs when the patient is recovering the leucocyte count after an episode of immunosuppression, when the inflammatory response is greater and facilitates perforation.
- Clinical deterioration requiring support with vasopressors, or large volumes of fluid, suggesting uncontrolled sepsis together. In this situation, a necrotic loop may be present. In order to decide if surgery is indicated or not, the most experienced surgeon should repeatedly evaluate the patient.
- Intestinal obstruction
- Abscess (could be drained percutaneously if localised)
- Development of symptoms of an intra-abdominal fulminating process, in the absence of neutropenia, which would normally require surgery.

An algorithm for the evaluation and management of NC patients is outlined in Figure 1 [29].

### Surgical goals:

- The aim of surgical treatment in NC patients is to resect all the necrotic and compromised areas, to control bleeding, and/or divert bowel content and achieve local control of the infection [3, 6, 20, 30, 31]. Care should be taken to avoid excessive resection of bowel so as to avoid short bowel syndrome.

### Surgical approach:

- Standard surgical approach includes emergent laparotomy and resection of necrotic bowel. Since the caecum and the right colon are the most severely affected areas [7, 32, 33], right hemicolectomy is often the procedure of choice, extended if necessary to resect further necrotic areas of disease.
- If there is evidence of significant soilage of the peritoneal cavity or the patient is haemodynamically unstable, needing large volume of fluids, vasopressors or inotropes to maintain blood pressure, ileostomy and mucous fistula are the safest options. If the patient is stable and there is minimal peritoneal soilage primary intestinal anastomosis can be considered [6, 20, 21, 33–37], but associated neutropenia needs to be taken into account. Most of the time, performing osteotomies is safer.
- Less extensive procedures such as simple drainage of necrotic areas, appendectomy or cecostomy may be insufficient for the definitive management of NC as the extent of mucosal necrosis may often be greater than that which is obvious by macroscopic inspection of the bowel [32, 35, 36].
- Laparoscopy or diagnostic peritoneal lavage can be considered a diagnostic tool for perforation or necrosis, when CT is inconclusive.

### Complications

- Complications in the post-operative period include wound infection, intra-abdominal sepsis, respiratory failure and wound dehiscence [31, 32]. Long-term complications have been described, such as entero-colic or biliary fistulae, and entero-cutaneous and/or entero-atmospheric fistulae [28]. Such complications are mainly related to the infective complications secondary to prolonged neutropenia [37] and to the haemodynamic changes induced by septic shock in the mesenteric circulation, causing mesenteric ischaemia [38].
- Progression of bowel ischaemia after exploratory laparotomy requiring ‘second-look’ surgery has been described [33] as well as recurrence of NC after conservatively treated episodes that required surgical treatment [36, 39].

### Postoperative considerations:

- Admission to the Intensive Care Unit after the surgical procedure is recommended for haemodynamic monitoring and support. Continued postoperative sepsis until recovery of neutrophil counts should be anticipated and therefore appropriate antibiotic and antifungal therapy is of critical paramount importance [25, 35, 36].
- Parenteral nutrition is usually necessary in the early post-operative period [6]. Enteral feeding plays an important role for the integrity of intestinal mucosa; ‘nil po’ regimen should be limited to the acute phase of illness and enteral feeding should then be restored as soon as the patient can tolerate it [8].
- Transfusion support with platelets may be necessary to maintain the platelet count above 50,000/μL. If patients have coagulation defects or disseminated intravascular coagulation (DIC), fresh frozen plasma (FFP) may be needed as well to replace clotting factors. Cryoprecipitate may be necessary to treat hypofibrinogenaemia [6].

### Tips

The earlier correct clinical treatment is instituted, the less surgical treatment will be needed. Waiting for neutropenia resolution in the face of failure of conservative treatment might expose patients to a septic degradation course toward septic shock. Inadequately treated typhlitis carries a high risk of death and the lack of access to surgical management is a significant adverse prognostic factor. Delaying surgical therapy due to neutropenia, thrombocytopenia or other chemotherapy or malignancy associated reasons is not recommended and may be detrimental; however, in these circumstances, patients may still die regardless of surgical intervention [40].

## Pancreatitis

### Epidemiology

Pancreatitis is the inflammation of the pancreas and is diagnosed when the serum amylase or lipase is significantly elevated, with clinical signs and radiological findings consistent with pancreatitis. L-asparaginase associated pancreatitis is most commonly encountered.

Of paediatric oncology patients who receive L-asparaginase, 1%–18% will develop pancreatitis [41]. Asparaginase associated pancreatitis generally occurs within the first few weeks of therapy and is believed to be related to possible underlying predisposition rather than the cumulative effect of the drug. Older children and adolescents (10 -18 years) are at higher risk of developing pancreatitis. Acute lymphoblastic leukaemia (ALL), which is treated with L-asparaginase, systemic steroids and 6-mercaptopurine, is the neoplasm most commonly associated with pancreatitis in this age group.

### Clinical presentation

Patients would usually present with abdominal or back pain with nausea or vomiting. About 18% developed *pancreatic* pseudocyst [42].

### Workup

Complete blood count, serum amylase and serum lipase. Elevated levels of serum amylase and lipase would be diagnostic.

Radiological imaging. Ultrasonography and CT scan would usually show oedematous pancreatitis, with necrosis, peripancreatic collections and pseudocyst formation. As both imaging techniques show a high degree of concordance in findings, good USs may be sufficient. A reasonable indication for CT scan is to distinguish acute pancreatitis from another serious intra-abdominal condition. Chest radiograph may show signs of acute respiratory distress syndrome and pleural fluid.

### Management

It is usually self-limiting. The need for surgical intervention is rare and long-term sequelae are not usually observed.

Non-operative management is recommended as the initial care of patients with pancreatitis. It often responds favourably to NG decompression, bowel rest and intravenous hyperalimentation. Data to support the role of somatostatin is limited. Total parenteral nutrition should be initiated, and chemotherapy suspended until acute pancreatitis resolves. Oral feeding may be commenced when abdominal pain subsides and diet advanced as tolerated.

However, complications such as severe haemorrhagic pancreatitis, necrosis or pseudocyst formation may occur. Controversies exist over the management of pancreatic pseudocysts, with options of surgery, percutaneous drainage or without intervention. Surgical treatments available include endoscopic, laparoscopic or open cystgastrostomy but all require sufficient maturation of the cyst wall, which usually takes between 4 and 6 weeks. Safety in observing asymptomatic pseudocyst has been reported [43].

### Prognosis

Significant morbidity may arise when severe pancreatitis develops during neutropenia resulting in multi-organ dysfunction, haemorrhage, sepsis and pseudocyst formation. Mortality has been reported between 2% and 15% [42]. ALL patients who developed pancreatitis are more likely to relapse in their malignancy and have poorer event-free survival than those without pancreatitis [41, 42].

## Cholelithiasis and Cholecystitis

### Epidemiology

Cholelithiasis is a rare disease of children with an incidence of 0.13%–0.21% [44, 45]. Children with cancer show a higher risk for developing cholelithiasis (1.03%) than the general population [46]. This higher incidence has been attributed to an increased number of risk factors including previous abdominal surgery or radiation, ileal conduit and total parenteral nutrition [47]. Patients who had BMT exhibit many risk factors associated with cholelithiasis [48]. Children undergoing BMT for bone marrow failure are at higher risk of developing gallstones than those being treated for malignancy. Of note, acute acalculous cholecystitis may develop in the presence of stress, sepsis and co-existing conditions such as leukaemia.

### Clinical presentation

Most cases present with jaundice, fever and abdominal pain. In few cases, the diagnosis may be incidental on ultrasound evaluation. It is important to remember that biliary obstruction resulting from external pressure due to adjacent tumour bulk could result in similar symptoms.

### Workup

Complete blood counts, clotting profile, liver function test.

Imaging: Abdominal US.

### Surgical management

Surgery is indicated in children who develop cholecystitis. Depending on the haematological status, clinical treatment is initiated until the patient has conditions to be operated safely. Surgical management includes cholecystectomy (laparoscopic or open) or cholecystostomy, in association with intravenous antibiotics. Indications for urgent cholecystectomy include biliary peritonitis Cholecystostomy is useful for selected patients who require an operation but are considered unfit for the procedure. Bile duct stones should be treated endoscopically after recovery from immunosuppressive therapy, unless cholangitis and severe jaundice occurs, asymptomatic children with cholelithiasis should be managed nonoperatively [48].

### Outcome/Prognosis

In a retrospective study on the management of cholelithiasis in children who underwent BMT, most did not require surgical intervention. 45% died from their primary disease, 25% had resolution of the gallstones and 15% continued survival with asymptomatic gallstones, while 15% progressed to acute cholecystitis requiring surgical intervention [48]. Non-operative management is safe and therefore recommended for the asymptomatic patient.

## Gastrointestinal Haemorrhage

### Epidemiology

Significant GI haemorrhage is when there is loss of blood in the GI tract requiring blood transfusion. Very often these patients have concomitant thrombocytopenia secondary to myelosuppression from chemotherapy or coagulopathy that may complicate its management. Without adequate control of the bleeding, further anti-neoplastic treatment may be delayed.

There are diverse causes of GI haemorrhage in paediatric oncology patients. These include those that are a result of immunosuppression (infections like CMV or typhlitis), portal hypertension (varices), tumour recurrence with GI invasion, post-transplant lymphoproliferative disorder, steroidal effects (peptic ulcers, erosions), radiation effects (gastritis, proctocolitis), Cushing ulcer (raised intracranial pressure) and causes unrelated to treatment (like intussusception, Meckel’s diverticulum, juvenile polyps, vascular malformation).

### Clinical presentation

Patient will present with varied degree of GI bleeding.

### Workup

Complete blood counts performed serially to monitor ongoing bleeding. Coagulation profile.

Plain abdominal X-ray (to look out for toxic dilatation). Ultrasonography of abdomen.

Endoscopy (oesophagogastroduodenoscopy, colonoscopy) is often the preferred initial evaluation as it is likely to diagnose the majority of the conditions with a potential of concurrent treatment. In the evaluation of the small intestines, technetium red blood cell scan and capsule endoscopy are available options.

### Surgical management

Adequate initial resuscitation is crucial. This is followed by the correction of any coagulopathy which may improve haemostasis and render the patient suitable for any surgical intervention if required.

Many endoscopic techniques are available for haemostasis, including clip application, sclerotherapy, ligation of varices and argon plasma coagulation. Such minimally invasive techniques would allow early recovery and shorten the delay in subsequent anti-neoplastic therapy. Exploratory laparotomy with intra-operative enteroscopy would be indicated if endoscopic option of treatment did not work.

## Anorectal Complications

### Epidemiology

Anorectal complications and severe soft tissue infections are more frequent in patients with haematological malignant diseases as compared with patients with other cancers, especially after BMT. Early identification of predisposing factors, early diagnosis and prompt treatment are mandatory for reducing morbidity and mortality. *Pseudomonas Spp. and Klebsiella Spp*. are the most common agents involved with these conditions.

### Clinical presentation

Patients may suffer from different conditions, varying from simple perianal abscess, haemorrhoids or anal fissures, to severe conditions like necrotising fasciitis involving perineum and scrotum (Fournier’s gangrene) or ecthyma gangrenosum. Soft tissue lesions in the perineum can have devastating effects (i.e. tissue destruction and sphincter damage). For these reasons, patients with any lesion in the perineum/anal area should be examined daily in order to evaluate the progression or regression of the infection locally and determine the need for surgical procedures.

### Workup

Nursing care is paramount, and the extent of the lesions should be marked and size changes documented. All lesions have to be examined by experienced surgeons and frequent examination of the lesion by trained nursing staff is imperative.

Aplastic patients in the early phase of recovery after BMT should routinely have blood cultures, gut surveillance swabs taken and specific antimicrobic agents started intravenously in addition to intestinal decontamination to eradicate gut carriage of the organisms.

### Surgical management

Treatment should be performed by a multidisciplinary team consisting of surgeons, oncologists, microbiologists and nurses. Surgical strategies may include different procedures such as early debridement or drainage of abscess, local wound care with advanced dressing (to be performed in-consultation with plastic surgeon if required) or extensive surgical procedures including colostomy, debridement and perineoplasty with delayed skin graft in patients with frank necrosis and necrotising fasciitis.

## Haemorrhagic Cystitis

### Epidemiology

Haemorrhagic cystitis refers to is the inflammation of the bladder with bleeding. It is defined by the presence of sustained haematuria and lower urinary tract symptoms, such as dysuria, frequency and urgency. It occurs in 10%–70% of paediatric patients undergoing paediatric bone marrow transplantations [49]. The potential for life-long sequelae from fibrosis and contraction of the bladder is significant of great consequence in paediatric populations.

Paediatric patients who undergo BMT and those who received high-dose cyclophosphamide, ifosfamide and busulfan are predisposed to developing haemorrhagic cystitis. Prior or concurrent pelvic irradiation is also a risk factor. In addition, viral infections like BK virus, adenovirus and CMV may be responsible. Patients who have a predisposition for haemorrhagic cystitis include those older than 10 years of age and those who underwent allogeneic transplantations.

### Clinical presentation

The patients may present with various grades of haematuria, from microscopic to gross haematuria with clots causing urinary tract obstruction that may require instrumentation. Clinical symptoms may occur hours, days, weeks or years after chemotherapy administration, with recurrence of bleeding. Haemorrhagic cystitis may also be associated with vesico-ureteric reflux and hydronephrosis, leading to beyond which renal dysfunction and renal failure in selected cases.

### Workup

Determination of virus titres in blood and urine samples would determine its aetiology. Ultrasonography of the renal tract should be performed to detect upper tract dilatation from clot obstruction.

Electrolyte, Urea and creatinine: Renal function should be monitored closely.

### Management

Preventive measures are effective in reducing the incidence of haemorrhagic cystitis. Vigorous prophylaxis to minimise contact between noxious metabolites and the bladder mucosa by hyperhydration, continuous bladder irrigation or administration of thiol compound, sodium 2-mercaptoethane sulfonate (mesna). Mesna combines with the metabolites of ifosfamide and cyclophosphamide to form nontoxic compounds in the urine. This may reduce the incidence of haemorrhagic cystitis to <2 %. Once haemorrhagic cystitis occurs, hyperhydration, continuous bladder irrigation, platelet transfusion and treatment of existing coagulopathy are recommended. Oxybutinin chloride may provide symptomatic relief of the associated bladder spasms. In the presence of urinary obstruction, cystoscopic removal of clots, placement of suprapubic catheter, bladder washouts may be helpful. Intravenous or intravesical antiviral therapy is effective in the treatment of viral infections. Refractory bleeding may be treated with Nd:YAG laser-induced coagulation, electrocautery, local instillation of prostaglandin E1, alum, silver nitrate or formalin [49]. Bladder resection is a rarely required option. The choice of surgical options depends on age of patient, size of urethra, availability of instruments and long-term sequelae of therapeutic options.

## Invasive Fungal Infections (Invasive Aspergillosis)

### Epidemiology

Invasive aspergillosis (IA) is one of the several types of fungal infections that have been encountered in paediatric oncology patients. It almost exclusively affects individuals with weakened immune systems due to cancer, HIV infection AIDS, leukaemia, organ transplantation or chemotherapy. It occurs in patients at HSCT (2.3%–7.3%) [50], at induction chemotherapy, at relapse of a malignancy, with neutropenia for more than 10 days, or receiving high-dose corticosteroids. It most often occurs within the first 100 days after HSCT. It is associated with a mortality of 40%–50 % in those who received chemotherapy, and 90% in those with HSCT [51].

### Clinical presentation

Aspergillus is an airborne infection and lung is the most common site of infection, followed by skin, sinus, nasal and cerebral disease. About 86% of the infections reported are localised [52] and the rest are disseminated. Patients with pulmonary aspergillosis may present with cough, fever and sputum, tachypnoea, pleuritic chest pain or even haemoptysis. Some patients may be asymptomatic and diagnoses are made based on incidental finding of pulmonary nodules or other radiographic results.

### Workup

Complete blood count. Typically shows neutropenia. Sputum examination.

Sputum stains and culture for Aspergillus, and Galactomannan enzyme immunoassay tests may be diagnostic. Often, the definitive diagnosis may require histopathologic examination of pulmonary or other involved tissue.

Imaging: Chest radiographs show wedge-shaped peripheral areas of consolidation usually extending to the pleural surface, with or without cavitation. CT scan of the thorax shows nodules, cavitation, halo sign or air-crescent sign.

### Management

Once IA is detected, aggressive operative treatment of fungal infections combined with antifungal chemotherapy before transplantation may offer the best hope of extended survival. Antifungal drugs currently available include amphotericin B, voriconazole, itraconazole and caspofungin. Combined administration of antifungal drugs with synergistic action improves the prognosis.

Surgical intervention is indicated when antifungal therapy fails to completely eradicate IA and when confirmation of diagnosis is required. Lung-sparing resection should be the aim, though lobectomy could be performed when required. Eradication of the disease is recommended before high-dose chemotherapy or HSCT so as to avoid reactivation during myeloablative chemotherapy. No surgery should be performed in disseminated disease or when a curative resection is not feasible.

### Outcome/prognosis

Early diagnosis and aggressive treatment improve long-term survival of the patients [52]. Immune reconstitution is the best predictor of survival. Isolated pulmonary aspergillosis is associated with better outcome when compared to disseminated disease. Patients who are amenable to complete surgical excision [52, 53] and showed favourable response to antifungal therapy have a better chance of survival [54]. Neutropenia, extra-pulmonic extension of pulmonary aspergillosis, allogeneic BMT [55] and progression or recurrence of underlying haematologic malignancy or fungal relapse predict a worse outcome [52].

**Extremities**

Paediatric oncology patients can develop extremity ischaemia, venous thrombosis and severe necrotising skin and soft tissue infections. Some severe lesions occur due to chemotherapy extravasation.

Deep venous thrombosis is usually related to impaired venous return, such as pelvic tumours, or as paraneoplastic phenomena. It is particularly important to look for clinical signs in unconscious patients. Anticoagulation and preventive measures to avoid pulmonary embolism are indicated.

Necrotising infections should be treated aggressively with antibiotics and surgery. Abscesses should be drained.

## Figures and Tables

**Figure 1.** Decision-making plan – Management of NC [29].

**Table 1.** Agents associated with NC [5–7, 9] (G-CSF, Granulocyte-colonystimulating factor; HIV, Human immunodeficiency virus; HHV6, Human herpes virus 6).

Chemo-therapeutic agents	Non-chemotherapeutic agents	Biologic agents	Other causes
Topotecan	Metamizole	Influenza	Myelodysplastic syndromes
G-CSF	Clozapine	HIV	Alcohol use disorder
Idarubicin	Sulfasalazine	Mucormycosis	Vitamin B12 deficiency
Cytarabine	Thiamazole	Hepatitis A, B, C	Rheumatoid disorders
Vincristine	Carbimazole	Epstein–Barr Virus	Hypersplenism
Daunorubicin	Amoxicillin	COVID-19	Autoimmune
Mercaptopurine	Benzylpenicillin	Cytomegalovirus (CMV)	Neonatal alloimmune neutropenia
L-asparginase	Cefepime	HHV6	
Methotrexate	Linezolid	Malaria	
Etoposide	Meropenem	Lyme disease	
Amsacrine	Valganciclovir		
Thioguanine			
Cyclophosphamide			
Mitoxatrone			
Alemtuzumab			

## References

[1] NIH (2013). SEER Cancer Statistics Review, 1975–2010.

[2] Prusakowski MK, Cannone D (2014). Pediatric oncologic emergencies. Emerg Med Clin North Am.

[3] Chui CH (2012). Surgical management of complications of multimodal therapy. Pediatr Blood Cancer.

[4] Lieber J, Hauch H, Lang P (2012). Surgical management of stem cell transplantation-related complications in children. Pediatr Transplant.

[5] McCarville MB (2006). Evaluation of typhlitis in children: CT versus US. Pediatr Radiol.

[6] Bremer CT, Monahan BP (2006). Necrotizing enterocolitis in neutropenia and chemotherapy: a clinical update and old lessons relearned. Curr Gastroenterol Rep.

[7] Katz JA, Wagner ML, Gresik MV (1990). Typhlitis. An 18-year experience and postmortem review. Cancer.

[8] Urbach DR, Rotstein OD (1999). Typhlitis. Can J Surg.

[9] McCarville MB, Adelman CS, Li C (2005). Typhlitis in childhood cancer. Cancer.

[10] Moran H, Yaniv I, Ashkenazi S (2009). Risk factors for typhlitis in pediatric patients with cancer. J Pediatr Hematol Oncol.

[11] Xia R, Zhang X (2019). Neutropenic enterocolitis: a clinico-pathological review. World J Gastrointest Pathophysiol.

[12] Pelletier JH, Nagaraj S, Gbadegesin R (2017). Neutropenic enterocolitis (typhlitis) in a pediatric renal transplant patient. A case report and review of the literature. Pediatr Transplant.

[13] Altınel E, Yarali N, Isık P (2012). Typhlitis in acute childhood leukemia. Med Princ Pract.

[14] Gomez L, Martino R, Rolston KV (1998). Neutropenic enterocolitis: spectrum of the disease and comparison of definite and possible cases. Clin Infect Dis.

[15] Sloas MM, Flynn PM, Kaste SC (1993). Typhlitis in children with cancer: a 30-year experience. Clin Infect Dis.

[16] Mullassery D, Bader A, Battersby AJ (2009). Diagnosis, incidence, and outcomes of suspected typhlitis in oncology patients--experience in a tertiary pediatric surgical center in the United Kingdom. J Pediatr Surg.

[17] Yi HS, Sym SJ, Park J (2010). Typhlitis due to mucormycosis after chemotherapy in a patient with acute myeloid leukemia. Leuk Res.

[18] Roca B, Fernandez P, Roca M (2018). Typhlitis as a complication of influenza in a patient with advanced HIV infection. Postgrad Med.

[19] Shafey A, Ethier MC, Traubici J (2013). Incidence, risk factors, and outcomes of enteritis, typhlitis, and colitis in children with acute leukemia. J Pediatr Hematol Oncol.

[20] Shamberger RC, Weinstein HJ, Delorey MJ (1986). The medical and surgical management of typhlitis in children with acute nonlymphocytic (myelogenous) leukemia. Cancer.

[21] Davila ML (2006). Neutropenic enterocolitis. Curr Opin Gastroenterol.

[22] Goldberg SR, Godder K, Lanning DA (2007). Successful treatment of a bowel perforation after chemotherapy for Burkitt lymphoma. J Pediatr Surg.

[23] Kirkpatrick ID, Greenberg HM (2003). Gastrointestinal complications in the neutropenic patient: characterization and differentiation with abdominal CT. Radiology.

[24] Cartoni C, Dragoni F, Micozzi A (2001). Neutropenic enterocolitis in patients with acute leukemia: prognostic significance of bowel wall thickening detected by ultrasonography. J Clin Oncol.

[25] Lehrnbecher T, Robinson P, Fisher B (2017). Guideline for the management of fever and neutropenia in children with cancer and hematopoietic stem-cell transplantation recipients: 2017 update. J Clin Oncol.

[26] Gorschlüter M, Mey U, Strehl J (2006). Invasive fungal infections in neutropenic enterocolitis: a systematic analysis of pathogens, incidence, treatment and mortality in adult patients. BMC Infect Dis.

[27] De Caen AR, Berg MD, Chameides L (2015). Part 12: pediatric advanced life support: 2015 American Heart Association Guidelines Update for Cardiopulmonary Resuscitation and Emergency Cardiovascular Care. Circulation.

[28] Sundell N, Boström H, Edenholm M (2012). Management of neutropenic enterocolitis in children with cancer. Acta Paediatr.

[29] Alvares HPG (2020). Decision Making on the Management of Neutropenic Colitis.

[30] Hohenberger P, Buchheidt D (2005). Surgical interventions in patients with hematologic malignancies. Crit Rev Oncol Hematol.

[31] Skibber JM, Matter GJ, Pizzo PA (1987). Right lower quadrant pain in young patients with leukemia. A surgical perspective. Ann Surg.

[32] Ettinghausen SE (1993). Collagenous colitis, eosinophilic colitis, and neutropenic colitis. Surg Clin North Am.

[33] Koea JB, Shaw JH (1989). Surgical management of neutropenic enterocolitis. Br J Surg.

[34] Cunningham SC, Fakhry K, Bass BL (2005). Neutropenic enterocolitis in adults: case series and review of the literature. Dig Dis Sci.

[35] Machado NO (2010). Neutropenic enterocolitis: a continuing medical and surgical challenge. N Am J Med Sci.

[36] Moir CR, Scudamore CH, Benny WB (1986). Typhlitis: selective surgical management. Am J Surg.

[37] Wade DS, Nava HR, Douglass HO (1992). Neutropenic enterocolitis. Clinical diagnosis and treatment. Cancer.

[38] Reilly PM, Wilkins KB, Fuh KC (2001). The mesenteric hemodynamic response to circulatory shock: an overview. Shock.

[39] Wilson DB, Rao A, Hulbert M (2004). Neutropenic enterocolitis as a presenting complication of acute lymphoblastic leukemia: an unusual case marked by delayed perforation of the descending colon. J Pediatr Surg.

[40] Saillard C, Zafrani L, Darmon M (2018). The prognostic impact of abdominal surgery in cancer patients with neutropenic enterocolitis: a systematic review and meta-analysis, on behalf the Groupe de Recherche en Reanimation Respiratoire du patient d’Onco-Hematologie (GRRR-OH). Ann Intensive Care.

[41] Knoderer HM, Robarge J, Flockhart DA (2007). Predicting asparaginase-associated pancreatitis. Pediatr Blood Cancer.

[42] Kearney SL, Dahlberg SE, Levy DE (2009). Clinical course and outcome in children with acute lymphoblastic leukemia and asparaginase-associated pancreatitis. Pediatr Blood Cancer.

[43] Spraker HL, Spyridis GP, Pui CH (2009). Conservative management of pancreatic pseudocysts in children with acute lymphoblastic leukemia. J Pediatr Hematol Oncol.

[44] Palasciano G, Portincasa P, Vinciguerra V (1989). Gallstone prevalence and gallbladder volume in children and adolescents: an epidemiological ultrasonographic survey and relationship to body mass index. Am J Gastroenterol.

[45] Rescorla FJ (1997). Cholelithiasis, cholecystitis, and common bile duct stones. Curr Opin Pediatr.

[46] Mahmoud H, Schell M, Pui CH (1991). Cholelithiasis after treatment for childhood cancer. Cancer.

[47] Holcomb Jr GW, Holcomb III GW (1990). Cholelithiasis in infants, children, and adolescents. Pediatr Rev.

[48] Safford SD, Safford KM, Martin P (2001). Management of cholelithiasis in pediatric patients who undergo bone marrow transplantation. J Pediatr Surg.

[49] Decker DB, Karam JA, Wilcox DT (2009). Pediatric hemorrhagic cystitis. J Pediatr Urol.

[50] Rubio PM, Sevilla J, González-Vicent M (2009). Increasing incidence of invasive aspergillosis in pediatric hematology oncology patients over the last decade: a retrospective single centre study. J Pediatr Hematol Oncol.

[51] Lin SJ, Schranz J, Teutsch SM (2001). Aspergillosis case-fatality rate: systematic review of the literature. Clin Infect Dis.

[52] Cesaro S, Cecchetto G, De Corti F (2007). Results of a multicenter retrospective study of a combined medical and surgical approach to pulmonary aspergillosis in pediatric neutropenic patients. Pediatr Blood Cancer.

[53] Gow KW, Hayes-Jordan AA, Billups CA (2003). Benefit of surgical resection of invasive pulmonary aspergillosis in pediatric patients undergoing treatment for malignancies and immunodeficiency syndromes. J Pediatr Surg.

[54] Cesaro S, Giacchino M, Locatelli F (2007). Safety and efficacy of a caspofungin-based combination therapy for treatment of proven or probable aspergillosis in pediatric hematological patients. BMC Infect Dis.

[55] Salerno CT, Ouyang DW, Pederson TS (1998). Surgical therapy for pulmonary aspergillosis in immunocompromised patients. Ann Thorac Surg.

# Discussion: Emergencies related to tumour bulk

**Published:** 2022

**Authors:** Ahmed Elgendy, Sharon Cox, Paul D Losty, Theodoros Dionysis, Ibiyeye Taiye, Jan Godzinski, Jaime Shalkow

## Bowel Obstruction

(Please also refer to *Surgery and Lymphoma guideline*: page 204)

### Epidemiology

Common intra-abdominal malignancies in children are Wilms tumour, neuroblastoma, Non-Hodgkin’s lymphoma and rhabdomyosarcoma, presacral teratoma [1, 2]. The bowel related emergencies in such patients with abdominal tumours include intussusception, intestinal obstruction, ischaemia and perforation [3]. Intestinal obstruction can occur in patients with primary abdominopelvic tumours and also from metastasis seeding to the abdomen from other solid tumours [4]. Chemotherapy and radiotherapy may also cause bowel obstruction or perforation [1].

Intestinal obstruction in patients with abdominal malignancy can be complete or partial. Obstruction could be mechanically related from extrinsic compression of the bowel by a solid tumour or peritoneal deposits, malignant adhesions, infiltration of the bowel wall, intraluminal growth of tumour and malignant strictures [4, 5]. Obstruction can also occur from paralytic ileus or dysmotility as a result of electrolyte imbalance (e.g. low serum potassium), sepsis or from infiltration of the enteric nerves and bowel wall muscle, use of narcotic agents and cytotoxic drugs like vinca-alkaloids [2, 5].

### Preoperative evaluation

Clinical presentation may be of a child either known with a malignancy, or as a first presentation of an advanced intra-abdominal malignancy. There may be a history of a progressively enlarging abdominal mass, colicky pains, progressive abdominal distention and fever, bilious or feculent vomiting and associated constipation. The patients may have been on chemotherapy or narcotics.

Physical examination may reveal a child with obvious signs of malignancy such as weight loss, cachexia and jaundice. The child may be dehydrated and febrile. The abdomen will be distended, with or without a palpable abdominal mass. Bowel sounds may be hyperactive in mechanical obstruction but hypoactive or absent in adynamic obstruction. The rectum may be empty or contain some stool.

Relevant investigations will include complete blood count showing leukocytosis with elevated neutrophil count in sepsis or leukopenia with or without neutropenia in patients who are on chemotherapy. Serum electrolytes may be deranged, and creatinine and urea may be raised with dehydration. There may be elevated serum bilirubin on liver function tests.

### Images

- Plain abdominal X-ray: this may show dilated bowel loops, multiple air fluid levels, paucity of gas in the distal bowel and/or a soft tissue shadow with displacement of bowel loops in solid intra-abdominal tumours.
- Abdominal CT scan imaging: CT scan with oral contrast may confirm the presence, site and degree of bowel obstruction. It will also confirm the presence and organ of origin of a tumour and degree of involvement of contiguous structures.

### Biopsy

Biopsy should be obtained during laparotomy. Radiology guided ultrasound needle biopsy can also be considered in equipped specialist centres.

### Indications for surgery

Surgery is indicated in patients with complete mechanical intestinal obstruction and in those with associated complications such as bowel perforation.

Patients with incomplete bowel obstruction may require recourse to non-operative management; this will involve placing the patient on nil by mouth, with NG tube drainage, delivery of intravenous fluids with hyoscine hydrobromide or glycopyrolate to reduce episodes of colic, and occasionally octreotide to reduce intestinal secretion(s) [5]. Antiemetic agents may also be administered [5]. Chemotherapy that rapidly reduces tumour bulk may be helpful – depending on the nature of the tumour. Non-resolving or recurrent obstruction may necessitate laparotomy.

### Surgical goals

The primary goal of surgery is to ensure adequate bowel decompression. The emphasis is on non-mutilating surgery. Primary resection of the tumour is a secondary goal if feasible. If resection is not feasible, bowel diversion via enterostomy stoma and tumour biopsy is advised, specifically in the emergency situation where the diagnosis is unknown, as chemotherapy may decrease tumour bulk and avoid extensive surgery.

## Bowel Gangrene and Perforation

### Epidemiology

Bowel gangrene in abdominal malignancy may result from intussusception, malignant intestinal bands including adhesions and volvulus. Bowel perforation can result from infiltration of the bowel wall by tumours, bowel ischaemia secondary to vascular thrombosis or insufficiency and use of medications that predispose to frank ulcer formation such as prednisolone [6].

Bowel gangrene and perforation present with fulminant peritonitis which can progress to sepsis, and septic shock. Bowel gangrene and perforation in an already immune compromised patient worsens the prognosis of any abdominopelvic tumour.

### Preoperative evaluation

Clinical presentation is that of a child with abdominal tumour with sudden onset or progressively worsening abdominal pain. There may be associated progressive abdominal distension, vomiting, passage of bloody stool and fever.

Examination may reveal an acutely ill child, who is pale, febrile and dehydrated. The child may be in hypovolaemic or septic shock, and have a fast and thready low volume pulse with slow capillary refill. Low blood pressure is an ominous late sign. The abdomen may be distended, with generalised abdominal tenderness, rebound tenderness and rigidity.

Relevant investigations will include complete blood count which may show elevated neutrophils and an electrolyte profile with varied anomalies and elevated serum creatinine and urea. There may be elevated serum bilirubin on liver function tests. Serum lactate may be elevated in bowel ischaemia.

### Images

Plain abdominal X-ray may show free intra-abdominal air.

CT scan may have findings of increased bowel wall thickness and pneumatosis intestinalis [7, 8]. Free air in the peritoneum, and extra luminal oral contrast and gas within the tumour are features of bowel perforation on CT scan [3, 9]. It may also show the presence of the tumour, the organ of origin and involvement of contiguous structures.

### Biopsy and Indications for surgery

Bowel gangrene and perforation are indications for laparotomy. Biopsy of the tumour should be taken during surgery.

### Surgical goals

Bowel gangrene: complete resection of gangrenous bowel (with preservation of adequate bowel length) and ensuring control of bowel contents either via primary anastomosis (in a stable child with macroscopically normal bowel), or stoma creation if there is any concern relating to an anastomosis. In the case of severe sepsis and haemo-dynamic instability, a damage control approach with a ‘clip and drop’ procedure may be required, with a relook laparotomy when the patient is stable.

Bowel perforation: simple debridement and closure of the perforation in macroscopically normal bowel if feasible or resection with primary anastomosis will be determined by the site and number of perforations. Thorough irrigation of the peritoneum should be done to control peritoneal sepsis.

Tumour debulking if feasible – with the emphasis on non-mutilating surgery, should only be performed if the patient is stable enough to withstand the surgery, otherwise biopsy of the tumour should be taken.

### Perioperative management of patients with complete bowel obstruction, gangrene and perforation

The patient should be resuscitated, this will involve:

- An intra-venous access and correction of fluid deficit using isotonic fluid such as normal saline or Ringer’s lactate.
- Passage of an appropriately sized NG tube to decompress the stomach and urethral catheter to monitor urine output.
- Replacement of ongoing fluid loss from the NG tube with equivalent volume of isotonic fluid.
- Commencement of maintenance fluid based on the child’s weight should be commenced once dehydration has been corrected and urine output is optimal.
- Administration of broad-spectrum intravenous antibiotics.
- Correction of anaemia and electrolyte imbalance.

Monitoring during the pre-operative period should include vital signs, urine output and serum electrolytes.

### Special needs:

Central venous line, invasive monitoring (such as central venous pressure, arterial blood gases) and total parenteral nutrition may be required. Patients with evidence of septic shock will require vasopressors.

### Surgical approach

Exploratory laparotomy should be performed through a midline or transverse abdominal incision depending on the age of the child. During exploration, the tumour should be identified, involvement of contiguous structures and presence or absence of peritoneal and hepatic metastasis should be determined.

- For complete bowel obstruction, the site of bowel obstruction, feasibility of tumour resection, the need for bowel resection with primary anastomosis or creation of a stoma should be determined.
- For bowel gangrene or perforation: Control of spillage of bowel contents followed by assessment of the extent of the gangrene and decisions on the length of bowel to be resected.

Following bowel resection, a stoma should be fashioned if primary anastomosis is not feasible. Biopsy of the tumour should be obtained at surgery if tumour resection is also not feasible. When considering primary anastomosis, the surgeon needs to remember that chemotherapy may be initiated soon after surgery, possibly with high doses of steroids, and it may be safer to create a stoma and restore bowel continuity after discontinuation of chemotherapy, to avoid the risk of anastomotic dehiscence, sepsis and death. If the patient has a very localised tumour, resection with primary anastomosis are viable options.

### Complications

- Intraoperative complications include haemorrhage, tumour rupture, injuries to surrounding structures and anaesthetic complications.
- Early complications include wound infection or dehiscence, anastomotic leakage and intra-abdominal abscess.
- Late complications include adhesive intestinal obstruction and tumour relapse.

### Postoperative considerations

- The primary tumour: completely resected or presence of residual tumours during emergent laparotomy.
- Post-operative chemotherapy and radiotherapy.
- Post-operative complications.
- Stoma function.

### Tips

- The intervention that will provide the best results for the patient at the time of presentation should be opted for.
- In case of bowel resection, primary anastomosis should be performed if distal obstruction is ruled out, the child is in a good physiological state, and the bowel to be anastomosed is not involved in the inflammatory or malignant process. In all other situations, a stoma is the safest option – bearing in mind that they do have their own complications [10].
- In the case of an unstable child, a damage control approach with ‘clip and drop’ procedure and a subsequent relook is a good option.
- Conserve as much bowel length as possible so as to reduce the risk of short bowel syndrome.
- In case a stoma needs to be created; consider a divided stoma.
- In non-resectable tumours, consider stoma creation and tumour biopsy instead of tumour debulking; this will reduce the risk of injury to contiguous structures.

### Pitfalls

- Widespread infiltration of the bowel wall may make primary anastomosis and stoma creation not feasible.
- High risk of internal fistula and enterocutaneous fistula during laparotomy in patients with malignant adhesions.

## Intussusception

(Please also refer to *Surgery and Lymphoma guideline*: page 204)

### Epidemiology:

Intussusception can be caused by benign small bowel tumours (polyps, haemangioma/vascular malformations, neurofibroma, fibroma, leiomyoma, GI stromal tumour, lipoma and lipoblastoma) or malignant tumours (Burkitt lymphoma and carcinoid tumour) [11]. Intussusception is the initial presentation in 17%–25% patients with abdominal Burkitt lymphoma [12, 13]. Physicians should strongly suspect underlying pathology when older children (aged > 6 years) present with intussusception [14].

Postoperative intussusception is an uncommon, but important complication, and must be suspected in every child who shows symptoms of postoperative intestinal obstruction occurring within a week following an abdominal (and more especially retroperitoneal) operation. Since the typical features of intussusception are usually absent and radiology frequently unhelpful (except for ultrasound), a high index of clinical suspicion is necessary for early diagnosis and treatment, to avoid intestinal necrosis. (Please refer to *Wilms tumour guideline*: page 57)

### Preoperative evaluation, images, special needs, biopsy and Indications for surgery:

The classic presentation is an infant or a young child with intermittent, crampy abdominal pain associated with ‘red-currant jelly’ stools and palpable mass on physical examination, although this triad is seen in less than a fourth of children. Vomiting is seen in 80% of the cases and is an early symptom [15].

Abdominal radiographs may show evidence of obstruction. US and CT (if performed) show bowel within bowel (‘target/doughnut sign’, ‘pseudo-kidney sign’ or ‘concentric ring sign’ on US) [16]. Of note, most small bowel intussusceptions found in children with US or CT are transient and of no clinical significance [17]. Findings that suggest transient small bowel intussusception on US include: small size without wall swelling; short segment; preserved wall motion; absence of the lead point [17]. Repeat focused US can document spontaneous resolution when necessary.

Findings that suggest pathological intussusception include intussusception length > 3.5 cm, and evidence of bowel obstruction or an identifiable lead point [18, 19].

The indication for surgery, especially for patients with possible Burkitt lymphoma, is the ‘acute abdomen’ with perforation of the bowel, an older child with intussusception and an identifiable lead point on ultrasound.

### Surgical goals:

In the case of intussusception related to a lead point, the aim is total resection of the tumour. In the case of postoperative intussusception, enema reduction can be attempted if at the ileocaecal area but as many of these intussusceptions are present in the small bowel territory, operative intervention is often needed. Reduction without resection and anastomosis is preferable, and can be performed if necrosis has not yet been established.

### Perioperative management:

Routine perioperative fluid resuscitation and correction of and haematological, electrolyte or hydration abnormalities needs to be performed before embarking on surgical procedures [20].

Antibiotics may be indicated, specifically in a patient with a potentially ischaemic intussusceptum, or in patients with peritonitis.

Oncology patients with a postoperative intussusception are normally in an altered physiological state due to effects of chemo and/or radiotherapy. These treatments target rapidly proliferating neoplastic tissues and the GI mucosa is also particularly vulnerable. In addition, chemotherapeutic agents cause nausea and vomiting, and may result in diarrhoea, mucositis, stomatitisand neutropenic enterocolitis. These adverse effects may all exacerbate malnutrition and dehydration. Oncology patients may have chronic nausea and vomiting or delayed gastric emptying exacerbated by opioids. These children may be at an increased risk for aspiration on anaesthetic induction and should be managed accordingly.

### Surgical approach:

In a patient with a suspected lead point intussusception, the approach can be via laparoscopy, be laparoscopic assisted or open. In most cases of a malignancy presenting as intussusception, the mass is easily resectable and primary anastomosis is possible. If the child is physiologically unstable or there is extensive soiling of the peritoneal cavity, or in the event of an unresectable mass, bowel content diversion by means of enterostomy is preferred. Biopsy of the tumour is then performed.

Post-operative intussusception should be approached in a similar fashion as discussed above.

### Complications:

Postoperative complications are not uncommon in this vulnerable patient group. Because of immunosuppression, preoperative chemotherapy and radiotherapy, wound infections or poor wound healing can occur.

### Postoperative considerations:

Routine observations related to electrolytes, hydration status and pain control are to be applied.

Other postoperative considerations are liver function and pulmonary status, especially in patients with disseminated disease. Post-operative monitoring should incorporate assessment of all these issues.

### Tips:

- Early recognition and management is the key for early treatment.
- Avoid mutilating procedures.
- Meticulous monitoring of the patient is important for a smooth postoperative period.

### Pitfalls:

- Intussusception in the case of a lead point should not be reduced via enema techniques.
- Vomiting and abdominal distention in retroperitoneal oncology procedures can be caused by intussusception.
- Resect and perform primary anastomosis in tumour related intussusception.
- Avoid a stoma if anastomosis is possible.

## Ovarian Tumour Torsion

(Please refer to *GCT guideline*: page 103)

### Epidemiology:

The annual incidence of ovarian neoplasms is estimated at 2.6 cases per 100,000 females and they are very rarely malignant, representing only 1% of all cancers in children and adolescents [21–23]. Ovarian tumours are one of the more common germ cell tumours in the female patient. Of all ovarian masses, most (80%) are benign (epithelial cysts, teratomas, immature teratomas) often with predominantly cystic components.

### Preoperative evaluation, images, special needs, biopsy and indications for surgery:

Preoperative evaluations with imaging are the following:

- Abdominal ultrasonography [24] with a full bladder indicates the nature of the mass (cystic or solid), its size, borders, location and relations and any possible repercussions on the upper urinary tract [25]. Doppler ultrasonography may help to identify the structure of a mass [26]. Certain signs on imaging may be useful in evaluating the probable benign or malignant nature of an ovarian mass lesion. These signs have a varying predictive value, which is increased when several elements are found together (poorly defined borders, a thick irregular wall, thick rigid septations, mainly solid component, size, local spread, etc.). The contribution of ultrasonography in paediatric practice is restricted by the limitation of endovaginal sonography in this young patient cohort.
- Sectional Imaging: CT or magnetic resonance imaging (MRI) helps to locate the mass and identify its nature. MRI is particularly valuable for characterising the various fluid and tissue structures [27–29].

Some ovarian neoplasms secrete protein or hormone biomarkers that can be assayed in either peripheral blood samples or the ovarian cyst fluid [30]. These substances are not specific markers for ovarian origin but are specific of tissue function, and are more useful for follow-up surveillance than initial primary diagnosis [31]. The main markers are carcinoembryonic antigen in epithelial and germ cell tumours; alpha-fetoprotein, which is produced by mixed germ cell tumours and immature teratomas, and human chorionic gonadotropin, which is elevated in choriocarcinoma and embryonic ovarian carcinomas [30, 32]. Cancer antigen 125 (CA 125) is a sensitive but not specific marker for non-mucinous epithelial ovarian cancer. Elevated serum CA 125 is typically associated with ovarian malignancies, but it can also be found in association with other intraperitoneal processes [33, 34]. Elevated serum inhibin B was reported in granulosa cell tumours [35] and in some mucinous carcinomas. Mullerian inhibiting substance is found elevated in females with ovarian sex cord tumours [36].

The only indication for emergency surgery with ovarian tumour pathology is clinical deterioration in the setting of an acute abdominal crisis.

### Surgical goals:

The surgical goals for the paediatric and adolescent population, which suffers from an acute abdomen due to ovary tumour torsion, are to minimise u spillage and preserve reproductive function.

### Perioperative management:

As per all emergency situations, stabilisation of the patient is the cornerstone of therapy. Abdominal tenderness and a history of ‘rapid-ischemic’ pain with vomiting in a female patient, strongly indicates likelihood of ovary torsion. Laboratory evaluation with biochemistry and serum biomarkers as well as cross match blood should be performed. Emergency pelvic ultrasound is an adjunct to timely diagnosis.

### Surgical approach

Many adolescent female patients present with primarily cystic lesions and most of these are benign. Laparoscopy has been widely utilised. The main controversy surrounds the ability of the surgeon to perform a cancer type procedure in cases where the exact tumour histology (benign versus malignant) cannot be determined accurately preoperatively. If the lesion is primarily solid or if the serum biomarkers are elevated, an open procedure is indicated. If the serum markers are normal and the lesion is primarily cystic, particularly if there is a very large cystic component, a less invasive technique may be considered; however, avoidance of tumour spill must be assured. One minimal access procedure involves laparoscopic excision of the tumour from its attachments, placement in a retrieval bag and delivering the neck of the bag outside of the abdominal cavity through the umbilical opening. The cyst is then punctured, the fluid removed and the cystic lesion, contained within the bag removed without spillage and sent for pathology examination. In the second technique, useful for giant ovarian cysts, the cyst is exposed through an approximate 5-cm incision, a bag glued to the cyst with cyanoacrylate adhesive as described by Shozu *et al* [37]. The cyst is then incised by cutting through the centre of the bag-cyst interface, the fluid removed without spillage and the decompressed cyst removed from the abdominal cavity. The remainder of the operative procedure notably peritoneal/ascitic fluid sampling, omental inspection and excision and evaluation of the peritoneal surface can be performed laparoscopically.

There is another strategy regarding the timing of tumour resection in some patients with normal markers and without suspicion of malignancy during imaging evaluation. Ovarian detorsion can be considered initially through classical open approach or minimally invasive surgery, and tumour resection can be performed after 7–10 days from the first procedure. The postponement of definitive surgery gives time for oedema and tissue congestion to subside and can facilitate ovarian-sparing surgery and gonadal tissue preservation [38].

### Complications:

Tumour spillage or unnecessary oophorectomy are considered major complications.

### Postoperative considerations:

Routine post-operative observations are required. In the case of elevated tumour biomarkers, these can be assayed some 5 days postoperatively. Pain management is also an important postoperative consideration.

### Tips:

- Always evaluate imaging with an experienced Paediatric Radiologist.
- Prefer open ‘classical’ approaches with giant ovarian cystic lesions.

### Pitfalls:

- Avoid tumour spillage.
- Don’t assume a cystic lesion is benign.
- Adult ovarian tumours have different biology from paediatric ovary tumours. A paediatric gynaecologist or paediatric oncology surgeon is the most suitable health care professional to treat such tumours.

## Rupture of Renal and Liver Tumours as Emergencies

(Please refer to *hepatoblastoma and HCC and Wilms tumour guidelines*: page 94)

One of the classical emergencies in paediatric oncology is the rupture of a solid tumour. Rupture has important consequences for both the immediate status of the child and the oncological outcome. This issue was best explored in renal tumours of childhood.

### Ruptures:

Rupture may occur before any treatment, before surgery or intraoperatively. Pre-treatment tumour ruptures are usually an emergent clinical presentation with associated internal haemorrhaging that may be life threatening.

Immediate surgery may be required if the patient cannot be stabilised haemodynamically. If the rupture is limited or retroperitoneal, conservative management may be successful (Figure 2). Diagnostic work-up, preoperative chemotherapy and planned elective surgery may all occur according to protocol. If at the post-chemotherapy elective scheduled operation neither surgeon nor pathologist is able to find any traces of the pre-treatment rupture, there is no mandatory obligation to upstage the patient to stage III disease and limit radiotherapy.

Pre-surgical ruptures in patients already on treatment are possible, but less likely. These patients have a known disease burden, care should be taken to avoid further trauma where possible.

According to multicentre nephroblastoma studies, intra-operative ruptures occur more frequently in the primarily operated patients undergoing resection (e.g. NWTS/Children’s Oncology Group and UK studies) as opposed to those cases where neoadjuvant chemotherapy is first initiated (e.g. International Society of Paediatric Oncology Renal Tumor Study Group studies) [39–43]. Improvement in diagnostic imaging and surgical techniques have resulted in decreased rupture rates in both subgroups, however, even in major centres which recommend primary surgery, the particularly large and technically difficult cases may be subjected to preoperative chemotherapy. To decrease intraoperative rupture rate(s), it is important to have good exposure, handle the tumour mass with extreme care during resection and consider ligation of renal vessels (artery sometime first then vein) prior to mobilisation, leading to ‘vascular exclusion’ theoretically decreasing intralesional pressure so as to decrease the fragility of the tumour mass. Early access to renal vessels is more difficult in massive tumours which cross the midline with vessel anatomy distortion [44]. These cases may require tumour mobilisation first, with later visualisation and ligation of the feeding vessels.

In the case of intraoperative rupture or emergency nephrectomy is mandated due to rupture, the local stage is graded III and radiotherapy is mandatory. Outcomes in stage 3 post-rupture patients are still satisfactory; however, low-dose radiotherapy may have some adverse late effects for the child [40, 44].

Ruptures of liver tumours occur less frequently however similar care strategies may apply. Thus, the approach at rupture may depend on the local expertise of the oncology service and current ‘PRETEXT’ grouping. Stabilisation of the patient, initiation of local therapy protocols and consideration of surgery should be carefully planned. If it is impossible to control refractory haemorrhage operation with ‘damage control’, plans should proceed. Small and favourably located tumour lesions can be resected respecting oncological rules and adequate clear margins. In case of huge tumours with high ‘PRETEXT’ when anatomical or non-anatomical resection of the tumour is impossible, the rupture should be folded closed ‘like a book’ and a tumour biopsy sample taken. From a technical point of view, Pringle’s manoeuvre may decrease bleeding and make surgery easier. In extreme cases, cardio-pulmonary by-pass or even extracorporeal circulation may be helpful. A pivotal role for radiology guided interventional endovascular procedures, particularly hepatic artery embolisation techniques, is expanding with interesting data published available [45–48].

## Haemoperitoneum

Malignant tumours may produce haemoperitoneum due to spontaneous rupture, blunt or surgical trauma, or chemotherapy-induced necrosis. Patients present with abdominal mass or distension, compartment syndrome, shock and low haematocrit levels. Haemorrhage control with interventional radiology has been described. At surgery, the tumour should be resected when feasible, providing the patient is stable. Otherwise, damage control surgery is indicated. Blood products should be available in the operating room before starting the procedure, since abdominal decompression usually exacerbates the bleed and makes patients unstable. Most patients require ICU admission after surgery. Malignant haemoperitoneum is a serious complication in paediatric cancer patients, with a dismal prognosis.

## Urinary Tract Obstruction

### Epidemiology

Urinary tract obstruction from tumours can occur as a result of external compression by solid tumours or infiltration of the wall or growth of tumour within the lumen of the urinary tract. Urinary tract obstruction can be seen in about 25% of patients with pelvic and retroperitoneal tumours [3]. Obstruction of the urinary tract can involve the upper tracts (renal pelvis and ureters) and the lower urinary tract (bladder and urethra).

The tumours found to cause hydronephrosis in children include neuroblastoma, immature teratoma, rhabdomyosarcoma, yolk sac tumour, ganglioneuroma, dysgerminoma, endodermal sinus tumour, desmoplastic small round cell tumour, appendix adenocarcinoma and Ewing’s sarcoma [49]. (Please refer to the respective guidelines: add citation/ page number) Rhabdomyosarcoma is the commonest bladder tumour in children under 10 years of age. It accounts for some 5% of solid tumours in children and is usually located around the trigone area and bladder neck thereby causing ureteric and bladder neck outlet obstruction [50].

Urinary tract obstruction can result in profound electrolyte imbalance, urinary tract infection and renal failure [51]. These complications can significantly worsen prognosis in children with abdominopelvic tumours.

### Preoperative evaluation

Patients with malignant obstruction of the upper urinary tract may present with pain, anorexia, nausea, vomiting and haematuria in a background of abdominal or pelvic tumour. Patients with bladder outlet obstruction will classically present with history of a lower abdominal mass and pain, with a poor urinary stream.

Physical examination will reveal a patient with an obvious abdominal or pelvic mass with or without obvious signs of metastasis. There may be features of renal failure such as facial oedema, bilateral ankle swelling, elevated blood pressure and abdominal ascites. Presence of fever and tachycardia will likely suggest urosepsis.

Investigations should include serum electrolyte(s), urea and creatinine which may reveal metabolic acidosis, hyperkalaemia, elevated serum urea and creatinine. Complete blood count may show leukocytosis, relative neutrophilia, presence of toxic granulations and immature white blood cells.

Urine microscopy, culture and sensitivity may confirm the presence of a bacterial infection, the offending organism and the antibiotics the organism is likely sensitive to.

### Images

Hydrocalycosis, hydronephrosis and retroperitoneal or pelvic masses will be seen on abdominal ultrasound imaging [3]. CT scan will show uptake and excretion of contrast by the two kidneys, presence of hydrocalycosis and hydronephrosis (unilateral or bilateral) and the level of obstruction of the urinary tract. It will also show the presence of a tumour, primary organ of origin and involvement of contiguous structures [3].

### Biopsy

Trucut biopsy of retroperitoneal tumours can be obtained under ultrasound guidance; biopsy of pelvic tumours may be obtained with laparoscopy while cysto-urethroscopy will aid in the visualisation and biopsy of bladder tumours. Histology of the lesion will provide information on tumour biology, grade and immunohistochemistry which will guide further treatment.

### Indications for surgery

Bladder outlet obstruction from pelvic or bladder tumours in most instances may be relieved with passage of urethral catheter. If this is not possible, a suprapubic catheter will be required. Preoperative chemotherapy where indicated will cause reduction in tumour size and degree of urinary tract obstruction, and possibly allow for removal of the emergently placed catheter. Indication(s) for surgery in this setting are limited to resection of the primary tumour.

For upper tract obstruction, relative indication(s) for operative intervention would include:

- Persistent urosepsis despite antimicrobial therapies
- Worsening renal function
- Persistent hydronephrosis following initial treatment of primary tumour

### Surgical goals

The goals of urologic intervention include relieving urinary tract obstruction to allow free urine drainage, control of sepsis and improvement in renal function.

### Perioperative management

Correct fluid and electrolyte imbalance and commencement of empirical broad spectrum antibiotics with coverage for Gram negative organisms is necessary. Antibiotics should be tailored according to antimicrobial sensitivity(s).

Chemotherapy should be commenced in patients with large tumours causing urinary tract obstruction; this permits reduction in tumour bulk and relief of the obstruction. Surgery can then be performed in an elective setting to resect the primary tumour.

### Surgical approach

Percutaneous nephrostomy and placement of ureteric stents can be usefully deployed to relieve malignant ureteric obstruction [3, 51, 52]. The use of metallic stents is well documented in malignant ureteric obstruction in adults; however, there is paucity of information with regard to use in children. Stents are preferably passed into the ureter in a retrograde fashion though the working channel of the cystoscope. Antegrade passage is guided radiologically with ultrasound to establish nephrostomy(s).

Percutaneous nephrostomy is indicated in patients with hydronephrosis with associated urosepsis or worsening renal function. It is performed under image guidance. The procedure can be performed using local or general anaesthesia depending on the age and the clinical state of the patient. The patient should be placed prone, or supine in those with compromised cardiorespiratory function. After cleaning and draping, local anaesthetic should be infiltrated. A sterile ultrasound probe should be used in locating the posterior renal calyx below the 12th rib. An appropriately sized Chiba needle should then be introduced into the calyx under ultrasound guidance, aspiration of urine will confirm successful placement of the needle. A guidewire is then introduced into the calyx, and the tract should be dilated. An adequate sized nephrostomy tube/catheter should then be passed over the guidewire [54]. Correct placement of the catheter in the calyx is confirmed with injection of contrast through the catheter.

### Complications

Complications of percutaneous nephrostomy include bleeding, infection, blockage and dislodgement of the tube, and leakage [51–53] while complications from the use of stents include stent migration and occlusion [52].

### Postoperative considerations

Considerations in the postoperative period will include:

- Renal function monitoring in terms of patient recovery from uraemia and acute renal failure.
- Recovery from urosepsis.
- Further treatment required for the primary tumour.

### Tips

A high percentage of urinary tract obstruction from tumours will resolve following treatment of the primary tumour (via surgery, chemotherapy or radiotherapy), a small percentage will require further urologic interventions as a result of persistent hydronephrosis, patients in this category usually often have a higher tumour stage, grade and poor prognosis [49].

### Pitfalls

The use of chemotherapy in the management of the primary tumour with the aim of reducing tumour bulk and relieving obstruction in patients with impaired renal function can be challenging. This may require pharmacological reduction in the dose(s) of cytotoxic drugs to be administered in such patients.

## Spinal Cord Compression

Spinal cord compression is a surgical oncological emergency that may lead to long-term or permanent neurological impairment if the diagnosis and/or the management are delayed for even only a few hours [54]. The incidence of such emergency(s) has been reported in up to 5% of all children and adolescents with solid neoplasms. Spinal cord compression can be the first presenting symptom of malignant tumours, or it may occur as a consequence of late metastases, a second malignancy or for example, as an isolated relapse, with neuroblastoma, Ewing sarcoma and rhabdomyosarcoma being the main causes, and certain malignant germ cell tumours (such as yolk sac tumours) to a lesser extent [55].

The intraspinal extension can cause acute neurological symptoms and permanent neurological sequelae with a significant impact on the patient’s daily activities and health. Back pain is often the primary symptom and when any child who has had a solid malignant tumour presents at the emergency department with back pain, spinal cord compression should be considered first until proven otherwise [56, 57]. Motor and sensory disturbances in addition to urinary retention or incontinence represent major clinical findings. In these patients, tender percussion on the vertebra usually exacerbates back pain [58].

Physical examination and neurological assessment are fundamental in the primary survey. Moreover, urgent radiological scans are a perquisite for a prompt, accurate diagnosis. Plain X-rays are diagnostic in only about 30% of all children with spinal cord compression. The modality of choice to localise the cause and site/extent of cord compression is spinal MRI under sedation or general anaesthesia if required [59].

Prompt management is essential especially in those children with dumbbell neuroblastomas that account for about 5%–10% of all neuroblastomas index cases. Neoadjuvant chemotherapy, as per contemporary protocols, can reduce the role of surgical intervention and decrease the serious orthopaedic consequences [60]. Intravenous injection of corticosteroids in a dose range of 1–2 mg/kg is also recommended [55]. Urgent decompression of the spinal cord is imperative if the patient has no neurological improvement within a day and it is usually best performed by a team of neurosurgeons and paediatric surgeons. Other treatment for intraspinal neuroblastoma includes chemotherapy or local radiation therapy with 2,400 cGy [59]. Decompression should not be unduly delayed in order to confirm the diagnosis, as neuronal ischaemia from spinal cord compression usually leads to rapid irreversible loss of function.

There is no consensus regarding the merits and the disadvantages of different management strategies, and therapy algorithms vary between centres. The data in the literature suggest that immediate administration of chemotherapy is the first choice and is a potential valid therapeutic option in the absence of an experienced surgical team [61]. However, the expert management of these uncommon events requires a dedicated interdisciplinary team including paediatric oncologists, paediatric neurologists, paediatric neurosurgeons, paediatric surgeons, paediatric orthopaedic surgeons and paediatric physiotherapists.

Eventually, the outcome is generally linked with the timing of diagnosis and intervention, and favourable results can be achieved in tertiary referral centres that do not need any transfer of these patients.

## Ventilatory Compromise

Ventilation can be impaired by large abdominal masses, especially in neuroblastoma in patients younger than 18 months of age with massive liver infiltration by the tumour (Pepper syndrome), whose ventilation is predominately diaphragmatic. In most cases, chemotherapy and ventilatory support are enough to solve the problem in a few days; however, an open abdomen can be useful in selected patients. One has to bear in mind that this procedure poses a high risk for infection in immunocompromised patients, so it should be avoided if possible.

Anterior mediastinal masses such as teratomas or thymic carcinomas or lymphomas can result in an airway compression in affected children and adolescents. The common clinical presentations include dyspnoea, cough, wheezing, stridor and pleural effusions. Such symptoms increase in the supine position. These patients present with a respiratory compromise. Prompt administration of steroids and oxygen support should be imperative. After the improvement of the general condition, a precise diagnosis is required in order to commence the definitive treatment. Certain manoeuvres such as open biopsy, imaging guided core biopsy and/or pleural aspiration are often performed for a final tissue diagnosis. The less invasive procedures should be conducted first to reduce the potential risks in these critical patients; however, if failed, invasive approaches are mandatory [62].

## Take Home Messages

- Surgeons must practise and have expertise in advanced life support to treat emergencies and complications in paediatric oncology.
- Surgeons must be aware of the characteristics of paediatric oncology diseases and their urgencies to correctly manage patients, without impairing the oncological prognosis.
- Be aware that surgical complications can occur in the postoperative period.
- In neutropenic patients, treatment should be tailored individually. Conservative treatment often is enough for the majority of scenarios, but surgery can be needed.
- In case surgery is indicated, the multidisciplinary team should talk about the best timing to operate the patient and the surgeon should not be afraid to operate, if there is blood products and ICU support available.

## Figures and Tables

**Figure 2.** (a): (Left image) CT scan of a right-sided Wilms tumour with retroperitoneal rupture. (b): (Right image) The same patient after 4 weeks of administration of chemotherapy where practically no trace of ‘limited rupture’ is seen.

## References

[1] Aguayo P, Ho B, Fraser JD (2010). Bowel obstruction after treatment of intra-abdominal tumors. Eur J Pediatr Surg.

[2] Handa A, Nozaki T, Makidono A (2019). Pediatric oncologic emergencies: clinical and imaging review for pediatricians. Pediatr Int.

[3] Katabathina VS, Restrepo CS, Betancourt Cuellar SL (2013). Imaging of oncologic emergencies: what every radiologist should know. Radiographics.

[4] Dolan EA (2011). Malignant bowel obstruction: a review of current treatment strategies. Am J Hosp Palliat Care.

[5] Ripamonti CI, Easson AM, Gerdes H (2008). Management of malignant bowel obstruction. Eur J Cancer.

[6] Prusakowski MK, Cannone D (2017). Pediatric oncologic emergencies. Hematol Oncol Clin North Am.

[7] Dhatt HS, Behr SC, Miracle A (2015). Radiological evaluation of bowel Ischemia. Radiol Clin North Am.

[8] Saini DK, Chaudhary P, Durga CK (2013). Role of multislice computed tomography in evaluation and management of intestinal obstruction. Clin Pract.

[9] Zissin R, Osadchy A, Gayer G (2009). Abdominal CT findings in small bowel perforation. Br J Radiol.

[10] Ferguson HJ, Ferguson CI, Speakman J (2015). Management of intestinal obstruction in advanced malignancy. Ann Med Surg (Lond).

[11] Coley B (2013). Caffey’s Pediatric Diagnostic Imaging.

[12] Gupta H, Davidoff AM, Pui CH (2007). Clinical implications and surgical management of intussusception in pediatric patients with Burkitt lymphoma. J Pediatr Surg.

[13] LaQuaglia MP, Stolar CJ, Krailo M (1992). The role of surgery in abdominal non-Hodgkin’s lymphoma: experience from the Childrens Cancer Study Group. J Pediatr Surg.

[14] Miller R (2015). Intussusception and Bowel Obstruction Symptoms, Diagnosis and Treatment Options.

[15] Biko DM, Anupindi SA, Hernandez A (2009). Childhood Burkitt lymphoma: abdominal and pelvic imaging findings. AJR Am J Roentgenol.

[16] Strouse PJ, DiPietro MA, Saez F (2003). Transient small-bowel intussusception in children on. CT Pediatr Radiol.

[17] Kim, JH (2004). US features of transient small bowel intussusception in pediatric patients. Korean J Radiol.

[18] Munden MM, Bruzzi JF, Coley BD (2007). Sonography of pediatric small-bowel intussusception: differentiating surgical from nonsurgical cases. AJR Am J Roentgenol.

[19] Turkyilmaz Z, Sönmez K, Demirogullari B (2005). Postoperative intussusception in children. Acta Chir Belg.

[20] Pumberger W, Pomberger G, Wiesbauer P (2002). Postoperative intussusception: an overlooked complication in pediatric surgical oncology. Med Pediatr Oncol.

[21] Breen JL, Bonamo JF, Maxson WS (1981). Genital tract tumors in children. Pediatr Clin North Am.

[22] Norris HJ, Jensen RD (1972). Relative frequency of ovarian neoplasms in children and adolescents. Cancer.

[23] Pfeifer SM, Gosman GG (1999). Evaluation of adnexal masses in adolescents. Pediatr Clin North Am.

[24] Elhage AN, Ghossain V, Germanos M (2000). Kystes de l’ovaire: valeurs des explorations paracliniques. Gynecol Obstet.

[25] Audebert A (1999). Kystes annexiels: les limites techniques du traitement coelioscopique. A propos d’une série de 1,154 kystes. Gynecol Obstet.

[26] Scully RE (1977). Ovarian tumors. A review. Am J Pathol.

[27] Berta P, Hawkins JB, Sinclair AH (1990). Genetic evidence equating SRY and the testis-determining factor. Nature.

[28] Guinet C, Ghossain MA, Buy JN (1995). Mature cystic teratomas of the ovary: CT and MR findings. Eur J Radiol.

[29] Serov SS, Sculty RE, Sobin LH (1973). Histological Typing of Ovarian Tumours International Histological Classification of Tumours.

[30] Schwartz PE (1991). Ovarian masses: serologic markers. Clin Obstet Gynecol.

[31] Tuxen MK, Soletormos G, Dombernowsky P (2001). Serum tumour marker CA 125 in monitoring of ovarian cancer during first-line chemotherapy. Br J Cancer.

[32] Perrone T, Steeper TA, Dehner LP (1987). Alpha-fetoprotein localization in pure ovarian teratoma. An immunohistochemical study of 12 cases. Am J Clin Pathol.

[33] Bast RC, Lazarus H, Feeney M (1981). Reactivity of a monoclonal antibody with human ovarian carcinoma. J Clin Invest.

[34] Giordano S, Miglionico L, Pellegrino M (1996). Hydronephrosis associated with elevated serum levels of CA 125 antigen. Report of a case. Minerva Pediatr.

[35] Lappohn RE, Burger HG, Bouma J (1992). Inhibin as a marker for granulosa cell tumor. Acta Obstet Gynecol Scand Suppl.

[36] Gustafson ML, Lee MM, Scully RE (1992). Mullerian inhibiting substance as a marker for ovarian sex-cord tumor. N Engl J Med.

[37] Shozu M, Segawa T, Sumitani H (2001). Leak-proof puncture of ovarian cysts: instant mounting of plastic bag using cyanoacrylate adhesive. Obstet Gynecol.

[38] Karpelowsky JS, Hei ER, Matthews K (2009). Laparoscopic resection of benign ovarian tumours in children with gonadal preservation. Pediatr Surg Int.

[39] Godzinski J, Tournade MF, Dekraker J (1998). Rarity of surgical complications after postchemotherapy nephrectomy for nephroblastoma. Experience of the International Society of Paediatric Oncology-Trial and Study “SIOP-9” International Society of Paediatric Oncology Nephroblastoma Trial and Study Committee. Eur J Pediatr Surg.

[40] Godzinski J, Weirich A, Tournade MF (2001). Primary nephrectomy for emergency: a rare event in the International Society of Paediatric Oncology Nephroblastoma Trial and Study no 9. Eur J Pediatr Surg.

[41] Kaste SC, Dome JS, Babyn PS Wilms tumour: prognostic factors, staging, therapy and late effects. Pediatr Radiol.

[42] Powis M, Messahel B, Hobson R (2013). Surgical complications after immediate nephrectomy versus preoperative chemotherapy in non-metastatic Wilms’ tumour: findings from the 1991–2001 United Kingdom Children’s Cancer Study Group UKW3. Trial J Pediatr Surg.

[43] Ritchey ML, Shamberger RC, Haase G (2001). Surgical complications after primary nephrectomy for Wilms’ tumor: report from the National Wilms’ Tumor Study Group. J Am Coll Surg.

[44] Godzinski J, Graf N, Audry G (2014). Current concepts in surgery for Wilms tumor – the risk and function-adapted strategy. Eur J Pediatr Surg.

[45] Aronson DC, Meyers RL (2016). Malignant tumors of the liver in children. Semin Pediatr Surg.

[46] Lau KY, Wong TP, Wong WW (2003). Emergency embolization of spontaneous ruptured hepatocellular carcinoma: correlation between survival and Child-Pugh classification. Australas Radiol.

[47] Meyers RL, Maibach R, Hiyama E (2017). Risk-stratified staging in paediatric hepatoblastoma: a unified analysis from the Children’s Hepatic tumors International Collaboration. Lancet Oncol.

[48] Nejmeddine A, Bassem A, Salah B (2010). Spontaneous rupture of hepatocellular carcinoma in children. Afr J Paediatr Surg.

[49] Alexander A, Weber B, Lorenzo A (2011). Hydronephrosis in children with abdominal and pelvic neoplasms: outcome and survival analysis of a single center pediatric oncology series. J Urol.

[50] Shelmerdine SC, Lorenzo AJ, Gupta AA (2017). Pearls and Pitfalls in diagnosing pediatric urinary bladder masses. Radiographics.

[51] Kouba E, Wallen EM, Pruthi RS (2008). Management of ureteral obstruction due to advanced malignancy: optimizing therapeutic and palliative outcomes. J Urol.

[52] Tabib C, Nethala D, Kozel Z (2020). Management and treatment options when facing malignant ureteral obstruction. Int J Urol.

[53] Hwang JY, Shin JH, Lee YJ (2018). Percutaneous nephrostomy placement in infants and young children. Diagn Interv Imaging.

[54] Higdon ML, Higdon JA (2006). Treatment of oncologic emergencies. Am Fam Physician.

[55] Lee DM, Poplack DK (2013). Emergencies in Pediatric Cancer Patients.

[56] Lewis DW, Packer RJ, Raney B (1986). Incidence, presentation, and outcome of spinal cord disease in children with systemic cancer. Pediatrics.

[57] Rhingold SL, Pizzo PP (2006). Oncologic emergencies. Principles and Practice of Pediatric Oncology.

[58] Quint DJ (2000). Indications for emergent MRI of the central nervous system. JAMA.

[59] Byrne TN (1992). Spinal cord compression from epidural metastases. N Engl J Med.

[60] Pio L, Blanc T, Saint Denis T Multidisciplinary surgical strategy for dumbbell neuroblastoma: a single-center experience of 32 cases. Pediatr Blood Cancer.

[61] Poretti A, Grotzer MA (2012). Neuroblastoma with spinal cord compression: is there an emergency treatment of choice?. Dev Med Child Neurol.

[62] Perger L, Lee EY, Shamberger RC (2008). Management of children and adolescents with a critical airway due to compression by an anterior mediastinal mass. J Pediatr Surg.

# Discussion: Emergency access procedures

**Published:** 2022

**Authors:** Ahmed Elgendy, Sharon Cox, Paul D Losty, Stefano Avanzini, Florin Filip, Jaime Shalkow

## Tube or Pigtail Thoracostomy

The pleural space may be the site of various emergency conditions in children with malignancies notably here malignant pleural effusions with widespread lung metastases. This space is occupied by pleural fluid, which is generated as a result of hydrostatic and oncotic pressure gradients between the pleural space and plasma. The pleural fluid is drained by lymphatics and capillaries at an estimated rate of 20 mL/hour/haemithorax in an average weight 70-kg person.

Pneumothorax is defined as entry of air into the pleural space, most frequently as a consequence of trauma or local lung abnormalities, and it can result in decreased lung expansion and significant impairment of gas exchange. Massive pneumothorax may lead to mediastinal shift and cardio-pulmonary compromise. Pneumothorax has been identified as a complication before treatment or after initiation of chemotherapy, radiation or targeted biological therapy in patients with lung malignancies [1]. Removal of the air is performed emergently if the patients are clinically unstable, require mechanical ventilation or exhibit signs of tension pneumothorax.

Pleural effusions represent the accumulation of serous fluid in the pleural space. They could be either transudative or exudative based on the composition of the fluid and the aetiology. Large pleural effusions require chest tube for drainage as they restrict lung expansion. Malignant pleural effusions may also occur in children with lung/breast cancers (rare), lymphoma or sarcomas and are almost exclusively (95% of cases) caused by metastases in the pleural space. Chemotherapy-induced pleural effusion (Gemcitabine/Docetaxel) has also been described, usually in combination with anasarca. They may require diagnostic aspiration of pleural fluid for cytology or insertion of a chest tube for therapeutic purposes (injection of sclerosing agents, pleurodesis, pleurectomy and insertion of indwelling pleural catheters) [2, 3]. Pleural effusions can be aspirated to relieve respiratory distress. Chemical pleurodesis is beneficial for recurrent pleural effusion.

Haemothorax is the presence of blood inside the pleural space. Haemothorax can be secondary to tumour rupture, tumour vascular invasion or interventions (biopsy or central line placement), and should be drained after correction of any clotting abnormalities to ensure lung expansion and monitoring of blood loss. Indication for thoracotomy would be a massive haemothorax, defined as 15 mL/kg initial tube drainage, or ongoing bleeding, defined as 3–4 mL/kg per hour over 4 hours.

Complete drainage of blood is of essence to prevent empyema and fibrothorax. Empyema and chylothorax represent other two conditions in which chest tube insertion may be required.

In cases of chylothorax, conservative treatment with diet and octreotide is the best option and mostly sufficient, unless respiratory distress develops.

### Technical issues

Drainage of the pleural space can be achieved using a sterile silicone or polyvinyl chloride tube inserted into the pleural cavity through the chest wall and connected to a drainage system. Both open and percutaneous (Seldinger) techniques can be used for insertion. The size of the chest tube depends on the nature of the fluid as well as the size of the patient. The procedure is performed in sterile conditions, either as a bedside manoeuvre or in the operating room. For the most part, tubes are inserted in the mid clavicular line in the fourth intercostal space, but certain circumstances, such as loculated effusions may require a different site. In this instance, the use of ultrasound guided techniques should be employed. Care should be taken to insert the tube over the top of a rib, to avoid injury to the neurovascular bundle that runs below each rib. Pleural drainage systems are available for connection to the chest tube, either as continuous or intermittent aspiration techniques.

Special care should be taken post insertion to ensure adequate functioning of the drainage system. Kinking, obstruction, dislodging or disconnections of the chest tube are common complications that should be avoided. Local complications related to chest tube insertion such as bleeding or soft tissue injury may need specific wound care management.

There are no absolute contraindications for the procedure, but coagulation parameters should be ideally normalised in children with bleeding disorders or in those receiving anticoagulant therapy to reduce the risk of ongoing bleeding.

### Chest tube versus pigtail insertion

Recently, there has been a move towards the use of soft chest tubes or pigtail-like systems for pleural space drainage especially in children [4]. Both techniques are effective. The overall complication rates are comparable, and vary between 5% and 8%. Pigtail catheters have certain advantages related to their smaller size (12–14 Fr). They are less traumatic, more flexible and easier to insert. They cause less pain and tissue disruption, however they are more liable to kinking, obstruction and ‘clogging’. Their placement should be performed under ultrasound guidance, which may be difficult in some settings. Their overall efficacy is estimated to be around 83%. Other advantages are related to shorter hospital stay when compared to traditional chest tube insertion. Some studies have reported that pigtail catheters can be also used for the first-line drainage of tension pneumothorax.

Chest tubes are larger in size (28–40 Fr) and allow better drainage of haemothorax and thick fluids. Their insertion is more painful and requires larger skin incisions. Due to their large size, injuries to chest wall structures can occur and may result in serious complications such as bleeding from intercostal arteries or pneumothorax. Their use has also been linked to complications such as lung injury, dislodgement, open or tension pneumothorax. Their overall efficiency is estimated to be around 80% [5]. It is recommended by some workers that in patients with empyema, haemothorax or chylous effusions, large-bore chest tubes are better used [6, 7].

Ultimately, the choice of pleural space drainage technique should be decided by the paediatric surgeon and based on the nature of drainage required as well as the available resources.

## Central Venous Line Complications

### Epidemiology

Multiple lumen catheters are indicated in patients undergoing intensive treatments or stem cell transplantation, because of complexity of therapies, since administration of multiple intravenous drugs and solutions is accompanied with non-negligible risk of undesirable reactions of the drug interacting with other substance (drugs and inappropriate IV solutions as diluent, drug–drug incompatibility, etc.). For those reasons, dual lumen vascular access devices are often preferred and their placement is strongly encouraged by physicians. However, handling and caring of multiple lumen catheters is associated with higher risk of infection compared to single lumen devices (more manoeuvres and actions required), so their use as the first-choice device is still debated.

### Surgical management

In case of catheter-related blood stream infection, catheter removal should be considered, depending on the agent and the remaining venous access sites. This procedure should be done in a dedicated procedure room equipped with adequate facilities, under sedation, with trained medical and nursing staff and according to institutional guidelines. Surgical technique must include blunt dissection and removal of the polyester cuff together with the device. A retained cuff may remain as an innocuous foreign body, but may also be nidus for infection/chronic inflammation, may create determine a false image (calcification deposits or metastases) at radiology and last but not least have a cosmetically unacceptable reaction on the skin of the patient. In selected cases, change of catheter over a guidewire could also be considered, in order to minimise risks of anaesthesia/surgical procedure during septic condition or in the most acute phase of aplasia.

Catheter dislodgment and/or tip migration may lead to malfunction or inability to impossibility in using the device either in infuseion or withdrawing blood-draw and, in worst cases, to complete removal. Children undergoing chemotherapy and/or high dose steroids are more prone to these complications for different reasons (reduced healing ability and coagulation, increased infection, skin and subcutaneous tissue disease such as GvHD, etc.). Strategies to reduce those events includes the use of non-cuffed third generation polyurethane (which are also less predisposed to rupture compared to silicone-made devices) secured with both sutureless devices and subcutaneously anchored securement systems. Use of cyanoacrylate glue on the exit site has been also demonstrated to be a valid option to reduce failure of VADs also in the paediatric population (better securement, reduced infection rate for quick skin closure and healing, good haemostatic action, etc.)

**Please also refer to**: *IPSO Guideline*: Venous access for the paediatric cancer patient. page 38

## Insertion of Dialysis Catheter

### Tumour lysis syndrome (TLS)

TLS is a metabolic and oncological emergency that is encountered in oncology clinical practice and may occur spontaneously or shortly after administration of chemotherapy. It is associated with hyperkalaemia, hyperphosphataemia, hypocalcaemia and hyperuricaemia leading to end-organ damage. Most symptoms are related to the release of intracellular chemical substances that cause impairment in various organs. This can lead to acute kidney injury, fatal cardiac arrhythmia(s) and death. Since this condition is lethal, it is imperative to promptly identify patients at high risk for developing TLS and to start preventative therapy [8].

The diagnosis is based on laboratory and clinical criteria that were declared in 2004 to identify those patients who have metabolic abnormalities at baseline (spontaneous TLS) or after initiation of cytotoxic therapy [9–11]. A grading system (score from 0 to 5) for measuring the severity of clinical TLS based on the degree of serum creatinine elevation and the degree of medical intervention required has been reported [10].

### Prevention

Close observation, monitoring of fluid status and laboratory investigations, and the use of sufficient intravenous fluid and hypouricaemic agents are the main preventative options. Prevention is focused on preservation of renal function. Prophylaxis should be started whenever possible prior to the initiation of chemotherapy, especially in those patients where tumour lysis may occur, such as patients with very large tumour burden.

### Treatment using hydration, hypouricaemic agents and electrolyte management

It is fundamental to obtain baseline values prior to the initiation of cytoreductive therapy or administration of hypouricaemic agents. Monitoring includes serum uric acid, electrolytes, lactate dehydrogenase and creatinine levels. Hydration and diuresis are essential for management. Adequate hydration improves the intravascular volume, enhances renal perfusion and glomerular filtration rate and further promotes the urinary excretion of uric acid, potassium and phosphate. This scenario may consequently delay and prevent the need for renal replacement therapy.

Allopurinol is a xanthine oxidase inhibitor that blocks *de-novo* synthesis of uric acid and it is the preferred prophylactic agent in low-to-intermediate risk patients. However, it has several limitations as it does not break down preexisting uric acid and urate nephropathy can occur. Consequently, it is not the preferred agent in the presence of hyperuricaemia. Furthermore, xanthine nephropathy should be considered in patients who develop TLS while receiving appropriate prophylactic therapy with allopurinol. Allopurinol is excreted by the kidneys and the dose should be accordingly adjusted in those patients with renal insufficiency. Additionally, hypersensitivity reactions may happen. Lastly; because allopurinol reduces purine degradation, chemotherapeutic agents such as 6-mercaptopurine and azathioprine must be dose reduced by 50%–70% when concurrently administered. Rasburicase is a recombinant urate oxidase that converts uric acid to the highly soluble form allantoin. Therefore, it is an effective alternative to allopurinol in the treatment of hyperuricaemia and it is the preferred prophylactic agent for patients at high risk of TLS. It is also the treatment of choice for established TLS.

Patients with hyperkalaemia or hypocalcaemia should be placed on intensive cardiac monitoring, and laboratory and chemical tests should be repeated every 4–6 hours due to the risk of fatal cardiac arrhythmia. A serum potassium level above 7.0 mEq/L or widening of the ECG QRS complex requires immediate intervention. Standard therapies to decrease the potassium level include loop diuretics, insulin and glucose, inhaled β-agonists, polystyrene sulphate and calcium gluconate for symptomatic hyperkalaemia or electrocardiographic changes. These measures are often a temporary bridge to haemodialysis. Hyperphosphataemia is treated by phosphate binders; however, the presence of secondary hypocalcaemia can be life-threatening and generally necessitates the use of haemodialysis.

### Renal replacement therapy and haemodialysis in selected patients

Indications for renal replacement therapy in TLS are similar to those in patients with other causes of acute renal injury. Such indications include significant fluid overload, uraemia, severe electrolytes and metabolic disturbances. This is particularly true in TLS cases with oliguria because of potentially rapid release and accumulation of electrolytes and metabolites, which could lead to sudden death. Hyperphosphataemia-induced symptomatic hypocalcaemia may also warrant dialysis, in which continuous renal replacement therapies (CRRT) may be the preferred modality as phosphate clearance with dialysis therapy is time-dependent. For these reasons, CRRT such as haemofiltration, haemodialysis and haemodiafiltration are preferred rather than peritoneal dialysis, as these approaches result in better phosphate and uric acid clearance rates and faster clinical improvement. There are no major trials showing which approach of haemodialysis is more superior to the other. For the majority of patients, intermittent haemodialysis may be enough. However, in patients with rebound of electrolytes imbalance with intermittent haemodialysis, CRRT may be required. Dialysis should be carried out until there is adequate return of renal function and urine output. Prophylactic CRRT in children at high risk of TLS was also reported to be beneficial [12, 13].

### Hyperleukocytosis

Hyperleukocytosis (HL) is considered when peripheral blood leukocyte count exceeds 100,000/mm3, with acute leukaemia as its most common aetiology in paediatric oncology practice. The increased blood viscosity secondary to high white cell count and leukocyte aggregates, results in stasis in the small blood vessels. This predisposes to neurological, pulmonary or gastrointestinal complications. In addition, such patients are at risk for TLS due to the increased tumour burden [14, 15]. Initial management includes aggressive hydration, prevention of TLS and correction of metabolic abnormalities. A red blood cell transfusion is not indicated in a haemodynamically stable child, as it adversely affects the blood viscosity. Leukapheresis is the treatment of choice for a very high count, or in patients with symptomatic HL [14, 15]. The technical expertise requires a relatively difficult venous access in younger children, risks of anticoagulation and the possible non-availability of the procedure in emergency ‘out of hours’ practice are some key limitations of leukapheresis. However, it is a worthwhile procedure and performed with relative ease in experienced centres that regularly perform the procedure. An exchange transfusion is often a practical option when HL is complicated with severe anaemia. The partial exchange aids in correcting both problems without the risk of volume overload or hyperviscosity which are the key limitations of hydration and blood transfusion, respectively.

### Supportive care

Hyper-hydration is indicated in all patients affected by HL, with the goal of haemodilution and reduction of viscosity. Aggressive hydration is generally performed at levels of 2–3 l/m2/d, or 200 mL/kg/d for patients with a body weight of less than 10 kg with the aim of maintaining a urine output greater than 4 mL/kg/h for infants and 100 mL/m2/h for older patients. The use of balanced or isotonic solutions related to the patient’s age, cardiac function and urine output is recommended. Potassium must not be added to the hydration fluid. Renal replacement therapy should be considered if necessary [15].

Coagulation abnormalities include DIC, thrombocytopenia and anaemia. DIC is mainly determined by high cell turnover and associated high levels of released tissue factor that triggers the extrinsic pathway via Factor VII. Since TLS may occur as a result of spontaneous or treatment-induced cell death, DIC and TLS are closely related. The treatment of DIC is primarily directed against the cause that produces the blood coagulation disorder. It is based on platelet transfusions and standard measures to restore normal coagulation such as substitution of FFP or fibrinogen.

### Induction chemotherapy and leukapheresis

The initiation of induction chemotherapy is an essential part of the treatment of HL and leukostasis. In some patients, it has contraindications for immediate induction treatment such as severe metabolic disturbances or renal insufficiency. Intravenous steroids should be started at low dose then gradually escalated to full dose and this can lead to significant reduction in the circulating WBC count.

Leukapheresis is a procedure that involves the withdrawal of circulating blast cells from the body, with re-infusion of leukocyte-poor plasma through a mechanism of separation and retention of blood components. The aim of this procedure is to reduce the peripheral white blood count, limiting the severity of TLS and DIC. It can be continuous or discontinuous, depending on the technique of blood sampling and infusion used. It is typically reserved for patients with symptomatic HL who are unable to start induction chemotherapy immediately. Contraindications such as cardiovascular comorbidities, haemodynamic instability and coagulation disturbances should be evaluated carefully in order to avoid any extra-procedural risk for the patients.

A single session of leukapheresis reduces the WBC count by 20%–50%. A reduction of 50% in the peripheral count is equivalent to an 85% decrease of the circulating WBC mass. However, the effect is generally transient since blast counts often rebound quickly after leukapheresis. The decision to perform additional leukapheresis procedures should be based on symptoms and peripheral WBC count. Leukapheresis cannot be recommended for routine therapy as a treatment in patients with high blast counts because of its potential complications. Another problem regarding leukapheresis is that it requires specialised equipment and trained personnel that are not always available as well as the positioning of a vascular access [16–18]. Paediatric and adolescent patients are rarely treated by apheresis procedures especially if the bodyweight is less than 40 kg as apheresis systems are mainly constructed for adults. Efforts to overcome in paediatric patients are the limited possibilities for venous access, the management of the disparity of extracorporeal volume of the apheresis system and the total blood volume of the patient, the management of the anticoagulation and to meet the special demands of younger patients [19]. Thus, it appears that most indications for the use of apheresis in children are derived from evidence obtained in adult studies.

## Vascular Access

The majority of apheresis procedures are centrifugation based; therefore, they require withdrawal blood flow rates of 50–150 mL/minute. In contrast, filtration therapies require a blood flow rate of at least 150–200 mL/minute. Other considerations specific to therapeutic apheresis (TA) include whether the treatment relies on discontinuous, sequential blood exchange cycles (one lumen is sufficient) or continuous processing (two lumens are needed). When a CVC is necessary for a limited (<2 weeks) course of TA, a non-tunnelled, semi-rigid polyurethane catheter should be considered. For a longer duration (>2 weeks) of TA, a tunnelled CVC is preferred over a non-tunnelled CVC due to less risk of infection. Typically, tunnelled catheters designed for long-term use (weeks to months) are made of either silicone or polyurethane, the latter has a lower incidence of rupture and the least thrombogenicity. The preferred venous site of CVC insertion are the brachiocephalic vein and the internal jugular vein, and both ultrasound guidance with fluoroscopy have been shown to be associated with a lower rate of complications during insertion. CVCs are associated with a higher total complication rate. They include infection (2%–28%), thrombosis (0.2%–11%), haemorrhage (2%–14%) and venous stenosis (10%–26%) with internal jugular catheters and up to 42% with subclavian vein catheters. In most series, the incidence of total adverse events associated with all vascular access devices is low at 1%–2%, especially when ultrasound guidance and the brachiocephalic vein are routinely considered [20]. Complications of peripheral cannulation include risk of infection, venous infiltration, patient discomfort, thrombosis and sclerosis of veins and the loss of future venous access. Peripheral vein access for TA is not a viable option in children due to their small venous calibre anatomy. Peripherally inserted central catheters are too small in calibre (4–5 Fr) to accommodate the negative pressure and blood flow rates required for TA procedures. Arteriovenous fistulas and grafts are viable options in adult patients for long-term access when the treatment duration is expected to be over a period of several months or years [16, 21].

## Tracheostomy Insertion in Certain Emergency Situations

Tracheostomy is a commonly performed manoeuvre in adult patients; however, such procedures are less often undertaken in children and conducted in about 3% of all paediatric patients [22]. Paediatric tracheostomy was first described during the 15th Century for the relief of airway obstruction due to huge, enlarged tonsils [23]. Tracheostomy in paediatric oncology patients is an urgent procedure that is usually performed within surgical emergency care settings. Upper airway obstruction secondary to advanced malignant tumours in the nose or pharynx is the commonest indication in paediatric oncology [24]. Head and neck malignancies, mainly rhabdomyosarcoma in the nasal cavity or nasopharynx are often initially asymptomatic but may progress rapidly to airway obstruction in patients unresponsive to treatment or in relapse, or eventually in children having palliative care [25].

### Preoperative evaluation and surgical aspects

Clinical assessment is usually conducted rapidly through vital signs measurement, followed by evaluation of oxygen saturation. Administration of corticosteroids may temporarily improve the airway function; however, in patients without significant improvement and with progressive respiratory distress, urgent tracheostomy is a lifesaving procedure.

The procedure is performed under aseptic precautions and general anaesthesia with endotracheal intubation (with or without fibre optic guidance). The laryngeal or face mask may be other options for anaesthesia if difficulty in intubation is encountered. The tracheostomy operation is conducted in the supine position with a slight extension of the patient’s neck. A transverse skin crease incision (between suprasternal notch and cricoid cartilage) is deployed due to its cosmetic advantage, however a vertical one is theoretically considered less vascular. Blunt dissection is continued thereafter in the vertical plane using an artery forceps in the midline followed by retraction of the strap muscles until the trachea is visible. Haemostasis is achieved by bipolar diathermy to obtain a bloodless operative field. The isthmus of thyroid gland is dissected then the tracheal fascia is opened to expose second, third and fourth tracheal rings. Two stay sutures are placed on either side of the airway and a vertical incision created between the stay sutures. Bleeding and/or other secretions should be properly suctioned to clear the surgical field, and a suitable-sized tracheostomy tube is inserted and secured to the neck to complete the procedure. The endotracheal tube is removed once the tracheostomy is secured in place. A flexible fiberoptic bronchoscope can be used to assess the final anatomical position of the distal part of the tube that should ideally be placed alongside the second, third and fourth tracheal rings [22].

### Post-procedural care and complications

Postoperative management of the tracheostomy tube is crucial as the majority of children may require a long-duration placement or even a permanent tracheostomy. Such care of the tube is usually performed by a trained stoma nursing team or by the parents after further training. Early and late complications of the tracheostomy procedure in children are not uncommon, with an incidence rate that varies between 7% and 40% in published studies [26]. Bleeding or injury to various vital structures are the main intraoperative complications. Inadvertent injury to the innominate artery, or aberrant vessels, or thyroid gland can cause profuse haemorrhage during the procedure. Furthermore, the cervical oesophagus or recurrent laryngeal nerve(s) may be injured during dissection or exposure. Postoperative complications may sometimes lead to life-threating respiratory distress, and this may occur due to injury of the main bronchus or the trachea by the inserted tube. Airway obstruction may be also encountered by mucus plugging. Pneumothorax can occur due to air leak(s). Subcutaneous ‘surgical’ emphysema and pulmonary oedema are also potential post-procedural morbidities that may happen [22].

Tracheostomy insertion in paediatric oncology patients is a critical surgical manoeuvre that needs special perioperative protocol(s) by the team care-givers to manage the child safely meanwhile avoiding any serious consequences.

## Take Home Messages

- Surgeons must practise and have expertise in advanced life support to treat emergencies in paediatric oncology.
- Surgeons must be aware of the characteristics of paediatric oncology diseases and their urgencies to correctly manage patients, without impairing the oncological prognosis.
- Be aware that surgical complications can occur in the postoperative period.
- In neutropenic patients, treatment should be tailored individually. Conservative treatment often is enough for the majority of scenarios, but surgery can be needed.
- In case surgery is indicated, the multidisciplinary team should talk about the best timing to operate the patient and the surgeon should not be afraid to operate, if there is blood products and ICU support available.

## References

[1] Interiano RB, McCarville MB, Wu J (2015). Pneumothorax as a complication of combination antiangiogenic therapy in children and young adults with refractory/recurrent solid tumors. J Pediatr Surg.

[2] Bibby AC, Dorn P, Psallidas I (2019). ERS/EACTS statement on the management of malignant pleural effusions. Eur J Cardiothorac Surg.

[3] Feller-Kopman DJ, Reddy CB, DeCamp MM (2018). Management of malignant pleural effusions. An official ATS/STS/STR clinical practice guideline. Am J Respir Crit Care Med.

[4] Martin K, Emil S, Zavalkoff S (2013). Transitioning from stiff chest tubes to soft pleural catheters: prospective assessment of a practice change. Eur J Pediatr Surg.

[5] Elsayed A, Alkhalifa R, Alodayni M (2018). Implication of pigtail catheters vs. chest tube drainage. Int J Community Med Public Health.

[6] Liman ST, Elicora A, Akgul AG (2014). Is a small-bore catheter efficient for most pleural pathologies?. Surg Today.

[7] Roberts JS, Bratton SL, Brogan TV (1998). Efficacy and complications of percutaneous pigtail catheters for thoracostomy in pediatric patients. Chest.

[8] Adeyinka A, Bashir K (2021). Tumor Lysis syndrome. StatPearls.

[9] Belay Y, Yirdaw K, Enawgaw B (2017). Tumor Lysis syndrome in patients with hematological malignancies. J Oncol.

[10] Cairo MS, Bishop M (2004). Tumour lysis syndrome: new therapeutic strategies and classification. Br J Haematol.

[11] Williams SM, Killeen AA (2019). Tumor Lysis syndrome. Arch Pathol Lab Med.

[12] Cheung WL, Hon KL, Fung CM (2020). Tumor lysis syndrome in childhood malignancies. Drugs Context.

[13] Coiffier B, Altman A, Pui CH (2008). Guidelines for the management of pediatric and adult tumor lysis syndrome: an evidence-based review. J Clin Oncol.

[14] Jain R, Bansal D, Marwaha RK (2013). Hyperleukocytosis: emergency management. Indian J Pediatr.

[15] Ruggiero A, Rizzo D, Amato M (2016). Management of hyperleukocytosis. Curr Treat Options Oncol.

[16] Connelly-Smith LS, Linenberger ML (2015). Therapeutic apheresis for patients with cancer. Cancer Control.

[17] Malchesky PS, Koo AP, Roberson GA (2010). Apheresis technologies and clinical applications: the 2007 International Apheresis Registry. Ther Apher Dial.

[18] Stegmayr B, Wikdahl AM (2003). Access in therapeutic apheresis. Ther Apher Dial.

[19] Witt V, Stegmayr B, Ptak J (2008). World apheresis registry data from 2003 to 2007, the pediatric and adolescent side of the registry. Transfus Apher Sci.

[20] Avanzini S, Mameli L, Disma N (2017). Brachiocephalic vein for percutaneous ultrasound-guided central line positioning in children: a 20-month preliminary experience with 109 procedures. Pediatr Blood Cancer.

[21] Golestaneh L, Mokrzycki MH (2013). Vascular access in therapeutic apheresis: update 2013. J Clin Apher.

[22] Watters KF (2017). Tracheostomy in infants and children. Respir Care.

[23] Goodall EW (1934). On infectious diseases and epidemiology in the hippocratic collection: (section of the history of medicine). Proc R Soc Med.

[24] Gergin O, Adil EA, Kawai K (2016). Indications of pediatric tracheostomy over the last 30 years: has anything changed?. Int J Pediatr Otorhinolaryngol.

[25] Radzikowska J, Kukwa W, Kukwa A (2015). Rhabdomyosarcoma of the head and neck in children. Contemp Oncol (Pozn).

[26] Goldenberg D, Ari EG, Golz A (2000). Tracheotomy complications: a retrospective study of 1130 cases. Otolaryngol Head Neck Surg.

# Discussion: Surgical biopsies in the management of BMT patients

**Published:** 2022

**Authors:** Abdulrasheed Nasir, Joyce Freitas, Alessandro Crocoli, Chan Hon Chui

BMT patients often develop life-threatening conditions with similar clinical presentations but different aetiologies. Histological and microbiological examination of biopsies from the affected organ may be the only means of arriving at the diagnoses [1].

## Indication for biopsy

Most patients manifest a wide range of non-specific symptoms and signs. The symptoms may be related to GI system, pulmonary, hepatobiliary or skin (most maculopapular rash). Radiological imaging modalities are unlikely to provide definitive diagnoses [2]. If required, the GI tract is best evaluated by endoscopy and biopsy [3–5] while dysfunction of the liver [6, 7] and the lung [8], and the skin lesions may undergo surgical biopsy for accurate diagnosis [1].

## Workup

- Complete blood counts, clotting profile.
- Relevant radiological imaging associated with the condition of interest.

## Surgical management

Biopsies should be obtained by the least invasive means. Sigmoidoscopy is preferred over colonoscopy for GI biopsy. Percutaneous image-guided biopsy core needle or Tru-cut biopsy of liver and lung is preferred over an open incisional biopsy.

## Preoperative considerations

It is essential that patients are as stable as possible before going to operating theatre. Good communication between the medical and surgical teams is critical. Patients should be adequately supported with the necessary blood products. In general, the ideal preoperative hemoglobin should be at least 10 g/dL, platelets > 80 × 109/L and INR less than 1.2. A lower haemoglobin level may be acceptable if the patient has chronic anaemia, and refractory thrombocytopenia may be overcome by platelet transfusion at the induction of anaesthesia so as to maximise the platelet concentration at surgery. The procedure should always be carefully planned to reduce the amount of time spent.

## References

[1] McCarville MB, Adelman CS, Li C (2005). Typhlitis in childhood cancer. Cancer.

[2] Wick MR, Moore SB, Gastineau DA (1983). Immunologic, clinical, and pathologic aspects of human graft-versus-host disease. Mayo Clin Proc.

[3] Roy J, Snover D, Weisdorf S (1991). Simultaneous upper and lower endoscopic biopsy in the diagnosis of intestinal graft-versus-host disease. Transplantation.

[4] Terdiman JP, Linker CA, Ries CA (1996). The role of endoscopic evaluation in patients with suspected intestinal graft-versus-host disease after allogeneic bone-marrow transplantation. Endoscopy.

[5] Wall SD, Jones B (1992). Gastrointestinal tract in the immunocompromised host: opportunistic infections and other complications. Radiology.

[6] Anand N, Anand A, Anand A (1995). Fungal liver involvement in bone marrow transplant recipients. Clin Infect Dis.

[7] Wasserheit C, Acaba L, Gulati S (1995). Abnormal liver function in patients undergoing autologous bone marrow transplantation for hematological malignancies. Cancer Invest.

[8] Winer-Muram HT, Gurney JW, Bozeman PM (1996). Pulmonary complications after bone marrow transplantation. Radiol Clin North Am.

# Discussion: Fertility preservation in children

**Published:** 2022

**Authors:** Marianna Cornet, Lucas E Matthyssens, Cristina Martucci, Stefano Avanzini, Justin T Gerstle, Denise B Klinkner, Mark Powis, Florin Filip, Khalid Elmalik, Sarah Braungart, Rodrigo Romao, Alexander Siles Hinojosa, Fernanda Kelly Marques de Souza, Sabine Sarnacki

## Epidemiology

Childhood cancers represent nowadays 1% of all cancers. Advances in understanding paediatric cancers and their biology allowed improving therapeutics, leading to 80% long-term overall survival [1], mostly by increasing the intensity of targeted chemo- and/or radiation therapies. As a result, many paediatric patients may suffer from long-term adverse effects [2]. Fertility disorders are one of the major long-term sequalae after paediatric cancer treatment with risks of reduced fertility and important effects on psychological health.

They may result from gonadotoxic treatments as chemotherapy and radiotherapy but also from mutilating surgery [3].

## Induced Infertility Mechanisms

In girls: Alkylating agents (such as cyclophosphamide) which are widely used and particularly in conditioning high-dose chemotherapy are the most prone to lead to ovarian failure.

Ionising radiation acts on dividing and non-dividing cells. As with chemotherapy, the effects of radiotherapy on ovarian function depend on the patient’s age, the field of treatment, the daily treatment doses and the total amount of radiation received. Doses of 10–20 Gy in children and 4–6 Gy in adults are associated with permanent ovarian failure. In addition, irradiation of the uterus in the childhood is known to predispose to premature delivery or to get offspring with low birth weight [1, 4, 5]. Dose fractionation and intensity modulation in external beam radiation treatment (EBRT) are associated with potentially lower damage of ovarian function, due to cell reparations that can occur between doses [6], [7]. Pelvic brachytherapy may be associated with vaginal stenosis making conception more difficult [8]. Finally, irradiation of the hypothalamic pituitary adrenal (HPA)-axis can also lead to gonadotropic failure, usually for doses higher than 30 Gy. However, the ovarian reserve in these cases remains normal [9].

In boys: The prepubertal gonads are sensitive to the gonadotoxic effects of chemotherapy administrated during childhood. In males, alkylating agents may affect spermatogenesis, but testosterone secretion is usually well preserved.

In contrast, radiation therapy affects both spermatogenesis and testosterone secretion. Spermatogonia are impaired by doses as low as 0.15 Gy and completely die from a dose of 2 Gy. Prepubertal boys seem to be more susceptible to the effects of irradiation on spermatogenesis and Leydig cell function than adolescent and adult males. It should be noted that, in contrast to females, fractioning doses induce a much higher toxicity than that of a single dose. Lastly, irradiation doses above 25–30 Gy on the HPA-axis can induce hypogonadotropic hypogonadism with dose-dependent relation [10], leading to pubertal delay, sexual disorders or sterility.

A 50% decrease in descent has been reported in childhood cancer survivors versus random adults [10] and a significant increase in fertility disorders (46% in survivors versus 17.5% in siblings (RR = 2.64, 95% confidence interval: 1.88–3.70, *p* < 0.001)) with sperm alterations has been reported in a follow-up study including 1,600 patients treated for childhood cancer [11, 12].

## Preoperative Workup

Blood analysis: Complete blood count and coagulation profile, if surgical treatment is being considered. In addition, if cryopreservation is planned, serologies for virus are usually required (HIV serology, hepatitis B serology, hepatitis C serology and Treponema Pallidum Hemagglutination Assay (TPHA)/Venereal Disease Research Laboratory (VDRL)).

Imaging: No imaging is needed but in case of gonadal transposition to protect form radiotherapy, it is mandatory to share the planned radiotherapy field with the radiotherapist in order to displace the gonads appropriately.

Regarding the wide spectrum of proposals for preservation of fertility which depends on the age, tumour type and treatments, it is highly recommended to discuss indication for fertility preservation in a multidisciplinary board integrating medical oncologist, radiotherapists, preservation fertility specialists.

A detailed informed consent is proposed and discussed with the patient and his/her parents.

## Fertility preservation in girls

### Ovarian and Genital Tract Sparing Surgery

In girls, ovarian sparing surgery is mandatory to preserve fertility in case of benign ovarian tumours, which represent around 90% of childhood ovarian lesions (benign germ cell tumours (GCTs) or benign epithelial tumours).

Indeed, 13% of ovarian teratomas are bilateral (either synchronous or metachronous) [13], and unilateral ovariectomy leads to depletion of the total number of primordial follicles and therefore might increase the risk of premature or early menopause, although not clearly demonstrated in children. If in adults evidence indicates that having a single ovary does not reduce the fertility potential (with normal live birth rates, spontaneously or after assisted reproductive techniques), evidence from long-term studies in children is missing [14, 15]. In the absence of evidence in the paediatric population, ovarian sparing surgery should always be considered.

In everyday practice, deciding whether to spare the ovary because the lesion seems benign or to perform a total ovariectomy because signs of malignancy are present is not always straightforward. Two different situations should be considered. In asymptomatic (painless) girls, the surgeon has enough time to perform reliable imaging (ensuring that the tumour does not show any sign of malignancy) and to dose tumour markers alpha-fetoprotein (AFP), human chorionic gonadotrophin (HCG), Inhibin B, anti-Mullerian hormone and Calcaemia. When the patient is complaining of acute pain due to adnexal torsion or ovarian rupture, the best approach is to perform a laparoscopic exploration in emergency. If ovarian rupture is diagnosed, peritoneal inspection, sampling of ascites is the rule. Complete ovariectomy or adnexectomy in the same operative time should be discussed only if the ovary and/or the peritoneum indicate malignant features. If adnexal torsion is seen, detorsion is recommended as it allows to approach the potential underlying diagnosis (benign or malignant tumour) with imaging and markers dosage in the post-operative period.

Operative technique: it is highly recommended to start with laparoscopic exploration of the abdominal cavity, with sampling of ascites in the upper abdomen and pelvis and sampling of any peritoneal suspect lesions, particularly on the diaphragm. Ovarian sparing surgery is then performed through a suprapubic incision (Pfannenstiel). Although this approach is debatable, as some surgeons prefer complete laparoscopic resection, this technique allows to spare as much ovarian tissue as possible while avoiding any intraperitoneal spillage (of importance in benign GCTs as well as in the presence of non-secreting malignant components) [16].

Postoperative follow-up with repetitive pelvic ultrasound examinations during childhood is recommended to rule out metachronous ipsilateral or contralateral lesions at an early stage, allowing iterative sparing surgery. This follow-up also gives the opportunity to inform the young girl about the symptoms that should alert for a potential adnexal torsion and in case of an important amputation of the ovarian parenchyma of the different assisted medial procreation techniques available after puberty.

Preservation of genital tract should also be considered when surgery is planned for tumours involving these structures. Different entities encountered are: rhabdomyosarcoma (RMS) of the vagina, cervix or uterus, malignant GCTs of the vagina and clear cell adenocarcinoma (mostly diagnosed later in life). Prognosis of these tumours relies on the local tumour control obtained nowadays with less radical surgical management than in past decades. For urogenital RMS, treatment may be achieved without surgery when remission is obtained after chemotherapy or may be completed by local brachytherapy avoiding mutilating surgery, which is currently exceptional [17, 18]. For vaginal GCT, surgical resection of the primary location is always mandatory but the efficiency of neoadjuvant chemotherapy usually allows to avoid mutilating surgery and to perform a partial colpectomy.

### Ovarian Transposition (Oophoropexy)

Ovarian transposition was the first procedure proposed to preserve fertility in girls with cancer and is indicated for patients with tumours requiring pelvic radiations of 42–58,4 Gy, much higher doses than those that can induce loss of ovarian function (4–20 Gy) [4]. The ovaries are very radiosensitive organs. The extent of damage depends on the dose of radiotherapy that reaches the ovaries, the age of the girl at the time of treatment and the associated drugs used for chemotherapy. Doses of 10–20 Gy in children and 4–6 Gy in adults are associated with permanent ovarian failure [19]. Preliminary ovarian transposition should be considered for all children with tumours requiring radiotherapy implicating part of the pelvis. RMS of the bladder, vagina or uterus, or soft tissue or pelvic bone sarcomas such as Ewing’s sarcoma, are the main indications for ovarian transposition in children [20–22].

Irrespective of the surgical technique, ovarian transposition aims to place the ovaries outside of the irradiation field. The planning target volume should be precisely defined by the surgeon and the radiotherapist before ovarian transposition. Various sites for ovarian transposition can be considered. In case of a midline irradiation field (urogenital tumours or medulloblastoma), both ovaries are usually placed away from the midline, laterally in the paracolic gutters, or laterally and anteriorly near the inguinal ring (bilateral ovarian transposition). In case of a lateral tumour (RMS or Ewing’s sarcoma), the compromised ovary is placed on the opposite site of the tumour (unilateral ovarian transposition). In some cases of Hodgkin’s lymphoma, when the irradiation field implicates the bilateral iliac chains and inguinal regions, the ovaries are placed in line with the iliac crests (bilateral ovarian transposition) [23].

Laparoscopic ovarian transposition is performed under general anaesthesia. The bladder is emptied with a transurethral Foley catheter. The patient is positioned supine and in Trendelenburg position, which causes the intestine to fall into the upper part of the abdomen to free the pelvis and enable visualisation of the genital tract. An umbilical trocar is inserted either via an infra-umbilical incision or directly through the umbilicus. Carbon dioxide is insufflated at a pressure of 8–12 mm Hg dependent on the patient’s age and weight. Two working trocars (3–5 mm) are inserted under direct vision into the right and left hypogastric regions, almost as the same level as umbilicus, depending on the child’s age. A 0° or 30° angled camera can be used. In most of the cases, is it not necessary to section any ovarian ligament. In prepubertal girls, the ovaries are located higher in the pelvis and the ligaments are more stretchable than in adult women [24]. The blood supply to the ovaries should be carefully preserved, especially the ovarian vessels should be examined to ensure the absence of any kinking or direct injuries [25]. The ovaries are grasped with an atraumatic forceps and mobilised above the iliac crest level (in the case of midline irradiation field) as high as possible without any dissection or division of the ovarian ligaments or of the fallopian tube. The ovaries are sutured to the peritoneum with resorbable or non-resorbable suture material and are marked by metallic clips to make them visible on imaging before the initiation of EBRT.

In case of pelvic brachytherapy, ovarian transposition is only needed for a short time. The ovaries can then be sutured to the anterior abdominal wall by a transfixing stitch of non-resorbable suture material, knotted on the outside of the patient on a pledget. The stitch knotted on the outside holding the ovary to the abdominal wall is removed at the end of brachytherapy, to return the ovaries to their normal position without the need for reoperation [24].

Whatever technique used, the surgical procedure should be performed as close to the time of radiation treatment as possible, due to the risk of remigration of the ovaries [26].

Few studies have reported long-term results of ovarian transposition in children. Preservation of endocrine function has been estimated to range between 60% and 80% [27, 28]. Concerning side effects, the major complication reported is painful ovarian cysts [29], which raises the question of a detransposition upon completion of treatment. This side effect is not systematic and depends on the procedure performed. It seems reasonable to adopt a wait and see policy before planning a detransposition. Other side effects include abdominal pain and bowel obstruction.

### Uterine Transposition

In some cases, radiation is an important complementary treatment. But the area can involve the pelvic region and result in deleterious effect to fertility, which may affect the uterus and ovaries. Uterine and adnexal transposition to an upper abdominal region during radiation therapy may protect these organs with security. Some successful cases are described in adult women [29] and a recent case was described in a prepubertal girl [30]. Laparoscopic approach is recommended (see Minimal Invasive Surgery Guideline), but if not available, open surgery can be used, analysing the risks and benefits of the approach.

### Ovarian Tissue Cryopreservation

Main indications nowadays are represented by myeloablation before bone marrow transplantation, total body irradiation and high dose chemotherapy with alkylating agents [30].

There is some controversy about the amount of ovarian tissue to retrieve as some teams propose to do a partial ovariectomy or to harvest only the cortex [62]. Since follicular loss can reach up to 65% only due to the ischaemia hit [31], it seems for many groups essential to collect an entire ovary in younger children in order to get enough tissue or cells for the future pregnancy project. It has to be noted that in most of the team, the ovarian tissue is more used for fertility than for puberty restauration [32, 33]. In case abdominal surgery is planned, the procedure may be performed simultaneously but otherwise, a laparoscopic approach is recommended.

Laparoscopic ovariectomy for cryopreservation is performed under general anaesthesia. The bladder is emptied with a transurethral Foley catheter. The patient is positioned supine and in Trendelenburg position. An umbilical trocar is inserted either via an infra-umbilical incision or directly through the umbilicus. Carbon dioxide is insufflated at a pressure of 8–12 mm Hg dependent on the patient’s age and weight. Two working trocars (3–5 mm) are inserted under direct vision into the right and left flank, almost as the same level as umbilicus, depending on the child’s age. A 0° or 30° angled camera can be used. The ovary is grasped with atraumatic forceps. Haemostasis is performed with bipolar haemostatic forceps, and the mesovarium is cut with scissors. The ovary is placed within a bag and removed via the umbilical trocar.

After isolation and fragmentation, ovarian cortex fragments are slowly frozen in an automated freezer down to temperature of liquid nitrogen, in which they are stored. Histological analysis of some fragments is mandatory as it allows searching for malignant cells in both cortex and medulla, particularly in tumours with potential ovarian spread (haematological cancer and neuroblastoma) and also to estimate the follicular wealth of the tissue, notably in case of previous gonadotoxic treatment.

Cryopreserved ovarian fragments might further be used for either autografting or *in vitro* maturation of primordial follicles. Since Donnez *et al* [35, 36] and Meirow *et al* [37] published the first two pregnancies obtained after autograft of ovarian cortex, more than 200 livebirths have been documented in literature after autologous grafting of previously cryopreserved (or fresh) adult ovarian tissue [34]. The ovarian function is restored after 4 months following grafting and 23%–37% of these women will have pregnancies. In prepubertal patients, results are not assessable, as most of these patients have not achieved the age of parental desire. Poirot *et al* [38] confirmed that induction of puberty was efficient by heterotopic autografts of ovarian fragments. In 2015, Demeestere *et al* [39] reported the first pregnancy after cryopreservation at a paediatric age. The patient, who required myeloablation before bone marrow transplantation for sickle cell anaemia, was 14 years old but premenarchal at the age of cryopreservation. She gave birth 13 years later to a normal child, 2 years after bilateral autografting of ovarian fragments and natural conception. Beside this publication, no cohort studies or even isolated case of livebirths after prepuberty ovarian tissue cryopreservation for malignancy have been reported [38].

A major problem raised by autografting of previously cryopreserved ovary is the risk of reintroduction of the primary disease in the patient’s organism, especially when the primary disease is likely to give metastasis to gonads such as leukaemia or lymphoma. Shaw *et al* [41] managed to prove, in mice models, that cryopreserved ovarian tissue samples from donors with lymphoma can transmit cancer to grafted recipients. Recent studies showed that the risk is highest in leukaemia patients, moderate in gastrointestinal cancers and low in breast cancer, sarcomas, gynaecological cancers, Hodgkin’s and Non-Hodgkin’s lymphomas [40, 41]. A way to avoid this problem will be the *in vitro* culture of primordial follicle harvested from the cryopreserved tissue, a technic which is currently still experimental [42].

## Fertility preservation in boys

### Testicular Sparing Surgery

The most common types of prepubertal testicular tumours are GCTs: benign GCT (teratoma) and yolk sac tumours (YST) [43, 44]. The rarest types, accounting for less than 5% of the patients, are sex cord-stromal tumours (granulosa cells tumours and Sertoli or Leydig cells tumours).

In the GCT group, testicular sparing surgery has become the standard of care for teratomas, but malignant YST can only be treated by radical orchidectomy. The operation is performed through an inguinal incision, with early control of the spermatic cord at the internal ring as in radical orchidectomy. Intraoperative frozen sections can be useful to rule out malignancy.

Regarding sex cord-stromal tumours, although considered malignant, the excellent prognosis after inguinal orchidectomy in non-metastatic tumours [45–47], and increasing reports of complete cure after testicular sparing surgery [48–50] has prompted to discuss the possibility of sparing surgery in localised tumours. Moreover, Fresneau *et al* [48] proposed to offer sparing surgery in all types of childhood testicular tumours provided that they are small (less than the third of the testicular parenchyma), well-delimited on ultrasound, without AFP and/or HCG secretion and after intraoperative testicular frozen section assessment to exclude the diagnosis of a malignant GCT.

### Testicular Shielding

During the pelvic radiation therapy, testis can be the subject of unintended exposure, due to lack of protective devices or malpractice. So the option is to protect this region with a clam shell-shaped device. It is positioned around the scrotal sac to reduce testicular exposure [51, 52].

### Testicular Transposition

Temporary transposition of one or two testis in the inguinal region has been proposed for patients requiring radiotherapy to the scrotum in some cases of paratesticular RMS or in brachytherapy for bladder-prostate RMS [51]. After section of the gubernaculum testis by an inguinal approach, the testis is placed above the external oblique muscle outside the superficial ring of the inguinal canal. The spermatic pedicle can be protected by a non-resorbable silicone sheath, to facilitate the dissection when the testis can be replaced in the scrotum at the end of the radiotherapy course. This technique is rarely used and current results are not reported yet.

### Semen/Testicular Cryopreservation

While semen cryopreservation is an established way to preserve fertility in adult male patients, it is more difficult in adolescents. Pubertal boys can be unable to provide a semen sample via masturbation for sperm banking. In these patients, semen can be obtained via penile vibratory stimulation, electro-ejaculation, testicular sperm extraction (TESE) or microdissection TESE (mTESE) under general anaesthesia [52]. What makes this fertility preservation technique most difficult is the fact that semen samples in adolescence are frequently of poor quality, even so, this can be the only option in low- and middle-income countries. After cryopreservation, stored spermatozoa are further used for *in vitro* fertilisation, mainly intracytoplasmic sperm injection (ICSI) procedure.

The technique of sperm banking cannot be applied to prepubertal boys as they lack mature spermatozoa. Thus, following the example of female patients, testicular cryopreservation in prepubertal patients has been proposed. Before gonadotoxic cancer treatment is started, part of the testicular parenchyma is harvested via an open scrotal approach under general anaesthesia. One side or two sides testis harvesting is done according to local usages. When bilateral, one longitudinal approach along the median raphe or two scrotal approaches may be performed. There is no consensus on the volume of testicular tissue to harvest. The fact that there is a heterogeneity in the distribution of spermatogonia within the testis may favour a bilateral harvesting in order to get as much as cells as possible, and has a cosmetic advantage as both testis display then the same volume. The prepubertal testis contains germ cells from which spermatozoa can potentially be derived *in vitro* or *in vivo* after reimplantation, as reported in animal models [53–56]. Immature testicular tissue is hoped to proliferate and differentiate into mature sperm *in vitro* after subsequent reintroduction into seminiferous tubules, once the patient is cured and pubertal; alternatively, conserved sperm could also be used for ICSI. Testicular tissue can also be autotransplanted into the patient’s own testis, waiting for spermatogonia to differentiate into mature sperm cells [57]. This technique of testicular germ-cell harvested, cryopreservation and subsequent transplantation has been proved to be effective in mice [58, 59]. Its success in humans is yet to be proven [30, 60]. However, similarly to cryopreservation of ovaries, the problem raised by autografting of previously cryopreserved testicular tissue is the risk of reintroduction of malignancy, especially in blood cancers. One possible way to reduce this risk would also be through *in vitro* maturation of germ cells.

## Conclusion

While important advances have been achieved in childhood cancer treatment, with a lower mortality and a better life expectancy, consequent loss of fertility remains an important issue. At the initial treatment planning, efforts must be made to preserve fertility in both boys and girls. Organ sparing surgery in gonadal tumours is the best and easiest way when malignancy has been excluded. In young girls treated by abdominal and/or pelvic radiotherapy, ovarian transposition is the technique of choice (with or without associated ovarian cryopreservation). In children receiving gonadotoxic treatments, ovarian and testicular cryopreservation should be proposed as they appear very promising and remain currently the only suitable method for pre- and post-pubertal children. Finally, in view of the broad range of fertility protection methods available and progress in medically assisted reproductive techniques, the best option of fertility preservation and follow-up should be established at a multidisciplinary meeting, as soon as the treatment protocol has been defined.

## References

[1] Anderson RA, Mitchell RT, Kelsey TW (2015). Cancer treatment and gonadal function: experimental and established strategies for fertility preservation in children and young adults. Lancet Diabetes Endocrinol.

[2] Phillips SM, Padgett LS, Leisenring WM (2015). Survivors of childhood cancer in the United States: prevalence and burden of morbidity. Cancer Epidemiol Prev Biomark.

[3] Raffoul L, Capito C, Sarnacki S (2016). Fertility considerations and the pediatric oncology patient. Semin Pediatr Surg.

[4] Isachenko V, Todorov P, Isachenko E (2016). Cryopreservation and xenografting of human ovarian fragments: medulla decreases the phosphatidylserine translocation rate. Reprod Biol Endocrinol.

[5] Irtan S, Orbach D, Helfre S (2013). Ovarian transposition in prepubescent and adolescent girls with cancer. Lancet Oncol.

[6] Thibaud E, Rodriguez-Macias K, Trivin C (1998). Ovarian function after bone marrow transplantation during childhood. Bone Marrow Transplant.

[7] Presti AL, Ruvolo G, Gancitano RA (2004). Ovarian function following radiation and chemotherapy for cancer. Eur J Obstet Gynecol Reprod Biol.

[8] Levy A, Martelli H, Fayech C (2015). Late toxicity of brachytherapy after female genital tract tumors treated during childhood: prospective evaluation with a long-term follow-up. Radiother Oncol.

[9] Sudour-Bonnange H, Tabone MD, Thomas-Teinturier C (2013). Fertility preservation in children and teenagers with cancer. Bull Cancer (Paris).

[10] Sathyapalan T, Dixit S (2012). Radiotherapy-induced hypopituitarism: a review. Expert Rev Anticancer Ther.

[11] Green DM, Kawashima T, Stovall M (2010). Fertility of male survivors of childhood cancer: a report from the Childhood Cancer Survivor Study. J Clin Oncol.

[12] Wasilewski-Masker K, Seidel KD, Leisenring W (2014). Male infertility in long-term survivors of pediatric cancer: a report from the childhood cancer survivor study. J Cancer Surviv.

[13] Taskinen S, Urtane A, Fagerholm R (2014). Metachronous benign ovarian tumors are not uncommon in children. J Pediatr Surg.

[14] Bellati F, Ruscito I, Gasparri ML (2014). Effects of unilateral ovariectomy on female fertility outcome Arch Gynecol Obstet.

[15] Zhai A, Axt J, Hamilton EC (2012). Assessing gonadal function after childhood ovarian surgery. J Pediatr Surg.

[16] Watanabe E, Tanaka K, Takeda N (2013). Surgical technique to prevent spillage of cyst fluid during operation for cystic ovarian tumors. Pediatr Surg Int.

[17] Martelli H, Oberlin O, Rey A (1999). Conservative treatment for girls with nonmetastatic rhabdomyosarcoma of the genital tract: a report from the Study Committee of the International Society of Pediatric Oncology. J Clin Oncol.

[18] Magné N, Oberlin O, Martelli H (2008). Vulval and vaginal rhabdomyosarcoma in children: update and reappraisal of Institut Gustave Roussy brachytherapy experience. Int J Radiat Oncol Biol Phys.

[19] Chemaitilly W, Mertens AC, Mitby P (2006). Acute ovarian failure in the childhood cancer survivor study. J Clin Endocrinol Metab.

[20] Réguerre Y, Martelli H, Rey A (2012). Local therapy is critical in localised pelvic rhabdomyosarcoma: experience of the International Society of Pediatric Oncology Malignant Mesenchymal Tumor (SIOP-MMT) committee. Eur J Cancer.

[21] Potratz J, Dirksen U, Jürgens H Ewing sarcoma: clinical state-of-the-art. Pediatr Hematol Oncol.

[22] Thibaud E, Ramirez M, Brauner R (1992). Preservation of ovarian function by ovarian transposition performed before pelvic irradiation during childhood. J Pediatr.

[23] Scott SM, Schlaff W (2005). Laparoscopic medial oophoropexy prior to radiation therapy in an adolescent with Hodgkin’s disease. J Pediatr Adolesc Gynecol.

[24] de Lambert G, Haie-Meder C, Guérin F (2014). A new surgical approach of temporary ovarian transposition for children undergoing brachytherapy: technical assessment and dose evaluation. J Pediatr Surg.

[25] Hwang JH, Yoo HJ, Park SH (2012). Association between the location of transposed ovary and ovarian function in patients with uterine cervical cancer treated with (postoperative or primary) pelvic radiotherapy. Fertil Steril.

[26] Oktay K, Harvey BE, Partridge AH (2018). Fertility preservation in patients with cancer: ASCO clinical practice guideline update. J Clin Oncol.

[27] Teinturier C (2002). Fertility in adult survivors of cancer in infancy: sex inequalities before therapy. Arch Pediatr Organe Off Soc Francaise Pediatr.

[28] Sauvat F, Binart N, Poirot C (2009). Preserving fertility in prepubertal children. Horm Res Paediatr.

[29] Ribeiro R, Rebolho JC, Tsumanuma FK (2017). Uterine transposition: technique and a case report. Fertil Steril.

[30] Vieira MA, Vieira AGS, Fonseca DSL (2021). Uterine transposition in a prepubertal patient. Int J Gynecol Cancer.

[31] Morice P, Juncker L, Rey A (2000). Ovarian transposition for patients with cervical carcinoma treated by radiosurgical combination. Fertil Steril.

[32] Wallace WHB, Anderson RA, Irvine DS (2005). Fertility preservation for young patients with cancer: who is at risk and what can be offered?. Lancet Oncol.

[33] Sauvat F, Bouilly J, Capito C (2013). Ovarian function is restored after grafting of cryopreserved immature ovary in ewes. FASEB J.

[34] Poirot C, Schubert B (2011). Fertility preservation in prepubertal children. Médecine Reprod.

[35] Donnez J, Dolmans MM, Demylle D (2004). Livebirth after orthotopic transplantation of cryopreserved ovarian tissue. Lancet.

[36] Donnez J, Dolmans MM (2015). Ovarian cortex transplantation: 60 reported live births brings the success and worldwide expansion of the technique towards routine clinical practice. J Assist Reprod Genet.

[37] Meirow D, Levron J, Eldar-Geva T (2005). Pregnancy after transplantation of cryopreserved ovarian tissue in a patient with ovarian failure after chemotherapy. N Engl J Med.

[38] Poirot C, Abirached F, Prades M (2012). Induction of puberty by autograft of cryopreserved ovarian tissue. Lancet.

[39] Demeestere I, Simon P, Dedeken L (2015). Live birth after autograft of ovarian tissue cryopreserved during childhood. Hum Reprod.

[40] Mulder RL, Font-Gonzalez A, Hudson MM (2021). Fertility preservation for female patients with childhood, adolescent, and young adult cancer: recommendations from the PanCareLIFE consortium and the International Late Effects of Childhood Cancer Guideline Harmonization Group. Lancet Oncol.

[41] Shaw JM, Bowles J, Koopman P (1996). Ovary and ovulation: fresh and cryopreserved ovarian tissue samples from donors with lymphoma transmit the cancer to graft recipients. Hum Reprod.

[42] Rosendahl M, Greve T, Andersen CY (2013). The safety of transplanting cryopreserved ovarian tissue in cancer patients: a review of the literature. J Assist Reprod Genet.

[43] Greve T, Wielenga VT, Grauslund M (2013). Ovarian tissue cryopreserved for fertility preservation from patients with Ewing or other sarcomas appear to have no tumour cell contamination. Eur J Cancer.

[44] Soares M, Saussoy P, Maskens M (2017). Eliminating malignant cells from cryopreserved ovarian tissue is possible in leukaemia patients. Br J Haematol.

[45] Agarwal PK, Palmer JS (2006). Testicular and paratesticular neoplasms in prepubertal males. J Urol.

[46] Akiyama S, Ito K, Kim WJ (2016). Prepubertal testicular tumors: a single-center experience of 44 years. J Pediatr Surg.

[47] Hofmann M, Schlegel PG, Hippert F (2013). Testicular sex cord stromal tumors: analysis of patients from the MAKEI study. Pediatr Blood Cancer.

[48] Fresneau B, Orbach D, Faure‐Conter C (2015). Sex‐cord stromal tumors in children and teenagers: results of the TGM‐95 study. Pediatr Blood Cancer.

[49] Zu’bi F, Koyle MA, Rickard M (2019). Testis-sparing surgery for pediatric Leydig cell tumors: evidence of favorable outcomes irrespective of surgical margins. Urology.

[50] Cecchetto G, De Corti F, Rogers T (2012). Surgical compliance with guidelines for paratesticular rhabdomyosarcoma (RMS) Data from the European Study on non-metastatic RMS. J Pediatr Surg.

[51] Practice Committee of the Oncofertility Consortium Installing oncofertility programs for common cancers in optimum resource settings (Repro-Can-OPEN Study Part II): a committee opinion. J Assist Reprod Genet.

[52] Kelvin JF (2017). Fertility preservation in young patients with cancer. Oncology (Williston Park).

[53] Bujons A, Sfulcini JC, Pascual M (2011). Prepubertal testicular tumours and efficacy of testicular preserving surgery. BJU Int.

[54] Henderson CG, Ahmed AA, Sesterhenn I (2006). Enucleation for prepubertal leydig cell tumor. J Urol.

[55] de Lambert G, Chargari C, Minard-Colin V (2018). Testicular transposition in children undergoing brachytherapy for bladder and/or prostate rhabdomyosarcoma. J Pediatr Surg.

[56] Schmiegelow ML, Sommer P, Carlsen E (1998). Penile vibratory stimulation and electroejaculation before anticancer therapy in two pubertal boys. J Pediatr Hematol Oncol.

[57] Brinster RL, Zimmermann JW (1994). Spermatogenesis following male germ-cell transplantation. Proc Natl Acad Sci.

[58] Sato T, Katagiri K, Gohbara A (2011). In vitro production of functional sperm in cultured neonatal mouse testes. Nature.

[59] Wu X, Goodyear SM, Abramowitz LK (2012). Fertile offspring derived from mouse spermatogonial stem cells cryopreserved for more than 14 years. Hum Reprod.

[60] Faes K, Tournaye H, Goethals L (2013). Testicular cell transplantation into the human testes. Fertil Steril.

[61] Brinster RL (2007). Male germline stem cells: from mice to men. Science.

[62] Detti L, Martin DC, Williams LJ (2012). Applicability of adult techniques for ovarian preservation to childhood cancer patients. J Assist Reprod Genet.

# Discussion: Paediatric surgical oncology and palliative care

**Published:** 2022

**Authors:** Kagiso Batka-Makwinja, Alessandro Inserra

Palliative surgical oncology is a relatively new term for an ancient problem. Adopted from the adult nomenclature, it now has the need to explode in the paediatric field. In children, unlike with adults, the focus is not on the individual patient that needs the palliative care plan but rather necessitates intense involvement of the family, caregivers and community for successful implementation. This topic is so important and delicate that it has earned the following definition from the World Health Organization (WHO): ‘*Palliative care is an approach that improves the quality of life of patients and their families facing the problem associated with life-threatening illness, through the prevention and relief of suffering by means of early identification and impeccable assessment and treatment of pain and other problems, physical, psychosocial and spiritual*’.

The etymology of the word ‘*palliative*’ is from the Latin term pallium – dress or overcoat of ancient Roma and the verb ‘*palliation*’ means to cloak or to cover. The reason to cloak or to cover a subject, could be to either hide something or to protect against something. In medical and surgical care, this coverage is not to hide but rather to protect the subject.

The discussion of palliation may seem to be a semantic or philosophical issue but it is important to get to the more correct meaning of the word, which helps us understand its clinical importance and implications. In 1967, Cicely Sunders – forerunner of palliative care said that you feel a sense of *surrender* until the last moment of your life. There are now great advancements in paediatric oncological treatment protocols. Contrary to the early interpretation of palliative, as surrendering to an unmanageable phenomenon, it is now more appropriate to interpret it as ‘protective’ care.

The first United States hospital-based palliative care consult service was developed by the Wayne State University School of Medicine in 1985 at Detroit Receiving Hospital. The first palliative medicine programme in the United States was started in 1987 by Declan Walsh, MD, at the Cleveland Clinic, Cleveland, Ohio. This is the first comprehensive integrated programme designated as a WHO international demonstration project and accredited by the European Society of Medical Oncology as an Integrated Center of Oncology and Palliative Care. Other programmes followed: The Palliative Care Program at the Medical College of Wisconsin (1993), Pain and Palliative Care Service, Memorial Sloan-Kettering Cancer Center (1996) and The Lilian and Benjamin Hertzberg Palliative Care Institute, Mount Sinai School of Medicine (1997). Since then there has been a dramatic increase in hospital-based palliative care programmes, now numbering more than 1,400. 80% of the USA hospitals with more than 300 beds have a programme at the moment.

Recent palliative care plans involve multidisciplinary protective support in social, emotional, spiritual, mental, financial and physical aspects. Modern palliative care plans are highlighted in the African philosophy of ‘*Ubuntu*’ which basically says that one’s humanity is dependent on the appreciation, preservation and affirmation of the other person’s humanity.

For many years, palliative care was not offered to paediatric patients and even today, in Europe, Asia and Africa, only a minority of children with incurable illnesses benefit or have the luxury of a dedicated palliative care service.

Paediatric oncology is seen as one of the success stories of the cancer world. The overall survival rate in developed countries is up to 80%. Survival rates in middle- and low-income countries are, however, much lower. Unfortunately, in South Africa, the survival rate is closer to 50% and even lower in other African countries. This is due to late presentation, late diagnosis, poor accessibility to treatment and management centres and a vast variation in culture, health behaviour and beliefs. This necessitates for comprehensive palliative care teams and management programmes. A dedicated, separate palliative care service is a luxury in most of Africa and other low income countries due to resource constraints. South Africa has, however, demonstrated that effective multidisciplinary palliative care that involves the family and community of the children can be successfully implemented even in resource-limited settings. Often this success is made possible by the assistance, voluntary and unstructured forms of activities and participations.

Children with life-limiting and/or life-threatening illness deserve a thorough cultural and organisational reappraisal of how we care for them when treatment is not aimed at recovery but at offering the best quality of life possible. In this respect, children with life-limiting and life-threatening conditions and their families have diverse and multiple needs:

## Clinical needs:

Managing signs and symptoms, such as pain, skin reactions, nutrition and development by using pharmacological and non-pharmacological means in an attempt to reach a balance between relief of adverse events and avoiding too many hospitalisations and/or focusing only on treating ailments and sacrificing other aspects of quality of life.

## Psychological needs:

Palliative care needs time. It cannot be rushed. It needs patient contact. It needs ‘heart’. Yes, treating the physical signs and symptoms are important, but a time will come in the management plan when the soul needs the most caring. To achieve the psychological support, there must be open and clear communication, continual emotional support to help the child, family and society to cope with the emotional issues (understanding, acceptance, anger, self-confidence, trust, love). The care plan should offer access to resources and tools that promote the development of the child’s personality, continuation of daily routines, motivation to achieve targets and realistic future goals.

## Social needs:

It is important to have recreational opportunities that are appropriate for the individual’s needs, to have the child remain an active school participant and to be an age-appropriate socially functional community member. A comprehensive paediatric oncology palliative care plan should encourage opportunities for peer-group interactions, spiritual and religious fulfilment and partaking in family and cultural aspects.

The current picture of the size of the problem (data as best collected in 2021) is illustrated in Table 1.

Numerous advancements in managing children with cancer have led to improved survival rates. This in turn means that there are more children surviving and longer survival periods. This results in more patients requiring palliative care and for longer periods of time. This again highlights the importance of establishing effective palliative care programmes for oncology children.

## The role of surgery in effective palliative care

The role of palliative surgery is to achieve the best quality of *life* for the longest possible period of time. This may involve, but is not limited to:

- to obtain correct and representative biopsies that facilitate confirming the diagnosis
- intravascular access for short- and long-term usage
- curative or tumour-debulking surgeries where beneficial
- function-sparing surgery where possible
- minimal access surgery, early or appropriately timed surgery to avoid lengthy and/or intensive chemotherapy or radiation
- procedures to aid feeding and nutrition to allow for nourishment and growth
- management of clinical signs, symptoms and adverse effects, such as soft tissue infections, bleeding and obstruction of the gut, urological system or biliary system, ascitis, pleural effusions, accesses to dialysis.

A paediatric surgical oncologist continuously battles the conflict between science and consciousness, as they try to reach a balance between the clinical and ethical aspects of palliative care. For this dilemma, there are no guidelines that can serve as a ‘one-size-fits-all’ solution. Each patient must have an individualised care plan. Personalised considerations must be made for available, feasible and beneficial surgical options.

It is more challenging to offer quality palliative care because the illness trajectory is often unpredictable. It is advisable to start introducing palliative care principles from the first diagnosis so that if the chances of cure become small, the mutual understanding between children and parents and good communication makes a rocky road easier to navigate and the grieving process less complicated. The question for the surgical management of palliative care patients remain: ‘How far is too far?’, ‘What is far enough?’ and ‘How far is not far enough?’

In 2005, the American College of Surgeons published their statement on the principles of palliative care (Table 2).

## Common physical signs in cancer children that may require surgical attention:

- Pain
- Bleeding
- Obstruction (gut, urinary, liver)
- Feeding
- Vascular access
- Fluid collections
- Intracranial hypertension
- Respiratory complication
- Infections management
- Selective intra-lesion therapies
- Tumour surgery (biopsies and resections)

**Pain** is the first and most important sign because it hinders the quality of life. Many factors apart from physical causes may influence the pain experience [1–3]. Take time to assess the pain properly. The management of pain follows a biopsychosocial approach and should always be combined with non-pharmacological methods such as biofeedback. Do not be afraid to maximise pharmacological treatment options for pain control in children with cancer [4–9]. When the mentioned options have been exhausted and residual pain that hinder quality of life remains then surgery, if feasible, may be the only available solution to alleviate the pain [9]. A multidisciplinary surgical team (e.g. surgeons, anaesthesiologists, radiologist, interventionist, nursing staff) is necessary to achieve pain relief that requires surgical inventions such as intrathecal therapy, peripheral nerve block [10–16], cryoablation, radiofrequency, neuromodulation procedures [17–27], cordotomy, midline myelotomy [28–68].

**Bleeding** can be an occasional immediate life-threatening complication in paediatric cancer patients. Consider the intention of each element of treatment in palliative care. Is it comfort care, invasive therapeutic care or both? If the quality of life may be preserved with treating the bleeding, then haemorrhage must be treated by immediate transfusion of blood products and definitive stopping of the bleed (surgery, embolisation, occlusion or complete devascularisation).

**Bowel obstruction** in children with cancer may be acute but is usually progressive in presentation. It may be due to many causes such as progression of tumour growth with a compressive effect on the gut, intra-luminally obstructing masses, a sequela of treatment with radiation therapy, previous surgery or aggressive medical treatment causing stenosis or occlusion. Intestinal obstruction will worsen the patient’s general condition and may negatively affect the performance status and thus influence the quality of life. The aim of treatment is to reduce the abdominal distention, relieve the obstruction and avoid respiratory compromise caused by increased intra-abdominal pressures. The principles of managing bowel obstruction in paediatric oncology patients is to relieve the obstruction and restore bowel continuity and/or bypass the obstruction while avoiding the complications of infection, metabolic and surgical adversities, while ensuring adequate feeding for meeting the nutritional requirements necessary for growth and development.

**Feeding** is an important aspect of a good quality of life. The immediate assistance to meet nutritional requirements may be achieved by giving total parenteral nutrition (TPN) via surgically inserted central vascular catheters. Moderate- and long-term feeding options such as naso- or orogastric feeding, gastrostomy feeding tube or feeding jejunostomy can be offered.

**Urinary obstruction/bleeding** is a symptom that requires urgent attention by urinary relief or diversion depending on the level of occlusive using transurethral catheter, supra-pubic catheter or stoma, double J stent/s or internal/external nephro-ureteral stents.

Currently there is a lack of data that indicates the true incidence of urinary obstruction in paediatric patients with advanced primary or metastatic intra-abdominal malignancies and this results in a lack of clear guidelines for the management of urinary obstruction in children with cancer. The current clinical management strategies rely on liaison amongst paediatric surgeons, urologists, radiologists, interventionists, medical oncologists and the child and their support structure. With this in question, it is of fundamental importance to remember that these interventions would form part of the palliation in patients who often have terminal illness. The child’s wishes must be considered. Again, it is difficult to reach a balance on whether the benefits outweigh the burden of the intervention in palliative care.

**Vascular access**: This is an extremely important surgical aspect of managing oncology children. Its importance is also pivotal in the paediatric oncology palliative management plan. There are two main groups of vascular access: peripheral and central. The indications of peripheral vascular lines are for intravenous fluids, medication, blood products administration and blood sampling. The surgical team is mostly relied on for the central line insertions. The role of central lines in the palliative management plan are for TPN, chemotherapy, managing the chronically ill child, long-term pharmacotherapy, emergency access, critical-care monitoring, dialysis, extra-corporeal membrane oxygenation and medication administration. Assessment must be made if the vascular access fits in with the end goals of care for each individual patient’s palliative care plan.

**Fluid collections**: Refractory ascites may need surgical intervention to avoid abdominal distension, diaphragm lifting and splinting which may result in respiratory compromise and alterations of venous return to the heart which may affect all organ systems. Fluid collections in other organ systems (chest, heart, liver, etc.) may follow and result in similar organ dysfunctions and may later result in failure of that organ system and more severely may have the sequalae of multi-organ failure. When non-operative and medical treatments such as bed rest, diet modifications drugs and fluid restriction fail, then several surgical procedures are available, if they do not impede on the child’s quality of life thereafter. Options available are drainage or shunting of the fluid to reduce the symptoms (e.g. paracentesis, pleurocentesis, cardiocentesis, chest drains, peritoneo-venous shunt, *Trans jugular Intrahepatic Porto-systemic Shunt*) and even organ transplantation if the child qualifies to be added onto the organ transplant waiting list. Every decision on surgical intervention must be tuned into the greater palliative management plan and idea of balancing the preservation of the quality of life and preparing the child and their community for a ‘good death’. Making objective and rational choices in emotionally painful and taxing situations is not easy because empathy and psychological defense mechanisms invariably come into play.

**Intracranial hypertension**: Solid organ tumours make up about 30% of tumours in children. The most common solid organ paediatric tumour is brain tumours. The most aggressive sign and symptom of central nervous system tumours is intracranial hypertension which may cause neurological symptoms such as seizures, deteriorating levels of consciousness and debilitation. Quality of life can be surgically achieved by shunting (ventriculo-peritoneal or ventriculo-atrial shunting) to reduce the intracranial pressure and where shunting is contra-indicated then drainage to reduce the pressure (external ventricular drain). These surgical interventions must be carefully decided on with consideration of all the parties involved in the child’s palliative management plan. In an effective palliative care plan, each surgical intervention must attempt balancing adequate pain and symptom control and avoid inappropriate prolongation of dying.

**Respiratory complications**: What about the question of creating a tracheostomy in a child with cancer who is on a palliative management plan? Many paediatric oncology patients could benefit from a tracheostomy when treating conditions such as space-occupying lesion in the thorax, breathing fatigue or weaning patients off a ventilator. Tracheostomy procedure may aid to achieve better comfort for the patient, more effective airway suctioning, a decrease in airway resistance, an increase in patient mobilisation, simplification in the ability to speak, achieving oral feeding all of which make home management more possible and comfortable. When deciding on such interventions involve everyone whose views count in the ethical dilemma, which in the case of a tracheostomy includes the person who will be responsible for the stoma care at home. Be absolutely fair. Give each involved person a say and try not to discriminate against anyone, especially the child.

**Infections**: The management of infections follows the same surgical principles in paediatric oncology palliative care. Infections must be treated actively to get source control (e.g. debridement, abscess drainage and wound care). Where central line associated bloodstream infections (CLABSI) are suspected then paired (peripheral and central) blood cultures could be done to assess for the possibility of CLABSI diagnosis. However, if the diagnosis of central line sepsis is confirmed, then the decision must be made on which decision in terms of removal of the line, replacement of the line or leaving the line *in-situ* will best aid in the most effective palliative care plan for the child.

**Selective intra-tumoural/intra-lesional therapies and minimally invasive surgical procedures** must not be forgotten or underestimated by the surgical team in the palliative care plan of children with cancers. A good example is using intra-arteriolar chemo-infusion performed through super-selective catheterisation of the involved area in retinoblastoma. Image guided interventions for liver metastasis are also examples. These approaches may allow for symptom treatment with the preservation of the quality of life. When the child can tolerate the anaesthesia or sedation well for these surgical procedures to be carried out, then these surgical interventions are easily agreed upon by the whole palliation care team.

Obtaining **biopsies** to confirm the diagnosis and whether to **operate resectable tumours** are also usually fairly easy decisions to conclude. Ethical conflict comes into play when needing to decide on tumour debulking and resection of malignant metastases in palliative patients. The ethical decisions must be based upon beliefs, duties of all involved parties, consequences of treating or not treating, values and the rights of the child and the closely involved parties.

Protective surgical palliative care in paediatric oncology patients is of utmost importance. The surgical principles of managing palliative children with cancer is to respect the life and health of the patient, perform to acceptable standards, maximise the benefit for the patient and minimise the harm, respect the patient’s autonomy and to act rationally, honestly, fairly and professionally. The palliative care starts at the diagnosis of the condition. Each patient must have an individualised care plan which conforms to the principles discussed. The aim of palliative care is not only to help oncological children and their families to face (and hopefully overcome) cancer as peacefully and painlessly as possible, but also, if death is unavoidable, to gently support them at end of life. ‘The good physician treats the disease; the great physician treats the patients who have the disease’ – William Osler.

## Figures and Tables

**Table 1.** The size of the problem (data as best collected in 2021).

	USA	Europe	Italy	S. Africa
Population data (millions)	321	503	61	60
New cancer diagnoses	16,000	25,071	1,500	*
Number of deaths from neoplasm	2,000	3,133	320	*

**Table 2.** American College of Surgeons’ statement of principles of palliative care. Adapted from: American College of Surgeons Committee on Ethics and the Surgical Palliative Care Task Force. Bulletin of the American College of Surgeons 2005;90(8) August.

Respect the dignity and autonomy of the patients, patients’ surrogates and caregivers.
Honour the right of the competent patient or surrogate to choose among treatments, including those that may or may not prolong life.
Communicate effectively and empathically with patients, their family, their support and caregivers.
Identify the primary goals of care from the patient’s perspective and address how the surgeon can best achieve the patient’s.
Strive to alleviate pain and other bothersome physical and nonphysical symptoms.
Recognise, assess, discuss and offer access to services for psychological, social and spiritual issues.
Provide access to therapeutic support, encompassing the spectrum from life-prolonging treatments to through to hospice care, when they can realistically be expected to improve the quality of life as perceived by the patient.
Recognise the physician’s responsibility to discourage treatments that are unlikely to achieve then patient’s goals, and encourage patients and families to consider hospice care when the prognosis for survival is likely to be less than half a year.
Arrange foe continuity of care by the patient’s primary and/or specialist physician, alleviating the sense of abandonment patients may feel when ‘curative’ therapies are no longer useful.
Maintain a collegial and supportive attitude toward others entrusted with care of the patient.

## References

[1] van den Beuken-van Everdingen MH, Rijke JM, Kessels AG, Schouten HC (2007). Prevalence of pain in patients with cancer: a systematic review of the past 40 years. Ann Oncol.

[2] Feudtner C, Kang TI, Hexem KR (2011). Pediatric palliative care patients: a prospective multicenter cohort study. Pediatrics.

[3] Daut RL, Cleeland CS (1982). The prevalence and severity of pain in cancer. Cancer.

[4] Foley KM (2011). How well is cancer pain treated?. Palliat Med.

[5] Maltoni M (2008). Opioids, pain, and fear. Ann Oncol.

[6] Wolfe J, Grier HE, Klar N (2000). Symptoms and suffering at the end of life in children with cancer. N Engl J Med.

[7] Hewitt M, Goldman A, Collins GS (2008). Opioid use in palliative care of children and young people with cancer. J Pediatr.

[8] Sirkiä K, Hovi L, Pouttu J (1998). Pain medication during terminal care of children with cancer. J Pain Symptom Manage.

[9] Goldman A, Hewitt M, Collins GS (2006). Symptoms in children/young people with progressive malignant disease: United Kingdom Children’s Cancer Study Group/Paediatric Oncology Nurses Forum survey. Pediatrics.

[10] Collins JJ, Grier HE, Sethna NF (1996). Regional anesthesia for pain associated with terminal pediatric malignancy. Pain.

[11] Aram L, Krane EJ, Kozloski LJ (2001). Tunneled epidural catheters for prolonged analgesia in pediatric patients. Anesth Analg.

[12] Amery J (2009). (London: Oxford University Press). Children’s Palliative Care in Africa.

[13] Meiring M (2012). (Hospice Palliative Care Assosciation). Paediatric Palliative Care Guidelines.

[14] Plancarte R, Patt R (1991). Intractable upper body pain in a pediatric patient relieved with cervical epidural opioid administration. J Pain Symptom Manage.

[15] Portas M, Marty JY, Buttin C (1998). Refractory pain in children with cancer: role of peridural analgesia. Arch Pediatr.

[16] Queinnec MC, Estève M, Vedrenne J (1999). Positive effect of regional analgesia (RA) in terminal stage paediatric chondrosarcoma: a case report and the review of the literature. Pain.

[17] Miralbell R, Tolnay M, Bieri S (1999). Pediatric medulloblastoma: prognostic value of p53, bcl-2, Mib-1, and microvessel density. J Neurooncol.

[18] Berg SL, Chamberlain MC (2005). Current treatment of leptomeningeal metastases: systemic chemotherapy, intrathecal chemotherapy and symptom management. Cancer Treat Res.

[19] Gwak HS, Joo J, Kim S (2013). Analysis of treatment outcomes of intraventricular chemotherapy in 105 patients for leptomeningeal carcinomatosis from non-small-cell lung cancer. J Thorac Oncol.

[20] Wasserstrom WR, Glass JP, Posner JB (1982). Diagnosis and treatment of leptomeningeal metastases from solid tumors: experience with 90 patients. Cancer.

[21] Chamberlain MC (2009). Leptomeningeal metastasis. Curr Opin Neurol.

[22] Omuro AM, Lallana EC, Bilsky MH (2005). Ventriculoperitoneal shunt in patients with leptomeningeal metastasis. Neurology.

[23] van der Ree TC, Dippel DW, Avezaat CJ (1999). Leptomeningeal metastasis after surgical resection of brain metastases. J Neurol Neurosurg Psychiatry.

[24] Perrin RG, Lishner M, Guha A (1990). Experience with Ommaya reservoir in 120 consecutive patients with meningeal malignancy. Can J Neurol Sci.

[25] Sandberg DI, Bilsky MH, Souweidane MM (2000). Ommaya reservoirs for the treatment of leptomeningeal metastases. Neurosurgery.

[26] Bokstein F, Lossos A, Siegal T (1998). Leptomeningeal metastases from solid tumors: a comparison of two prospective series treated with and without intra-cerebrospinal fluid chemotherapy. Cancer.

[27] Chowdhary S, Chamberlain M (2005). Leptomeningeal metastases: current concepts and management guidelines. J Natl Compr Canc Netw.

[28] Roebuck DJ (2014). Interventional radiology in paediatric palliative care. Pediatr Radiol.

[29] Queinnec MC, Esteve M, Vedrenne J (1999). Positive effect of regional analgesia (RA) in terminal stage paediatric chondrosarcoma: a case report and the review of the literature. Pain.

[30] Spiller W, Martin E (1912). The treatment of persistent pain of organic origin in the lower part of the body by division of the anterolateral column of the spinal cord. JAMA.

[31] Collins JJ, Grier HE, Kinney HC (1995). Control of severe pain in children with terminal malignancy. J Pediatr.

[32] Rauck RL, Cherry D, Boyer MF (2003). Long-term intrathecal opioid therapy with a patient-activated, implanted delivery system for the treatment of refractory cancer pain. J Pain.

[33] Sjöström S, Tamsen A, Persson MP (1987). Pharmacokinetics of intrathecal morphine and meperidine in humans. Anesthesiology.

[34] Deer TR, Levy R, Prager J (2012). Polyanalgesic Consensus Conference--2012: recommendations to reduce morbidity and mortality in intrathecal drug delivery in the treatment of chronic pain. Neuromodulation.

[35] Deer TR, Provenzano DA (2013). Recommendations for reducing infection in the practice of implanting spinal cord stimulation and intrathecal drug delivery devices: a physician’s playbook. Pain Physician.

[36] Wang JK, Nauss LA, Thomas JE (1979). Pain relief by intrathecally applied morphine in man. Anesthesiology.

[37] World Health Organization (1986). Cancer Pain Relief.

[38] Miser AW, Dothage JA, Wesley RA (1987). The prevalence of pain in a pediatric and young adult cancer population. Pain.

[39] Cooper MG, Keneally JP, Kinchington D (1994). Continuous brachial plexus neural blockade in a child with intractable cancer pain. J Pain Symptom Manage.

[40] Baker L, Lee M, Regnard C (2004). Evolving spinal analgesia practice in palliative care. Palliat Med.

[41] Coyne PJ, Smith T, Laird J (2005). Effectively starting and titrating intrathecal analgesic therapy in patients with refractory cancer pain. Clin J Oncol Nurs.

[42] Chambers WA (2008). Nerve blocks in palliative care. Br J Anaesth.

[43] Staats PS, Kost-Byerly S (1995). Celiac plexus blockade in a 7-year-old child with neuroblastoma. J Pain Symptom Manage.

[44] Goldschneider KR, Racadio JM, Weidner NJ (2007). Celiac plexus blockade in children using a three-dimensional fluoroscopic reconstruction technique: case reports. Regional Anesth Pain Med.

[45] Carachi R, Currie JM, Steven M (2009). New tools in the treatment of motility disorders in children. Semin Pediatr Surg.

[46] Kambadakone A, Thabet A, Gervais DA (2011). CT-guided celiac plexus neurolysis: a review of anatomy, indications, technique, and tips for successful treatment. Radiographics.

[47] Hoffer FA, Daw NC, Xiong X (2009). A phase 1/pilot study of radiofrequency ablation for the treatment of recurrent pediatric solid tumors. Cancer.

[48] Lessard AM, Gilchrist J, Schaefer L (2009). Palliation of recurrent Ewing sarcoma of the pelvis with cryoablation and somatosensory-evoked potentials. J Pediatr Hematol Oncol.

[49] Kanpolat Y, Deda H, Akyar S (1989). CT-guided percutaneous cordotomy. Acta Neurochir Suppl (Wien).

[50] Kanpolat Y, Ugur HC, Ayten M (2009). Computed tomography-guided percutaneous cordotomy for intractable pain in malignancy. Neurosurgery.

[51] Mullan S, Harper PV, Hekmatpanah J (1963). Percutaneous interruption of spinal-pain tracts by means of a Strontium90 needle. J Neurosurg.

[52] Hodge CJ, Christensen M, Burchiel KJ (2002). Anterolateral cordotomy. Surgical Management of Pain.

[53] Raslan AM, Cetas JS, McCartney S (2011). Destructive procedures for control of cancer pain: the case for cordotomy. J Neurosurg.

[54] Sindou M, Fischer G, Goutelle A (1974). Selective surgery of posterior nerve roots. First results of surgery for pain. Neurochirurgie.

[55] Nashold BS, Ostdahl RH (1979). Dorsal root entry zone lesions for pain relief. J Neurosurg.

[56] Gildenberg P, Lozano L, Tasker RR (2009). Mesenecephalotomy for cancer pain. Textbook of Stereotactic and Functional Neurosurgery.

[57] Colombo F (1984). Somatosensory-evoked potentials after mesencephalic tractotomy for pain syndromes. Neuroradiologic and clinical correlations. Surg Neurol.

[58] Tasker RR (1982). Identification of pain processing systems by electrical stimulation of the brain. Hum Neurobiol.

[59] Spiegel EA, Wycis HT (1953). Mesencephalotomy in treatment of intractable facial pain. AMA Arch Neurol Psychiatry.

[60] Spiegel EA, Wycis HT (1966). Present status of stereoencephalotomies for pain relief. Confin Neurol.

[61] Gildendberg P, Tasker R, K A (1989). Destructive central lesions for persistent pain. Textbook of Stereotactic and Functional Neurosurgery.

[62] Amery J (2016). A Really Practical Handbook of Children’s Palliative Care.

[63] Meyerson BA, Linderoth B (2000). Mechanisms of spinal cord stimulation in neuropathic pain. Neurol Res.

[64] Inserra A, Crocoli A (2020). Neuroblastoma. Clinical and Surgical Management.

[65] Inserra A, Crocoli A (2019). Palliative care: a surgical perspective. Pediatr Blood and Cancer.

[66] Berde CB, Fischel N, Filardi JP (1989). Caudal epidural morphine analgesia for an infant with advanced neuroblastoma: report of a case. Pain.

[67] Tsui BC, Berde CB (2005). Caudal analgesia and anesthesia techniques in children. Curr Opin Anaesthesiol.

[68] Steel TR, Burchiel KJ (2002). Neurosurgical techniques in the treatment of chronic pain. Pain: Overview.
